# Perioperative durvalumab plus chemotherapy plus new agents for resectable non-small-cell lung cancer: the platform phase 2 NeoCOAST-2 trial

**DOI:** 10.1038/s41591-025-03746-z

**Published:** 2025-05-31

**Authors:** Tina Cascone, Laura Bonanno, Florian Guisier, Amelia Insa, Moishe Liberman, Olivier Bylicki, Lorenzo Livi, Thomas Egenod, Romain Corre, Dong-Wan Kim, Maria Rosario Garcia Campelo, Mariano Provencio Pulla, Byoung Yong Shim, Giulio Metro, Jaafar Bennouna, Agata A. Bielska, Alula R. Yohannes, Yun He, Adam Dowson, Gozde Kar, Lara McGrath, Rakesh Kumar, Italia Grenga, Jonathan Spicer, Patrick M. Forde

**Affiliations:** 1 Department of Thoracic/Head and Neck Medical Oncology, The University of Texas MD Anderson Cancer Center, Houston, TX USA; 2 Department of Surgery, Oncology and Gastroenterology, University of Padova, Padova, Italy; 3 Medical Oncology 2, Istituto Oncologico Veneto IRCCS, Padova, Italy; 4 Univ Rouen Normandie, LITIS Lab QuantIF team EA4108, CHU Rouen, Department of Pneumology and Inserm CIC-CRB 1404, Rouen, France; 5 Medical Oncology Department, Hospital Clínico Universitario de Valencia, Valencia, Spain; 6 Division of Thoracic Surgery, University of Montréal, Montréal, Quebec Canada; 7 CETOC - CHUM Endoscopic Tracheobronchial and Oesophageal Center, Centre Hospitalier de l’Université de Montréal, Montreal, Quebec Canada; 8 Pneumology Department, Hôpital d’Instruction des Armées Sainte-Anne, Toulon, France; 9 Department of Radiation Oncology, University of Florence, Florence, Italy; 10 Department of Thoracic Oncology, Dupuytren University Hospital, Limoges, France; 11 Department of Medical Oncology, CH de Cornouaille, Quimper, France; 12 Department of Internal Medicine, Seoul National University College of Medicine and Seoul National Hospital, Seoul, South Korea; 13 Hospital de A Coruña, Sergas, A Coruña, Spain; 14 Puerta de Hierro University Hospital, Majadahonda, Spain; 15 Department of Medical Oncology, The Catholic University of Korea, St. Vincent’s Hospital, Seoul, South Korea; 16 Santa Maria della Misericordia Hospital, University of Perugia, Perugia, Italy; 17 Department of Medical Oncology, Hôpital Foch, Suresnes, France; 18 AstraZeneca, Waltham, MA USA; 19 AstraZeneca, Gaithersburg, MD USA; 20 AstraZeneca, Cambridge, UK; 21 Department of Thoracic Surgery, McGill University, Montreal, Quebec Canada; 22 Trinity St. James’s Cancer Institute, Trinity College Dublin, Dublin, Ireland

**Keywords:** Immunotherapy, Non-small-cell lung cancer

## Abstract

In the phase II NeoCOAST-2 platform study, 202 patients with untreated, resectable stage IIA–IIIB non-small-cell lung cancer (NSCLC) were randomized to receive neoadjuvant durvalumab plus platinum-doublet chemotherapy with oleclumab, a CD73 inhibitor (Arm 1), or with monalizumab, a NKG2A inhibitor (Arm 2), or neoadjuvant durvalumab plus single-agent platinum chemotherapy with the TROP-2 antibody–drug conjugate (ADC) datopotamab deruxtecan (Arm 4), followed by surgical resection and adjuvant durvalumab with oleclumab or monalizumab (Arms 1 and 2) or durvalumab alone (Arm 4). Primary endpoints were pathological complete response (pCR) rate and safety; secondary endpoints included feasibility of surgery and major pathological response (mPR) rate. In the modified intention-to-treat population (*n* = 198; Arm 1, *n* = 74; Arm 2, *n* = 70; Arm 4, *n* = 54), pCR rates were 20.3% (15/74; 95% CI, 11.8–31.2), 25.7% (18/70; 95% CI, 16.0–37.6) and 35.2% (19/54; 95% CI, 22.7–49.4), and mPR rates were 41.9% (31/74; 95% CI, 30.5–53.9), 50.0% (35/70; 95% CI, 37.8–62.2) and 63.0% (34/54; 95% CI, 48.7–75.7) in arms 1, 2, and 4, respectively. In the safety population, 69/74 (93.2%), 66/71 (93.0%), and 51/54 (94.4%) patients underwent surgery, respectively. Overall, grade ≥3 treatment-related adverse events occurred in 27/74 (36.5%), 29/71 (40.8%) and 11/54 (20.4%) patients, respectively. In NeoCOAST-2, the first neoadjuvant trial examining an ADC plus chemo-immunotherapy in resectable NSCLC, pCR rates were highest in the datopotamab-deruxtecan-containing arm, warranting further investigation in larger trials of ADCs and checkpoint inhibition in the neoadjuvant setting. ClinicalTrials.gov identifier: NCT05061550.

## Abstract (web summary)

As presented at the 2025 ASCO Annual Meeting: in a randomized platform phase II trial, high rates of pathologic complete response were seen for neoadjuvant durvalumab plus chemotherapy when combined with new agents, most notably a TROP2-targeting antibody–drug conjugate, in patients with resectable non-small-cell lung cancer.

## Main

NSCLC accounts for most lung cancer diagnoses^1^. Approximately 25% of patients with NSCLC initially present with early-stage disease, and that number is expected to grow owing to updated screening guidelines^1–3^. Treatment of early-stage NSCLC, which is potentially curable, has traditionally relied on surgery as the primary curative-intent approach, with or without neoadjuvant or adjuvant chemotherapy^4^. Despite these interventions, 30–55% of patients experience disease recurrence, leading to cancer-related morbidity and ultimately mortality^4,5^.

Recent advances have broadened the standard of care (SoC) to include neoadjuvant or perioperative programmed cell death-1 (PD-1) or PD-ligand 1 (PD-L1) immune checkpoint inhibitors combined with neoadjuvant chemotherapy, which enhance clinical benefits compared with neoadjuvant chemotherapy alone^6–9^. In the phase III AEGEAN study, perioperative durvalumab plus neoadjuvant chemotherapy led to significantly higher rates of pCR (17.2% versus 4.3%; *P* < 0.001) and significantly longer event-free survival (EFS; hazard ratio 0.68; 95% confidence interval (CI), 0.53–0.88) compared with neoadjuvant chemotherapy alone in eligible patients with resectable stage IIA–IIIB NSCLC^6^. Surgery was completed for 77.6% of patients in the durvalumab group and 76.7% in the placebo group; of these, 94.7% and 91.3% had complete resection (R0), respectively, and 65.8% and 63.4% of patients in the entire study population started adjuvant durvalumab or placebo, respectively. The safety profile of the combination was consistent with those of the individual agents^6^. Similar findings were reported in the phase III KEYNOTE-671, CheckMate 77T and CheckMate 816 studies^7–9^. These trials highlight the importance of pCR as a potential early predictor of survival, given the demonstrated correlation between pCR and improved EFS seen across trials.

Despite recent advances, a minority of patients receiving perioperative or neoadjuvant treatment have a pCR, and the 3-year EFS rates remain around 54–60% (refs. ^6–9^), highlighting the high risk of relapse and disease-related mortality^7,8^. The persistent unmet need in this patient population underscores the importance of developing innovative therapies and new combinations to further improve long-term outcomes for individuals with resectable NSCLC.

NeoCOAST-2 (NCT05061550) is a phase II, open-label, multiarm, multicenter, global, randomized platform study of perioperative durvalumab in combination with novel agents, including oleclumab, monalizumab and datopotamab deruxtecan (Dato-DXd), and chemotherapy. Here we report results from three arms.

Durvalumab is a selective, high-affinity human IgG1 monoclonal antibody that inhibits the interaction of PD-L1 with PD-1 and CD80 by binding to PD-L1. This increases T cell activation, enhances tumor-cell detection and enables tumor-cell killing^10^. Oleclumab is a human IgG1 monoclonal antibody that selectively binds and inhibits CD73, thereby inhibiting the catalysis of adenosine monophosphate to inorganic phosphate and adenosine, which is believed to mediate multiple immunosuppressive effects^11^. CD73 inhibition reduces extracellular adenosine production and promotes antitumor immunity^11^. Monalizumab is a first-in-class, humanized, IgG4 monoclonal antibody that blocks the inhibitory receptor NKG2A, expressed on natural killer (NK) and CD8^+^ T cells, from binding to major histocompatibility complex E (HLA-E) on tumor cells, which reduces the inhibition of these effector cells and enhances antitumor immunity^12^. In the phase II NeoCOAST study, neoadjuvant durvalumab combined with oleclumab or monalizumab led to numerical improvement in mPR rates and increased pro-inflammatory effects in the tumor microenvironment compared with durvalumab alone in patients with resectable stage IA3–IIIA NSCLC^13^. The safety profiles of the combination regimens were generally similar to that of durvalumab alone^13^. Dato-DXd is an ADC composed of a humanized trophoblast cell surface antigen 2 (TROP2)-directed monoclonal antibody covalently linked to DXd, a topoisomerase 1 inhibitor (TOP1i) payload, through a plasma-stable, tumor-selective, cleavable linker^14^. After binding to TROP2, the ADC is internalized and the payload is released into the target cell, inducing DNA damage and cell death^14^. In the phase III TROPION-Lung01 study, Dato-DXd significantly improved progression-free survival compared with docetaxel in patients with previously treated, locally advanced or metastatic NSCLC^15^.

Here, we report final pCR data from a central blinded independent pathology review and safety data (data cutoff, 19 December 2024) for the neoadjuvant and postsurgery periods, along with surgical feasibility and interim adjuvant safety data for the above-mentioned three arms of the NeoCOAST-2 trial.

## Results

### Trial design

**Extended Data Fig. 1:**
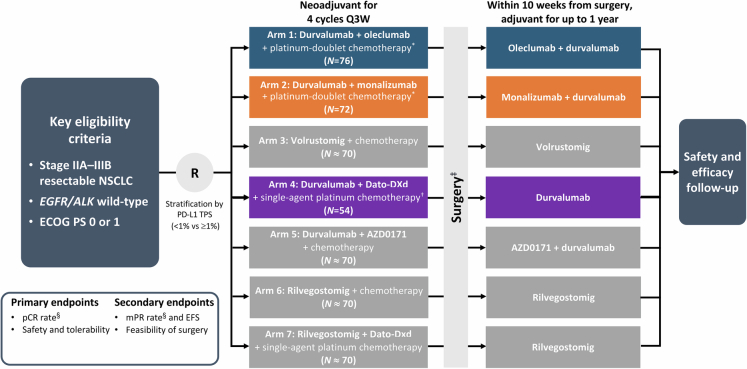
Study design. Arms shown in gray are ongoing and will be reported at a later date. *Carboplatin + paclitaxel for squamous tumor histology, pemetrexed + cisplatin or carboplatin for non-squamous tumor histology. ^†^Physician’s choice of carboplatin or cisplatin. ^‡^Within 40 days of the last dose of neoadjuvant treatment. ^§^Proportion of patients with no viable tumor cells (pCR) or ≤10% residual viable tumor cells (mPR) in resected tumor specimen and sampled nodes at surgery (primary tumor only for mPR). ALK, anaplastic lymphoma kinase; Dato-DXd, datopotamab deruxtecan; ECOG PS, Eastern Cooperative Oncology Group Performance Status; EGFR, epidermal growth factor receptor; IASLC, International Association for the Study of Lung Cancer; PD-L1, programmed cell death ligand-1; Q3W, every 3 weeks; TPS, tumor proportion score.

NeoCOAST-2 is a phase II, open-label, multiarm, multicenter, global, randomized platform study. For the results reported here, patients were randomized into three arms. Those in Arm 1 received neoadjuvant durvalumab, oleclumab and platinum-doublet chemotherapy (Extended Data Table 1), followed by surgery and adjuvant durvalumab and oleclumab. Arm 2 received neoadjuvant durvalumab, monalizumab and platinum-doublet chemotherapy, followed by surgery and adjuvant durvalumab and monalizumab. Arm 4 received neoadjuvant durvalumab, Dato-DXd and single-agent platinum chemotherapy, followed by surgery and adjuvant durvalumab (Extended Data Fig. 1). Results from Arms 3, 5, 6 and 7 are not included here and will be reported separately. Patients with stage IIA–IIIB resectable NSCLC (described by the International Association for the Study of Lung Cancer (IASLC) (ref. ^5^)) with no sensitizing epidermal growth factor receptor (*EGFR*) mutations or anaplastic lymphoma kinase (*ALK*) translocations were eligible for the study. The primary endpoints of the study were the pCR rate, defined as the proportion of patients without any viable tumor cells after complete evaluation of the resected lung cancer specimen and all sampled regional lymph nodes, as determined by a central blinded independent pathology review and described by the IASLC 2020 (ref. ^16^); and the safety of perioperative treatment. Key secondary endpoints included the mPR rate, defined as the proportion of patients with resected lung cancer specimens with ≤10% viable tumor cells; feasibility of surgery; the clearance of circulating tumor DNA (ctDNA) while on treatment before surgery; EFS; and overall survival. Exploratory endpoints included, among others, tissue- and blood-based biomarker analyses, and will be reported at a later date.

### Patients

At the 19 December 2024 data cutoff, 202 patients were randomized to three arms: 76 to durvalumab plus oleclumab plus chemotherapy (Arm 1), 72 to durvalumab plus monalizumab plus chemotherapy (Arm 2) and 54 to durvalumab plus Dato-DXd plus chemotherapy (Arm 4). Enrollment in Arms 1, 2 and 4 occurred from 14 April 2022 to 4 March 2024; from 28 July 2022 to 1 March 2024; and from 3 August 2023 to 23 January 2024, respectively. These patients were defined as the intention-to-treat (ITT) population. The modified ITT (mITT) population included 198 randomized patients with confirmed NSCLC histology who received at least one dose of the study treatment: of the four patients in the ITT population who were excluded from the mITT population, two were randomized in error after not meeting the clinical study protocol requirements and did not receive treatment, one was randomized but was not treated owing to medical complications and one patient was treated but was later confirmed to not have NSCLC. The safety population included all 199 treated patients.

Baseline characteristics and patient demographics for the mITT population are shown in Table 1 and for the ITT population in Extended Data Table 2. Approximately half of the study population had stage IIIA disease and approximately two-thirds had a PD-L1 tumor proportion score (TPS) ≥ 1%. Although this is a non-comparative study, patient characteristics were generally well balanced across the three arms.

**Table 1:** Patient demographics and baseline disease characteristics of the mITT population

	Arm 1	Arm 2	Arm 4
	Durvalumab + oleclumab + CT	Durvalumab + monalizumab + CT	Durvalumab +Dato-DXd + Plt
	*n* = 74	*n* = 70	*n* = 54
Age			
Median (range), years	66.5 (30–79)	66.0 (48–83)	65.0 (38–81)
<65 years, *n* (%)	28 (37.8)	30 (42.9)	25 (46.3)
≥65 years, *n* (%)	46 (62.2)	40 (57.1)	29 (53.7)
Sex, *n* (%)			
Female	28 (37.8)	28 (40.0)	22 (40.7)
Male	46 (62.2)	42 (60.0)	32 (59.3)
Smoking status, *n* (%)			
Current	15 (20.3)	22 (31.4)	15 (27.8)
Former	55 (74.3)	46 (65.7)	36 (66.7)
Never smoked	4 (5.4)	2 (2.9)	3 (5.6)
Race, *n* (%)			
Asian	7 (9.5)	5 (7.1)	5 (9.3)
Black or African American	1 (1.4)	0	0
White	46 (62.2)	42 (60.0)	37 (68.5)
Not reported	20 (27.0)	23 (32.9)	12 (22.2)
ECOG PS, *n* (%)^a^			
0	45 (61.6)	48 (68.6)	35 (64.8)
1	28 (38.4)	22 (31.4)	19 (35.2)
Missing	1	0	0
PD-L1 TPS, *n* (%)^b^			
<1%	25 (33.8)	28 (40.0)	16 (29.6)
≥1%	49 (66.2)	42 (60.0)	38 (70.4)
1–49%	20 (27.0)	18 (25.7)	21 (38.9)
≥50%	29 (39.2)	24 (34.3)	17 (31.5)
Planned platinum agent, *n* (%)			
Cisplatin	21 (28.4)	15 (21.4)	7 (13.0)
Carboplatin	53 (71.6)	55 (78.6)	47 (87.0)
Primary tumor, *n* (%)^c^			
T1	1 (1.4)	3 (4.3)	0
T1a	0	0	0
T1b	3 (4.1)	5 (7.1)	2 (3.7)
T1c	4 (5.4)	2 (2.9)	5 (9.3)
T2	7 (9.5)	5 (7.1)	3 (5.6)
T2a	3 (4.1)	2 (2.9)	2 (3.7)
T2b	12 (16.2)	13 (18.6)	10 (18.5)
T3	28 (37.8)	22 (31.4)	18 (33.3)
T4	16 (21.6)	18 (25.7)	14 (25.9)
Regional lymph nodes, *n* (%)^d^			
N0	25 (33.8)	24 (34.3)	17 (31.5)
N1	17 (23.0)	19 (27.1)	10 (18.5)
N2	32 (43.2)	27 (38.6)	27 (50.0)
Single station	21 (28.4)	17 (24.3)	18 (33.3)
Multi-station	11 (14.9)	10 (14.3)	9 (16.7)
Distant metastases, *n* (%)			
M0	74 (100)	70 (100)	54 (100)
Stage, *n* (%)^e^			
IIA	7 (9.5)	8 (11.4)	2 (3.7)
IIB	15 (20.3)	17 (24.3)	13 (24.1)
IIIA	38 (51.4)	32 (45.7)	28 (51.9)
IIIB	14 (18.9)	13 (18.6)	11 (20.4)
Histology, *n* (%)			
Squamous cell carcinoma	23 (31.1)	19 (27.1)	17 (31.5)
Adenocarcinoma	49 (66.2)	46 (65.7)	33 (61.1)
Other^f^	2 (2.7)	5 (7.1)	4 (7.4)

Data are shown for the mITT population, which includes all randomized patients with confirmed NSCLC histology who received at least one dose of study treatment. ^a^Percentages were calculated with the number of patients with reported performance status as the denominator. ^b^Central results in the mITT population were reported for 70.3%, 75.7% and 63.0% of patients in Arms 1, 2 and 4, respectively. ^c^T1–T4 refers to the size and extent of the primary tumor, with T1 indicating a smaller tumor and T4 indicating a larger, more advanced tumor. ^d^N0–N2 refers to the extent of lymph node involvement in the TNM staging system. ^e^The stages were described by the International Association for the Study of Lung Cancer. ^f^Arm 1: carcinoma, type not determined (*n* = 2); Arm 2: carcinoma, type not determined (*n* = 1), other (*n* = 4); Arm 4: carcinoma, type not determined (*n* = 1), large cell carcinoma (*n* = 2), other (*n* = 1).

CT, platinum-doublet chemotherapy; ECOG PS, Eastern Cooperative Oncology Group performance status; Plt, single-agent platinum chemotherapy; TNM, tumor node metastasis.

### Neoadjuvant treatment exposure

**Fig. 1:**
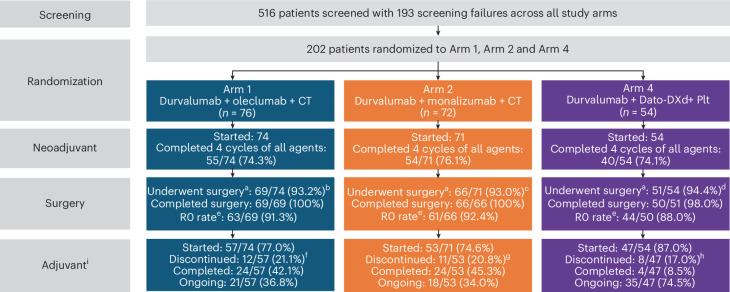
CONSORT diagram and summary of patient disposition. The median (and range) numbers of adjuvant cycles completed in Arms 1, 2 and 4 were 9 (1–12), 10 (1–12) and 7 (1–12), respectively. ^a^The denominator includes patients from the safety population. ^b^The reasons for not having surgery (and the number of patients for each reason) were as follows: AE, 1 (grade 3 renal infarct); PD, 2; study discontinuation, 1; death, 1 (intestinal ischemia and septic shock). ^c^The reasons for not having surgery (and the number of patients for each reason) were as follows: AE, 2 (1 patient experienced grade 4 mediastinal abscess; 1 patient experienced grade 3 syncope); investigator or patient decision, 3. ^d^The reasons for not having surgery (and the number of patients for each reason) were as follows: investigator or patient decision, 3. ^e^Surgical margins are calculated for patients who completed surgery. ^f^The reasons for discontinuation of study treatment (and the number of patients for each reason) were as follows: AE, 3; PD, 6; other, 3. ^g^The reasons for discontinuation of study treatment (and the number of patients for each reason) were as follows: AE, 5; PD, 4; other, 2. ^h^The reasons for discontinuation of study treatment (and the number of patients for each reason) were as follows: AE, 2; PD, 3; other, 3. ^i^The reasons for not starting adjuvant therapy after receiving surgery (and the number of patients for each reason) were as follows. In Arm 1: AE, 3 (1 patient each owing to grade 3 empyema, grade 3 pneumonia and grade 2 asthenia); PD, 3; investigator or patient decision, 4; death, 2 (1 due to pneumonia and 1 due to atrial fibrillation). In Arm 2: AE, 5 (1 each owing to grade 3 psoriasis, grade 3 anaphylaxis and grade 3 eczema; 1 owing to grade 2 arthralgia and grade 2 asthenia; 1 owing to grade 2 renal disorder, grade 2 rash, grade 2 alanine aminotransferase (ALT) increase, grade 4 neutrophil count decrease and grade 2 C-reactive protein increase); PD, 1; patient decision, 2; death, 3 (1 due to sepsis, 1 due to septic shock and 1 due to postoperative renal failure); other, 2. In Arm 4: AE, 1 (grade 2 colitis); death, 1 (Idiopathic pulmonary fibrosis); other, 2. *n* = 202 randomized patients, *n* = 199 patients in the safety population and *n* = 198 in mITT population. PD, progressive disease.

At the data cutoff, all patients in Arms 1, 2 and 4 had the opportunity to complete neoadjuvant treatment and receive surgery and had completed, discontinued or were still receiving treatment in the adjuvant period. In the safety population, four neoadjuvant cycles were received per protocol (all agents) by 74.3%, 76.1% and 74.1% of patients in Arms 1, 2 and 4, respectively (Fig. 1); 87.8%, 84.5% and 92.6% of patients received at least 3 neoadjuvant cycles per protocol (all agents) in each respective arm. In Arm 1, 23.0% of patients had discontinued oleclumab and 25.4% had discontinued monalizumab in Arm 2 before starting adjuvant treatment. In Arm 4, 18.5% had discontinued neoadjuvant Dato-DXd. The most common reason for discontinuing oleclumab, monalizumab or Dato-DXd before starting adjuvant treatment was adverse events (AEs), which occurred in 6.8%, 9.9% and 7.4% of patients, respectively. Carboplatin was started in 71.6%, 77.5% and 87.0% of patients in Arms 1, 2 and 4, respectively.

### Surgery

Overall, 93.2%, 93.0% and 94.4% of patients in the safety population in Arms 1, 2 and 4, respectively, underwent surgery (Fig. 1 and Extended Data Table 3). The median number of days from the last neoadjuvant dose to surgery was 34 (range, 19–75), 34 (range, 21–119) and 36 (range, 20–162) in Arms 1, 2 and 4, respectively. One patient in Arm 4 did not complete surgery because of scar tissue that prevented completion of the sleeve lobectomy. Most patients who completed surgery had R0 resections (Fig. 1 and Extended Data Table 3). Across all three arms, 13 patients did not undergo surgery: 6 owing to investigator or patient decision (*n* = 3 in Arm 2; *n* = 3 in Arm 4), 3 owing to AEs (*n* = 1 in Arm 1; *n* = 2 in Arm 2), 2 owing to disease progression (both in Arm 1), 1 owing to study discontinuation (Arm 1) and 1 owing to death from intestinal ischemia and septic shock (Arm 1). Among patients who underwent surgery, surgical delays occurred for 14.5%, 18.2% and 27.5% of patients in Arms 1, 2, and 4, respectively (Extended Data Table 4). The most commonly reported reasons were logistical or scheduling issues and instances in which the reason was not reported. Among patients with surgical delays, the majority experienced delays of <4 weeks. Two patients faced delays of >8 weeks; one was due to workup for surgical eligibility and patient decision in Arm 2, and one was due to grade 3 stroke in Arm 4 (Extended Data Table 4). Surgical complications, classified according to the Clavien–Dindo system, occurred in 36.2%, 30.3% and 29.4% of patients in Arms 1, 2 and 4, respectively; grade ≥3 surgical complications occurred in 13.0%, 9.1% and 3.9% of patients in the respective arms (Extended Data Table 3). Among patients who completed surgery (as determined by the investigator), lobectomy was the most common surgical procedure across all arms. The next most common procedures were bilobectomy and pneumonectomy (Extended Data Table 3). Although planned pneumonectomy at screening was an exclusion criterion, pneumonectomy after neoadjuvant treatment was allowed following surgical re-evaluation. The median number of days from surgery to the first dose of adjuvant therapy were 55 (range, 31–143), 54 (range, 23–140) and 50 (range, 32–133) days in Arms 1, 2 and 4, respectively.

### Efficacy

**Fig. 2:**
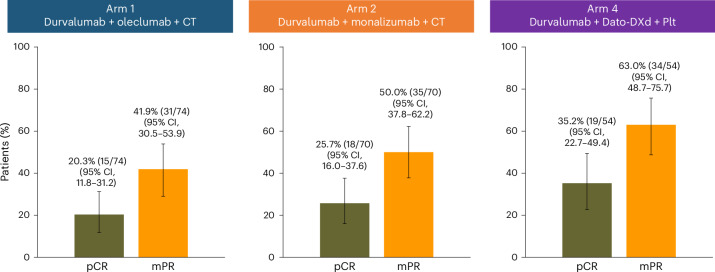
pCR and mPR rates across treatment arms in the mITT population. The proportion of patients with pCR or mPR for Arm 1 (*n* = 74), Arm 2 (*n* = 70) and Arm 4 (*n* = 54) in the mITT population. Data are presented as percentages, with error bars showing the 95% CI around the observed proportion of patients in the treatment arm. The CIs for each treatment arm were calculated using the Clopper–Pearson method. The number of patients with pCR or mPR and the total number of patients in the mITT population in each treatment arm are shown above each bar. The mITT population includes all randomized patients with confirmed NSCLC histology who received at least one dose of study treatment.

**Fig. 3:**
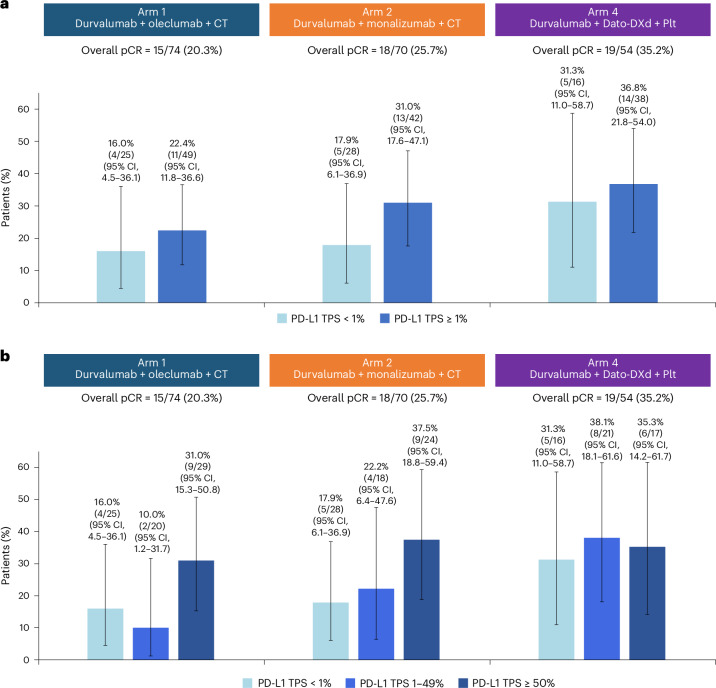
pCR rates across baseline PD-L1 tumor proportion scores and in the mITT population. **a**, pCR rates in patients in the mITT population with a baseline PD-L1 tumor proportion score of <1% (Arm 1, *n* = 25; Arm 2, *n* = 28; Arm 4, *n* = 16) or ≥1% (Arm 1, *n* = 49; Arm 2, *n* = 42; Arm 4, *n* = 38). **b**, pCR rates in patients in the mITT population with a baseline tumor proportion score of <1% (Arm 1, *n* = 25; Arm 2, *n* = 28; Arm 4, *n* = 16), 1-49% (Arm 1, *n* = 20; Arm 2, *n* = 18; Arm 4, *n* = 21) and ≥50% (Arm 1, *n* = 29; Arm 2, *n* = 24; Arm 4, *n* = 17). Data are presented as percentages with error bars showing the 95% CI around the observed proportion of patients in the subgroup. The CIs for each subgroup were calculated using the Clopper–Pearson method. The number of patients with pCR and the total number of patients in each subgroup are shown above each bar. The mITT population includes all randomized patients with confirmed NSCLC histology who received at least one dose of study treatment. Baseline PD-L1 status was assessed using central (VENTANA SP263) or local testing (VENTANA SP263, pharmDx 28-8, or pharmDx 22C3). Central results in the mITT population were reported for 70.3%, 75.7% and 63.0% of patients in Arms 1, 2 and 4, respectively.

**Extended Data Fig. 2:**
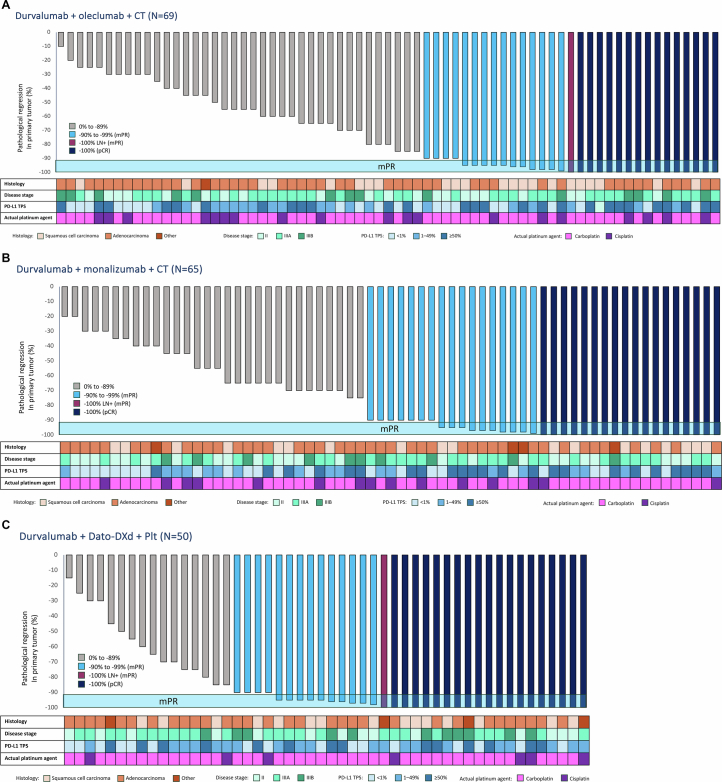
Pathological regression in primary tumor. (**a**,**b**,**c**) Pathological regression in primary tumor assessed by central blinded independent pathology review in patients in the mITT population with evaluable %RVT in (**a**) Arm 1, (**b**) Arm 2, and (**c**) Arm 4. Data cutoff: December 19, 2024. Pathological regression in primary tumor is defined as %RVT -100%. CT, platinum-doublet chemotherapy; Dato-DXd, datopotamab deruxtecan; LN, lymph node; mITT, modified intention-to-treat population; mPR, major pathological response; pCR, pathological complete response; PD-L1, programmed cell death ligand-1; Plt, single agent platinum chemotherapy; RVT, residual viable tumor.

All efficacy endpoints are reported for the mITT population (*n* = 198), which included all randomized patients with confirmed NSCLC histology who received at least one dose of the study intervention. At the final analysis of pathological response, pCR rates were 20.3% (95% CI, 11.8–31.2) in Arm 1, 25.7% (95% CI, 16.0–37.6) in Arm 2 and 35.2% (95% CI, 22.7–49.4) in Arm 4 (Fig. 2 and Extended Data Fig. 2a–c). pCR rates for the ITT population are shown in Extended Data Table 5. In Arms 1 and 2, pCR rates observed in patients with PD-L1 expression ≥ 1% were numerically higher than those in patients with PD-L1 < 1% (Fig. 3a and Extended Data Table 6). In Arms 1 and 2, pCR rates were highest in tumors with PD-L1 expression ≥ 50%, whereas in Arm 4, pCR rates were similar in the PD-L1 < 1%, 1–49% and ≥50% subgroups (Fig. 3b and Extended Data Table 6). Across all histological subtypes and treatment arms, pCR rates were numerically higher in those with squamous cell carcinoma than in those with adenocarcinoma (Extended Data Table 6).

In the final analysis of the mITT population, the mPR rates were 41.9% (95% CI, 30.5–53.9) in Arm 1, 50.0% (95% CI, 37.8–62.2) in Arm 2 and 63.0% (95% CI, 48.7–75.7) in Arm 4 (Fig. 2 and Extended Data Fig. 2a–c). The mPR rates for the ITT population are provided in Extended Data Table 5. Similar trends to the pCR rates were observed, with Arms 1 and 2 exhibiting numerically higher mPR rates in patients with PD-L1 expression ≥ 1%. In Arms 1 and 2, the mPR rates were highest in patients with PD-L1 expression ≥ 50%; in Arm 4, the mPR rates were similar in the PD-L1 <1%, 1–49% and ≥50% subgroups (Extended Data Table 6). Across histological subtypes, similar trends emerged: mPR rates were numerically higher in those with squamous cell carcinoma than in those with adenocarcinoma (Extended Data Table 6). Among those who completed surgery, patients in Arm 4 had the lowest median percentage of residual viable tumor (%RVT) at 4.5% (interquartile range (IQR), 0–25.0), with a numerically higher median %RVT of 20.0% (IQR, 2.0–45.0) in Arm 1 and 10.0% (IQR, 0–35.0) in Arm 2. The objective response rate, assessed by the Response Evaluation Criteria in Solid Tumours v1.1, is detailed in Extended Data Table 7.

### Adjuvant treatment exposure

In the safety population, 77.0%, 74.6% and 87.0% of patients began adjuvant treatment in Arms 1, 2 and 4, respectively (Fig. 1). Arms 1 and 2 opened for enrollment before Arm 4, resulting in a greater proportion of patients on those arms having the opportunity to complete adjuvant treatment. At the data cutoff, 36.8%, 34.0% and 74.5% of patients who started adjuvant treatment were still receiving it in Arms 1, 2 and 4, respectively, and 42.1%, 45.3% and 8.5% of patients, respectively, completed adjuvant treatment. Of the patients who started adjuvant treatment, 21.1%, 20.8% and 17.0% discontinued any agent in Arms 1, 2 and 4, respectively, by the data cutoff (Fig. 1). The most common reasons for discontinuing any adjuvant therapy were AEs (5.3% in Arm 1; 9.4% in Arm 2; 4.3% in Arm 4) and disease progression (10.5% in Arm 1; 7.5% in Arm 2; 6.4% in Arm 4). Among those who started adjuvant treatment, delays to first treatment (defined as the first adjuvant dose administered more than 10 weeks from the surgery date) occurred in 7.0%, 13.2% and 6.4% of patients in Arms 1, 2 and 4, respectively, with AEs being the most common cause.

### Safety

**Fig. 4:**
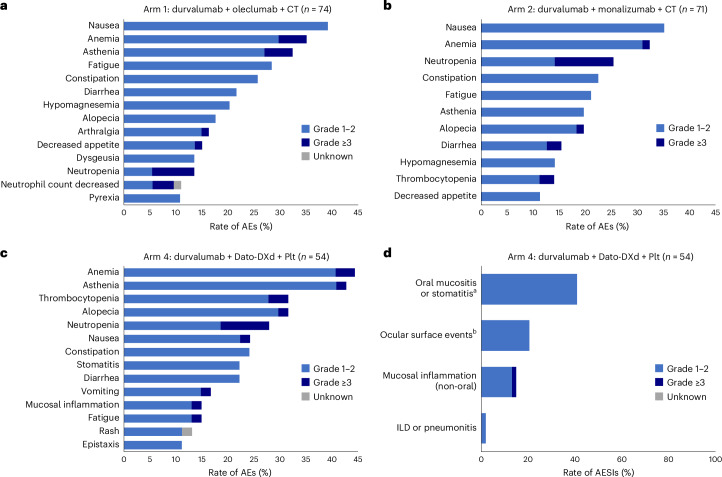
Any-grade treatment-emergent adverse events in ≥10% of patients in Arms 1, 2 and 4, and rates of adverse events of special interest in Arm 4 in the neoadjuvant period (safety population). **a**–**c**, Any-grade TEAEs in ≥10% of patients in the neoadjuvant period in Arm 1 (**a**), Arm 2 (**b**) and Arm 4 (**c**). **d**, Rates of adverse events of special interest (AESIs) in the neoadjuvant period in Arm 4. Data are shown for the safety population, which consisted of all patients who received at least one dose of any study intervention. Only the neoadjuvant period is shown owing to the maturity of the data. Patients with multiple occurrences in the same category were counted once per category regardless of the number of occurrences. ^a^This grouped term includes mouth ulceration, odynophagia, oral pain, oropharyngeal pain, pharyngeal inflammation and stomatitis. ^b^This grouped term includes conjunctivitis, dry eye, keratitis, lacrimation increased and ocular toxicity.

Safety profiles are reported overall across the study, as well as by treatment period (neoadjuvant, postsurgery and adjuvant) in the safety population (Table 2). As of the data cutoff, the median duration of safety follow-up was 12.3 months (range, 1.2–20.2) in Arm 1, 10.4 months (range, 1.0–20.1) in Arm 2 and 11.2 months (range, 3.5–15.7) in Arm 4. Overall, AEs of any cause occurred in 100%, 98.6% and 100% of patients in Arms 1, 2 and 4, respectively, and AEs that were possibly related to durvalumab occurred in 83.8% of patients in Arm 1, 70.4% of patients in Arm 2 and 87.0% of patients in Arm 4. AEs that were possibly related to oleclumab, monalizumab or Dato-DXd occurred in 82.4%, 74.6% and 83.3% of patients in Arms 1, 2 and 4, respectively. The overall incidence of grade ≥3 AEs was 51.4% in Arm 1, 63.4% in Arm 2 and 40.7% in Arm 4, with 36.5%, 42.3% and 24.1% of patients in the respective arms experiencing these events in the neoadjuvant period. The overall incidence of grade ≥3 AEs that were possibly related to durvalumab was 20.3%, 25.4% and 16.7% in Arms 1, 2 and 4, respectively, with 14.9%, 15.5% and 14.8% of patients in the respective arms experiencing these events in the neoadjuvant period. Overall, grade ≥3 AEs possibly related to oleclumab, monalizumab or Dato-DXd occurred in 18.9%, 26.8% and 16.7% patients in Arms 1, 2 and 4, respectively, with 14.9%, 16.9 and 16.7% of patients in the respective arms experiencing these events in the neoadjuvant period. The most common AEs of any cause in the neoadjuvant period were generally similar across the three arms and reflected the safety profile of the cytotoxic agents used in each arm. These AEs were nausea (39.2%), anemia (35.1%) and asthenia (32.4%) in Arm 1; nausea (35.2%), anemia (32.4%) and neutropenia (25.4%) in Arm 2; and anemia (44.4%), asthenia (42.6%), alopecia (31.5%) and thrombocytopenia (31.5%) in Arm 4 (Fig. 4a–c and Supplementary Table 1).

**Table 2:** Safety summary in neoadjuvant, postsurgery and adjuvant periods (safety population)

*n* (%)	Arm 1: durvalumab + oleclumab + CT				Arm 2: durvalumab + monalizumab + CT				Arm 4: durvalumab + Dato-DXd + Plt			
	Overall	Neoadjuvant	Postsurgery	Adjuvant	Overall	Neoadjuvant	Postsurgery	Adjuvant	Overall	Neoadjuvant	Postsurgery	Adjuvant
	*n* = 74	*n* = 74	*n* = 69	*n* = 57	*n* = 71	*n* = 71	*n* = 66	*n* = 53	*n* = 54	*n* = 54	*n* = 51	*n* = 47
Any TEAE	74 (100)	74 (100)	47 (68.1)	55 (96.5)	70 (98.6)	70 (98.6)	43 (65.2)	44 (83.0)	54 (100)	53 (98.1)	32 (62.7)	40 (85.1)
Any AE possibly related to any investigational treatment or chemotherapy	74 (100)	72 (97.3)	12 (17.4)	41 (71.9)	67 (94.4)	66 (93.0)	9 (13.6)	31 (58.5)	53 (98.1)	52 (96.3)	8 (15.7)	26 (55.3)
Grade ≥3 TEAE	38 (51.4)	27 (36.5)	17 (24.6)	5 (8.8)	45 (63.4)	30 (42.3)	15 (22.7)	11 (20.8)	22 (40.7)	13 (24.1)	7 (13.7)	8 (17.0)
Any grade ≥3 AE possibly related to any investigational treatment or chemotherapy	27 (36.5)	23 (31.1)	1 (1.4)	3 (5.3)	29 (40.8)	23 (32.4)	0	7 (13.2)	11 (20.4)	10 (18.5)	1 (2.0)	3 (6.4)
AE leading to discontinuation of any investigational treatment or chemotherapy	12 (16.2)	7 (9.5)	2 (2.9)	4 (7.0)	16 (22.5)	12 (16.9)	0	4 (7.5)	6 (11.1)	6 (11.1)	1 (2.0)	0
Any SAE	25 (33.8)	12 (16.2)	11 (15.9)	5 (8.8)	26 (36.6)	11 (15.5)	15 (22.7)	8 (15.1)	18 (33.3)	10 (18.5)	7 (13.7)	5 (10.6)
SAE with outcome of death	3 (4.1)	1 (1.4)^a^	2 (2.9)^b^	0	4 (5.6)	0	3 (4.5)^c^	1 (1.9)^d^	2 (3.7)	0	1 (2.0)^e^	1 (2.1)^f^

Data are shown for the safety population, which consisted of all patients who received at least one dose of any study intervention.

The overall period began with the first treatment dose and continued until the earliest of the following events: the last dose of the treatment (or surgery, whichever occurred later) plus 90 days, the date of DCO or the date of the first dose of subsequent anticancer therapy (excluding radiotherapy).

The neoadjuvant period extended from the date of first dose of neoadjuvant study treatment until the date of surgery for patients who underwent surgery. For patients who did not undergo surgery, this period lasted until the earliest of the following: the date of the last dose of neoadjuvant treatment plus 90 days, the date of the first dose of subsequent anticancer therapy (excluding radiotherapy) or the date of DCO.

The postsurgery period began on the day of surgery and extended to the earliest of the following dates: the day of the first dose of the study treatment postsurgery, the date of DCO, the date of the first dose of subsequent anticancer therapy (excluding radiotherapy) or 90 days after surgery.

The adjuvant period began on the date of the first dose of study treatment postsurgery and ended on the earliest of these dates: 90 days after the last study treatment postsurgery, the date of DCO or the date of the first dose of subsequent anticancer therapy (excluding radiotherapy).

Deaths were due to: ^a^intestinal ischemia and septic shock related to chemotherapy (carboplatin and paclitaxel); ^b^atrial fibrillation and pneumonia, both related to surgery (both patients had a lobectomy); ^c^ sepsis (related to pneumonectomy), septic shock (related to lobectomy) and renal failure (related to bilobectomy); ^d^colonic obstruction related to durvalumab and monalizumab; ^e^idiopathic pulmonary fibrosis that was unrelated to the study treatment, according to the principal investigator, although independent adjudication found that it was related to the study treatment; and ^f^infectious endocarditis that was not related to any drugs or the surgery.

DCO, data cutoff; SAE, serious adverse event; TEAE, treatment-emergent adverse event.

Overall, AEs of any cause leading to discontinuation of any investigational product or chemotherapy occurred in 16.2% of patients in Arm 1, 22.5% in Arm 2 and 11.1% in Arm 4; 9.5%, 16.9%, and 11.1% of patients in the respective arms experienced these AEs in the neoadjuvant period. Overall, AEs of any cause with an outcome of death occurred in 4.1% of patients in Arm 1, 5.6% in Arm 2 and 3.7% in Arm 4. AEs possibly related to the study treatment with an outcome of death occurred in one patient (1.4%) in Arm 1, which happened during the neoadjuvant period and was due to intestinal ischemia and septic shock, deemed possibly related to chemotherapy; one patient (1.4%) in Arm 2, which occurred during the adjuvant period and was due to colonic obstruction, deemed possibly related to durvalumab and monalizumab; and one patient (1.9%) in Arm 4, which occurred during the postsurgery period and was due to interstitial lung disease (ILD), which was deemed not related to study treatment by the treating physician but was deemed related to study treatment by independent adjudication. This patient had pre-existing pulmonary fibrosis noted on computed tomography scans during screening, which was asymptomatic and stable through the neoadjuvant period.

Immune-mediated AEs for durvalumab occurred in 6.8%, 12.7% and 9.3% of patients in the neoadjuvant period in Arms 1, 2 and 4, respectively. The most common AEs of special interest for Dato-DXd in Arm 4 in the neoadjuvant period were oral mucositis or stomatitis (40.7%), ocular surface events (20.4%) and mucosal inflammation (14.8%) (Fig. 4d); most events were low-grade. One patient (1.9%) in Arm 4 had grade 2 ILD in the neoadjuvant period. This patient remains on study on adjuvant durvalumab.

## Discussion

NeoCOAST-2 is, to our knowledge, the first global phase II study to report clinical data on an ADC in the neoadjuvant setting for patients with resectable NSCLC and the first to report data on neoadjuvant combination regimens (immune checkpoint inhibitor therapy plus chemotherapy plus novel agents) in this patient population. In patients with resectable NSCLC, neoadjuvant durvalumab plus platinum-based chemotherapy combined with oleclumab, monalizumab or Dato-DXd, followed by adjuvant durvalumab combined with oleclumab or monalizumab, or durvalumab alone, resulted in promising pathological response rates while maintaining a manageable safety profile consistent with that of current SoC perioperative regimens.

Findings from recent phase III trials (AEGEAN, CheckMate 816, KEYNOTE-671 and CheckMate 77T), among other studies, suggest that there is a strong positive association between pathological response and long-term benefit^6–9^. Although cross-trial comparisons are challenging owing to inherent differences in key trial design elements, Arms 1 and 2 exhibited similar overall pCR rates compared with current SoC regimens; Arm 4 yielded a numerically higher overall pCR rate (35.2%; 95% CI, 22.7–49.4) compared with Arms 1 and 2 as well as the SoC (pCR rates ranging from 17% to 25% in AEGEAN, CheckMate-816, KEYNOTE-671 and CheckMate 77T)^6–9^. Overall mPR rates in Arms 1, 2 and 4 were numerically higher than those seen with the SoC (mPR ranging from 30% to 37%)^6–9^, with Arm 4 exhibiting the highest mPR rate (63.0%; 95% CI, 48.7–75.7). Additional follow-up is needed to assess the long-term benefit in Arms 1, 2 and 4, to better understand whether the numerically higher mPR rates in Arms 1 and 2, and the numerically higher pCR and mPR rates in Arm 4, will translate to improvement in long-term outcomes, such as EFS and overall survival. The contribution of individual agents in the tested regimens cannot be directly assessed. However, using AEGEAN as a reference evaluating neoadjuvant durvalumab in combination with platinum chemotherapy (pCR rate: 17.2% (95% CI, 8.7–17.6); mPR rate: 33.3% (95% CI, 28.5–38.4)) to perform an exploratory comparison, our results suggest that adding novel agents such as ADCs in the neoadjuvant setting may augment the pathological response benefit without a meaningful increase in toxicity^6^.

Pathological responses were observed across PD-L1 subgroups and histologies, with higher response rates in patients with squamous tumor histology, which is generally consistent with observations from recent trials^6,8,9^. In Arms 1 and 2, the magnitude of pCR and mPR benefit was greater in patients with tumor cell PD-L1 expression ≥ 1% versus < 1%, with the greatest benefit seen in patients with PD-L1 ≥ 50%. These results were consistent with other studies of neoadjuvant or perioperative immune checkpoint inhibitor therapy plus chemotherapy^6,8,9^. In Arm 4, pCR and mPR rates were generally similar across PD-L1 subgroups (<1%, 1–49% and ≥50%). The largest relative pCR benefit was seen in patients with tumor cell PD-L1 expression < 1%; AEGEAN and CheckMate 816 reported pCR rates of 9% and 17% in the PD-L1 < 1% population^6,9^, compared with 31.3% in Arm 4 of the NeoCOAST-2 study. Preclinical evidence for Dato-DXd and other TOP1i ADCs suggests that they could potentiate the effect of immune checkpoint inhibitors by inducing immunogenic cell death^17–19^, providing a potential mechanism for the numerically improved pCR and mPR rates seen in Arm 4 across PD-L1 subgroups. Larger studies are needed to confirm this effect, given the small sample size of these PD-L1 subgroups.

Arms 1, 2 and 4 in the NeoCOAST-2 trial exhibited an overall safety profile that was comparable to those of currently approved regimens^6–9^. As expected, across all three arms, the neoadjuvant period had the highest incidence of maximum grade ≥ 3 AEs of any cause, followed by the postsurgery and adjuvant periods. Of note, the adjuvant safety data were more mature in Arms 1 and 2 than in Arm 4 owing to those arms starting enrollment earlier. At the time of the data cutoff, 74.5% of patients in Arm 4 who started adjuvant therapy were still receiving treatment, compared with 36.8% in Arm 1 and 34.0% in Arm 2. Referencing the durvalumab group in AEGEAN, Arms 1, 2 and 4 exhibited similar rates of all-cause serious AEs and AEs leading to death in the overall period, whereas Arm 4 showed a numerically lower incidence of all-cause AEs of grade ≥3 (ref. ^6^). These data highlight the relatively manageable safety of novel combination therapies, particularly with an ADC in this setting.

Surgery has been and remains a cornerstone of curative treatment for early-stage resectable NSCLC. Most patients (approximately 80%) undergoing neoadjuvant treatment proceed to surgery, with a substantial proportion having R0 resections in the AEGEAN (94.7%), CheckMate 816 (83.2%), KEYNOTE-671 (92.0%) and CheckMate 77T (89.3%) trials^6–9^. In NeoCOAST-2, there were similar trends in the feasibility of surgery (93.2% in Arm 1, 93.0% in Arm 2 and 94.4% in Arm 4) and R0 resection rates (91.3%, 92.4% and 88.0%, respectively). Most patients in all arms underwent surgery with no delays. In the minority of patients who did have delays, most were <4 weeks. In all arms, most patients underwent lobectomy, with bilobectomy and pneumonectomy being performed in a minority of patients. The low rate of pneumonectomy was expected given that patients with planned pneumonectomies at screening were not eligible to enroll.

Our study has some limitations, including the lack of a dedicated control arm and power for statistical comparisons between arms, which precludes definitive cross-arm comparisons. Small sample sizes and limited enrollment of patients from minority ethnic groups also limit the interpretation of data within the PD-L1 and other subgroups. Nevertheless, the innovative platform study design allowed for the rapid assessment of multiple novel neoadjuvant and perioperative treatment regimens in association with translational and biomarker analyses to identify the most promising regimens for meaningful clinical impact, along with the patients most likely to derive benefit.

NeoCOAST-2 builds on the findings from recent perioperative trials in the early-stage resectable NSCLC setting and shows that novel treatment combinations of durvalumab with oleclumab, monalizumab or Dato-DXd plus chemotherapy provide promising efficacy with a manageable safety profile and no adverse impacts on surgical feasibility compared with historical benchmarks, albeit with small sample sizes, heterogeneous patient populations and different treatment settings. In addition, NeoCOAST-2 highlights the promising role of ADCs and immune checkpoint inhibition in potentially improving outcomes over immune checkpoint inhibitors plus chemotherapy in the neoadjuvant setting for resectable NSCLC, warranting further investigation. Further evaluations of new bispecific monoclonal antibodies targeting both PD-1 and CTLA-4 (volrustomig) in combination with chemotherapy, and both PD-1 and TIGIT (rilvegostomig) in combination with chemotherapy with or without Dato-DXd are currently ongoing in NeoCOAST-2.

## Methods

### Study design

NeoCOAST-2 is a phase II, open-label, multiarm, multicenter, global, randomized platform study. For the results reported here, patients were randomized into one of three arms. Arm 1 consisted of neoadjuvant durvalumab (1,500 mg intravenously (i.v.) every 3 weeks (Q3W)), oleclumab (3,000 mg i.v. Q3W) and platinum-doublet chemotherapy for four cycles (Extended Data Table 1), followed by surgery and adjuvant durvalumab and oleclumab Q4W for up to 1 year. Arm 2 consisted of neoadjuvant durvalumab (1,500 mg i.v. Q3W), monalizumab (1,500 mg i.v. Q3W) and platinum-doublet chemotherapy for four cycles, followed by surgery and adjuvant durvalumab plus monalizumab Q4W for up to 1 year. Arm 4 consisted of neoadjuvant durvalumab (1,500 mg i.v. Q3W), Dato-DXd (6 mg kg^–1^ body weight i.v. Q3W) and single-agent platinum chemotherapy for four cycles, followed by surgery and adjuvant durvalumab Q4W for up to 1 year. Additional study arms are being evaluated and will be reported separately.

A randomization method featuring a dynamically changing allocation ratio for treatment assignments was used to account for fluctuations in the number of patients enrolled in the treatment arms over the course of the study, and to enable an increase in enrollment for one or more treatment arms at the discretion of the sponsor and/or upon recommendation by the safety review committee (SRC). In instances when only a single arm was enrolling, all patients were allocated to that arm. Any changes to the allocation ratio were promptly communicated to the investigators, and patients were informed by the investigator or delegate of the enrolling treatment arms at the time that informed consent was given. The actual treatment administered to patients was determined by the randomization scheme using interactive response technology (IRT). The randomization scheme was produced by software that incorporates a standard procedure for generating randomization numbers. A randomization list was produced for each randomization strata. A blocked randomization was generated, and randomization was balanced within the IRT at the central level.

Patients were stratified on the basis of their baseline PD-L1 expression status (<1% versus ≥1%). Surgery was scheduled to occur within 40 days following the last dose of neoadjuvant treatment. Patients were slated to start adjuvant treatment within 10 weeks after surgery (those receiving postoperative radiation therapy (PORT) started adjuvant treatment within 8 weeks after surgery and within 3 weeks from the end of PORT) and received additional cycles of adjuvant treatment for up to 1 year postsurgery. Treatment continued until radiological disease progression was confirmed by Response Evaluation Criteria in Solid Tumors (RECIST) 1.1, withdrawal of consent, death or study completion.

An early safety evaluation was conducted by the SRC, including study principal investigators (PI), sponsor team members and an independent SRC chair who was not involved as a PI or sub-PI in the NeoCOAST-2 study, to review all available data after the first ten patients in each enrolling treatment arm had received two cycles of neoadjuvant treatment. An additional review of safety data by the SRC occurred when the first ten patients in each enrolling treatment arm underwent surgery and completed 21 days of follow-up, to assess perioperative mortality and surgery delays.

The SRC met regularly at approximately 6-month intervals to review the safety and tolerability of the neoadjuvant and adjuvant treatment regimens, until all patients had the opportunity to undergo surgery and those who had surgery completed at least 6 months of adjuvant treatment.

For all safety summaries by period, the following data were included:

- Neoadjuvant period: from the date of the first dose of neoadjuvant study treatment until the date of surgery for patients who underwent surgery; for patients who did not undergo surgery, this period extended to the minimum of either the date of the last dose of neoadjuvant treatment plus 90 days, the date of the first dose of subsequent anticancer therapy (excluding radiotherapy) or the date of data cutoff
- Postsurgery period: from the day of surgery extending to the minimum of the day of first dose of study treatment postsurgery, the date of data cutoff, the date of first dose of subsequent anticancer therapy (excluding radiotherapy) or the date of surgery plus 90 days
- Adjuvant period: from the date of first dose of study treatment postsurgery until the minimum of the date of the last study treatment postsurgery plus 90 days, the date of data cutoff or the date of first dose of subsequent anticancer therapy (excluding radiotherapy)
- Overall period: from the date of the first dose of study treatment until the minimum of the last dose of study treatment (or surgery, whichever is later) plus 90 days, the date of data cutoff or the date of first dose of subsequent anticancer therapy (excluding radiotherapy)

Data on sex were collected through the case report form, as allowed by local regulatory guidelines, and are reported in the patient baseline characteristics table. Analyses reported in this paper were not controlled for sex. No further sex-based analyses were conducted because a substantive difference in pathological response and safety were not expected between male and female patients.

### Eligibility criteria

Eligible patients were aged ≥18 years with newly diagnosed, previously untreated, histologically and cytologically documented stage IIA–IIIB NSCLC (as described by the International Association for the Study of Lung Cancer^5^), who had an Eastern Cooperative Oncology Group performance status of 0–1 and no sensitizing *EGFR* mutations or *ALK* translocations. Patients with stage IIIB N2 disease were also eligible following a protocol amendment. Key exclusion criteria included N3 disease (cancer spread to lymph nodes on the opposite side of the chest from the tumor, or to lymph nodes above the collarbone), history of allogeneic organ transplantation, history of non-infectious interstitial lung disease or pneumonitis that required steroids and need for pneumonectomy, segmentectomies or wedge resections, as assessed by the surgeon at baseline, to obtain potentially curative resection of the primary tumor.

Full eligibility criteria are shown below:

Inclusion criteria:

1. Capable of giving signed informed consent, which includes compliance with the requirements and restrictions listed in the ICF and in this protocol.
2. Provision of signed and dated written ICF prior to any mandatory study specific procedures, sampling and analyses.
3. Provision of signed and dated written ICF prior to collection of samples for genetic analysis.
4. Patients must be ≥18 years at the time of screening.
5. Newly diagnosed and previously untreated patients with histologically or cytologically documented NSCLC. Patients should have resectable (stage IIA–IIIB) disease (according to Version 8 of IASLC Staging Manual in Thoracic Oncology 2016 (ref. ^5^)). Patients with N2 disease are eligible if they were a candidate for lobectomy, sleeve resection or bilobectomy at the time of screening. Patients with N3 disease are excluded.
  At screening, complete surgical resection of the primary NSCLC must be deemed achievable, as assessed by a multidisciplinary evaluation, which must include a thoracic surgeon who performs lung cancer surgery as a prominent part of their practice.
    ∘ T4 tumours will be eligible if they are defined as T4 on the basis of their size (more than 7 cm) or if separate lesions are present in different ipsilateral lobes; any other reason for T4 (such as adherent to any of the following structures: diaphragm, mediastinum, heart, great vessels, trachea, recurrent laryngeal nerve, oesophagus, vertebral body or carina) will be considered ineligible.
    ∘ Nodal status should be investigated with whole body fluorodeoxyglucose positron emission tomography (PET), plus contrast-enhanced computed tomography (CT). If the PET or CT scan is positive in the mediastinum, or if the scan is negative but there is *T* > 3 cm, central tumor or cN1, then it is recommended that nodal status be proven by biopsy through endobronchial ultrasound, mediastinoscopy or thoracoscopy.
6. World Health Organization or ECOG performance status of 0 or 1 at enrollment.
7. Adequate organ and marrow function, as defined below:
  Hemoglobin ≥9.0 g dl^–1^ (transfusion of red blood cells or plasma is not allowed within 1 week prior to screening assessment).
  Absolute neutrophil count ≥1.5 × 10^9^ L^–1^.
  Platelet count ≥100 × 10^9^ L^–1^.
  Serum bilirubin ≤1.5 × upper limit of normal (ULN). This will not apply to patients with confirmed Gilbert’s syndrome, who will be allowed in consultation with their physician.
  Alanine transaminase and AST ≤ 3.0 × ULN.
  Measured creatine clearance (CrCL) > 45 ml min^–1^ or calculated CrCL > 45 ml min^–1^ as determined by Cockcroft–Gault (using actual body weight) using https://www.kidney.org/professionals/KDOQI/gfr_calculatorCoc.
  Left ventricular ejection fraction ≥ 50% as assessed by echocardiogram or MUGA scan (this criterion only applies if Arms 3A, 3B or 3C are open for enrollment)
  Troponin I or *T* ≤ lower limit of normal (per institutional guidelines and/or not clinically meaningful per investigator judgement).
8. Must have a life expectancy of at least 12 weeks.
9. Body weight > 35 kg.
10. Male and/or female.
  Females of childbearing potential should agree to use an acceptable method of contraception from the time of screening throughout the total duration of the study and for the following period after receiving the last dose of study interventions:
  Durvalumab: 90 days
  Oleclumab or monalizumab: 180 days
  Dato-DXd: 210 days
  For the chemotherapy agents, follow the local prescribing information relating to contraception, the time limits for such precautions and any additional restrictions for the agents administered. For patients receiving more than one study intervention, the longest washout period must be followed after the last dose of study interventions to prevent pregnancy. Female patients must not donate, bank or retrieve for their own use, ova during this same time period.
  Non-sterilized male partners of a woman of childbearing potential must use a male condom and spermicide (condom alone in countries where spermicides are not approved) throughout this period.
  Male patients who intend to be sexually active with a female partner of childbearing potential must be surgically sterile or using an acceptable method of contraception from the time of screening throughout the total duration of the study and for the following period after the last dose of study interventions:
  Durvalumab: 90 days
  Oleclumab, monalizumab: 180 days
  Dato-DXd: 120 days
  For the chemotherapy agents, follow the local prescribing information relating to contraception, the time limits for such precautions, and any additional restrictions for the agents administered. For patients receiving more than one study intervention, the longest washout period must be followed after the last dose of study interventions to prevent pregnancy in a partner. Male patients must not donate or bank sperm during the same time period as for contraception use. Female partners (of childbearing potential) of male patients must also use a highly effective method of contraception throughout this period.
11. Negative pregnancy test (serum) for women of childbearing potential
12. Provision of tumor samples (newly acquired or archival tumor tissue (≤6 months old) to confirm PD-L1 status, *EGFR* or *ALK* status where required during screening and prior to randomization.
  1. PD-L1 status:
    (i) Documented PD-L1 expression status from analytically validated, local-regulatory approved test or,
    (ii) Provision of tumor for local testing on analytically approved, local regulatory approved assay; SP263 antibody is preferred if available.
    Note: Local test results must differentiate between PD-L1 < 1%, 1% to 49% and ≥ 50%.
  2. *ALK* and *EGFR* status:
    (i) Previous local laboratory results for *ALK* and *EGFR* can be used if performed on well-validated, local regulatory-approved assay, or
    (ii) Provision of tumor for local testing on analytically approved, local regulatory approved assay
    (iii) Patients with an *EGFR* mutation, *ALK* rearrangement or unknown *ELK* or *ALK* status will not be randomized, with the following exceptions:
      ∘ Patients with squamous cell carcinoma do not require *ALK* status
13. Provision of tumor appropriate for exploratory biomarker analyses. Newly acquired or archival tumor tissue (≤6 months old) must be available from core needle biopsy, punch biopsy, excisional biopsy or surgical specimen. Fine needle aspirate is not acceptable. Core needle biopsies obtained by endobronchial ultrasonography are acceptable. Tissue cores must contain cells and stroma. Cytology samples and specimens with limited tumor content are considered inadequate and will not be acceptable.
14. Patients will be suitable for inclusion if the planned surgery to be performed will be lobectomy, sleeve resection or bilobectomy, as determined by the attending surgeon on the basis of the baseline findings.
15. A pre- or post-bronchodilator forced expiratory volume in 1 (FEV_1_) second of 1.0 L and diffusing capacity of the lung for carbon monoxide (DLCO) > 40% of the postoperative predicted value. Use of these cut-off values to assess candidacy for resection should be guided by the results of cardiopulmonary exercise testing as outlined in the ESMO guidelines on pretreatment risk assessment. Both an FEV_1_ and a DLCO test are required for assessing lung function at screening.

Exclusion criteria:

1. Sensitizing *EGFR* mutations or *ALK* translocations.
2. History of allogeneic organ transplantation.
3. Active or prior documented autoimmune or inflammatory disorders (including inflammatory bowel disease such as colitis or Crohn’s disease, diverticulitis (with the exception of diverticulosis), systemic lupus erythematosus, sarcoidosis, granulomatosis with polyangiitis, Graves’ disease, rheumatoid arthritis, hypophysitis, uveitis, autoimmune pneumonitis or autoimmune myocarditis). The following are exceptions to this criterion:
  Patients with vitiligo or alopecia.
  Patients with hypothyroidism (such as following Hashimoto syndrome) stable on hormone replacement.
  Any chronic skin condition that does not require systemic therapy.
  Patients without active disease in the last 5 years may be included, but only after consultation with the study physician or medical scientist.
  Patients with celiac disease controlled by diet alone.
4. Uncontrolled intercurrent illness, including but not limited to, uncontrolled hypertension, unstable angina pectoris, uncontrolled cardiac arrhythmia, active bleeding diseases, serious chronic gastrointestinal conditions associated with diarrhoea, or psychiatric illness or social situations that would limit compliance with study requirements, substantially increase the risk of incurring AEs or compromise the ability of the patient to give written informed consent.
5. History of another primary malignancy, except for the following:
  Malignancy treated with curative intent and with no known active disease ≥3 years before the first dose of study interventions and of low potential risk for recurrence.
  Adequately treated non-melanoma skin cancer or lentigo maligna without evidence of disease.
  Adequately treated carcinoma in situ without evidence of disease.
6. Patients with small-cell lung cancer or mixed small-cell lung cancer.
7. History of active primary immunodeficiency.
8. History of non-infectious ILD or pneumonitis that required steroids, has current ILD or pneumonitis or has suspected ILD or pneumonitis that cannot be ruled out by imaging at screening.
9. Evidence of the following infections:
  - Active infection including tuberculosis (clinical evaluation that includes clinical history, physical examination and radiographic findings and tuberculosis testing in line with local practice).
  - Known HIV infection that is not well controlled. All of the following criteria are required to define an HIV infection that is well controlled: undetectable viral RNA, CD4^+^ count ≥ 350, no history of acquired immune deficiency syndrome-defining opportunistic infection within the past 12 months and stable for at least 4 weeks on the same anti-HIV medications (meaning there are no expected further changes in that time to the number or type of antiretroviral drugs in the regimen). If an HIV infection meets the above criteria, monitoring of viral RNA load and CD4^+^ count is recommended. If Arms 4, 6 or 7 are open for enrollment, all participants must be tested for HIV during the screening period if acceptable by local regulations or an IRB/EC.
  - Active or uncontrolled HBV or HCV. Patients are eligible if they:
    ∘ Have controlled hepatitis C viral load defined as undetectable hepatitis C RNA by PCR either spontaneously or in response to a successful prior course of antihepatitis C therapy.
    ∘ Have received HBV vaccination with only anti-HBs positivity and no clinical signs of hepatitis.
    ∘ Are Hepatitis B surface antigen (HBsAg)-negative and hepatitis B core antibody-positive (that is, those who have cleared HBV after infection) and meet conditions i–iii bdelow:
    ∘ Are HBsAg^+^ with chronic HBV infection (lasting 6 months or longer) and meet conditions i–iii below:
      (i) Hepatitis B virus DNA viral load <100 IU ml^–1^.
      (ii) Have normal transaminase values.
      (iii) Start or maintain antiviral treatment if clinically indicated as per the investigator.
  - Patients with active hepatitis A.
10. Patients who have preoperative radiotherapy treatment as part of their care plan.
11. Patients who require or may require pneumonectomy, segmentectomies or wedge resections, as assessed by their surgeon at baseline, to obtain potentially curative resection of primary tumour.
12. QT interval corrected by Fridericia’s formula (QTcF) ≥ 470 ms (if prolonged, then two additional electrocardiograms should be obtained and the average QTcF interval should be used to determine eligibility).
13. Known allergy or hypersensitivity to any of the study interventions or any of the study intervention excipients or history of severe hypersensitivity reactions to other monoclonal antibodies.
14. Any medical contraindication to treatment with chemotherapy as listed in the local labelling.
15. Patients with moderate or severe cardiovascular disease:
  Presence of cardiac disease, including myocardial infarction or any other arterial thrombotic event, including cerebrovascular accident, transient ischemic attack or unstable angina pectoris, within 6 months prior to study enrollment.
  New York Heart Association class 3 or 4 congestive heart failure, or uncontrolled hypertension.
16. Patients with clinically meaningful corneal disease (this exclusion criterion only applies if arms 4 or 7 are open for enrollment).
17. Any concurrent chemotherapy, investigational product, biologic or hormonal therapy for cancer treatment. Concurrent use of hormonal therapy for non-cancer-related conditions (such as hormone replacement therapy) is acceptable.
18. Receipt of live attenuated vaccine within 30 days prior to the first dose of study interventions. Patients, if enrolled, should not receive live vaccine while receiving study interventions and up to 30 days after the last dose of study interventions.
19. Major surgical procedure (as defined by the investigator), including highly invasive dental procedures, within 30 days prior to the first dose of study interventions (the exclusion of highly invasive dental procedures only applies if Arm 5 is open for enrollment).
20. Prior exposure to approved or investigational immune-mediated therapy including, but not limited to, other anti-CTLA-4, anti-TIGIT, anti-PD-1, anti-PD-L1 and anti-PD-L2 antibodies. Patients who have received agents targeting the adenosine pathway (such as anti-CD73, anti-A2AR, anti-CD39), anti-NKG2A, anti-HLA-E agents and anti-LIF agents are also excluded. Patients who have received previous treatment with a TROP2 targeting ADC or with another ADC containing a chemotherapy agent that inhibits TOP1 activity are also excluded.
21. Current or prior use of immunosuppressive medication within 14 days before the first dose of study interventions. The following are exceptions to this criterion:
  Intranasal, inhaled, topical steroids or local steroid injections (such as intra articular injection).
  Systemic corticosteroids ≤10 mg day^–1^ of prednisone or its equivalent.
  Steroids as premedication for hypersensitivity reactions (such as CT scan premedication).
  Steroids as premedication for chemotherapy or for Dato-DXd
22. Participation in another clinical study with an investigational product administered within 30 days prior to enrollment
23. Previous study interventions (durvalumab, oleclumab, monalizumab, volrustomig, Dato-DXd, AZD0171 or rilvegostomig) assignment in the present study
24. Female patients who are pregnant or breastfeeding, or male or female patients of reproductive potential who are not willing to use effective birth control from the time of screening throughout the total duration of the study and for the following period after receiving the last dose of study interventions:
  Durvalumab: 90 days
  Oleclumab, monalizumab: 180 days
  Dato-DXd: 210 days (female patients) and 120 days (male patients)
  For the chemotherapy agents, the local prescribing information relating to contraception, the time limits for such precautions, and any additional restrictions for the agents administered must be followed. For patients receiving more than one study intervention, the longest washout period must be followed after the last dose of study interventions to prevent pregnancy.
25. Involvement in the planning and/or conduct of the study (applies to both AstraZeneca staff and/or staff at the study site).
26. Judgement by the investigator that the patient should not participate in the study if the patient is unlikely to comply with study procedures, restrictions and requirements.
27. Exclusion criteria for participation in the optional (DNA) genetics research component of the study include the following:
  Previous allogeneic bone marrow transplant.
  Non-leukocyte-depleted whole blood transfusion in 120 days of genetic sample collection.

### Study endpoints and assessments

The primary objective was to assess the pCR rate and the safety and tolerability of neoadjuvant and adjuvant treatment. Secondary objectives included the rate of mPR and feasibility of surgery.

Pathological responses were collected locally and subsequently compiled and reported by a central blinded independent pathology review. Primary tumors and sampled lymph nodes were assessed by central pathology review for the percentage of residual viable tumor that was identified on routine hematoxylin and eosin staining. pCR rate was defined as the proportion of patients with no viable tumor cells after complete evaluation of the resected specimen, including all sampled regional lymph nodes. Patients with resected lung cancer specimens containing ≤10% residual viable tumor cells in the primary tumor were considered to have a mPR. Central pathology assessments of pCR and mPR were performed according to the IASLC 2020 criteria^16^. Patients were considered to have no response if they were not eligible for assessment or if a surgical specimen was not available.

Feasibility of surgery was defined as having the planned surgical resection within 40 days after the last dose of neoadjuvant treatment. CT and PET were used for staging within 30 days prior to surgical evaluation. In cases of CT-enlarged or PET-positive lymph nodes, tissue confirmation was recommended. The European Society of Medical Oncology and National Comprehensive Cancer Network guidelines for the resectability of NSCLC were followed. The Clavien–Dindo assessment was used to grade postoperative complications.

Provision of tumor samples to confirm PD-L1, *EGFR* and *ALK* status was required prior to randomization. The baseline PD-L1 tumor proportion score was assessed centrally using the analytically validated VENTANA SP263 immunohistochemistry assay or locally using VENTANA SP263, pharmDx 22C3 or pharmDx 28-8 assays. Evaluable tumor samples from patients who were randomized on the basis of local tumor PD-L1 expression results were analyzed retrospectively using VENTANA SP263 at the central testing laboratory. The study protocol was later amended to allow testing with any certified assay with local regulatory approval to reduce the burden of testing and minimize delays in patient enrollment. This amendment did not impact patients enrolled in Arms 1, 2 and 4, and samples collected locally continue to be centrally analyzed in retrospect. At the 19 December 2024 data cutoff, tumor PD-L1 expression was based on central results in 68.4%, 73.6% and 63.0% of patients in Arms 1, 2 and 4, respectively, and on local results in 31.6%, 26.4% and 37.0% of patients, respectively, in the ITT population. In the mITT population, tumor PD-L1 expression was based on central results in 70.3%, 75.7% and 63.0% of patients, respectively, and on local results in 29.7%, 24.3% and 37.0% of patients, respectively.

Adverse events were coded using the latest version of the Medical Dictionary for Regulatory Activities and graded according to the National Cancer Institute’s Common Terminology Criteria for Adverse Events version 5.0.

An AESI was defined as an AE of scientific and medical interest specific to understanding the study interventions that requires close monitoring and is specific to a study drug. An AESI could be serious or non-serious, and these events were reported in the eCRF regardless of causality. An immune-mediated AE (imAE) was defined as an AESI that was associated with drug exposure, was consistent with an immune-mediated mechanism of action and had no clear alternative etiology, and required the use of systemic corticosteroids or other immunosuppressants and/or endocrine therapy for specific endocrine events.

An independent ILD adjudication committee was responsible for reviewing all cases of potential ILD or pneumonitis in Arm 4 as part of the Dato-DXd program requirements. To ensure adequate and relevant independent evaluation, additional systematic data collection was conducted for all cases that were brought for adjudication. The additional data collection covered more in-depth relevant medical history (including, for example, smoking, radiation exposure, chronic obstructive pulmonary disease and other chronic lung conditions), diagnostic evaluation, treatment and outcome of the event. This data collection was triggered on the basis of a predefined list of preferred terms eligible for adjudication.

### Statistical analysis

We planned that up to 70 eligible patients per arm would undergo randomization in the ITT population. The sample size was not based on type I error and power considerations. Instead, it was based on the model-based drug development approach^20,21^. The primary intent of the study was to evaluate preliminary efficacy signals in terms of pCR rates. The study was not powered to make direct statistical comparisons between arms. Single-arm analyses were conducted without statistical tests. The efficacy endpoints were assessed using the mITT population, which included all randomized patients with confirmed NSCLC histology who received at least one dose of study treatment. The safety endpoints, including feasibility of surgery, were assessed using the safety population, which included all patients who received at least one dose of study treatment. pCR and mPR were presented as the number and percentage of patients including 95% Clopper–Pearson CIs. Feasibility of surgery was summarized by the number and percentage of patients having surgical resection for each arm, along with the number and percentage of patients with surgical delays or complications. Analyses of clinical data were conducted using SAS v9.4.

### Study oversight

The study was performed in accordance with consensus ethical principles derived from international guidelines including the Declaration of Helsinki and Council for International Organizations of Medical Sciences International Ethical Guidelines, applicable International Council for Harmonisation Good Clinical Practice Guidelines and all applicable laws and regulations. The study included a SRC that conducted safety reviews of all enrolled patients throughout the study. The study protocol, protocol amendments, informed consent form, investigator brochure and other relevant documents were reviewed and approved by the Institutional Review Boards and Ethics Committees at each participating center, as follows. Belgium, UZ Leuven. Canada: McGill University Health Centre; Centre hospitalier de l’Université de Montréal; Alberta Health Services Cross Cancer Institute. France: CHU de Bordeaux Hopital Saint André; Centre Hospitalier de Cornouaille; CHU Dupuytren; Hopital Foch; CHU Rouen Hopital; Hopital d’Instruction des Armées Sainte Anne; Centre Hospitalier D’Avignon Hopital Henri Duffaut Médecine Interne Onco-Hématolo. Hungary: Bacs-Kiskun Varmegyei Oktatakorhaz; Tudogyogyintezet Torokbalint; Fejér Vármegyei Szent György Egyetemi Oktató Kórház; Szent Borbala Korhaz. Ireland: University Hospital Galway; Mater Misercordiae Hospital. Italy: Regina Elena; Istituto Nazionale dei Tumori; Istituti Fisioterapici Ospitalieri (IFO); Istituto di Ricovero e Cura a Carattere Scientifico (IRCCS); Ospedale San Gerardo – ASST di Monza, IRCCS; Istituto Clinico Humanitas Rozzano, IRCCS; Istituto Oncologico Veneto, IRCCS; Ospedale S.Maria della Misericordia; AO di Perugia S.C. Oncologia Medica; Ospedale Policlinico San Martino, IRCCS; SO di Cisanello; AOU Pisana; AOU Careggi UO Radioterapia; Ospedale Niguarda; ASST Grande Ospedale Metropolitano Niguarda; ASST Spedali Civili di Brescia. Portugal: Hospital da Luz; Centro Hospitalar e Universitário Lisboa Central Hospital Santo António dos Capuchos; Instituto Português Oncologia Francisco Gentil do Porto; Instituto Português de Oncologia de Lisboa Francisco Gentil. South Korea: Asan Medical Center; Seoul National University Hospital Department of Internal; The Catholic University of Korea; St. Vincent’s Hospital Oncology; CHA Bundang Medical Center; CHA University; Inje University Haeundae Paik Hospital. Spain: Hospital Teresa Herrera (CHUAC); Hospital Universitario Puerta De Hierro De Majadahonda; H.U.M. de Terrassa; H.U. Sant Joan de Reus; Hospital Clínico San Carlos; H. Clínico de Valencia; Hospital Universitario Virgen De La Macarena; Hospital Regional Universitario de Malaga; Hospital Clinic De Barcelona; Fundacion Jimenez Diaz; Hospital General Universitario De Alicante. Taiwan: National Cheng Kung University Hospital; Taipei Medical University Shuang Ho Hospital Pulmonology. Turkey: Goztepe Prof. Dr. Suleyman Yalcin Sehir Hastanesi Tibbi Onkoloji. United States: MD Anderson Cancer Center; Cleveland Clinic Medical Oncology/Hematology; John Hopkins Medicine Hematology/oncology; US Oncology Virginia Cancer Specialists, P.C. (VCS); Cleveland Clinic Florida Martin Health.

### Reporting summary

Further information on research design is available in the Nature Portfolio Reporting Summary linked to this article.

## Online content

Any methods, additional references, Nature Portfolio reporting summaries, source data, extended data, supplementary information, acknowledgements, peer review information; details of author contributions and competing interests; and statements of data and code availability are available at 10.1038/s41591-025-03746-z.

## Supplementary information

Supplementary Information Redacted protocol, SAP, Supplementary Table 1

Reporting Summary

## Figures and Tables

**Extended Data Table 1:**
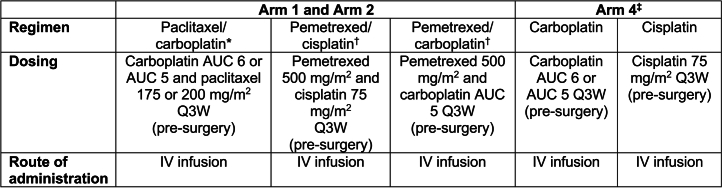
Chemotherapy regimen

*For squamous tumor histology

^†^For non-squamous tumor histology

^‡^Physician’s choice of carboplatin or cisplatin

AUC, area under the curve; IV, intravenous; Q3W, every 3 weeks.

**Extended Data Table 2:**
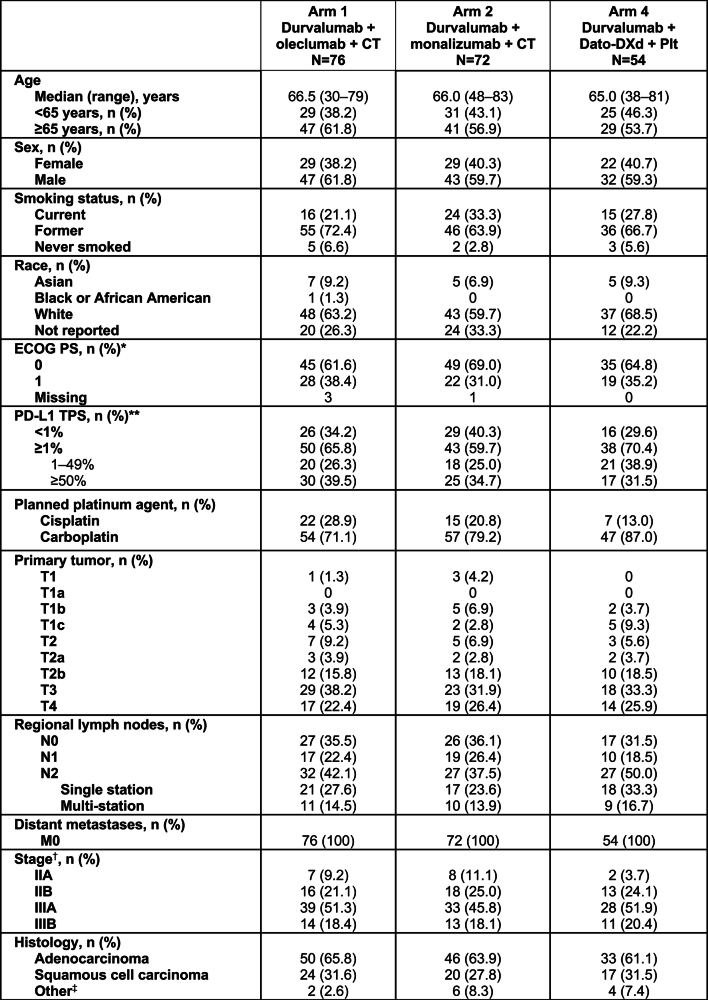
Patient demographics and baseline disease characteristics of the ITT population

Data cutoff: December 19, 2024. Based on the ITT population which consisted of all randomized patients. *Percentages calculated with the number of patients with reported performance status as denominator. **Central results in the ITT population were reported for 68.4%, 73.6% and 63.0% of patients in Arms 1, 2 and 4. ^†^Described by the International Association for the Study of Lung Cancer^5^. ^‡^Arm 1: carcinoma, type not determined (n = 2); Arm 2: carcinoma, type not determined (n = 1), other (n = 5); Arm 4: carcinoma, type not determined (n = 1), large cell carcinoma (n = 2), other (n = 1)

CT, platinum-doublet chemotherapy; Dato-DXd, datopotamab deruxtecan; ECOG PS, Eastern Cooperative Oncology Group performance status; ITT, intention-to-treat; PD-L1, programmed cell death ligand-1; Plt, single agent platinum chemotherapy; TNM, tumor node metastasis; TPS, tumor proportion score.

**Extended Data Table 3:**
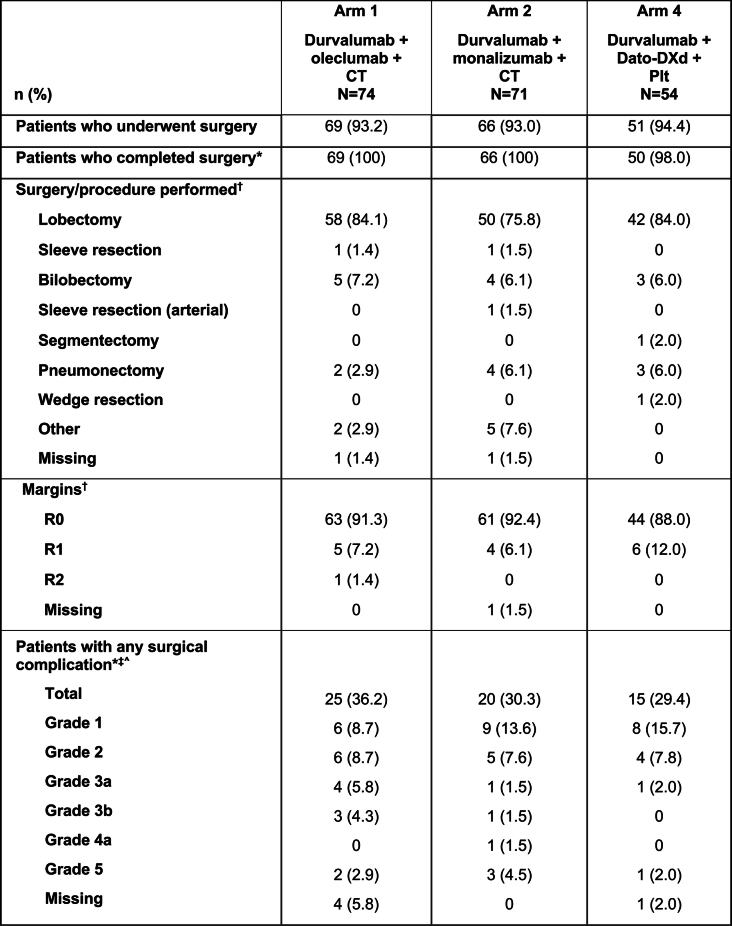
Surgical details (Safety population)

*Denominator is number of patients in the safety population who underwent surgery.

^†^Denominator is number of patients in the safety population who completed surgery.

^‡^Each patient is only represented at the maximum reported Clavien-Dindo classification grade.

CT, platinum-doublet chemotherapy; Plt, single agent platinum chemotherapy

^30-day mortality in Arms 1, 2 and 4 was 1.4%, 4.5%, and 2%, respectively. 90-day mortality in Arms 1, 2 and 4 was 2.9%, 4.5%, and 2%, respectively.

**Extended Data Table 4:**
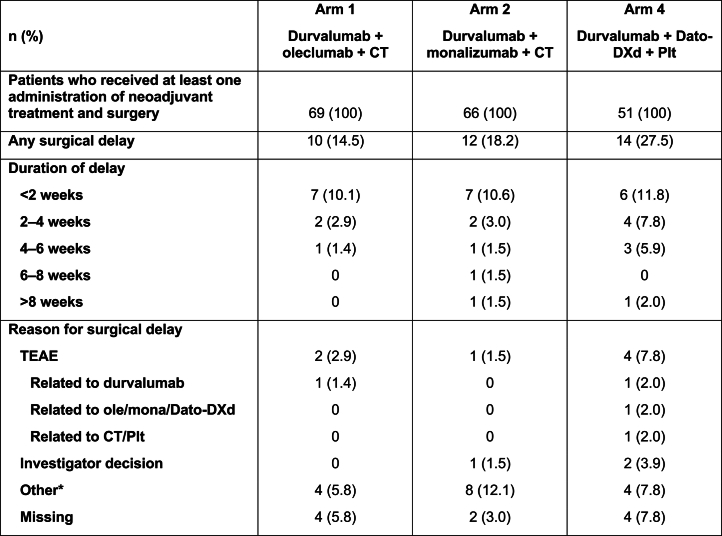
Surgical delay summaries

Data cutoff: December 19, 2024.

A surgical delay is defined as surgery occurring >40 days after the last dose of investigational product in the neoadjuvant period.

Denominator is number of patients who received at least one administration of neoadjuvant treatment and surgery.

*All surgical delays reported as Other were confirmed to be due to logistical, patient preference, or other scheduling issues.

CT, platinum-doublet chemotherapy; Dato-DXd, datopotamab deruxtecan; mona, monalizumab; ole, oleclumab; Plt, single agent platinum chemotherapy; TEAE, treatment-emergent adverse event.

**Extended Data Table 5:**
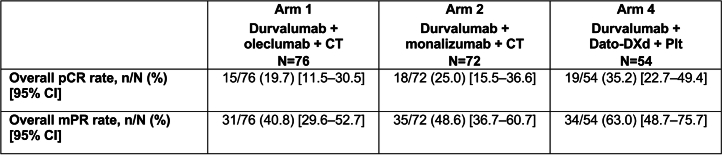
pCR and mPR rates for the ITT population

CI, confidence interval; CT, platinum-doublet chemotherapy; Dato-DXd, datopotamab deruxtecan; mPR, major pathological response; pCR, pathological complete response; Plt, single agent platinum chemotherapy.

**Extended Data Table 6:**
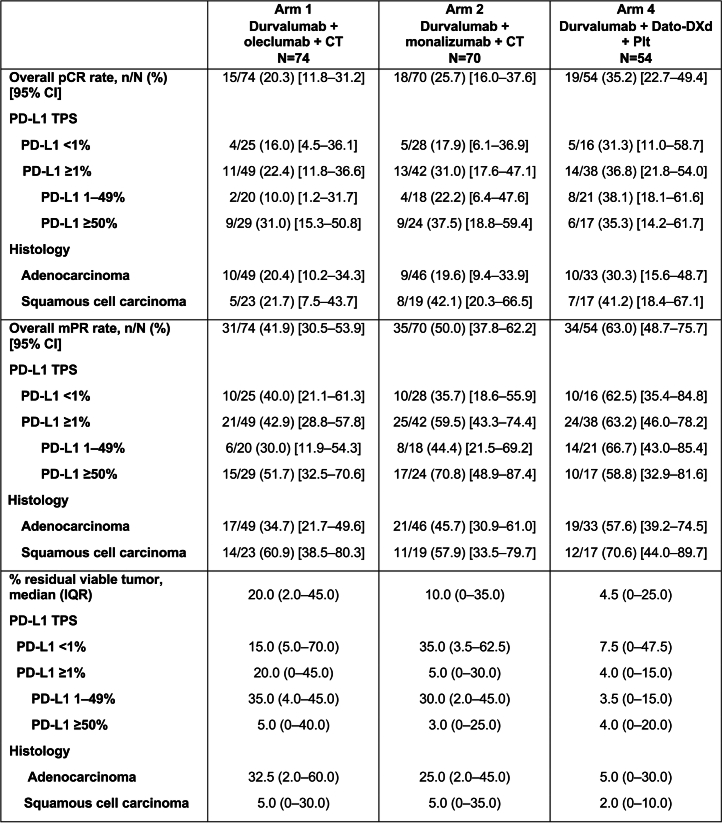
pCR and mPR rates and %RVT by PD-L1 TPS and histology in the mITT population

CI, confidence interval; CT, platinum-doublet chemotherapy; Dato-DXd, datopotamab deruxtecan; IQR, interquartile range; mPR, major pathological response; pCR, pathological complete response; PD-L1, programmed cell death ligand-1; Plt, single agent platinum chemotherapy; RVT, residual viable tumor; TPS, tumor proportion score.

**Extended Data Table 7:**
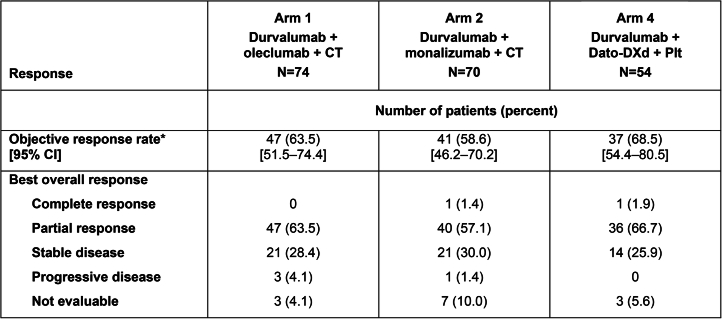
Objective response rate and best overall response by RECIST v1.1 in Arms 1, 2 and 4 in the mITT population

*Objective response rate was defined as a confirmed or unconfirmed complete or partial response from baseline to the pre-surgery scan per RECIST v1.1 based on investigator assessment. A RECIST 1.1 tumor assessment was performed after Cycle 2 and upon completion of the neoadjuvant period, prior to surgery. Confirmation was not required because there were only 2 scans prior to surgery.

CI, confidence interval; CT, platinum-doublet chemotherapy; mITT, modified intention-to-treat population; Plt, single agent platinum chemotherapy; RECIST, Response Evaluation Criteria in Solid Tumors.

## Data Availability

Data underlying the findings described in this manuscript may be obtained in accordance with AstraZeneca’s data sharing policy described at: https://astrazenecagrouptrials.pharmacm.com/ST/Submission/Disclosure. Data for studies directly listed on Vivli can be requested through Vivli at www.vivli.org. Data for studies not listed on Vivli could be requested through Vivli at https://vivli.org/members/enquiries-about-studies-not-listed-on-the-vivli-platform/. The AstraZeneca Vivli member page is also available outlining further details: https://vivli.org/ourmember/astrazeneca/.
