# Supplementary material for: Perioperative durvalumab plus chemotherapy plus new agents for resectable non-small-cell lung cancer: the platform phase 2 NeoCOAST-2 trial
Source: Nat Med. 2025 May 31;31(8):2788–96. doi: 10.1038/s41591-025-03746-z (PMC12353838; doi:10.1038/s41591-025-03746-z)
Supplement: Supplementary file 1 — Redacted protocol, SAP, Supplementary Table 1 [file 41591_2025_3746_MOESM1_ESM.pdf]

nature medicine

Supplementary information https://doi.org/10.1038/s41591-025-03746-z

Perioperative durvalumab plus chemotherapy plus new agents for resectable non-small-cell lung cancer: the platform phase 2 NeoCOAST-2 trial

In the format provided by the authors and unedited

Table of Contents

1. Supplementary Table 1

2. Redacted protocol

3. Redacted statistical analysis plan

Supplementary Table 1: Any-grade TEAEs in ≥5% of patients in the neoadjuvant, post-surgery and adjuvant periods in Arms 1,2, and 4

Arm 1: Durvalumab + oleclumab + CT					
Neoadjuvant N=74		Post-surgery N=69		Adjuvant N=57	
Nausea	29 (39.2)	Cough	9 (13.0)	Arthralgia	9 (15.8)
Anemia	26 (35.1)	Anemia	6 (8.7)	Hypothyroidism	8 (14.0)
Asthenia	24 (32.4)	Atrial fibrillation	6 (8.7)	COVID-19	7 (12.3)
Fatigue	21 (28.4)	Dyspnea	6 (8.7)	Constipation	7 (12.3)
Constipation	19 (25.7)	Pleural effusion	6 (8.7)	Cough	7 (12.3)
Diarrhea	16 (21.6)	Procedural pain	6 (8.7)	Pruritus	7 (12.3)
Hypomagnesemia	15 (20.3)	Asthenia	5 (7.2)	Pyrexia	6 (10.5)
Alopecia	13 (17.6)			Abdominal pain upper	5 (8.8)
Arthralgia	12 (16.2)			Asthenia	5 (8.8)
Decreased appetite	11 (14.9)			Bronchitis	5 (8.8)
Dysgeusia	10 (13.5)			Fatigue	5 (8.8)
Neutropenia	10 (13.5)			Lipase increased	5 (8.8)
Neutrophil count decreased	8 (10.8)			Nausea	5 (8.8)
Pyrexia	8 (10.8)			Pain in extremity	5 (8.8)
Headache	7 (9.5)			Rash	5 (8.8)
Pruritus	7 (9.5)			Back pain	4 (7.0)
Rash	7 (9.5)			Diarrhea	4 (7.0)
Blood creatinine increased	6 (8.1)			Headache	4 (7.0)
Cough	6 (8.1)			Abdominal pain	3 (5.3)
Myalgia	6 (8.1)			Amylase increased	3 (5.3)
Paresthesia	6 (8.1)			Anemia	3 (5.3)
Thrombocytopenia	6 (8.1)			Blood creatine phosphokinase increased	3 (5.3)
Vomiting	6 (8.1)			Decreased appetite	3 (5.3)
Dyspepsia	5 (6.8)			Paraesthesia	3 (5.3)
Pain in extremity	5 (6.8)			Pneumonia	3 (5.3)
Pneumonia	5 (6.8)			Vomiting	3 (5.3)
Abdominal pain upper	4 (5.4)				
Alanine aminotransferase increased	4 (5.4)				
Dysuria	4 (5.4)				
Mucosal inflammation	4 (5.4)				
Neuropathy peripheral	4 (5.4)				
Stomatitis	4 (5.4)				

Arm 2: Durvalumab + monalizumab + CT					
**Neoadjuvant N=71**		**Post-surgery N=66**		**Adjuvant N=53**	
Nausea	25 (35.2)	Procedural pain	7 (10.6)	Cough	7 (13.2)
Anemia	23 (32.4)	Pleural effusion	6 (9.1)	Hypothyroidism	7 (13.2)
Neutropenia	18 (25.4)	Non-cardiac chest pain	4 (6.1)	Arthralgia	6 (11.3)
Constipation	16 (22.5)	Pneumothorax	4 (6.1)	COVID-19	6 (11.3)
Fatigue	15 (21.1)			Dyspnea	5 (9.4)
Alopecia	14 (19.7)			Bronchitis	4 (7.5)
Asthenia	14 (19.7)			Asthenia	3 (5.7)
Diarrhea	11 (15.5)			Bronchospasm	3 (5.7)
Hypomagnesemia	10 (14.1)			Diarrhea	3 (5.7)
Thrombocytopenia	10 (14.1)			Hyperthyroidism	3 (5.7)
Decreased appetite	8 (11.3)			Hyperuricemia	3 (5.7)
Alanine aminotransferase increased	6 (8.5)			Lipase increased	3 (5.7)
Dysgeusia	6 (8.5)			Parasthesia	3 (5.7)
Dyspnea	6 (8.5)			Pneumonia	3 (5.7)
Pruritus	6 (8.5)			Pruritus	3 (5.7)
Vomiting	6 (8.5)			Rash	3 (5.7)
Mucosal inflammation	5 (7.0)				
Neuropathy peripheral	5 (7.0)				
Neutrophil count decreased	5 (7.0)				
Parasthesia	5 (7.0)				
Rash	5 (7.0)				
Gastroesophageal reflux disease	4 (5.6)				
Hyperkalemia	4 (5.6)				
Hypothyroidism	4 (5.6)				
Non-cardiac chest pain	4 (5.6)				
Pulmonary embolism	4 (5.6)				
Stomatitis	4 (5.6)				

Arm 4: Durvalumab + Dato-DXd + Plt					
Neoadjuvant N=54		Post-surgery N=51		Adjuvant N=47	
Anemia	24 (44.4)	Dyspnea	6 (11.8)	Pruritus	8 (17.0)
Asthenia	23 (42.6)	Cough	5 (9.8)	Hypothyroidism	6 (12.8)
Alopecia	17 (31.5)	Anemia	4 (7.8)	Asthenia	5 (10.6)
Thrombocytopenia	17 (31.5)	Atrial fibrillation	3 (5.9)	Arthralgia	4 (8.5)
Neutropenia	15 (27.8)	Dysphonia	3 (5.9)	Dyspnea	4 (8.5)
Constipation	13 (24.1)	Pneumothorax	3 (5.9)	Lipase increased	4 (8.5)
Nausea	13 (24.1)	Pyrexia	3 (5.9)	Alanine aminotransferase increased	3 (6.4)
Diarrhea	12 (22.2)			Amylase increased	3 (6.4)
Stomatitis	12 (22.2)			Back pain	3 (6.4)
Vomiting	9 (16.7)			Diarrhea	3 (6.4)
Fatigue	8 (14.8)			Edema peripheral	3 (6.4)
Mucosal inflammation	8 (14.8)			Pneumonia	3 (6.4)
Rash	7 (13.0)				
Epistaxis	6 (11.1)				
Decreased appetite	5 (9.3)				
Lacrimation increased	5 (9.3)				
Leukopenia	5 (9.3)				
Oropharyngeal pain	5 (9.3)				
Abdominal pain upper	4 (7.4)				
Cough	4 (7.4)				
Dry eye	4 (7.4)				
Dysgeusia	4 (7.4)				
Hypomagnesemia	4 (7.4)				
Hypotension	4 (7.4)				
Myalgia	4 (7.4)				
Pruritus	4 (7.4)				
Pyrexia	4 (7.4)				
COVID-19	3 (5.6)				
Dyspepsia	3 (5.6)				
Mouth ulceration	3 (5.6)				
Neutrophil count decreased	3 (5.6)				
Renal failure	3 (5.6)				
Rhinorrhea	3 (5.6)				
Urinary tract infection	3 (5.6)				

COVID-19, coronavirus disease 2019; CT, platinum-doublet chemotherapy; Plt, single-agent platinum chemotherapy

Clinical Study Protocol	
Study Intervention	Durvalumab, Oleclumab, Monalizumab, Volrustomig (MEDI5752), Dato-DXd, AZD0171, Rilvegostomig
Study Code	D9077C00001
Version	8.0
Date	06 Sep 2024

A Phase II, Open-label, Multicentre, Randomised Study of Neoadjuvant and Adjuvant Treatment in Patients with Resectable, Early-stage (II to IIIB) Non-small Cell Lung Cancer (NeoCOAST-2)

Sponsor Name: AstraZeneca AB

Legal Registered Address: AstraZeneca AB, 151 85 Södertälje, Sweden

Regulatory Agency Identifier Numbers

Investigational New Drug (IND) Number: 157077

European Clinical Trials Database (EudraCT) Number: 2021-003369-37

EU CT Number: 2023-508852-21-00

This CSP has been subject to a peer review according to AstraZeneca Standard procedures. The CSP is publicly registered and the results are disclosed and/or published according to the AstraZeneca Global Policy on Bioethics and in compliance with prevailing laws and regulations.

Protocol Number: D9077C00001

Amendment Number: 7

Study Intervention: Durvalumab, Oleclumab, Monalizumab, Volrustomig (MEDI5752), datopotamab deruxtecan (Dato-DXd), AZD0171, Rilvegostomig

Study Phase: II

Short Title: Neoadjuvant and Adjuvant Treatment in Resectable NSCLC

Acronym: NeoCOAST-2

Medical Monitor Name and Contact Information will be provided separately

International co-ordinating investigator

Tina Cascone, MD
MD Anderson Cancer Center
Houston, TX 77030

PROTOCOL AMENDMENT SUMMARY OF CHANGES TABLE

DOCUMENT HISTORY	
**Document**	**Date**
Amendment 7 Version 8.0	06 Sep 2024
Amendment 6 Version 7.0	22 May 2024
Amendment 5 Version 6.0	06 Mar 2024
Amendment 4 Version 5.0	28 Nov 2023
Amendment 3 Version 4.0	16 December 2022
Amendment 2 Version 3.0	24 August 2022
Amendment 1 Version 2.0	19 August 2021
Version 1.0	30 June 2021

The Protocol Amendment Summary of Changes Table is provided below for the current amendment and in Appendix T for previous amendments.

Amendment 7 (06 Sep 2024)

Overall Rationale for Amendment

This protocol was amended to incorporate 2 new study arms (6 and 7) in addition to clarifications and updates on the CSP. The updates are summarised below, and details are provided in Appendix P and Appendix Q. Other minor editorial updates were made throughout. Administrative changes, such as formatting, updates to abbreviations, and punctuation corrections are not presented in this summary.

List of Substantial Modifications

Section Number and Name	Description of Change	Brief Rationale
Throughout the document	Addition of rilvegostomig + platinum doublet chemotherapy arm (Arm 6) and Dato-DXd + rilvegostomig + single agent platinum chemotherapy arm (Arm 7). Updates to reflect this have been made to all relevant sections of the CSP, including updates to the overall sample size of the study (from 490 to 630 patients) and updates to the planned statistical analyses of pCR for the new arms, stratified by PD-L1 status.	To evaluate the safety and efficacy of rilvegostomig in combination with platinum doublet chemotherapy, and rilvegostomig in combination with Dato-DXd and single agent platinum chemotherapy in early-stage resectable NSCLC.

Section Number and Name	Description of Change	Brief Rationale
Throughout the document	Clarification added that patients enrolled into Arms 1 to 5 will be stratified by PD-L1 expression status (< 1% versus ≥ 1%), while patients enrolled into Arms 6 and 7 will be restricted to baseline PD-L1 expression status ≥ 1% and stratified by expression (1% to 49% versus ≥ 50%).<br>Clarification added that when Arms 6 and 7 are open to enrolment, no other arms will be open to enrolment concurrently.	To clarify how patients will be stratified by PD-L1 expression status at enrolment in the different arms.
Synopsis	Addition of text to provide rationale for the use of rilvegostomig in combination with chemotherapy and Dato-DXd in the treatment of NSCLC.	To provide rationale for new Arms 6 and 7.
Section 1.3 Schedule of Activities, Section 8.2.8 Ophthalmological Assessments, Appendix N1 Schedule of Activities, Appendix N6.1 Treatment Regimen, Appendix Q1 Schedule of Activities	• Use of prophylactic anti-emetics prior to Dato-DXd dosing and on subsequent days updated from "recommended" to "highly recommended"<br>• "eye dryness or irritation" replaced with "ocular surface events (eg, dry eye, decreased or blurred vision, photophobia, keratitis, corneal ulcer)"<br>• Clarification added that ophthalmologic assessments may not be limited to the assessments specified in the CSP<br>• Clarification added that a suitable alternative to fluorescein staining of the cornea may be used in exceptional circumstances<br>• Added additional details of ophthalmologic assessments to Schedules of Activities for study arms administering Dato-DXd	Updated to align with the Dato-DXd Product Safety Requirements.
Section 2.4.1 Potential Benefits	Addition of efficacy data from trials of rilvegostomig as monotherapy and in combination with chemotherapy.	To note evidence for the efficacy of rilvegostomig.
Section 2.4.2.3 Potential Risks	Addition of safety data from trials of rilvegostomig as monotherapy and in combination with chemotherapy or Dato-DXd.	To note evidence for the safety profile of rilvegostomig.
Section 5.1 Inclusion Criteria; Section 5.2 Exclusion Criteria; Section 8.3.13.2 Paternal Exposure; Appendix A3 Informed Consent Process; Appendix G1 Female Patients; Appendix G2 Male Patients with a Female Partner of Childbearing Potential	Requirements for use of contraception, reporting of pregnancies, and restrictions on donation and banking of sperm and ova updated to include time periods specific to rilvegostomig.	To add information specific to rilvegostomig.

Section Number and Name	Description of Change	Brief Rationale
Section 5.1 Inclusion Criteria, Section 6.3.1 Patient Enrolment and Randomisation, Section 8.6.1 Baseline Tumour Samples for Biomarker Assessments	Requirements for local testing of tumour samples for PD-L1, EGFR and ALK expression status updated to require local testing only.	Central testing for PD-L1, EGFR and ALK decommissioned; local testing required.
Section 5.2 Exclusion Criteria	Exclusion criterion #1 updated to exclude patients with baseline PD-L1 expression status < 1% (to apply only when Arms 6 or 7 are open for enrolment).<br>Exclusion criteria #9, #16, and #20 updated with requirements related to Arms 6 and 7.	To update with requirements related to addition of Arms 6 and 7.
Section 5.3 Lifestyle Considerations	Added length of time that patients should not donate blood or blood components for after receipt of the final dose of study interventions in Arms 6 and 7.	To add information specific to Arms 6 and 7.
Section 6.1.1 Investigational Products	In Table 5, addition of a column for rilvegostomig.<br>In Table 5 and Table 6, addition of footnotes to specify sequence of treatment administration in Arms 6 and 7.	To provide information for rilvegostomig and to clarify sequence of treatment administration in Arms 6 and 7.
Section 6.1.1 Investigational Products	Chemotherapy was updated from NIMP to IMP designation.	Updated per EU Clinical Trials Regulation.
Section 8.2.1 Clinical Safety Laboratory Assessments, Appendix M1 Schedule of Activities	Requirements for assessment of [redacted] for patients receiving [redacted] updated to require testing at screening and every [redacted] cycles starting from neoadjuvant Cycle [redacted] Day [redacted] until discontinuation of treatment and EOT for lipase,amylase and glycated haemoglobin.	Updated to align with the latest volrustomig Project Specific Safety Requirements.
Sections 9.4.2.1 Primary Endpoint and 9.5 Interim Analyses	Addition of text to provide additional analyses for Arms 6 and 7.<br>Clarification added that decision on futility will also be made based upon the totality of the data.	To add information specific to Arms 6 and 7, and to add clarification on futility decision.
Appendix A1 Regulatory and Ethical Considerations	Clarification added that, in the EU, the Sponsor will be responsible to ensure that the study is conducted in compliance with the protocol and the European Regulation 536/2014.	To confirm compliance with EU Clinical Trials Regulation.

Section Number and Name	Description of Change	Brief Rationale
	Addition of text regarding reporting of SUSARs in the EU.	
Appendix N5.2 Dato-DXd	Justification for study dose of Dato-DXd updated with data from more recent Dato-DXd IB DCO date of 16 November 2023.	To update with more recent safety data for Dato-DXd and align with justification in Appendix Q5.2.
Appendix N6.1 Treatment Regimen	Observation period after infusion of Dato-DXd shortened from 1 hour to 30 minutes.	Updated to align with the Dato-DXd Product Safety Requirements.
Appendix N7 Adverse Events of Special Interest Associated with Dato-DXd, Appendix N7.1 Adverse Event Reporting Requirements for Dato-DXd	IRR removed as an AESI. Requirement to report Grade ≥ 3 IRR events beyond the safety follow-up period removed.<br>The AESI of "Ocular Surface Toxicity" was renamed "Ocular Surface Events".<br>List of ILD/pneumonitis evaluations updated to:<br>• High-resolution CT<br>• Pulmonologist consultation (Infectious Diseases consultation as clinically indicated)<br>• Bronchoscopy and bronchoalveolar lavage if clinically indicated and feasible<br>• Pulmonary function tests (including forced vital capacity and carbon monoxide diffusing capacity) and pulse oximetry (SpO2)<br>• Clinical laboratory tests (arterial blood gases if clinically indicated, blood culture, blood cell count, differential white blood cell count, C-reactive protein, COVID-19 test)<br>• One blood sample collection for PK analysis<br>Keratitis was upgraded from "potential risk" to "important identified risk" following adverse drug reaction determination.<br>Mucosal inflammation updated from "identified risk" to "potential risk" following adverse drug reaction determination.<br>Requirement to report all events of ILD/pneumonitis beyond the 90-day safety follow-up period was added.	Updated to align with the Dato-DXd Product Safety Requirements.

List of Non-substantial Modifications

Section Number and Name	Description of Change	Brief Rationale
Throughout the document	Premedication required prior to Dato-DXd administration was updated as follows: "Must include antihistamines and **antipyretics, preferably** acetaminophen".	To ensure patients with allergy to acetaminophen can receive premedication and are not excluded from the study and to align with updated Dato-DXd program safety language.
Section 3 Objectives and Endpoints; Section 8.6.1 Baseline Tumour Samples for Biomarker Assessments	Exploratory biomarker analyses updated to include SLFN11.	To allow for analysis of SLFN11 expression.
Section 6.5 Concomitant Therapy, Appendix H1 Prohibited and Permitted Concomitant Medications/Therapies	Removal of text related to administration of chloroquine and hydroxychloroquine as concomitant medications for COVID-19 infection during Dato-DXd administration.	No longer relevant as chloroquine and hydroxychloroquine are no longer used for treatment of COVID-19.
Section 6.6.1 Dose Delays and Section 8.3.15 Toxicity Management Guidelines	Clarification added that agent-specific TMGs should be followed in the event that an agent is held due to treatment-related toxicity.<br>Clarification added that the AstraZeneca **study physician or medical scientist** should be consulted if the Investigator feels that a patient is ready to restart treatment prior to treatment-related toxicity resolving to Grade 2 or less.	For clarification of guidance.
Appendix R Country Specific Amendment	Addition of Appendix R to provide country-specific information for Belgium.	To clarify that Arms 4 and 5 are not open for enrolment in Belgium.

TABLE OF CONTENTS

TITLE PAGE 1
PROTOCOL AMENDMENT SUMMARY OF CHANGES TABLE 3
TABLE OF CONTENTS 8
LIST OF FIGURES 11
LIST OF TABLES 11
1 PROTOCOL SUMMARY 16
1.1 Synopsis 16
1.2 Schema 25
1.3 Schedule of Activities 25
2 INTRODUCTION 34
2.1 Disease Background 34
2.2 Study Interventions Background 36
2.3 Study Rationale 36
2.4 Benefit/Risk Assessment 38
2.4.1 Potential Benefits 38
2.4.2 Potential Risks 41
2.4.2.1 Durvalumab 41
2.4.2.2 Combination Therapy with Durvalumab Plus Oleclumab, Monalizumab, Dato-DXd or AZD0171 41
2.4.2.3 Chemotherapy Plus Combination Therapy with Durvalumab Plus Oleclumab, Monalizumab, Dato-DXd, or AZD0171; or Chemotherapy Plus Volrustomig or Rilvegostomig 42
2.4.3 Overall Benefit: Risk Conclusion 47
3 OBJECTIVES AND ENDPOINTS 49
4 STUDY DESIGN 53
4.1 Overall Design 53
4.1.1 Study Conduct Mitigation During Study Disruptions Due to Cases of Civil Crisis, Natural Disaster, or Public Health Crisis 57
4.2 Scientific Rationale for Study Design 57
4.2.1 Rationale for Efficacy Endpoints 57
4.3 Justification for Dose 58
4.3.1 Rationale for Duration of Neoadjuvant and Adjuvant Treatment 59
4.4 End of Study Definition 59
5 STUDY POPULATION 60
5.1 Inclusion Criteria 61
5.2 Exclusion Criteria 64
5.3 Lifestyle Considerations 68

5.4 Screen Failures 69
6 STUDY INTERVENTION 69
6.1 Study Intervention(s) Administered 69
6.1.1 Investigational Products 69
6.1.1.1 Study Interventions 74
6.1.1.2 Chemotherapy 74
6.1.2 Treatment Regimens 74
6.1.2.1 Chemotherapy 75
6.1.3 Surgery 76
6.1.4 Post-operative radiotherapy standardised guidance 80
6.1.5 Duration of Treatment 80
6.1.6 Post final data cut-off 81
6.2 Preparation/Handling/Storage/Accountability of Interventions 82
6.3 Measures to Minimise Bias: Randomisation and Blinding 82
6.3.1 Patient Enrolment and Randomisation 82
6.3.2 Procedures for Handling Incorrectly Randomised Patients 84
6.3.3 Methods for Assigning Treatment Groups 84
6.4 Study Intervention Compliance 85
6.5 Concomitant Therapy 85
6.5.1 Rescue Medication 86
6.6 Dose Modification 87
6.6.1 Dose Delays 87
6.7 Continued Access to Intervention after the End of the Study 89
7 DISCONTINUATION OF STUDY INTERVENTION AND PATIENT DISCONTINUATION/WITHDRAWAL 89
7.1 Discontinuation of Study Interventions 89
7.1.1 Follow-up for Safety 91
7.1.2 Follow-up for Survival 91
7.2 Patient Withdrawal from the Study 91
7.3 Lost to Follow-up 92
8 STUDY ASSESSMENTS AND PROCEDURES 93
8.1 Efficacy Assessments 94
8.1.1 Central Imaging 95
8.1.2 Overall Survival 95
8.2 Safety Assessments 96
8.2.1 Clinical Safety Laboratory Assessments 96
8.2.2 Physical Examinations 99
8.2.3 Vital Signs 99
8.2.4 Electrocardiograms 100
8.2.5 Echocardiogram/MUGA 100
8.2.6 WHO/ECOG Performance Status 101
8.2.7 Pulmonary Function Assessment 101

8.2.8 Ophthalmological Assessments 101
8.2.9 Other Safety Assessments 102
8.3 Adverse Events and Serious Adverse Events 103
8.3.1 Time Period and Frequency for Collecting AE and SAE Information 103
8.3.2 Follow-up of AEs and SAEs 104
8.3.3 Causality Collection 105
8.3.4 Adverse Events Based on Signs and Symptoms 105
8.3.5 Adverse Events Based on Examinations and Tests 105
8.3.6 Hy's Law 106
8.3.7 Disease Progression 106
8.3.8 New Cancers 106
8.3.9 Deaths 107
8.3.10 Adverse Events of Special Interest 107
8.3.10.1 Adverse Events of Special Interest for Durvalumab 107
8.3.10.2 Adverse Events of Special Interest Associated with Investigational Products 109
8.3.11 Safety Data to be Collected Following the Final Data Cut-off of the Study 109
8.3.12 Reporting of Serious Adverse Events 109
8.3.13 Pregnancy 110
8.3.13.1 Maternal Exposure 110
8.3.13.2 Paternal Exposure 111
8.3.14 Medication Error, Drug Abuse, and Drug Misuse 112
8.3.14.1 Timelines 112
8.3.14.2 Medication Error 112
8.3.14.3 Drug abuse 112
8.3.14.4 Drug Misuse 113
8.3.15 Toxicity Management Guidelines 113
8.3.15.1 Specific Toxicity Management and Dose Modification Information – Durvalumab, Monalizumab, Oleclumab, AZD0171, Volrustomig, and Rilvegostomig 114
8.3.15.2 Specific Toxicity Management and Dose Modification Information – Dato-DXd 115
8.3.15.3 Specific Toxicity Management and Dose Modification Information – Chemotherapy 115
8.4 Overdose 116
8.5 Human Biological Samples 116
8.5.1 Pharmacokinetics 116
8.5.1.1 Determination of Drug Concentration 117
8.5.2 Immunogenicity Assessments 117
8.5.3 Storage and Destruction of Pharmacokinetic/ADA samples 117
8.5.4 Pharmacodynamics 117
8.6 Human Biological Sample Biomarkers 117
8.6.1 Baseline Tumour Samples for Biomarker Assessments 118
8.6.2 Surgical Tumour Samples for Assessments 120
8.6.3 Blood Sample Collections for Biomarker Analysis 120
8.6.4 Collection of Optional On-Treatment Tumour Tissue Samples or Biopsy at

Progression 122
8.6.5 Management of Biomarker Data 123
8.6.6 Storage, Re-use, and Destruction of Biomarker Samples 123
8.7 CCI [redacted] 123
8.7.1 CCI [redacted] 123
8.7.2 CCI [redacted] 123
8.8 Health Economics OR Medical Resource Utilisation and Health Economics 124
9 STATISTICAL CONSIDERATIONS 124
9.1 Statistical Hypotheses 124
9.2 Sample Size Determination 124
9.3 Populations for Analysis 125
9.4 Statistical Analyses 125
9.4.1 General Considerations 125
9.4.2 Efficacy 126
9.4.2.1 Primary Endpoint 126
9.4.2.2 Secondary Endpoints 127
9.4.3 Safety 129
9.4.4 Other Analyses 130
9.4.4.1 Pharmacokinetics 130
9.4.4.2 Biomarkers 130
9.4.4.3 CCI [redacted] 131
9.4.4.4 Immunogenicity Data 131
9.5 Interim Analyses 131
9.6 Data Monitoring Committee 132
9.7 ILD Adjudication Committee 132
10 SUPPORTING DOCUMENTATION AND OPERATIONAL CONSIDERATIONS 133
11 REFERENCES 337

LIST OF FIGURES

Figure 1 Study Design 25
Figure 2 Protocol Structure 56

LIST OF TABLES

Table 1 Schedule of Activities for the Screening Period: All Patients 27
Table 2 Schedule of Activities for Patients who have Completed/Discontinued Treatment: All Patients 31
Table 3 Objectives and Endpoints 49

Table 4 Treatment Regimens 53
Table 5 Investigational Products 70
Table 6 Chemotherapy 73
Table 7 Clinical Chemistry 96
Table 8 Haematology 98
Table 9 Coagulation 98
Table 10 Urinalysis 98
Table 11 WHO/ECOG Performance Status 101
Table 12 CCI [redacted] 124
Table 13 Populations for Analysis 125
Table 14 RECIST 1.1 Overall Visit Response 160
Table 15 Highly Effective Methods of Contraception (< 1% Failure Rate) 164
Table 16 Prohibited medications/therapies 165
Table 17 Classification of Surgical Complications 168
Table K18 Schedule of Activities for Neoadjuvant Treatment for Patients Randomised to Arm 1 173
Table K19 Schedule of Activities for Adjuvant Treatment for Patients Randomised to Arm 1 178
Table L20 Schedule of Activities for Neoadjuvant Treatment for Patients Randomised to Arm 2 189
Table L21 Schedule of Activities for Adjuvant Treatment for Patients Randomised to Arm 2 194
Table M22 Schedule of Activities for Neoadjuvant Treatment for Patients Randomised to Arm 3A, 3B, or 3C 205
CCI [redacted]
Table N26 Schedule of Activities for Neoadjuvant Treatment for Patients Randomised to Arm 4 227
Table N27 Schedule of Activities for Adjuvant Treatment for Patients Randomised to Arm 4 232
Table N28 Dose Reduction Levels of Dato-DXd 243

CCI [redacted]

Table Q35 Overall Summary of TEAEs and TRAEs (Safety Analysis Set) (TROPION-Lung04) 295
Table Q36 Most Frequently Reported TEAEs (Occurring in ≥ 3 Participants) and TRAEs (Safety Analysis Set) (TROPION-Lung04) 296
Table Q37 BOR, ORR, and DCR as Assessed by Investigator per RECIST 1.1 (Interim Response Evaluable Analysis Set) (TROPION-Lung04) 297

LIST OF APPENDICES

Appendix A Regulatory, Ethical, and Study Oversight Considerations 134
Appendix B Adverse Events: Definitions and Procedures for Recording, Evaluating, Follow-up, and Reporting 142
Appendix C Handling of Human Biological Samples 148
Appendix D CCI [redacted] 150
Appendix E Actions Required in Cases of Increases in Liver Biochemistry and Evaluation of Hy's Law 153
Appendix F Guidelines for Evaluation of Objective Tumour Response Using RECIST 1.1 Criteria (Response Evaluation Criteria in Solid Tumours) 159
Appendix G Contraception Requirements 162
Appendix H Concomitant Medications 165
Appendix I Clavien-Dindo Classification of Surgical Complications 168
Appendix J Changes related to mitigation of study disruptions due to cases of civil crisis, natural disaster, or public health crisis 169
Appendix K Arm 1: Oleclumab + Durvalumab + Platinum Doublet Chemotherapy (Neoadjuvant Treatment) Followed by Oleclumab + Durvalumab (Adjuvant Treatment) 172
Appendix L Arm 2: Monalizumab + Durvalumab + Platinum Doublet Therapy (Neoadjuvant Treatment) Followed by Monalizumab + Durvalumab (Adjuvant Treatment) 188
CCI [redacted]
Appendix N Arm 4: Dato-DXd + Durvalumab + Single Agent Platinum Chemotherapy (Neoadjuvant Treatment) Followed by Durvalumab Alone (Adjuvant Treatment) 226
CCI [redacted]
[redacted]
[redacted]
Appendix R Country Specific Amendment 307

Appendix S Abbreviations ... 308
Appendix T Protocol Amendment History ... 314

1 PROTOCOL SUMMARY

1.1 Synopsis

Protocol Title: A Phase II, Open-label, Multicentre, Randomised Study of Neoadjuvant and Adjuvant Treatment in Patients with Resectable, Early-stage (II to IIIB) Non-small Cell Lung Cancer (NeoCOAST-2)

Short Title: Neoadjuvant and Adjuvant Treatment in Resectable NSCLC

Rationale:

A number of studies have demonstrated the clinical benefit of neoadjuvant chemotherapy in early-stage non-small cell lung cancer (NSCLC). Blockade of the programmed death-1 (PD-1) pathway in the neoadjuvant setting has been shown to provide clinical benefit by enhancing the antitumour response, and eradicating micrometastases prior to surgery, thereby reducing disease recurrence. However, despite recent improvements in pathologic complete response (pCR) rate, the majority of patients still do not achieve a pCR and may benefit from additional combination therapy approaches to improve responses and patient outcomes.

The NeoCOAST study (NCT03794544) evaluated one cycle of durvalumab alone or in combination with oleclumab or monalizumab as neoadjuvant treatment in patients with resectable, early-stage NSCLC. Safety and efficacy data suggests treatment across all arms were well tolerated with adverse event (AE) profile as expected in this patient population without impacting the feasibility of the surgical resection. Furthermore, adding oleclumab or monalizumab enhanced durvalumab benefit compared to durvalumab alone with improvement in major pathological response (mPR) and pCR rates.

Volrustomig (MEDI5752) plus chemotherapy is being evaluated in an ongoing study (D7980C00001; NCT03530397), enrolling patients into first-line advanced NSCLC expansion cohorts. Emerging efficacy data with volrustomig [redacted] mg plus platinum doublet chemotherapy show an improved objective response rate (ORR) in a PD-L1 < 1% subgroup compared to the historical benchmark of pembrolizumab plus platinum doublet chemotherapy in KN189 (Gandhi et al, 2018). Additionally, preliminary data from the NeoCOAST-2 study (D9077C00001; NTC05061550) using volrustomig [redacted] mg in combination with chemotherapy, followed by volrustomig [redacted] mg monotherapy, showed encouraging efficacy results with a similar proportion of subjects receiving surgery compared to neoadjuvant anti-PD-(L)1+ platinum doublet chemotherapy (Heymach et al, 2023; Forde et al, 2021; Appendix M 5). These encouraging data suggest volrustomig plus chemotherapy has the potential to deliver superior antitumour activity compared to anti-PD-1 agents alone or in combination with chemotherapy.

The clinical benefit of datopotamab deruxtecan (Dato-DXd) is currently being evaluated in advanced/metastatic NSCLC in combination with chemotherapy and PD-L1/PD-1 inhibitors

(TROPION-Lung02; NCT04526691, TROPION-Lung04; NCT04612751, TROPION-Lung07; NCT05555732; TROPION-Lung08; NCT05215340). The addition of a targeted antibody-drug conjugate (ADC) to anti-PD-1/PD-L1 immunotherapy plus chemotherapy combination may result in a synergistic tumour killing effect and therefore further improve treatment efficacy in the neoadjuvant setting.

Rilvegostomig has been evaluated in advanced/metastatic NSCLC in monotherapy or in combination with novel therapies, or chemotherapy (ARTEMIDE-01; NCT04995523, TROPION-Lung04 [in combination with Dato-DXd]; NCT04612751, MAGELLAN; NCT03819465). In NSCLC, 3 randomized Phase II studies have demonstrated superior efficacy of combined inhibition of TIGIT and PD-1 or PD-L1 compared to PD-1/PD-L1 blockade in participants with tumours with PD-L1 expression TPS ≥ 50% (CITYSCAPE, ARC-7, and NCT04672369). The addition of rilvegostomig to a platinum doublet chemotherapy regimen may result in a synergistic tumour killing effect and therefore further improve treatment efficacy in the neoadjuvant setting, while the addition of rilvegostomig to Dato-DXd and single agent chemotherapy may potentially generate stronger immune responses and extend the benefit of immune checkpoint blockade.

An ongoing Phase II study is currently evaluating the safety, pharmacokinetics (PK), and clinical activity of AZD0171 in combination with durvalumab and chemotherapy in first-line metastatic pancreatic adenocarcinoma (mPDAC) (D8151C00001; NCT04999969). Given the complementary mechanism of action to activate the immune antitumour response, the combined effects of anti-leukaemia inhibitory factor (LIF) and immune checkpoint inhibition may further induce tumour regression, improve survival and result in higher pCR rates and prolonged overall survival (OS).

The NeoCOAST-2 study, utilising a neoadjuvant and adjuvant approach to assess the role of durvalumab in combination with oleclumab, monalizumab or AZD0171 and platinum doublet chemotherapy; or volrustomig or rilvegostomig in combination with platinum doublet chemotherapy; or Dato-DXd in combination with durvalumab or rilvegostomig and single agent platinum chemotherapy has several advantages: first, the combination may result in pathological response and/or downstaging prior to surgery; second, early treatment of micrometastatic disease and avoidance of treatment delay due to post-operative complications; and finally, the resected tumours can be analysed, which provides a unique opportunity to perform correlative biomarker studies and investigate changes in the tumour microenvironment.

Objectives and Endpoints

Objectives	Estimand description/Endpoints
**Primary**	
To assess the antitumour activity of neoadjuvant treatment administered prior to surgery in terms of pCR	pCR is defined as lack of any viable tumour cells after complete evaluation in the resected lung cancer specimen and all sampled regional lymph nodes as determined by central BIPR and described by IASLC 2020 (Travis et al, 2020).<br>The measure of interest is the proportion of patients with 0% residual viable tumour cells within all resected tissue as assessed by the central blinded pathologist.
To assess the safety and tolerability of neoadjuvant and adjuvant treatment.	Safety and tolerability will be evaluated in terms of AEs, vital signs, and clinical laboratory parameters.
**Secondary**	
To assess the efficacy of neoadjuvant treatment administered prior to surgery followed by adjuvant treatment post-surgery in terms of EFS.	EFS is defined as time from randomisation to the first of the following:<br>• Documented local or distant recurrence as determined by Investigator using RECIST 1.1 assessment.<br>• Death due to any cause (event date is date of death).<br>• PD that precludes surgery (event date is the date of this determination) or PD discovered and reported by the Investigator upon attempting surgery that prevents completion of surgery (event date is the date of the first attempt at surgery).<br>A new primary malignancy confirmed by pathology is not considered an EFS event.<br>The measure of interest is the median of EFS and landmark EFS at 12 and 24 months.
To assess the efficacy of neoadjuvant treatment administered prior to surgery followed by adjuvant treatment post-surgery in terms of DFS (event from surgery onwards)	DFS is defined as the time from the date of surgery until the first date of disease recurrence as determined by Investigator using RECIST 1.1 assessment (local or distant), or date of death due to any cause, whichever occurs first. Pathological confirmation from biopsied lesions will also be taken into consideration (as applicable). A new primary malignancy confirmed by pathology is not considered a DFS event.<br>The measure of interest is the median of DFS and landmark DFS at 12 and 24 months.
To assess the feasibility of receiving the planned surgical tumour resection in patients receiving neoadjuvant treatment.	Feasibility to surgery is defined as having the planned surgical resection within 40 days from the end of the last dose of neoadjuvant study interventions.<br>The measure of interest is the proportion of patients that have intended surgery within 40 days from the end of last dose of neoadjuvant study interventions.

Objectives	Estimand description/Endpoints
To assess the antitumour activity of neoadjuvant treatment administered prior to surgery in terms of mPR	mPR is defined as ≤ 10% viable tumour cells in resected tumour after complete evaluation in the resected lung cancer specimen as determined by central BIPR as described by IASLC 2020 (Travis et al, 2020).<br>The measure of interest is the proportion of patients with ≤ 10% residual viable tumour cells within all resected tissue as assessed by the central blinded pathologist.
To assess the efficacy of neoadjuvant treatment administered prior to surgery in terms of ORR	ORR is defined as the proportion of patients who have a CR or PR as determined by Investigator using RECIST 1.1.<br>Data obtained from randomisation up until surgery, or the last evaluable assessment in the absence of progression, prior to surgery, will be included in the assessment of ORR, regardless of whether the patient withdraws therapy. Patients who go off therapy prior to surgery, without a response, receive a subsequent therapy prior to surgery, and then respond will not be included as responders in the ORR.<br>The measure of interest is the proportion of patients with OR.
To assess the efficacy of neoadjuvant and adjuvant treatment in terms of OS.	OS is defined as the time from randomisation until the date of death due to any cause.<br>The measure of interest is the landmark OS at 12 months and 24 months, and other clinically relevant timepoints if feasible. If reached by the end of the study, the median OS will also be of interest.
To describe the PK of study interventions in patients receiving neoadjuvant/adjuvant treatment	Concentration of study interventions in plasma or serum.
To assess the immunogenicity of study interventions in patients receiving neoadjuvant/adjuvant treatment.	Presence of ADA for study interventions.
To investigate baseline PD-L1 expression in patients treated with neoadjuvant and adjuvant treatment, and associations with clinical endpoints.	Baseline PD-L1 expression.
To evaluate changes in ctDNA during neoadjuvant treatment in patients with evaluable ctDNA and associations with clinical endpoints.	ctDNA clearance on-treatment prior to surgery.

Abbreviations: ADA: anti-drug antibody; Aes: adverse events; BIPR: blinded independent pathologist review; CR: complete response; ctDNA: circulating tumour DNA; DFS: disease-free survival; EFS: event-free survival; IASLC: International Association for the Study of Lung Cancer; mPR: major pathological response; OS: overall survival; ORR: objective response rate; pCR: pathological complete response; PD: progression of disease; PD-L1: programmed cell death ligand-1; PK: pharmacokinetics; PR: partial response; RECIST: Response Evaluation Criteria in Solid Tumours.

For Tertiary/exploratory objectives and endpoints, see Section 3 of the protocol.

Overall Design

Study D9077C00001 (NeoCOAST-2) is a Phase II, open-label, multi-arm, multicentre, randomised, neoadjuvant/adjuvant study for the treatment of patients with resectable early-stage NSCLC (Stage II to IIIB, according to Version 8 of the IASLC Staging Manual in Thoracic Oncology 2016).

Up to 630 patients will be enrolled and randomised to one of the following treatment regimens:

	Neoadjuvant treatment/Pre-Surgery (4 cycles)	Adjuvant treatment/Post-Surgery (1 year)
**Arm 1** (n=up to 70)	Oleclumab + durvalumab + CTX (Q3W) x 4 cycles	Oleclumab + durvalumab (Q4W) x 12 cycles
**Arm 2** (n=up to 70)	Monalizumab + durvalumab + CTX (Q3W) x 4 cycles	Monalizumab + durvalumab (Q4W) x 12 cycles
**Arm 3A** [a] (n=up to 70)	Volrustomig [redacted] mg + CTX [redacted] cycles)	Volrustomig [redacted] mg [redacted] x [redacted] cycles
**Arm 3B** (n=up to 70)	Volrustomig + CTX [redacted] (Volrustomig [redacted] mg + CTX x [redacted] cycle; Volrustomig [redacted] mg + CTX x [redacted] cycles)	Volrustomig [redacted] mg [redacted] x [redacted] cycles
**Arm 3C** (n=up to 70)	Volrustomig + CTX [redacted]	Volrustomig [redacted] mg [redacted] x [redacted] cycles
**Arm 4** (n=up to 70)	Dato-DXd + durvalumab + single agent platinum chemotherapy (Q3W) x 4 cycles	Durvalumab (Q4W) x 12 cycles
**Arm 5** (n=up to 70)	AZD0171 + durvalumab + CTX [redacted] x [redacted] cycles	AZD0171 [redacted] + Durvalumab [redacted] x [redacted] cycles
**Arm 6** (n=up to 70)	Rilvegostomig + CTX [redacted] x [redacted] cycles	Rilvegostomig [redacted] x [redacted] cycles
**Arm 7** (n=up to 70)	Dato-DXd + Rilvegostomig + single agent platinum chemotherapy [redacted] x [redacted] cycles	Rilvegostomig [redacted] x [redacted] cycles

Note: Treatment regimens may be given unless there is unacceptable toxicity, withdrawal of consent, or another discontinuation criterion is met (see Figure 1).

Note: Refer to Section 6.1.1 for details on study intervention and chemotherapy regimens.
Abbreviations: CTX: platinum doublet chemotherapy; Q2W: every 2 weeks; Q3W: every 3 weeks; Q4W: every 4 weeks.
[a] Recruitment closed at [redacted] patients, no additional patients will be enrolled in Arm 3A.

Patients enrolled into Arms 1 to 5 will be stratified by baseline PD-L1 expression status (< 1% versus ≥ 1%). Patients enrolled in Arms 6 and 7 will be restricted to baseline PD-L1 expression status ≥ 1% and stratified by expression (1% to 49% versus ≥ 50%).

In the neoadjuvant period, surgery is to be performed within 40 days from last dose of study interventions. Every attempt should be made to ensure surgery is performed within 40 days from the last dose of neoadjuvant treatment. Cases where surgery cannot be completed within 40 days from the last dose of study interventions should be promptly reported to the Sponsor and discussed with the Study Physician/Medical Scientist. Surgery may consist of lobectomy, sleeve resection, or bilobectomy, as determined by the attending surgeon based on the baseline findings. Patients whose planned surgery at enrolment includes pneumonectomy, segmentectomies, or wedge resections are not eligible for this study. After surgery, patients should be able to start adjuvant treatment as soon as clinically feasible and within 10 weeks from surgery (except for patients receiving post-operative radiation therapy (PORT), which must be started within 8 weeks after surgery. Adjuvant treatment must be started within 3 weeks from the end of PORT).

An early safety evaluation will be done by a safety review committee (SRC) to review all available data when the first [redacted] patients in each enrolling treatment arm have received [redacted] cycles of neoadjuvant treatment. An additional review of safety data by the SRC will occur when [redacted] patients in each enrolling treatment arm have undergone surgery and have had 21 days of follow-up, in order to assess perioperative mortality and surgery delays.

Additionally, throughout the study if more than [redacted]% of patients experience a delay in surgery of more than [redacted] weeks or become unable to receive surgery due to toxicity on any treatment arm, enrolment in that arm will be halted and SRC members will be notified to review these cases and provide their recommendation to the Sponsor.

The SRC will meet regularly at approximately [redacted]-month intervals to review the safety and tolerability of treatment as neoadjuvant and adjuvant regimen, until all patients have had the opportunity to undergo surgery and those having surgery have had at least [redacted] months of adjuvant treatment.

Reasons for delay to surgery which will not trigger enrolment hold are:

- Weather or other local emergencies impacting normal hospital activities.
- Unforeseen circumstances not related to the disease under study, such as, but not limited to, car accident, surgeon schedule, family illness or unexpected travel.
- Withdrawal of consent to further participation on the study not related to AEs or study procedures.

Other treatment arms may be added based on emerging nonclinical and clinical data via a protocol amendment.

Disclosure Statement: This is a Phase II, randomised, open-label, multi-arm, multicentre,

international study assessing the efficacy and safety of adjuvant and neoadjuvant treatment in patients with resectable, early-stage (II to IIIB) NSCLC.

Patient Population:

The target population of interest in this study is patients with resectable early-stage (II to IIIB) NSCLC.

Number of Patients:

Up to 630 patients will be enrolled.

Intervention Groups and Duration:

Patients will be randomised to the enrolling treatment arms and will receive one of the treatment regimens listed in Figure 1. A randomisation method with dynamically changing randomisation ratios will be employed to account for fluctuation in the number of treatment arms open for enrolment over the course of the study. Patients enrolled into Arms 1 to 5 will be stratified by baseline PD-L1 expression status (< 1% versus ≥ 1%). Patients enrolled in Arms 6 and 7 will be restricted to baseline PD-L1 expression status ≥ 1% and stratified by expression (1% to 49% versus ≥ 50%). When Arms 6 and 7 are open to enrolment, no other arms will be open to enrolment concurrently (Figure 1).

In the neoadjuvant treatment period (lasting for 4 cycles), treatment will be stopped at RECIST 1.1-defined radiological PD or clinical progression unless it is confirmed by the Investigator in agreement with the Study Physician/Medical Scientist that the patient continues to have a resectable tumour and continues to have clinical benefit. In the adjuvant treatment period (lasting for 1 year), treatment will be stopped at RECIST 1.1-defined radiological PD or clinical progression, unless it is confirmed by the Investigator in agreement with the Study Physician/Medical Scientist that the patient continues to have clinical benefit. Treatment will be stopped at RECIST 1.1-confirmed radiological PD.

Follow-up of patients post discontinuation of study interventions:

After study interventions completion / discontinuation, all patients will undergo an end-of-treatment visit (30 days [+/- 3 days] since last dose or study intervention, which includes surgery) and will be followed up for safety assessments 60 and 90 days after their last dose or of study interventions (i.e., the safety follow-up visits).

Patients who are discontinued from the study intervention will be followed for safety per Section 7.1.1, including the collection of any protocol-specified blood specimens, unless consent is withdrawn, or the patient is lost to follow-up or the patient is administered subsequent anticancer therapy.

In addition, all patients will be followed up for survival status as indicated in Schedule of

Activities (SoA) (Table 2) until death, withdrawal of consent, or the end of the study.

Data Monitoring Committee: No.

The study will have a SRC to conduct safety reviews. The membership, roles and responsibilities are defined in a charter (separate from the CSP).

Statistical methods

Statistical analyses will be performed by AstraZeneca or its representatives, including CROs.

The sample size of up to 70 patients per arm is to:

- Ensure the accuracy of making Go or No-Go decision at the interim analysis and final analysis.
- Obtain a preliminary assessment of antitumour activity with a certain degree of precision.

The sample size is not based on Type I error and power considerations. More details will be included in the Statistical Analysis Plan (SAP).

Continuous data will be summarised by the number of observations, mean, standard deviation (SD), median, minimum, and maximum. Geometric mean and coefficient of variation may be presented as applicable. Categorical variables will be summarised by frequency counts and percentages for each category. Unless otherwise stated, percentages will be calculated from the population total. Time-to-event variables will be presented using the Kaplan-Meier methodology, including median time and rates at landmark timepoints estimated from the Kaplan-Meier curves, where feasible.

In general, the last observed measurement prior to first dose of study interventions will be considered the baseline measurement. For assessment on the day of first dose where time is not captured, a nominal pre-dose indicator, if available, will serve as sufficient evidence that the assessment occurred prior to first dose. Assessments on the day of first dose, when neither time nor nominal pre-dose indicator are captured, will be considered prior to first dose if such procedures are required by the protocol to be conducted before the day of first dose.

1.2 Schema

Figure 1 Study Design

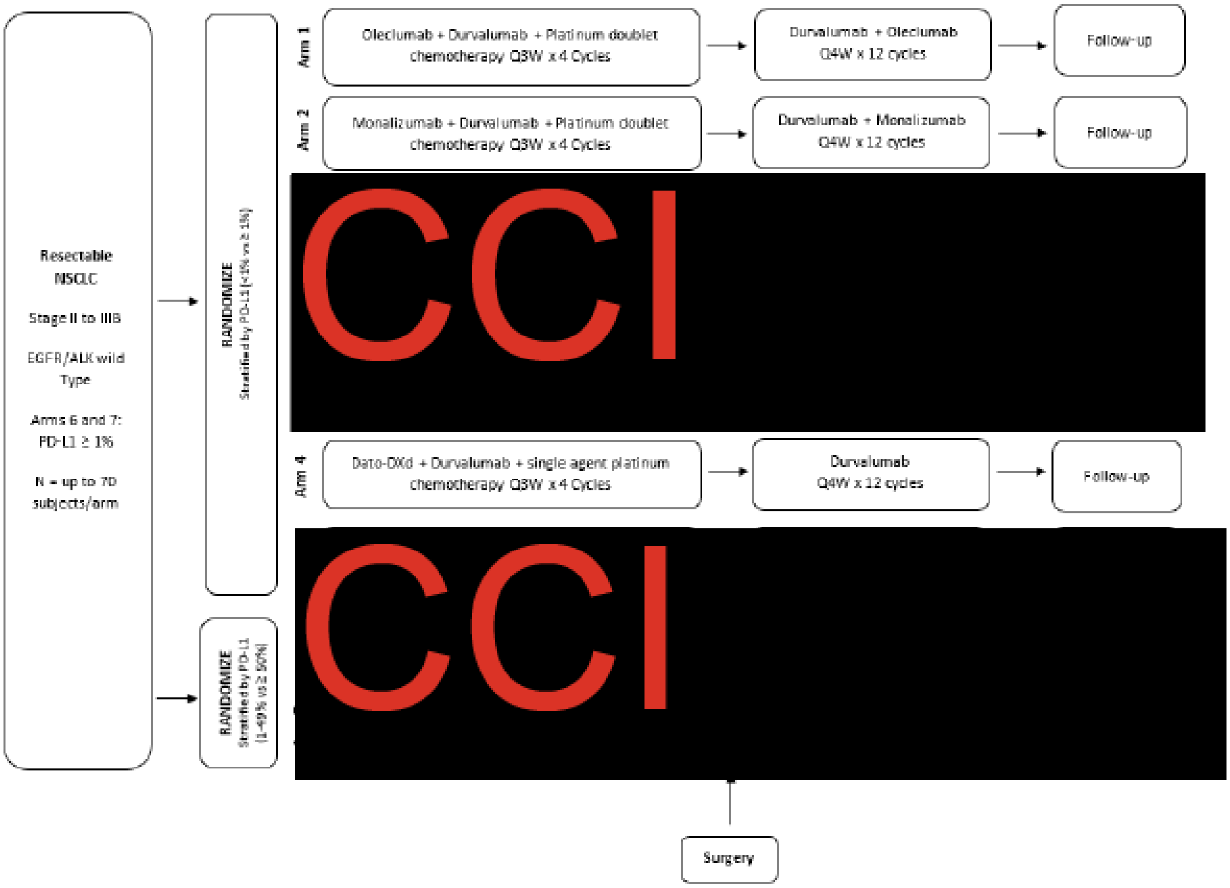

[redacted]

NOTE: Enrolment into individual treatment arms may be stopped on recommendation by the SRC due to safety concerns or if planned interim analyses show safety concerns or lack of efficacy. New combination treatment arms may be added based on emerging nonclinical and clinical data via a protocol amendment.
NOTE: Patients enrolled into Arms 1 to 5 will be stratified by baseline PD-L1 expression status (< 1% versus ≥ 1%). Patients enrolled in Arms 6 and 7 will be restricted to baseline PD-L1 status ≥ 1% and stratified by expression (1% to 49% versus ≥ 50%).
A randomisation method with dynamically changing allocation ratio of treatment assignment will be employed to account for fluctuations in the number of enrolling treatment arms over the course of the study and to allow an increase in enrolment to one or more treatment arms at the discretion of the Sponsor and/or recommendation of the SRC. [redacted]. When Arms 6 and 7 are open to enrolment, no other arm will be open to enrolment concurrently. Any changes to the allocation ratio will be communicated in a timely manner to the Investigators and patients will be informed by the Investigator or delegate of the enrolling treatment arms at the time of informed consent.
Abbreviations: ALK: anaplastic lymphoma kinase; CTX: platinum doublet chemotherapy; Dato-DXd: datopotamab deruxtecan; EGFR: epidermal growth factor receptor; NSCLC: non-small cell lung cancer; PD-L1: Programmed death-ligand 1; Q2W: every 2 weeks; Q3W: every 3 weeks; Q4W: every 4 weeks; SRC: Safety Review Committee.
[a] Recruitment closed at [redacted] patients, no additional patients will be enrolled in Arm 3A.

1.3 Schedule of Activities

The screening procedures for this study are identical across all treatment arms and are shown in Table 1.

The Schedule of Activities for neoadjuvant and adjuvant treatment after a patient is randomised can be found in the relevant appendix for each arm.

	**Neoadjuvant treatment/Pre-Surgery (4 cycles)**	**Adjuvant treatment/Post-Surgery (1 year)**	**Appendix**
**Arm 1**	Oleclumab + durvalumab + platinum doublet chemotherapy (Q3W)	Oleclumab + durvalumab (Q4W)	Appendix K
**Arm 2**	Monalizumab + durvalumab + CTX (Q3W)	Monalizumab + durvalumab (Q4W)	Appendix L
**Arm 3A**[a]	Volrustomig [redacted] mg + CTX ([redacted])	Volrustomig [redacted] mg [redacted]	Appendix M
**Arm 3B**	Volrustomig + CTX [redacted]	Volrustomig [redacted] mg [redacted]	Appendix M
**Arm 3C**	Volrustomig + CTX [redacted]	Volrustomig [redacted] mg [redacted]	Appendix M
**Arm 4**	Dato-DXd + durvalumab + single agent platinum chemotherapy (Q3W)	Durvalumab (Q4W)	Appendix N
**Arm 5**	AZD0171 + durvalumab + CTX [redacted]	AZD0171 [redacted] + Durvalumab [redacted]	Appendix O
**Arm 6**	Rilvegostomig + CTX [redacted]	Rilvegostomig [redacted]	Appendix P
**Arm 7**	Dato-DXd + Rilvegostomig + single agent platinum chemotherapy [redacted]	Rilvegostomig [redacted]	Appendix Q

Abbreviations: CTX: platinum doublet chemotherapy; Q2W: every 2 weeks; Q3W: every 3 weeks; Q4W: every 4 weeks.

[a] Recruitment closed at [redacted] patients, no additional patients will be enrolled in Arm 3A.

The procedures for patients who have completed or discontinued treatment are identical across all treatment arms and are shown in Table 2.

Table 1 Schedule of Activities for the Screening Period: All Patients

	Screening [a]	For details, see CSP Section or Appendix
**Week**	**-4 to -1**	
**Day**	**-28 to -1**	
**Informed consent**		
Informed consent: study procedures [b]	X	Section 5 and Appendix A 3
Informed consent: [redacted] sample (whole blood) [redacted]	X	Section 8.7.2 and Appendix D
**Study procedures**		
Physical examination (full)	X	Section 8.2.2
Vital signs [c]	X	Section 8.2.3
ECG [d]	X	Section 8.2.4
ECHO/MUGA [e]	X	Section 8.2.5
Concomitant medications	X	Section 6.5
Demography, including baseline characteristics and tobacco use	X	Section 5.1
Brain MRI (preferred) or brain CT with IV contrast	X	Appendix F
Contrast-enhanced CT/MRI scan of the chest and abdomen (including adrenal glands) and PET scan [f, g]	X	Section 6.1.3 and Section 8.1
Pulmonary function testing and cardiac risk assessment [h]	X	Section 8.2.7
Ophthalmological assessments [i]	X	Section 8.2.8
Eligibility criteria [j]	X	Section 5 and Section 6.1.3
**Laboratory assessments**		
Clinical chemistry [k]	X	Section 8.2.1
Cortisol	X	Section 8.2.1
Haematology	X	Section 8.2.1
aPTT and INR	X	Section 8.2.1
TSH (reflex free T3 or Total T3, or free T4) [l]	X	Section 8.2.1
Urinalysis	X	Section 8.2.1
Hepatitis B and C	X	Section 8.2.1

	**Screening [a]**	**For details, see CSP Section or Appendix**
**Week**	**-4 to -1**	
**Day**	**-28 to -1**	
HIV test [m]	X	Section 8.2.1
Pregnancy test [n]	X	Section 8.2.1
**Monitoring**		
WHO/ECOG performance status	X	Section 8.2.6
AE/SAE assessment [o]	X	Section 8.3
**Other assessments and assays**		
ctDNA	X	Section 8.6.3 and Section 8.7.1
Mandatory tumour specimen (newly acquired or archived [≤ 6 months old]) [b]	X	Section 8.6.1
PD-L1 testing [p]	X	Section 8.6.1
EGFR/ALK testing [q]	X	Section 8.6.1
Whole blood for [redacted] Expression	X	Section 8.6.3
Serum samples for circulating soluble factors	X	Section 8.6.3
Plasma samples for circulating soluble factors	X	Section 8.6.3
Whole blood for DNA analyses	X	Section 8.6.3
Whole blood for PBMC	X	Section 8.6.3
**Efficacy evaluation**		
RECIST 1.1 tumour assessments on CT and/or MRI scans [f]	X	Section 8.1.1 and Appendix F

[a] The 28-day screening period begins when the patient signs the consent form. Written informed consent and any locally required privacy act document authorisation must be obtained prior to performing any protocol-specific procedures, including screening/baseline evaluations. If laboratory or imaging procedures were performed for alternate reasons prior to signing consent, these can be used for screening purposes with consent of the patient. However, all screening laboratory and imaging results must have been obtained within 28 days of randomisation unless otherwise specified (eg, except for PET scan as described in footnote g and pulmonary function testing as described in footnote h).

[b] All patients will be required to provide consent to supply a sample of their tumour (archived or newly acquired biopsy) for enrolment into this study. This consent is included in the main patient ICF. Archived biopsies (tissue block preferred, or unstained slides) collected within ≤ 6 months of study enrolment or undergoing a pre-treatment tumour biopsy are both acceptable. Sites need to confirm the nature of archival tumour material (biopsy vs others [cytology]) and specifications before randomisation (see laboratory manual for additional details).

[c] Body weight is recorded at each visit along with vital signs.

[d] Any clinically significant abnormalities detected require triplicate ECG results.

[e] ECHO/MUGA will be performed at screening to assess LVEF. ECHO/MUGA is mandatory for Arms 3A, 3B, 3C, 6 and 7 and all patients must be assessed at screening when Arm 3A, 3B, 3C, 6 and 7 are open for enrolment. When Arm 3A, 3B, 3C, 6 or 7 are not open for enrolment, ECHO/MUGA can be done at the Investigator's discretion.

[f] A CT/MRI scan of the chest and abdomen (including the entire liver and both adrenals) must be performed within 28 days prior to and as close as possible to randomisation, and this scan is designated as the "Baseline" scan (ie, lesions are classified as baseline TLs or NTLs). A chest CT alone can be used if it includes the entire liver and both adrenals.

[g] A PET scan done as part of clinical practice within 42 days prior to randomisation is acceptable and doesn't need to be repeated during screening. If there are findings on screening CT scan that warrants a confirmation with PET scan then a PET scan must be repeated during the 28-day screen period.

[h] Pulmonary function testing is mandatory at screening and may be repeated at Investigator's or surgeon's discretion prior to surgery to inform fitness for surgery. A pulmonary function test done as part of clinical practice within 42 days prior to randomisation is acceptable and doesn't need to be repeated during screening.

[i] Ophthalmologic assessments including, but not limited to, visual acuity testing, slit lamp examination, intraocular pressure measurement, fundoscopy, and fluorescein staining will be performed at screening by an ophthalmologist, or if unavailable, another licensed eye care provider. Ophthalmologic assessments are mandatory for Dato-DXd and all patients must be assessed at screening when Arm 4 or 7 is open for enrolment. When Arm 4 or 7 is not open for enrolment, ophthalmologic assessments can be done at the Investigator's discretion. Please refer to the Dato-DXd Ophthalmologic Assessment Manual for further details.

[j] At screening, complete surgical resection of the primary NSCLC must be deemed achievable, as assessed by a multidisciplinary evaluation, which must include a thoracic surgeon who performs lung cancer surgery as a prominent part of his/her practice. Nodal status should be investigated with whole body 18F-fluoro-deoxyglucose PET, plus contrast-enhanced CT. If PET/CT scan is positive in the mediastinum, or if scan is negative but there is T > 3 cm, central tumour, or cN1, then it is recommended that nodal status be proven by biopsy via endobronchial ultrasound, mediastinoscopy, or thoracoscopy. See Section 5.1 (surgery eligibility) and Section 6.1.3 (preoperative mediastinal lymph node staging) for more details.

[k] Note: Anti-thyroid antibodies will be measured on all patients at Screening visit. LFTs and troponin will be measured on all patients at Screening visit.

[l] Free T3 or Total T3, or free T4 will only be measured if TSH is abnormal or if there is clinical suspicion of an AE related to the endocrine system.

[m] If Arm 4, 6, or 7 is open for enrolment, all participants must be tested for HIV during the screening period if acceptable by local regulations or an IRB/EC. When Arms 4, 6, and 7 are not open for enrolment, HIV testing can be done at the Investigator's discretion.

[n] For women of childbearing potential only. A serum pregnancy test is required. Women of childbearing potential are required to have a pregnancy test within 3 days prior to the first dose of study interventions. Pregnancy test may occur on Day 1, but results must be available and reviewed by the treating physician or Investigator prior to commencing an infusion.

[o] For AEs/SAEs reported during screening, additional information such as medical history and concomitant medications may be needed.

[p] PD-L1 expression determination (< 1%, 1% to 49%, ≥ 50%) is required prior to randomisation for all patients. Local PD-L1 testing with a local regulatory-approved analytically validated assay is required; SP263 antibody is preferred if available.

[q] *EGFR/ALK* status must be confirmed as wild-type prior to randomisation with the following exceptions: Patients with squamous cell carcinoma do not need to be tested for *ALK* status. Results from local *EGFR/ALK* testing are accepted for this study. Results from local *EGFR/ALK* testing should be from a well-validated, local regulatory-approved test.

Abbreviations: *ALK*: Anaplastic lymphoma kinase; AE: Adverse event; aPTT: Activated partial thromboplastin time; CSP: Clinical study protocol; CT: Computed tomography; ctDNA: Circulating tumour DNA; Dato-DXd: datopotamab deruxtecan; DNA: Deoxyribonucleic acid; ECG Electrocardiogram; ECHO: Echocardiogram; ECOG: Eastern Cooperative Oncology Group; *EGFR*: Epidermal growth factor receptor; ICF: Informed consent form; INR: International normalised ratio; IV: Intravenous; LFT: Liver function test; LVEF: Left ventricular ejection fraction; MRI: Magnetic resonance imaging; MUGA: multigated acquisition scans; NSCLC: Non-small cell lung cancer; NTL: Non-target lesion; PBMC: Peripheral blood mononuclear cells; *PD-L1*: Programmed cell death ligand-1; PET: Positron emission tomography; RECIST: Response Evaluation Criteria in Solid Tumours; SAE: Serious adverse event; T: Tumour; T3: Triiodothyronine; T4: Thyroxine; TL: Target lesion; TSH: Thyroid-stimulating hormone; WHO: World Health Organisation.

Table 2 Schedule of Activities for Patients who have Completed/Discontinued Treatment: All Patients

	Time since last dose or last study intervention[a]								
Evaluation	Day (± 3)/EOT	Months (± 1 week)						12 months and every 3 months (± 2 weeks) /EOS	For details, see CSP Section or Appendix
	30	2	3	4	6	8	10		
Physical examination (full)	X								Section 8.2.2
Vital signs	X								Section 8.2.3
Weight	X	X	X						Section 8.2.2
Pregnancy test [b]	X	As clinically indicated [c]							Section 8.2.1
AE/SAE assessment	X	X	X						Section 8.3
Concomitant medications	X	X	X						Section 6.5
WHO/ECOG performance status	At timepoints consistent with tumour assessments; at 30, 60, and 90 days; and then at initiation of subsequent anticancer therapy [d]								Section 8.2.6
Subsequent anticancer therapy [e, f]	X	X	X	X	X	X	X	X	Section 7.1.1
Survival status		X	X	X	X	X	X	X	Section 7.1.2
Ophthalmological assessments [g]	X								Section 8.2.8
Haematology	X	X	X						Section 8.2.1
Clinical chemistry	X	X	X						Section 8.2.1
ctDNA [h]	X	X	X					X (at 12 and 24 months)	Section 8.6.3 and Section 8.7.1
Urinalysis	As clinically indicated								Section 8.2.1
TSH (reflex free T3 or Total T3, or free T4 [i])	X	X	X						Section 8.2.1
Optional tumour biopsy specimen	X[j]								Section 8.6.1 and Section 8.6.4
Durvalumab immunogenicity assessment (ADA sampling) to identify ADA responses [k]	X		X						Section 8.5.2

	Time since last dose or last study intervention[a]								
Evaluation	Day (± 3)/EOT	Months (± 1 week)						12 months and every 3 months (± 2 weeks) /EOS	For details, see CSP Section or Appendix
	30	2	3	4	6	8	10		
Oleclumab, Monalizumab, volrustomig, Dato-DXd, AZD0171 or rilvegostomig immunogenicity assessment (ADA sampling) to identify ADA responses [k]	X		X						Section 8.5.2
Tumour assessment (CT or MRI) (RECIST 1.1)[l,m]	Follow-up scans are acquired Q12W ± 1 week (relative to the date of surgery) until week 48; Q24W ± 2 weeks (relative to the date of surgery) until week 192 (approximately 4 years); and then Q48W ± 2 weeks (relative to the date of surgery) thereafter, until RECIST 1.1-defined radiological PD, consent withdrawal, death, or study completion as determined by Sponsor. This on-study schedule MUST be followed regardless of any delays in dosing and regardless of tumour margins. These follow-up scans will use the original neoadjuvant screening scan as a baseline scan for RECIST 1.1 assessments.								Section 8.1.1 and Appendix F

a EOT visit date is calculated from the time since last dose or last study intervention (includes surgery), whichever occurs later. Cases where an EOT visit cannot be done within the protocol specified window due to medical reasons should be discussed with the Sponsor.
b For women of childbearing potential only. A urine or serum pregnancy test is acceptable.
c A monthly pregnancy test is required during the washout period after the last treatment administration. For patients receiving multiple study interventions, the longest washout period must be followed. Refer to Section 5.3 for details on drug washout periods.
d WHO/ECOG performance status should also be collected at other site visits that the patient attends, if appropriate site staff are available to collect such information. In addition, WHO/ECOG performance status should be provided when information on subsequent anticancer therapy is provided, where possible.
e Details of any treatment for NSCLC (including surgery) post the last dose of study interventions must be recorded in the eCRF. At minimum, collect the start date and description of the subsequent anticancer therapy.
f For patients who discontinue their assigned study interventions following progression, available readings of CT/MRI from local practice will be collected from patients' medical charts while information on subsequent anticancer treatment is collected.
g Ophthalmological assessments are only required for patients randomised to Arm 4 and 7. Ophthalmologic assessments including, but not limited to, visual acuity testing, slit lamp examination, intraocular pressure measurement, fundoscopy, and fluorescein staining will be performed for all patients at screening and for patients randomised to Dato-DXd as clinically indicated and at the EOT visit by an ophthalmologist, or if unavailable, another licensed eye care provider.
h ctDNA will be collected if EOS visit is before 24 month follow-up visit.
i Free T3 or Total T3, or free T4 will only be measured if TSH is abnormal or if there is clinical suspicion of an AE related to the endocrine system.
j It is strongly recommended/encouraged to collect additional tumour biopsies, if they become available at disease progression or unscheduled procedures during the study.
k Immunogenicity samples for durvalumab and oleclumab, or monalizumab, volrustomig, Dato-DXd, AZD0171, or rilvegostomig are collected 90 days (3 months) (± 7 days) after treatment ends.

[l] A new primary malignancy confirmed by pathology is not considered an EFS or DFS event.
[m] If a pre-planned surgery date is not available for planning RECIST 1.1 follow-up scans on patients who did not undergo surgery (for reasons other than PD), the follow-up schedule will use 40-days after the last study interventions administration as the date of pre-planned surgery.

Abbreviations: ADA: Anti-drug antibody; AE: Adverse event; CSP: Clinical study protocol; CT: Computed tomography; ctDNA: Circulating tumour; Dato-DXd: datopotamab deruxtecan; DFS: disease-free survival; DNA: Deoxyribonucleic acid; eCRF: electronic case report form; ECOG: Eastern Cooperative Oncology Group; EFS: Event-free survival; EOT: End of treatment; EOS: End of study; MRI: Magnetic resonance imaging; NSCLC: Non-small cell lung cancer; PD: Progression of disease; Q12W: Every 12 weeks; Q24W: Every 24 weeks; RECIST: Response Evaluation Criteria in Solid Tumours; SAE: Serious adverse event; T3: Triiodothyronine; T4: Thyroxine; TSH: Thyroid-stimulating hormone; WHO: World Health Organisation.

2 INTRODUCTION

Study D9077C00001 (NeoCOAST-2) is a Phase II study assessing the efficacy and safety of neoadjuvant and adjuvant treatment in patients with resectable, early stage (Stage II to IIIB) NSCLC. The treatment arms are shown in Figure 1. New combination treatment arms can be added during the course of the study via a protocol amendment as novel agents of interest become available for testing in this population of patients.

2.1 Disease Background

Lung cancer is the second most common cancer in both men and women, with an estimated 235,760 new cases of lung cancer in the US in 2021 and remains the main cause of cancer related deaths worldwide. Non-small cell lung cancer represents approximately 85% of all lung cancers, and 30% of patients present with resectable NSCLC at diagnosis (AJCC Cancer Staging Manual, 8th Edition, American Cancer Society, 2018). Resectable, early-stage NSCLC is considered a potentially curable disease, and the SoC for decades was surgery alone or surgery with adjuvant or neoadjuvant platinum-based doublet chemotherapy (National Comprehensive Cancer Network, 2022). Recently, PD-L1 agents have demonstrated a role in resectable NSCLC in the perioperative setting. Atezolizumab in a phase 3 trial (IMPower010) showed pronounced DFS benefit as compared to best supportive care after adjuvant chemotherapy in resectable disease whose tumours expressed PD-L1 of 1% or more of tumour cells (Felip et al, 2021) and is currently approved in this setting. Likewise, nivolumab in combination with platinum-doublet chemotherapy prior to surgery has been approved as neoadjuvant treatment in the US (Forde et al, 2022). Prior to this new approval, up to 55% of patients eventually relapse after surgery and die from NSCLC (Uramoto and Tanaka, 2014).

Based on the National Cancer Data Base dataset, patients had 5-year survival rates ranging from approximately 70% for Stage IA1 NSCLC to 10% for Stage IIIB disease NSCLC (Chansky et al, 2017). The use of adjuvant chemotherapy with a platinum-based regimen has been shown to provide survival benefit in patients with resected, early-stage NSCLC; a quantitative meta-analysis of multiple clinical trials evaluating adjuvant chemotherapy demonstrated a 4% to 5% improvement in OS with the use of adjuvant chemotherapy following surgery compared to surgery alone (Burdett et al, 2015). In addition, preoperative neoadjuvant chemotherapy has also been studied in several trials in patients with resectable NSCLC (Burdett et al, 2006). A meta-analysis of 7 trials showed that neoadjuvant chemotherapy improved OS versus surgery alone (5-year OS rate of 20% vs 14%, respectively).

Compared with adjuvant chemotherapy in early-stage NSCLC, the use of neoadjuvant chemotherapy resulted in a higher proportion of patients receiving the planned chemotherapy

regimen, which supports the notion that early-stage NSCLC patients have a more favourable clinical condition before compared to after a major surgical lung resection (Felip et al, 2010).

In addition, patients with resectable NSCLC treated with neoadjuvant chemotherapy have higher rates of pathological tumour response (complete response and/or ≤ 10% residual viable tumour cells) within the resected specimen compared to patients who underwent surgery alone, which may predict clinical benefit and correlates with improved outcomes (Cascone et al, 2018, Felip et al, 2010, Machtay et al, 2004, Pataer et al, 2012). Major pathological response (≤ 10% residual viable tumour) has shown to be a predictable surrogate endpoint for survival in resectable lung cancers (Hellmann et al, 2014).

The role of PD-1/PD-L1 inhibition has been evaluated for the treatment of resectable, early-stage NSCLC in the neoadjuvant setting. Recent results of the CheckMate-816 study have shown the benefit of nivolumab in combination with chemotherapy as neoadjuvant treatment in patients with resectable NSCLC (Forde et al, 2021). In this Phase III study, the combination of nivolumab and chemotherapy compared to chemotherapy demonstrated a statistically significant improvement in median EFS (31.6 months vs. 20.8 months; HR = 0.63; [97.38% CI, 0.43 to 0.91]; P=0.005), pCR (24.0% vs 2.2%; OR = 13.94 [99% CI, 3.49 to 55.75]; P < 0.001). The benefit was consistent across disease stages, histologies, TMB, and PD-L1 expression levels (Forde et al, 2022). In an exploratory subset analysis, ctDNA clearance was also more frequent in the combination treatment compared to chemotherapy alone and appeared to be associated with pCR. Nivolumab in combination with chemotherapy showed a tolerable safety profile and did not impact the feasibility of surgery.

Other Phase III studies evaluating the combination of immunotherapy and chemotherapy in the neoadjuvant and adjuvant setting are ongoing, including a study of durvalumab in combination with chemotherapy *vs* placebo and chemotherapy followed by surgery and adjuvant treatment with durvalumab *vs* placebo (AEGEAN study, NCT03800134) and a study of pembrolizumab in combination with chemotherapy vs placebo and chemotherapy followed by surgery and adjuvant treatment with pembrolizumab vs placebo (KEYNOTE-671, NCT03425643).

In the Phase III AEGEAN study, the combination of durvalumab and chemotherapy compared to chemotherapy demonstrated EFS benefit at 12-months, with EFS observed in 73.4% of the patients who received durvalumab (95% CI, 67.9 to 78.1), as compared with 64.5% of the patients who received placebo (95% CI, 58.8 to 69.6); HR for disease progression, recurrence, or death was 0.68 (95% CI, 0.53 to 0.88; P=0.004). Additionally, durvalumab and chemotherapy demonstrated significantly greater pathological responses vs those receiving placebo (17.2% vs. 4.3% at the final analysis; difference, 13.0 percentage points; 95% CI, 8.7 to 17.6; P<0.001 at interim analysis) (Heymach et al, 2023).

In the Phase III KEYNOTE-671 study, the combination of pembrolizumab and chemotherapy

compared to chemotherapy significantly improved OS, reducing the risk of death by 28% (HR=0.72 [95% CI, 0.56-0.93]; one-sided p=0.00517). For subjects who received the pembrolizumab based regimen, median OS was not reached (95% CI, NR-NR) vs 52.4 months (95% CI, 45.7-NR) for patients who received the chemotherapy-placebo regimen. The 36-month OS rates were 71.3% for patients who received pembrolizumab vs 64.0% for patients who received the chemotherapy-placebo regimen. Additionally, statistically significant EFS improvements were observed in the pembrolizumab arm vs chemotherapy arm (47.2 months [95% CI, 32.9-NR] vs 18.3 months [95% CI, 14.8-22.1], respectively; HR=0.59 [95% CI, 0.48-0.72]). The 36-month EFS rates were 54.3% for patients who received the pembrolizumab vs 35.4% for patients who received the chemotherapy-placebo regimen (Spicer et al, 2023b).

Overall, early-stage NSCLC, despite being potentially curable, has a risk of relapse and death due to disease progression. Thus, new therapies are needed to further improve the long-term prognosis for patients with resectable, early-stage NSCLC.

2.2 Study Interventions Background

A summary of the background for the study interventions used in each arm are provided in the relevant appendix.

2.3 Study Rationale

Five-year survival rates in early-stage NSCLC range from approximately 70% for Stage IA1 disease to 20% for Stage IIIA disease and 10% for Stage IIIB disease, with the majority of patients experiencing post-surgical relapse and disease recurrence (Chansky et al, 2017, Goldstraw et al, 2007). Adjuvant platinum-based chemotherapy provides an approximate 5% increase in survival benefit compared to surgery alone, with rates of overall Grade 3 to 4 toxicity of 66%.

The use of combination chemotherapy is a mainstay of oncology therapy. The goal of combination chemotherapy is to utilise agents that affect cancer cells by different mechanisms, thus reducing the risk of developing resistance. Moreover, given the synergistic effect of chemotherapy and immunotherapy in cancer therapy, several studies have been designed to assess the efficacy and safety of immunotherapy combined with chemotherapy in the neoadjuvant treatment of early-stage NSCLC (Awad et al, 2023, Bai et al, 2020). CheckMate-816 is the first Phase III study showing the benefit of immunotherapy in terms of improvement in pCR rate in combination chemotherapy in patients with resectable NSCLC (refer to Section 2, disease background for details). A study of durvalumab in combination with chemotherapy vs placebo and chemotherapy followed by surgery and durvalumab or placebo is currently ongoing (AEGEAN study, NCT03800134). Regular scheduled safety independent data monitoring committee reviews have been conducted, and no safety concerns were raised. Data from the AEGEAN trial demonstrated that neoadjuvant treatment with

durvalumab plus chemotherapy followed by surgery and durvalumab monotherapy provided a statistically significant and meaningful improvement in pCR rate and 24 month EFS (17.2% and 63.3%, respectively) over those treated with chemotherapy alone (4.3% and 52.4%, respectively) (Heymach et al, 2023). These data, along with surgical resection rates are consistent with results from CheckMate-816.

In addition, other studies have shown the benefit of combining immunotherapy with chemotherapy in the locally advanced resectable setting. A Phase II study of neoadjuvant chemotherapy with nivolumab in stage IIIA NSCLC (Provencio et al, 2020), enrolled 46 patients of which 41 had surgical resection. Overall, 83% of patients who had resected disease had mPR, of whom 63% had a pCR. The primary endpoint was PFS at 24 months which was 77.1%. None of reported AEs led to treatment discontinuation, dose reduction, or were associated with surgery delays or deaths. A second study that evaluated atezolizumab and chemotherapy (Shu et al, 2020) in patients with stage IB-IIIA enrolled 30 patients reported mPR rate of 57% and 33% pCR. The median DFS was 17.9 months and median OS was not reached. The treatment was safe and well tolerated with AEs consistent with published data with chemotherapy in metastatic NSCLC. Immune-related AEs were as expected and manageable.

The NeoCOAST-2 study, utilising a neoadjuvant and adjuvant approach to assess the role of immuno-check point inhibitors with chemotherapy and with other NSCLC targeting novel agents has several advantages: first, the combination may result in pathological response and/or downstaging prior to surgery; second, early treatment of micrometastatic disease and avoidance of treatment delay due to post-operative complications; and finally, the resected tumours can be analysed, which provides a unique opportunity to perform correlative biomarker studies and investigate changes in the tumour microenvironment. Predictive biomarkers or underlying resistance mechanisms may be identified, which will help inform future combination therapy studies.

Studies have also shown the potential benefit of neoadjuvant and adjuvant treatment in patients with multistation disease. The NADIM trial reported OS at 3 years > 80% with 54% of patients having N2 multistation disease (Provencio et al, 2020; Provencio et al, 2022). Moreover, 37% of patients enrolled in the NADIMII trial had N2 multistation disease with a reported pCR rate of 36.2% in the overall population (compared to 6.8% in the control arm) and surgery completion rate of 91% (compared to 69% in the control arm) (Provencio-Pulla M et al, 2022). Based on these promising data, the NeoCOAST-2 study has included patients with stage IIIB disease according to the latest NSCLC staging system (AJCC Cancer Staging Manual, 8th Edition).

Additionally, nonclinical evidence supports that immune response to neoadjuvant immunotherapies may be more effective compared to adjuvant approach because neoadjuvant

immunotherapy induces the release of tumour-specific antigens from dying tumour cells, which may act as a vaccine to further prime and expand tumour specific T cells (Li et al 2021

Li TR, Chatterjee M, Lala M, Abraham AK, Freshwater T, Jain L, et al. Pivotal dose of pembrolizumab: a dose-finding strategy for immuno-oncology. Clin Pharmacol Ther. 2021 Jul;110(1):200-209.

Liu et al, 2016).

The use of a neoadjuvant approach offers the possibility for assessing clinical and molecular biomarkers that may correlate with sensitivity to therapy and possibly, long-term outcomes.

A rationale for the use of each study intervention/combination of study interventions is provided in the relevant appendix.

2.4 Benefit/Risk Assessment

The study will be performed in accordance with ethical principles that have their origin in the Declaration of Helsinki and are consistent with ICH- GCP, and applicable regulatory requirements. More detailed information about the known and expected benefits, risks, and reasonably expected AEs of durvalumab, oleclumab, monalizumab, volrustomig, Dato-DXd, AZD0171, and rilvegostomig can be found in the respective IB for each study intervention.

2.4.1 Potential Benefits

CheckMate-816 has shown that blockade of PD-l in combination with chemotherapy compared to chemotherapy alone in the neoadjuvant setting yield statistically significant pCR (24% vs 2.2%) and EFS (31.6 months vs 20.8 months) (Forde et al, 2022). Likewise, pCR has also been associated with improved survival (Felip et al, 2010, Machtay et al, 2004, Pataer et al, 2012). Other studies have shown that neoadjuvant and adjuvant PD-1 blockade significantly improved pCR, EFS, and OS compared to chemotherapy alone (AEGEAN, Heymach et al, 2023; KEYNOTE-671, Wakelee et al, 2023; CheckMate-816, Spicer et al, 2023a). Additionally, several studies in metastatic NSCLC evaluating PD-1/PD-L1 inhibitors and other immunotherapy agents in combination with chemotherapy are now standard treatment in certain settings in NSCLC. These studies include: KEYNOTE-189 (pembrolizumab; PD-1 inhibitor) (Freeman et al 2000

Freeman GJ, Long AJ, Iwai Y, Bourque K, Chernova T, Nishimura H, et al. Engagement of the PD-1 immunoinhibitory receptor by a novel B7 family member leads to negative regulation of lymphocyte activation. J Exp Med. 2000;192(7):1027-34.

Frewer et al, 2016, Gandhi et al, 2018), KEYNOTE-407 (pembrolizumab; PD-1 inhibitor) (Paz-Ares et al, 2018), the IMpower150 study (atezolizumab; PD-L1 inhibitor, and bevacizumab; VEGF inhibitor) (Socinski et al, 2018), CheckMate 9LA (nivolumab; PD-1

inhibitor; ipilimumab; CTLA-4 inhibitor) (Paz-Ares et al, 2021) and POSEIDON study (durvalumab; PD-L1 inhibitor ± tremelimumab; CTLA-4 inhibitor) (Johnson et al, 2021).

Data from the ipilimumab/nivolumab arm of CheckMate-816 also support the additive role of neoadjuvant CTLA-4 blockade in combination with PD-1 inhibition in resectable NSCLC. In the ipilimumab/nivolumab arm of CheckMate-816, one dose of ipilimumab (1mg/kg) plus 3 doses of nivolumab (3mg/kg) in the neoadjuvant setting showed improvements in pCR (20.4%) and median EFS (54.8 months) vs neoadjuvant chemotherapy alone, which yielded a pCR rate of 4.6% and median EFS of 20.9 months (Awad et al, 2023).

Additionally, the NEOSTAR trial compared 3 cycles of neoadjuvant nivolumab + chemotherapy to 1 cycle of neoadjuvant ipilimumab (1 mg/kg) with 3 cycles of nivolumab and chemotherapy. While the pCR rates between the two arms were identical in the ITT populations at 18.2%, the mPR rates were significantly higher in the ipilimumab + nivolumab + chemotherapy arm (50%) compared to the nivolumab + chemotherapy arm (32.1%). The median EFS was not reached on either arm; the EFS rate was 77% at 24 months on the ipilimumab + nivolumab + chemotherapy arm compared to 73% at 24 months on the nivolumab + chemotherapy arm. While this data is immature and the numbers are small (n=22 for both arms), it is supportive of the benefit at least one dose of neoadjuvant CTLA-4 blockade in combination with PD-1 inhibition and chemotherapy for the treatment of resectable NSCLC (Cascone et al, 2023).

In the first-in-human Phase I dose-escalation/dose-expansion study evaluating Dato-DXd in patients with advanced NSCLC and other tumour types (TROPION-PanTumor01), Dato-DXd was shown to be associated with durable antitumour activity and a manageable safety profile in patients with pre-treated advanced NSCLC (Garon et al, 2021). Additionally, in the Phase Ib study of Dato-DXd in combination with pembrolizumab with or without platinum chemotherapy in patients with advanced or metastatic NSCLC (TROPION-Lung02), an interim analysis showed promising response rates in the overall population and in patients receiving first-line therapy, across PD-L1 expression levels (Levy et al, 2022).

In the first-in-human study of AZD0171 in patients with advanced solid tumours (MSC-1-101; NCT03490669), AZD0171 was shown to be safe and well tolerated in patients with advanced solid tumours. Clinical activity was evaluable in 38 patients. None had a CR or PR. The BOR was stable disease for 2 consecutive tumour assessments for 9 (23.7%) patients, and stable disease followed by progressive disease in 4 (10.5%) patients; 25 (65.8%) patients had progressive disease. The DCR was 23.7% (95% CI 11.4% to 40.2%). The overall median PFS was 5.9 weeks (95% CI 2.1 to 28.3 weeks), with 9 patients having a PFS of > 16 weeks. Although antitumour activity was limited, stabilisation of total circulating LIF concentrations and biomarker evidence of immunological reprogramming in the tumour microenvironment support the therapeutic hypothesis that AZD0171 could effectively block LIF signaling,

activate immune-mediated antitumour effects, and inhibit cancer stem cells (Borazanci et al, 2022).

Notably, AZD0171 monotherapy increased the frequency of CD8+ T cells in matched on treatment vs pre-treatment biopsies in a subset of patients. The potential for AZD0171 to reinvigorate T cell response or tumour infiltrating lymphocytes may be of clinical benefit in patients with basal detectable tumoural CD8+ T cells especially when combined with an immune checkpoint inhibitor. Therefore, targeting LIF with AZD0171 in combination with durvalumab and standard-of-care chemotherapy may lead to more synergistic durable responses in 1L mPDAC patients.

In the first in human Phase I/II ARTEMIDE-01 study in stage IV NSCLC (NCT04995523), rilvegostomig was shown to be safe and well tolerated with encouraging efficacy in CPI-naïve subjects with PD-L1 expression ≥ 1%. As of the 07 July 2024 DCO, in participants with PD-L1 TPS 1% to 49% [redacted] mg dose, n = 31), 9 (29.0%) participants achieved PR and 11 (35.5%) achieved SD ≥ 9 weeks. A total of 5 patients remain in response out of the 9 confirmed PRs. The median duration of response was estimated as 6.4 months (95% CI 4.2, NC). In participants with PD-L1 TPS ≥ 50% receiving [redacted] mg (n = 34), 17 (50.0%) participants achieved PR and 13 (38.2%) achieved SD ≥ 9 weeks, including 4 (11.8%) unconfirmed PRs pending confirmation, totalling an unconfirmed ORR of 61.8%. A total of 13 patients remain in response out of the 17 confirmed PRs (Hiltermann et al 2024).

Rilvegostomig has also been evaluated in combination with chemotherapy in MAGELLAN (D933IC00001), a Phase IB, open-label, multi-centre study to determine the efficacy and safety of durvalumab and/or novel oncology therapies, with or without chemotherapy, for 1L stage IV NSCLC. Subjects (PD-L1 < 50%) with stage IV NSCLC enrolled in Cohort B5 (n[redacted] received rilvegostomig + carboplatin/cisplatin + pemetrexed. As of the final analysis DCO of 23 May 2023, the confirmed ORR was [redacted]%. [redacted] ([redacted]%) subject had a confirmed PR, [redacted] [redacted]%) had disease progression, and [redacted]%) had SD, of which one [redacted]%) had an unconfirmed PR.

The NeoCOAST-2 study will allow patients with resectable, early-stage (Stage II to IIIB) NSCLC patient to receive chemotherapy in combination with a PD (L)-1 inhibitor and/or other novel therapies that potentially can improve upon current available therapies.

Selection of novel agents is based on nonclinical and clinical data supporting a favourable interaction with chemotherapy in this patient population. Novel agents will be evaluated in both the neoadjuvant and adjuvant settings.

2.4.2 Potential Risks

2.4.2.1 Durvalumab

Risks with durvalumab include, but are not limited to, diarrhoea/colitis, pneumonitis/ILD, endocrinopathies (ie, events of hypophysitis/hypopituitarism, thyroiditis, adrenal insufficiency, hyper- and hypothyroidism, type I diabetes mellitus and diabetes insipidus), hepatitis/increases in transaminases, nephritis/increases in creatinine, rash/dermatitis (including pemphigoid), myocarditis, myositis/polymyositis, immune thrombocytopenia, infusion related reactions, hypersensitivity reactions, pancreatitis, serious infections, encephalitis, meningitis, and other rare or less frequent inflammatory events including uveitis, immune-mediated arthritis, and neuromuscular toxicities (eg, Guillain-Barré syndrome, myasthenia gravis).

For information on all identified and potential risks with durvalumab alone or in combination with other anticancer therapies, refer to the current version of the durvalumab IB.

For mitigation strategies, please refer to specific TMGs and dose modification information (Section 8.3.15).

2.4.2.2 Combination Therapy with Durvalumab Plus Oleclumab, Monalizumab, Dato-DXd or AZD0171

Oleclumab plus durvalumab and durvalumab plus monalizumab were evaluated in the NeoCOAST study (Appendix K 4 and Appendix L 4). The AEs of the combinations were similar to durvalumab monotherapy, and mostly of Grade 1 and 2 intensities. There were no treatment discontinuations, deaths or delays to surgery due to AEs in the combination arms. Furthermore, the study was evaluated by a SRC (Cascone et al, 2022).

In a Phase Ib/II, 2 stage, open-label, multicentre, multi-arm platform study (Study D933LC00001 [BEGONIA]) evaluating the efficacy and safety of durvalumab with or without paclitaxel, in combination with novel oncology therapies, as first-line treatment for metastatic triple negative breast cancer, no dose-limiting toxicities have been reported in the first [redacted] patients receiving Dato-DXd 6.0 mg/kg in combination with durvalumab as of the DCO of 17 June 2021.

For Dato-DXd, the 2 most relevant risks considered for the benefit/risk assessment are the important identified risk of ILD/pneumonitis and the identified risk of IRR. Embryo-foetal toxicity is considered an important potential risk. In the ongoing Phase Ib study in advanced or metastatic NSCLC (Study DS1062-AU104 [TROPION-Lung04]) evaluating Dato-DXd in combination with durvalumab with or without carboplatin [redacted] patients in cohort [redacted] received Dato-DXd [redacted] mg/kg + durvalumab [redacted] mg [redacted]. As of DCO 28 September 2022, Grade ≥ 3 TEAEs occurred in [redacted] %) patients with the most common including [redacted] [redacted] %), hypokalaemia [redacted] %), anaemia [redacted] %), and pneumonia [redacted] %). [redacted] patients [redacted] %)

remained on study treatment at the time of analysis, with [redacted] patients [redacted]%) having discontinued all study treatment. The reasons for treatment discontinuation were progressive disease [redacted] patients [redacted]%]), clinical progression [redacted] patients [redacted]%]), and adverse event ([redacted] [[redacted]%]). No DLTs were reported.

Important potential risks for AZD0171 include IRRs, hepatotoxicity, and QT prolongation. Other potential risks for AZD0171 include fatigue, nausea, osteonecrosis of the jaw, and immunogenicity. These events were determined as potential risks based on clinical findings in individuals enrolled in a Phase I study (MSC-1-101; NCT03490669) with advanced solid tumours. Additional potential risks include hypersensitivity including severe allergic reactions which can be associated with the administration of mAbs. For information on all identified and potential risks with AZD0171, refer to the current AZD0171 IB.

In the completed Phase I study of AZD0171 in patients with advanced solid tumours (MSC-1-101; NCT03490669), AZD0171 as monotherapy was evaluated in 41 patients at increasing dose levels, including 18 patients at 1500 mg. AZD0171 was well tolerated in all 41 treated patients including the 18 patients receiving 1500 mg Q3W. There were no DLTs or discontinuations due to AZD0171-related AEs. AZD0171-related AEs, as assessed by the investigator, were reported in 46.3% of patients, the majority being Grade 1, and the most common being fatigue (19.5%) and nausea (9.8%). Grade 1 AZD0171-related AEs (increased AST, increased ALT), Grade 2 AZD0171-related AE (GGT increase) and Grade 3 AZD0171-related AE (increased AST) were reported. There were no reports of Grade 4 or 5 AZD0171-related events. There was one case of AZD0171-related SAE reported; Grade 2 osteonecrosis of the mandible, in a head and neck cancer patient who had previously received high dose radiation to the area and had a history of RANKL-inhibitor therapy (denosumab) and active periodical disease (Borazanci et al, 2022).

For information on all identified and potential risks with oleclumab, monalizumab, Dato-DXd, and AZD0171 refer to the respective IBs. For mitigation strategies, please refer to specific TMGs and dose modification information (Section 8.3.15).

2.4.2.3 Chemotherapy Plus Combination Therapy with Durvalumab Plus Oleclumab, Monalizumab, Dato-DXd, or AZD0171; or Chemotherapy Plus Volrustomig or Rilvegostomig

In regard to safety, durvalumab plus carboplatin and paclitaxel, durvalumab plus cisplatin and pemetrexed and durvalumab plus carboplatin and pemetrexed are being evaluated in the AEGEAN study (Section 2.3).

The safety and tolerability of oleclumab in combination with different chemotherapy agents with or without durvalumab is currently being evaluated in 2 clinical studies. Study D933IC00001 (MAGELLAN) is an ongoing Phase IB/II trial to determine the efficacy, safety, PK, and immunogenicity of durvalumab in combination with novel oncology therapies, with

or without chemotherapy, for first-line stage IV NSCLC. As of the DCO of 27 Jan 2020, [redacted] patients received the combination of durvalumab and oleclumab. [redacted] patients [redacted]%) in Cohort [redacted] (durvalumab + oleclumab [redacted] mg) experienced at least one AE. The AEs reported in Cohort [redacted] were pneumonia, cancer pain, hypothyroidism, decreased appetite, steroid diabetes, irritability, hydrothorax, productive cough, constipation, nausea, peripheral oedema, blood pressure increased ([redacted], [redacted]%, each), and [redacted] [redacted]%) death. [redacted] patients [redacted]%) in Cohort [redacted] (durvalumab + oleclumab [redacted] mg + chemotherapy) experienced at least 1 AE. The most frequently reported AEs (≥ 10% of total patients) were nausea [redacted]%), decreased appetite [redacted]%), anaemia [redacted]%), neutropenia and vomiting [redacted]% each), pneumonia [redacted]%), hypokalaemia, dyspnoea, cough, constipation, rash, pruritus, and ALT increased [redacted]% each). Study D6185C00001 is a Phase II, open-label, multi-drug, biomarker-directed, umbrella study that is modular in design to assess the efficacy, safety, and tolerability of multiple treatment arms in patients who have received an anti-PD-1/PD-L1 containing therapy and a platinum- doublet regimen for locally advanced or metastatic NSCLC either separately or in combination. The oleclumab-containing arms in the study consists of 3 arms in which durvalumab [redacted] mg [redacted] and oleclumab [redacted] mg [redacted] × [redacted] and then [redacted]) combinations were explored: [redacted] patients were dosed in Cohort [redacted] (durvalumab in combination with oleclumab in patients with high expression of CD73). There were [redacted] patients dosed in Cohort [redacted] (durvalumab in combination with oleclumab, stratified by prior response to immunotherapy; primary resistance cohort [PRI]) as of the DCO date of 26 January 2020. There were [redacted] patients dosed enrolled in the Cohort [redacted] (durvalumab in combination with oleclumab, stratified by prior response to immunotherapy; acquired resistance cohort [redacted]. As of the DCO of 26 January 2020, a total of [redacted] patients were dosed across [redacted] cohorts, [redacted] patients [redacted]%) had at least 1 AE, [redacted] patients [redacted]%) in Cohort [redacted] patients [redacted]%) in Cohort [redacted] and [redacted] patients [redacted]%) in Cohort [redacted] The most frequently reported AEs (in ≥ 10 % of total patients) were fatigue [redacted]%), decreased appetite and pruritis [redacted]%), anaemia and nausea [redacted]%), dyspnoea, diarrhoea, and asthenia [redacted]%). Overall, [redacted] patients experienced AEs causally related to durvalumab and oleclumab, of which hypertension, pneumonitis, asthenia, and increased blood creatine phosphokinase [redacted] patient each) and dyspnoea [redacted] patients) were Grade 3 or higher AEs. Overall, [redacted] patients [redacted]%) causally related to oleclumab only which were anaemia, decreased appetite, diarrhoea, pruritis, and weight decreased of which anaemia in [redacted] patient [redacted]%) in Cohort [redacted] was Grade 3 or higher.

[redacted] patients had SAEs. [redacted] patients in Cohort [redacted] had SAEs of essential tremor (Grade 1, dose withdrawn/interrupted), blood creatine phosphatase increased (Grade 4, dose withdrawn, causally related to durvalumab and oleclumab), brain oedema [redacted] patients, Grade 3 dose interrupted, and dose withdrawn, respectively), pain (Grade 3), decreased appetite (Grade 3), hypoxia (Grade 3), aspiration pleural effusion [redacted] patients, Grade 3). [redacted] patients in Cohort [redacted] had SAEs of pain in extremity (Grade 2), anaemia (Grade 3, dose withdrawn, causally related to oleclumab), and general physical health deterioration (Grade 3, dose

interrupted). [redacted] patients in [redacted] had SAEs of chronic obstructive pulmonary disease (Grade 3), cardiac failure and pleural effusion (each Grade 2), infectious pleural effusion (Grade 3), confusional state (Grade 2), lethargy (Grade 2), and pleural effusion (Grade 3), splenic infarction (Grade 3), and pneumonitis (Grade 3, drug withdrawn, causally related to durvalumab and oleclumab). Overall, [redacted] patients died in the durvalumab and oleclumab module, [redacted]%) in Cohort [redacted] and [redacted]%) in Cohort [redacted]. Most deaths were due to disease under investigation, followed by "other" deaths. One death in the [redacted] cohort was due to an AE, which was not considered related to any treatment.

Refer to the IB for safety information of oleclumab with other chemotherapy agents in other indications.

Monalizumab at [redacted] mg/kg [redacted] or [redacted] mg in combination with cetuximab, or monalizumab [redacted] mg [redacted] in combination with durvalumab and cetuximab was evaluated in [redacted] patients in a Phase II study (Refer to Monalizumab IB 2020). The most frequently reported AEs (≥ 30% patients in the total group) were dermatitis acneiform in [redacted] ([redacted]%) patients, fatigue in [redacted] [redacted]%) patients, diarrhoea in [redacted] [redacted]%) patients, hypomagnesemia in [redacted] [redacted]%) patients, nausea in [redacted] [redacted]%) patients, and paronychia and pruritus in [redacted] ([redacted]%) patients each. Grade 3 to 4 AEs were reported in [redacted] [redacted]%) patients. Those reported in more than one patient include anaemia, dermatitis acneiform, hypophosphatemia, sepsis ([redacted] [[redacted]%] patients each), hypertension [redacted] [[redacted]%] patients), hypokalaemia, lipase increased, tumour haemorrhage [redacted] [[redacted]%] patients each), amylase increased, dyspnoea, malnutrition, weight decreased [redacted] [[redacted]%] patients each), decreased appetite, diarrhoea, dysphagia, hypoalbuminemia, lymphopenia, pulmonary embolism, and vomiting [redacted]%] patients each). In Study D419NC00001 dose-exploration part of the study, Cohort [redacted] patients were exposed to combination treatment of monalizumab [redacted] mg [redacted] durvalumab [redacted] mg [redacted] and mFOLFOX6 [redacted] with either bevacizumab [redacted] mg/kg [redacted] patients) or cetuximab [redacted] mg/m^2 [redacted]; [redacted] patients). The most frequent AEs (> 30% of patients in the total group) were neuropathy peripheral [redacted]%] patients), fatigue and nausea [redacted]%] patients each), diarrhoea and neutropenia [redacted]%] patients each), lipase increased [redacted]%] patients), decreased appetite and rash [redacted]%] patients each), and amylase increased and pyrexia [redacted]%] patients each). Grade 3 to 4 AEs reported in more than 10% patients in the total group include lipase increased [redacted]%] patients), neutropenia [redacted]%] patients), neuropathy peripheral [redacted]%] patients), and amylase increased, neutrophil count decreased and rash [redacted]%] patients each).

Monalizumab has not been explored in combination with cisplatin or carboplatin-based therapy.

Volrustomig monotherapy is being evaluated in a Phase Ib, open-label, multi-centre study to determine the efficacy and safety in first-line stage IV NSCLC (D933IC00001 [MAGELLAN; NCT03819465]), in addition to the first time in human study in advanced solid tumours

(NCT03530397).

The safety and tolerability of volrustomig in combination with chemotherapy in 1L advanced NSCLC is currently being evaluated in an ongoing FTIH study D7980C00001 (NCT03530397) at doses of volrustomig [redacted] mg, [redacted] mg and [redacted] mg. As of 10 April 2022, [redacted] patients received volrustomig [redacted] mg) in combination with carboplatin and pemetrexed [redacted] in the NSCLC cohort. With a median duration of exposure of [redacted] months, [redacted]% of patients had at least one AE, with the most frequently reported being anaemia [redacted]%), nausea [redacted]%), constipation [redacted]%), and neutropenia [redacted]%). In the same cohort [redacted]% of patients had at least one AE considered to be volrustomig-related, with the most frequent being nausea [redacted]%), asthenia [redacted]%), pruritus [redacted]%), and rash [redacted]%). [redacted] patients [redacted]%) experienced at least one grade 3 or 4 AE with the most frequently reported being neutropenia [redacted]%), dyspnoea [redacted]%), and pneumonia [redacted]%). Serious adverse events occurred in [redacted]% of patients, with the most frequently reported being sepsis [redacted]%), febrile neutropenia [redacted]%), pneumonia [redacted]%), pneumothorax [redacted]%), and urinary tract infection [redacted]%). [redacted] patients [redacted]%) in this combination therapy population experienced AEs leading to the permanent discontinuation of treatment with volrustomig.

Updated safety data as of a DCO of 17 October 2022 in the volrustomig [redacted] mg plus platinum doublet chemotherapy first-line nonsquamous NSCLC cohort showed Grade 3 or 4 TEAEs and AEs leading to treatment discontinuation of [redacted]% and [redacted]%, respectively, similar to what was observed with pembrolizumab and chemotherapy in KN189 (70.1% and 28.9%, respectively) (Gandhi et al, 2018). There were [redacted] treatment-related deaths [redacted]%), all related to chemotherapy toxicities and none related to volrustomig-related immune mediated AEs.

The safety and tolerability of Dato-DXd ([redacted] mg/kg) in combination with durvalumab [redacted] mg/kg [redacted]) with or without carboplatin is currently being evaluated in a Phase Ib study in patients with advanced or metastatic NSCLC (TROPION-Lung04). As of 28 September 2022, [redacted] patients had received treatment with Dato-DXd + durvalumab with [redacted] of the patients also receiving carboplatin. In total, [redacted]%) patients reported at least one TEAE; the majority were non-serious, mild or moderate in severity (CTCAE Grade 1 or 2) and did not lead to treatment withdrawal. In the cohort of patients receiving Dato-DXd + durvalumab + carboplatin, [redacted] [redacted]%) patients had at least one TEAE of CTCAE Grade ≥3 which was causally related to treatment. In the same cohort, serious TEAEs occurred in [redacted] [redacted]%) patients and [redacted] patients [redacted]%) experienced a TEAE associated with treatment withdrawal. The most frequently reported TEAEs in the Dato-DXd + durvalumab + carboplatin cohort were nausea [redacted]%), alopecia [redacted]%), anaemia [redacted]%), [redacted]%), fatigue [redacted]%), oral candidiasis [redacted]%), platelet count decreased [redacted]%), decreased appetite [redacted]%), and neutropenia [redacted]%). The preliminary aggregate safety data from this study suggest that Dato DXd in combination with durvalumab, with and without carboplatin, has a manageable safety and tolerability profile, and support the selection of the

Dato-DXd [redacted] mg/kg dose in combination with established doses of carboplatin and durvalumab in future clinical studies.

For information on all identified and potential risks with Dato-DXd refer to the current version of the IB. For mitigation strategies, please refer to specific TMGs and dose modification information (Section 8.3.15).

To complement the safety data from the Phase I monotherapy study (MSC-1-101; NCT03490669), a single arm Phase II study evaluating the safety and clinical activity of the combination of AZD0171 with durvalumab and standard-of-care chemotherapy for the treatment of 1L mPDAC is currently ongoing. Emerging data from a safety review of [redacted] patients enrolled in the Phase II mPDAC study shows the most common AEs were ALT/AST elevation [redacted]%), nausea [redacted]%), skin rash [redacted]%), anaemia [redacted]%), vomiting [redacted]%), asthenia [redacted]%), alopecia [redacted]%), diarrhea [redacted]%), and IRR [redacted]%), which are consistent with recent findings from other 1L mPDAC studies (Padrón et al, 2022, Tempero et al, 2021). The most common Grade 3 or 4 TEAEs were asthenia ([redacted]%), ALT increase ([redacted]%), rash maculopapular ([redacted]%), and neutropenia ([redacted]%). Out of the [redacted] patients currently enrolled, [redacted] had PD, [redacted] [redacted] died due to disease progression and [redacted] died due to gastroenteritis possibly related to all 4 study interventions (AZD0171, durvalumab, gemcitabine, and nab-paclitaxel) and accompanying sepsis.

The safety of AZD0171 in combination with durvalumab and standard-of-care chemotherapy is currently being evaluated in a Phase II study; emerging data from this study are provided in Section 2.4.2.2.

The safety of rilvegostomig has been evaluated in ARTEMIDE-01 (NCT04995523), a Phase I/II, FTIH study in participants with advanced or metastatic, PD-L1 positive (TPS ≥ 1%) NSCLC who were either previously treated with one CPI and chemotherapy (Parts A and B), or who were CPI-naïve (parts C and D).

As of the DCO of 07 July 2024, [redacted] participants were treated at doses ranging from [redacted] mg to [redacted] mg [redacted] across the study, including [redacted] CPI-experienced and [redacted] CPI-naïve participants. No DLTs were observed during dose escalation, and all dose levels were assessed to be safe and tolerable. Overall, rilvegostomig was well tolerated with [redacted] ([redacted]%) treatment-related discontinuations, [redacted] ([redacted]%) Grade ≥ 3 TRAEs, and [redacted]%) Grade ≥ 3 imAEs. No fatal events related to rilvegostomig were reported. There were no evident differences in the safety profile between the CPI-experienced and CPI-naïve patients, with treatment-related discontinuation reported in [redacted] ([redacted]%) and [redacted] ([redacted]%) patients and treatment-related Grade ≥ 3 AEs in [redacted] ([redacted]%) and [redacted] ([redacted]%) patients, respectively.

The safety of rilvegostomig has also been evaluated in combination with chemotherapy in MAGELLAN (D933IC00001), a Phase IB, open-label, multi-centre study to determine the

efficacy and safety of durvalumab and/or novel oncology therapies, with or without chemotherapy, for 1L stage IV NSCLC. Data from the final analysis are provided in Appendix P 4.

Rilvegostomig is also being evaluated in combination with Dato-DXd in the ongoing Phase IB TROPION-Lung04 study, enrolling subjects with advanced or metastatic NSCLC without actionable genomic alterations. As of the 05 March 2024 DCO, preliminary safety data (n = [redacted] from Cohort [redacted] (Dato-DXd [redacted] mg/kg + rilvegostomig [redacted] mg) showed the observed events were in-line with the known safety profile of individual agents (ie, Dato-DXd and rilvegostomig). Most TEAEs were Grade 1 or 2. Grade 3 TEAEs were reported for [redacted] participants, one of which was related to study treatment. Three SAEs were reported (breast cancer, COPD exacerbation, and inguinal hernia, in [redacted] each), none of which were related to study treatment. No cases of Grade 5 TEAEs were reported. Additional details on the emerging safety data from TROPION-Lung04 are provided in Appendix Q 4.

2.4.3 Overall Benefit: Risk Conclusion

Clinical and/or nonclinical data to date have shown acceptable safety profiles and antitumour activity for oleclumab 3000 mg, monalizumab 1500 mg, volrustomig [redacted] mg, volrustomig [redacted] mg, volrustomig [redacted] mg, Dato-DXd 6 mg/kg, AZD0171 [redacted] mg, and rilvegostomig [redacted] mg proposed in this study. Neoadjuvant durvalumab in combination with oleclumab, monalizumab or AZD0171 and platinum doublet chemotherapy, or volrustomig or rilvegostomig and platinum doublet chemotherapy, or Dato-DXd in combination with durvalumab or rilvegostomig and single agent platinum chemotherapy may provide clinical benefit by enhancing the pCR response and reducing disease recurrence after surgery.

The design of the current study aims to minimise potential risks to patients, including the inclusion and exclusion criteria (Section 5.1 and Section 5.2), restrictions on concomitant medications during the study (Appendix H), safety monitoring (including review of all relevant data by the SRC, Section 4.1]), TMGs (Section 8.3.15), stopping criteria (Section 7.1), and rules and procedures to add new combination therapy arms. Specific intensive safety monitoring is in place for those risks deemed to be most likely for each of the combination therapies.

Further design elements aim to specifically minimise the risks to patients enrolled in this study. Any new combination treatment arm must adhere to the following elements:

- A rationale for additive or synergistic activity of the potential new combination candidate agent(s) based on its mechanism of action and supported by nonclinical or clinical evidence.
- An established, recommended combination dose for the candidate agent(s) with an acceptable safety profile for the target population in this study.

- Description of the safety profile and AESIs for the candidate agent(s) as monotherapy and/or in combination with other agents based on previous Phase I expansion cohorts or Phase II studies.
- Preliminary evidence of clinical activity of the candidate agent(s) as monotherapy or in combination in a solid tumour setting.
- Requirement of a protocol amendment and respective health authority and local IRB/IEC approvals prior to implementing any new treatment arm.
- Updated informed consent form with relevant information on the new combination therapy arm.

Thus, the benefit-risk assessment for this Phase II study is acceptable.

3 OBJECTIVES AND ENDPOINTS

Table 3 Objectives and Endpoints

Objectives	Estimand description/Endpoints
Primary	
To assess the antitumour activity of neoadjuvant treatment administered prior to surgery in terms of pCR.	pCR is defined as lack of any viable tumour cells after complete evaluation in the resected lung cancer specimen and all sampled regional lymph nodes as determined by central BIPR and described by IASLC 2020 (Travis et al, 2020).<br>The measure of interest is the proportion of patients with 0% residual viable tumour cells within all resected tissue as assessed by the central blinded pathologist.
To assess the safety and tolerability of neoadjuvant and adjuvant treatment.	Safety and tolerability will be evaluated in terms of AEs, vital signs, and clinical laboratory parameters.
Secondary	
To assess the efficacy of neoadjuvant treatment administered prior to surgery followed by adjuvant treatment post-surgery in terms of EFS.	EFS is defined as time from randomisation to the first of the following:<br>• Documented local or distant recurrence as determined by Investigator using RECIST 1.1 assessment.<br>• Death due to any cause (event date is date of death).<br>• PD that precludes surgery (event date is the date of this determination) or PD discovered and reported by the Investigator upon attempting surgery that prevents completion of surgery (event date is the date of the first attempt at surgery).<br>A new primary malignancy confirmed by pathology is not considered an EFS event.<br>The measure of interest is the median of EFS and landmark EFS at 12 and 24 months.
To assess the efficacy of neoadjuvant treatment administered prior to surgery followed by adjuvant treatment post-surgery in terms of DFS (event from surgery onwards).	DFS is defined as the time from the date of surgery until the first date of disease recurrence as determined by Investigator using RECIST 1.1 assessment (local or distant), or date of death due to any cause, whichever occurs first. Pathological confirmation from biopsied lesions will also be

Objectives	Estimand description/Endpoints
	taken into consideration (as applicable). A new primary malignancy confirmed by pathology is not considered a DFS event.<br>The measure of interest is the median of DFS and landmark DFS at 12 and 24 months.
To assess the feasibility of receiving the planned surgical tumour resection in patients receiving neoadjuvant treatment.	Feasibility to surgery is defined as having the planned surgical resection within 40 days from the end of the last dose of neoadjuvant study interventions.<br>The measure of interest is the proportion of patients that have intended surgery within 40 days from the end of last dose of neoadjuvant study interventions.
To assess the antitumour activity of neoadjuvant treatment administered prior to surgery in terms of mPR.	mPR is defined as ≤ 10% viable tumour cells in resected tumour after complete evaluation in the resected lung cancer specimen as determined by central BIPR as described by IASLC 2020 (Travis et al, 2020).<br>The measure of interest is the proportion of patients with ≤ 10% residual viable tumour cells within all resected tissue as assessed by the central blinded pathologist.
To assess the efficacy of neoadjuvant treatment administered prior to surgery in terms of ORR.	ORR is defined as the proportion of patients who have a CR or PR as determined by Investigator using RECIST 1.1.<br>Data obtained from randomisation up until surgery, or the last evaluable assessment in the absence of progression, prior to surgery, will be included in the assessment of ORR, regardless of whether the patient withdraws therapy. Patients who go off therapy prior to surgery, without a response, receive a subsequent therapy prior to surgery, and then respond will not be included as responders in the ORR.<br>The measure of interest is the proportion of patients with OR.
To assess the efficacy of neoadjuvant and adjuvant treatment in terms of OS.	OS is defined as the time from randomisation until the date of death due to any cause.<br>The measure of interest is the landmark OS at 12 months and 24 months, and other clinically relevant timepoints if feasible. If reached by the end of the study, the median OS will also be of interest.

**Objectives**	**Estimand description/Endpoints**
To describe the PK of study interventions in patients receiving neoadjuvant/adjuvant treatment	Concentration of study interventions in plasma or serum.
To assess the immunogenicity of study interventions in patients receiving neoadjuvant/adjuvant treatment.	Presence of ADA for study interventions.
To investigate baseline PD-L1 expression in patients treated with neoadjuvant and adjuvant treatment, and associations with clinical endpoints.	Baseline PD-L1 expression.
To evaluate changes in ctDNA during neoadjuvant treatment in patients with evaluable ctDNA and associations with clinical endpoints.	ctDNA clearance on-treatment prior to surgery.
Tertiary/Exploratory	
To evaluate baseline and/or on-treatment changes in ctDNA, blood-based mRNA expression signatures, circulating protein levels, and/or immune cell composition and repertoire in patients receiving neoadjuvant and adjuvant treatment, and associations with clinical endpoints.<br>To evaluate baseline and on-treatment changes by flow cytometry in patients receiving volrustomig and rilvegostomig, and associations with clinical endpoints.	ctDNA: May include, but not limited to, baseline allelic fractions, changes in levels on-treatment prior to surgery and following surgery, and association with outcomes.<br>Blood mRNA expression: May include, but not limited to, immune-relevant gene signatures at baseline, changes in levels on-treatment, and association with outcomes.<br>Circulating soluble factors from plasma and serum: May include, but not limited to, circulating levels of target proteins, cytokines, chemokines, and other soluble factors at baseline, changes in levels on-treatment and association with outcomes.<br>PBMC: May include, but not limited to, immune cell composition, phenotypes, and/or B/T cell repertoire at baseline, changes in levels on-treatment, and association with outcomes.<br>Flow cytometry: May include, but are not limited to, changes in immune cell populations, target occupancy, or immune cell phenotypes and association with outcomes.
To investigate baseline and/or on treatment and/or post-surgery changes in gene expression, DNA, and/or proteins in tumour tissue from patients with neoadjuvant and adjuvant treatment, and associations with clinical endpoints.	Biomarker expression, may include but not limited to, *PD-L1*, *CD73*, *NKG2A*, *HLA-E*, LIF, TROP2, SLFN11, and *CD8* expression, gene expression, mutations, TMB and/or proteogenomic profiling, in primary tumour and draining lymph nodes.

Abbreviations: ADA: Anti-drug antibody; AEs: Adverse events; BIPR: Blinded independent pathologist review; CD: Cluster of differentiation; CR: Complete response; CTCAE: Common Terminology Criteria for Adverse Event; ctDNA: Circulating tumour DNA; DFS: Disease-free survival; EFS: Event-free survival; IASLC: International Association for the Study of Lung Cancer; LIF: Leukaemia inhibitory factor; mRNA: Messenger ribonucleic acid; MPR: Major pathological response; OS: Overall survival; ORR: Objective response rate; PBMC: Peripheral blood mononuclear cells; pCR: Pathological complete response; PD: Progression of disease; PD-L1: Programmed cell death ligand-1; PK: Pharmacokinetics; PR: Partial response; RECIST: Response Evaluation Criteria in Solid Tumours; SLFN11: Schlafen 11; TROP2: Human trophoblast cell-surface antigen 2; TMB: Tumour mutational burden.

4 STUDY DESIGN

4.1 Overall Design

Study D9077C00001 (NeoCOAST-2) is a Phase II, open-label, multi-arm, multicentre, randomised, neoadjuvant and adjuvant study for the treatment of patients with resectable early-stage NSCLC (Stage II to IIIB, according to Version 8 of the IASLC Staging Manual in Thoracic Oncology 2016).

Up to 630 patients will be enrolled and randomised to one of the following treatment regimens:

Table 4 Treatment Regimens

	Neoadjuvant treatment/Pre-Surgery (4 cycles)	Adjuvant treatment/Post-Surgery (1 year)
**Arm 1** (n=up to 70)	Oleclumab + durvalumab + CTX (Q3W)	Oleclumab + durvalumab (Q4W) x 12 cycles
**Arm 2** (n=up to 70)	Monalizumab + durvalumab + CTX (Q3W)	Monalizumab + durvalumab (Q4W) x 12 cycles
**Arm 3A** [a] (n=up to 70)	Volrustomig [redacted] mg + CTX [redacted] ([redacted] cycles)	Volrustomig [redacted] mg [redacted]) x [redacted] cycles
**Arm 3B** (n=up to 70)	Volrustomig + CTX [redacted]<br>[redacted]	Volrustomig [redacted] mg [redacted] x [redacted] cycles
**Arm 3C** (n=up to 70)	Volrustomig + CTX [redacted]<br>[redacted]	Volrustomig [redacted] mg [redacted] x [redacted] cycles
**Arm 4** (n=up to 70)	Dato-DXd + durvalumab + single agent platinum chemotherapy (Q3W)	Durvalumab (Q4W) x 12 cycles
**Arm 5** (n=up to 70)	AZD0171 + durvalumab + CTX [redacted]	AZD0171 [redacted] + Durvalumab [redacted] x [redacted] cycles
**Arm 6** (n=up to 70)	Rilvegostomig + CTX [redacted]	Rilvegostomig [redacted] x [redacted] cycles
**Arm 7** (n=up to 70)	Dato-DXd + Rilvegostomig + single agent platinum chemotherapy [redacted]	Rilvegostomig [redacted] x [redacted] cycles

Note: Treatment regimens may be given unless there is unacceptable toxicity, withdrawal of consent, or another discontinuation criterion is met (see Figure 1).
Note: Refer to Section 6.1.1 for details on study intervention and chemotherapy regimens.
Abbreviations: CTX: platinum doublet chemotherapy; Dato-DXd: datopotamab deruxtecan; Q2W: every 2 weeks; Q3W: every 3 weeks; Q4W: every 4 weeks.
[a] Recruitment closed at [redacted] patients; no additional patients will be enrolled in Arm 3A.

Patients in Arms 1 to 5 will be stratified by baseline PD-L1 expression status (< 1% versus ≥ 1%). Patients in Arms 6 and 7 will be restricted to baseline PD-L1 expression status ≥ 1%

and stratified (1% to 49% versus ≥ 50%).

In the neoadjuvant period, patients will receive 4 cycles of treatment followed by surgical resection. Surgery is to be performed within 40 days from the last dose of study interventions. Every attempt should be made to ensure surgery is performed within 40 days from the last dose of neoadjuvant treatment. Cases where surgery cannot be completed within 40 days from the last dose of study interventions should be promptly reported to the Sponsor and discussed with the Study Physician/Medical Scientist. Surgery may consist of lobectomy, sleeve resection, or bilobectomy, as determined by the attending surgeon based on the baseline findings.

After surgery, patients should be able to start adjuvant treatment as soon as clinically feasible and within 10 weeks from surgery (except for patients receiving PORT, which must be started within 8 weeks after surgery. Adjuvant treatment must be started within 3 weeks from the end of PORT). Post-surgery, patients will receive additional cycles of treatment equivalent to 1 year.

An early safety evaluation will be done by an SRC to review all available data when the first [redacted] patients in each enrolling treatment arm have received [redacted] cycles of neoadjuvant treatment. An additional review of safety data by the SRC will occur when the first [redacted] patients in each enrolling treatment arm have undergone surgery and have had [redacted] days of follow-up, in order to assess perioperative mortality and surgery delays.

Additionally, throughout the study if more than [redacted]% of patients experience a delay in surgery of more than [redacted] weeks or become unable to receive surgery due to toxicity on any treatment arm, enrolment in that arm will be halted and SRC members will be notified to review these cases and provide their recommendation to the Sponsor.

The SRC will meet regularly at approximately [redacted] month intervals to review the safety and tolerability of treatment as neoadjuvant and adjuvant regimen, until all patients have had the opportunity to undergo surgery and those having surgery have had at least [redacted] months of adjuvant treatment.

Reasons for delay to surgery which will not trigger enrolment hold are:

- Weather or other local emergencies impacting normal hospital activities.
- Unforeseen circumstances not related to the disease under study, such as, but not limited to, car accident, surgeon schedule, family illness or unexpected travel.
- Withdrawal of consent to further participation on the study not related to AEs or study procedures.

Other treatment arms may be added based on emerging nonclinical and clinical data via a

protocol amendment.

Tumour evaluation using RECIST 1.1 will be conducted at screening (within 28 days prior to randomisation), after 2 cycles and at the completion of the neoadjuvant treatment prior to surgery, and then after surgery Q12W ± 1 week (relative to the date of surgery) until week 48, Q24W ± 2 weeks (relative to the date of surgery) until week 192 (approximately 4 years), and then Q48W ±2 weeks (relative to the date of surgery) thereafter, until RECIST 1.1-defined radiological PD, consent withdrawal, death, or study completion as determined by Sponsor. For patients who do not have surgery and therefore will not have a first post-surgical scan or adjuvant treatment, follow-up scans are acquired Q12W ± 1 week (relative to the date of surgery) until week 48; Q24W ± 2 weeks (relative to the date of surgery) until week 192 (approximately 4 years); and then Q48W ± 2 weeks (relative to the date of surgery) thereafter, until RECIST 1.1-defined radiological PD, consent withdrawal, death, or study completion as determined by Sponsor. This on-study schedule MUST be followed regardless of any delays in dosing and regardless of tumour margins. These follow-up scans will use the original neoadjuvant screening scan as a baseline scan for RECIST 1.1 assessments.

For an overview of the study design, see Figure 1, in Section 1.2. For details on treatments given during the study, see Section 6.1.

For details on what is included in the efficacy and safety endpoints, see Section 8.

In order to provide easier access to relevant information for each treatment arm the protocol is arranged as shown in Figure 2. Information which is only relevant to specific arms of the study are provided in appendices.

Figure 2 Protocol Structure

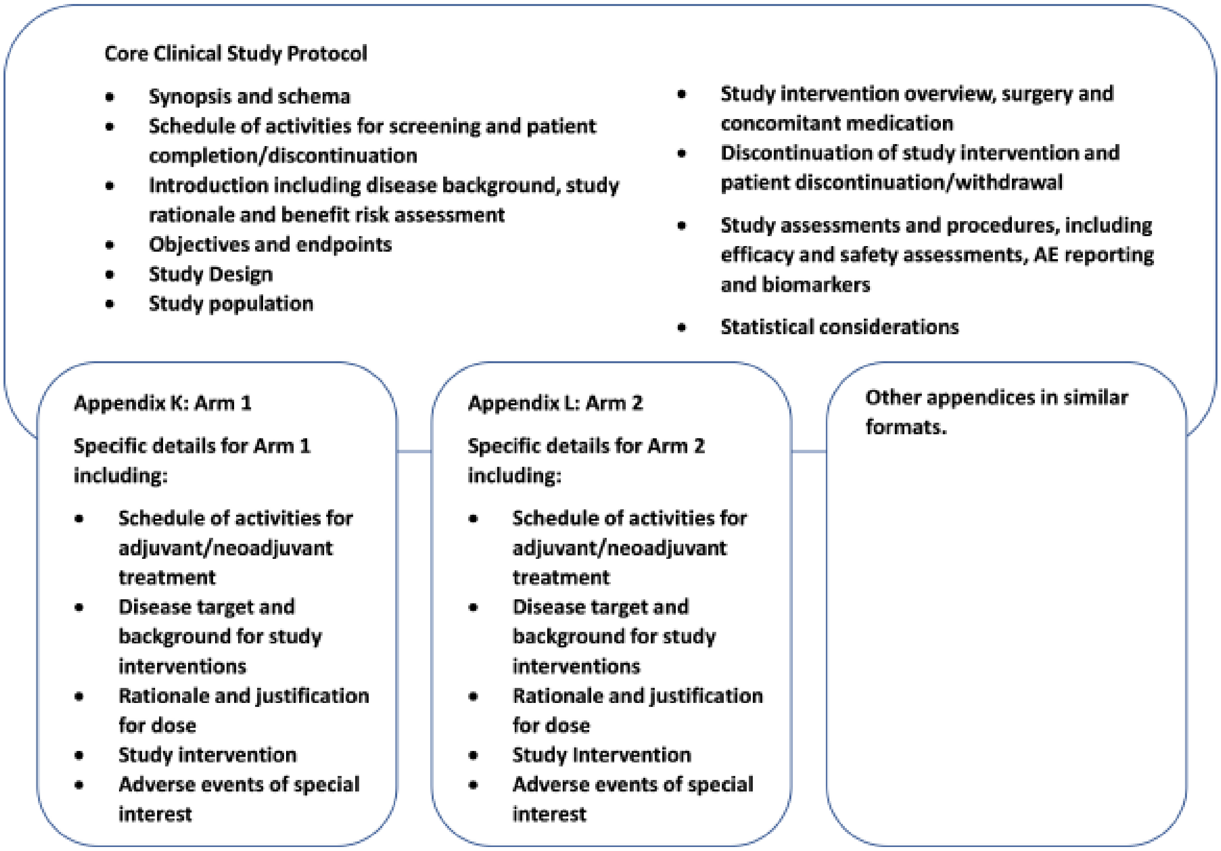

4.1.1 Study Conduct Mitigation During Study Disruptions Due to Cases of Civil Crisis, Natural Disaster, or Public Health Crisis

The guidance given below supersedes instructions provided elsewhere in this CSP and should be implemented only during cases of civil crisis, natural disaster or public health crisis (eg, during quarantines and resulting site closures, regional travel restrictions, and considerations if site personnel or study patients become infected with SARS-CoV-2 or similar pandemic infection), which would prevent the conduct of study-related activities at study sites, thereby compromising the study site staff or the patient's ability to conduct the study. The Investigator or designee should contact the study Sponsor to discuss whether the mitigation plans below should be implemented.

To ensure continuity of the clinical study during a civil crisis, natural disaster, or public health crisis, changes may be implemented to ensure the safety of study patients, maintain compliance with GCP, and minimise risks to study integrity.

Where allowable by local health authorities, ethics committees, HCP guidelines (eg, hospital policies) or local government, these changes may include the following options:

- Obtaining consent/reconsent for the mitigation procedures (note, in the case of verbal consent, the ICF should be signed at the patient's next contact with the study site).
- Rescreening: Additional rescreening for screen failure due to study disruption and to confirm eligibility to participate in the clinical study can be performed in previously screened patients. The Investigator should confirm this with the designated Study Physician/Medical Scientist.
- Home or Remote visit: Performed by a site qualified HCP or HCP provided by a TPV.
- Telemedicine visit: Remote contact with the patients using telecommunications technology including phone calls, virtual or video visits, and mobile health devices.
- At-home study interventions administration: Performed by a site-qualified HCP, by a HCP provided by a TPV, or by the patients or the patient's caregiver, if possible. Additional information related to the visit can be obtained via telemedicine.

For further details on study conduct during civil crisis, natural disaster, or public health crisis, refer to Appendix J.

4.2 Scientific Rationale for Study Design

4.2.1 Rationale for Efficacy Endpoints

The primary efficacy endpoint in this study is pCR.

Pathological complete response is defined as the lack of any viable tumour cells after complete evaluation in the resected lung cancer specimen and all sampled regional lymph

nodes (Travis et al, 2020, Hellmann et al, 2014, Cottrell et al, 2018). The median rate of pCR reported from 15 randomised controlled studies of neoadjuvant chemotherapy of 4% (ranging from 0 to 16%) is lower than mPR; however, there is a strong correlation between pCR and both 5-year survival rate and OS. Therefore, pCR shows promising potential as a surrogate endpoint for OS (Hellmann et al, 2014). Two published, single-arm Phase II studies reported higher rates of pCR following neoadjuvant immunotherapy (atezolizumab, 33%; nivolumab, 63%) in combination with chemotherapy (Provencio et al, 2020, Shu et al, 2020). The CheckMate-816, a Phase III study has shown that blockade of PD-L1 in combination with chemotherapy in the neoadjuvant setting yield statistically significant increase in pCR (24% vs 2.2%) and EFS (31.6 months vs 20.8 months) (Forde et al, 2022) compared to chemotherapy alone in patients with resectable NSCLC without delaying planned surgical resection.

Secondary efficacy endpoints in this study include EFS, DFS, mPR, ORR, and OS.

Major pathological response has been proposed as a potential surrogate endpoint for OS following neoadjuvant chemotherapy in patients with NSCLC because it has been observed that mPR reliably and statistically significantly associates with survival in retrospective and prospective studies in lung cancer (Hellmann et al, 2014). For example, a prospective study of 50 patients with Stages IB to IIIA non-squamous NSCLC treated with neoadjuvant chemotherapy and bevacizumab showed that 27% of resected patients had mPR ($\geq$ 90% treatment effect); 100% of these patients were alive at 3 years compared with only 49% of those who had < 90% treatment effect after resection (Chaft et al, 2013). Similarly, a study of 192 patients with resected Stages I to IV NSCLC given neoadjuvant chemotherapy showed robust improvement in survival in patients with less than 10% viable tumour compared to those patients with more than 10% viable tumour (5-year OS 85% versus 40%, respectively; Pataer et al, 2012).

Event-free survival allows the capture of peri-surgical events, whereas DFS captures only events from surgery onwards, when the patient is disease-free. Disease-free survival represents a direct measure of the study intervention's efficacy as it is not confounded by the efficacy of subsequent therapies used after disease relapse. Moreover, historical data showed that the DFS benefit seen with the use of adjuvant chemotherapy in this disease setting was consistent with an improvement in the OS outcome, which suggests an association between these 2 endpoints in this setting (Mauguen et al, 2013). Disease-free survival has been the primary basis of approval for adjuvant breast cancer hormonal therapy, adjuvant colon cancer, and adjuvant cytotoxic breast cancer therapy.

4.3 Justification for Dose

Justification for the dose of each study intervention used in each treatment arm is provided in the relevant appendix.

4.3.1 Rationale for Duration of Neoadjuvant and Adjuvant Treatment

In the proposed study, durvalumab + either oleclumab, monalizumab or AZD0171 + platinum doublet chemotherapy, or volrustomig + platinum doublet chemotherapy, or Dato-DXd + either durvalumab or rilvegostomig + single agent platinum chemotherapy, or rilvegostomig + platinum doublet chemotherapy is being administered for 4 cycles in the neoadjuvant period in order to have the greatest reduction in tumour volume as measured by pCR. This is in alignment with the NCCN guidelines, which recommends 4 cycles of chemotherapy in the neoadjuvant setting.

Pembrolizumab every 3 weeks was evaluated in NSCLC following complete resection versus placebo for up to 18 administrations (approximately 1 year). Pembrolizumab significantly improved the primary endpoint of DFS from 42 months in the placebo arm to 54 months in the pembrolizumab arm (HR=0.76, p=.00014). The safety profile observed with pembrolizumab was as expected and manageable (Paz-Ares et al, 2022). The one-year treatment duration demonstrated that agents can be administered safely administered for one year with clear benefit.

4.4 End of Study Definition

For the purpose of Clinical Trial Transparency, the definition of the end of the study differs under FDA and EU regulatory requirements:

European Union requirements define study completion as the last visit of the last subject for any protocol related activity.

Food and Drug Administration requirements defines two completion dates:

Primary Completion Date – the date that the final patient is examined or receives an intervention for the purposes of final collection of data for the primary outcome measure, whether the clinical study concluded according to the pre-specified protocol or was terminated. In the case of clinical studies with more than one primary outcome measure with different completion dates, this term refers to the date on which data collection is completed for all of the primary outcomes.

Study Completion Date – the date the final patient is examined or receives an intervention for purposes of final collection of data for the primary and secondary outcome measures and AEs (for example, last patient's last visit), whether the clinical study concludes according to the pre-specified protocol or is terminated.

The end of study is defined as the last expected visit/contact of the last patient completing the study (SoA, Table 2). The sponsor has the option to end enrolment in a treatment arm or the entire study per recommendation of the SRC or business decision.

A patient is considered to have completed the study if they have completed all phases of the study including the last scheduled visit or the last scheduled procedure shown in the SoA (Table 2).

Patients may be withdrawn from the study if the study itself is stopped. The study may be stopped if, in the judgement of AstraZeneca, study patients are placed at undue risk because of clinically significant findings or for regulatory, ethical or other reasons consistent with applicable laws, regulations, and GCP.

In the event that a rollover or safety extension study is available at the time of the final DCO and database closure, patients may be transitioned to such a study, and the current study would reach its end.

The rollover or safety extension study would ensure follow-up with visit assessments per its protocol. Any patient who would be proposed to move to such a study would be given a new ICF.

Arm-specific CSRs may be produced as each of the arms complete. A final integrated CSR will be written containing all analyses.

See Appendix A 6 for guidelines for the dissemination of study results.

See Section 6.7 for details on patient management following the final DCO as well as following study completion.

5 STUDY POPULATION

Prospective approval of protocol deviations to recruitment and enrolment criteria, also known as protocol waivers or exemptions, is not permitted. If a patient was discovered during the study to not meet study trial entry, that is inadvertently enrolled, their continued participation needs to be discussed with AstraZeneca Study Physician/Medical Scientist.

Each patient must meet all of the inclusion criteria and none of the exclusion criteria for this study in order to be assigned/randomised to a study intervention. Under no circumstances can there be exceptions to this rule. Patients who do not meet the entry requirements are screen failures, refer to Section 5.4.

In this protocol, "enrolled" patients are defined as those who sign an informed consent form. "Randomised" patients are defined as those who undergo randomisation and receive a randomisation number.

For procedures for withdrawal of incorrectly enrolled patients, see Section 6.3.

5.1 Inclusion Criteria

Patients are eligible to be included in the study only if all of the following criteria apply:

Informed consent

1 Capable of giving signed informed consent, which includes compliance with the requirements and restrictions listed in the ICF and in this protocol.

2 Provision of signed and dated written ICF prior to any mandatory study specific procedures, sampling, and analyses.

3 Provision of signed and dated written ICF prior to collection of samples for genetic analysis.

The ICF process is described in Appendix A 3.

Age

4 Patients must be ≥ 18 years at the time of screening.

Type of Patient and Disease Characteristics

5 Newly diagnosed and previously untreated patients with histologically or cytologically documented NSCLC. Patients should have resectable (Stage IIA to Stage IIIB) disease (according to Version 8 of IASLC Staging Manual in Thoracic Oncology 2016. Patients with N2 disease are eligible) if they are candidate for lobectomy, sleeve resection, or bilobectomy at the time of screening. Patients with N3 disease are excluded.

- At screening, complete surgical resection of the primary NSCLC must be deemed achievable, as assessed by a multidisciplinary evaluation, which must include a thoracic surgeon who performs lung cancer surgery as a prominent part of his/her practice.
    - T4 tumours will be eligible if they are defined as T4 based on their size (more than 7 cm) or if separate lesions are present in different ipsilateral lobes; any other reason for T4 (eg, adherent to any of the following structures: diaphragm, mediastinum, heart, great vessels, trachea, recurrent laryngeal nerve, oesophagus, vertebral body, carina) will be considered ineligible.
    - Nodal status should be investigated with whole body FDG-PET, plus contrast enhanced CT. If PET/CT scan is positive in the mediastinum, or if scan is negative but there is T > 3 cm, central tumour, or cN1, then it is recommended that nodal status be proven by biopsy via endobronchial ultrasound, mediastinoscopy, or thoracoscopy. See Section 6.1.3 (preoperative mediastinal lymph node staging) for more details.

6 WHO or ECOG performance status of 0 or 1 at enrolment.

7 Adequate organ and marrow function as defined below:

- Haemoglobin ≥ 9.0 g/dL (red blood cell/plasma transfusion is not allowed within one week prior to screening assessment).
- Absolute neutrophil count ≥ 1.5 × 10^9/L.
- Platelet count ≥ 100 × 10^9/L.
- Serum bilirubin ≤ 1.5 × ULN. This will not apply to patients with confirmed Gilbert's syndrome, who will be allowed in consultation with their physician.
- ALT and AST ≤ 3.0 × ULN.
- Measured CrCL > 45 mL/min or calculated CrCL > 45 mL/min as determined by Cockcroft-Gault (using actual body WT) using https://www.kidney.org/professionals/KDOQI/gfr_calculatorCoc
- LVEF ≥ 50% as assessed by echocardiogram or MUGA scan (Note: this criterion only applies if Arms 3A, 3B, or 3C are open for enrolment.)
- Troponin I or T ≤ ULN (per institutional guidelines and/or not clinically significant per investigator judgement).

8 Must have a life expectancy of at least 12 weeks.

Weight

9 Body WT > 35 kg.

Sex

10 Male and/or female.

Females of childbearing potential should agree to use an acceptable method of contraception (see Appendix G) from the time of screening throughout the total duration of the study and for the following period after receiving the last dose of study interventions:

- Durvalumab or volrustomig: 90 days
- Oleclumab, monalizumab, or AZD0171: 180 days
- Dato-DXd: 210 days
- Rilvegostomig: 60 days

For the chemotherapy agents follow the local prescribing information relating to contraception, the time limits for such precautions, and any additional restrictions for the agents administered. For patients receiving more than one study intervention, the longest washout period must be followed after the last dose of study interventions to prevent pregnancy. Female patients must not donate, bank or retrieve for their own use, ova during this same time period. For more details on contraceptive requirements of the study, see Appendix G.

Non-sterilised male partners of a woman of childbearing potential must use a male condom

plus spermicide (condom alone in countries where spermicides are not approved) throughout this period.

Male patients who intend to be sexually active with a female partner of childbearing potential must be surgically sterile or using an acceptable method of contraception (see Appendix G) from the time of screening throughout the total duration of the study and for the following period after the last dose of study interventions:

– Durvalumab or volrustomig: 90 days
– Oleclumab, monalizumab, or AZD0171: 180 days
– Dato-DXd: 120 days
– Rilvegostomig: 60 days

For the chemotherapy agents follow the local prescribing information relating to contraception, the time limits for such precautions, and any additional restrictions for the agents administered. For patients receiving more than one study intervention, the longest washout period must be followed after the last dose of study interventions to prevent pregnancy in a partner. Male patients must not donate or bank sperm during this same time period as for contraception use, see Appendix G. Female partners (of childbearing potential) of male patients must also use a highly effective method of contraception throughout this period.

11 Negative pregnancy test (serum) for women of childbearing potential

Tumour sample requirements:

12 Provision of tumour samples (newly acquired or archival tumour tissue [≤ 6 months old]) to confirm PD-L1 status, EGFR, or ALK status where required during screening and prior to randomisation.

a) PD-L1 status:

(i) Documented PD-L1 expression status from analytically validated, local-regulatory approved test or,

(ii) Provision of tumour for local testing on analytically approved, local regulatory approved assay; SP263 antibody is preferred if available.

Note: Local test results must differentiate between PD-L1 < 1%, 1% to 49%, and ≥ 50%.

b) *ALK* and *EGFR* status:

(i) Previous local laboratory results for ALK and EGFR can be used if performed on well-validated, local regulatory-approved assay, or

(ii) Provision of tumour for local testing on analytically approved, local regulatory approved assay

(iii) Patients with an EGFR mutation, ALK rearrangement or unknown EGFR/ALK status will not be randomised, with the following exceptions:

- Patients with squamous cell carcinoma do not require ALK status.

13 Provision of tumour appropriate for exploratory biomarker analyses. Newly acquired or archival tumour tissue (≤ 6 months old) must be available from core needle biopsy, punch biopsy, excisional biopsy, or surgical specimen. Fine needle aspirate is not acceptable. Core needle biopsies obtained by EBUS are acceptable. Tissue cores must contain cells and stroma. Cytology samples and specimens with limited tumour content are considered inadequate and will not be acceptable. See Section 8.6 and the laboratory manual for details.

Surgery eligibility (assessments performed at screening):

14 Patients will be suitable for inclusion if the planned surgery to be performed will be lobectomy, sleeve resection, or bilobectomy, as determined by the attending surgeon based on the baseline findings.

15 A pre- or post-bronchodilator FEV_1 of 1.0 L and DLCO > 40% postoperative predicted value. Use of these cut-off values to assess candidacy for resection should be guided by the results of cardiopulmonary exercise testing as outlined in the ESMO guidelines on pre-treatment risk assessment. Both an FEV_1 and a DLCO test are required for assessing lung function at screening.

5.2 Exclusion Criteria

Patients are excluded from the study if any of the following criteria apply:

Medical Conditions

1 Patients with:

(a) sensitising *EGFR* mutations or *ALK* translocations

(b) with baseline PD-L1 expression status < 1% (Arms 6 and 7 only)

(i) Note: PD-L1 expression status < 1% only applies as an exclusion when Arms 6 or 7 are open to enrolment

2 History of allogeneic organ transplantation.

3 Active or prior documented autoimmune or inflammatory disorders (including inflammatory bowel disease [eg, colitis or Crohn's disease], diverticulitis [with the exception of diverticulosis], systemic lupus erythematosus, sarcoidosis, granulomatosis with polyangiitis, Graves' disease, rheumatoid arthritis, hypophysitis, uveitis, autoimmune pneumonitis or autoimmune myocarditis). The following are exceptions to this criterion:

- Patients with vitiligo or alopecia.
- Patients with hypothyroidism (eg, following Hashimoto syndrome) stable on hormone replacement.
- Any chronic skin condition that does not require systemic therapy.
- Patients without active disease in the last 5 years may be included but only after consultation with the Study Physician/Medical Scientist.
- Patients with celiac disease controlled by diet alone.

4 Uncontrolled intercurrent illness, including but not limited to, uncontrolled hypertension, unstable angina pectoris, uncontrolled cardiac arrhythmia, active bleeding diseases, serious chronic gastrointestinal conditions associated with diarrhoea, or psychiatric illness/social situations that would limit compliance with study requirement, substantially increase risk of incurring AEs, or compromise the ability of the patient to give written informed consent.

5 History of another primary malignancy, except for the following:

- Malignancy treated with curative intent and with no known active disease ≥ 3 years before the first dose of study interventions and of low potential risk for recurrence.
- Adequately treated non-melanoma skin cancer or lentigo maligna without evidence of disease.
- Adequately treated carcinoma in-situ without evidence of disease.

6 Patients with small-cell lung cancer or mixed small-cell lung cancer.

7 History of active primary immunodeficiency.

8 History of non-infectious ILD/pneumonitis that required steroids, has current ILD/pneumonitis, or has suspected ILD/pneumonitis that cannot be ruled out by imaging at screening.

9 Evidence of the following infections:

(a) Active infection including tuberculosis (clinical evaluation that includes clinical history, physical examination, and radiographic findings and tuberculosis testing in line with local practice).

(b) Known HIV infection that is not well controlled. All of the following criteria are required to define an HIV infection that is well controlled: undetectable viral RNA, CD4+ count ≥ 350, no history of acquired immune deficiency syndrome-defining opportunistic infection within the past 12 months, and stable for at least 4 weeks on the same anti-HIV medications (meaning there are no expected further changes in that time to the number or type of antiretroviral drugs in the regimen). If an HIV infection meets the above criteria, monitoring of viral RNA load and CD4+ count is recommended. If Arms 4, 6 or 7 are open for enrolment, all participants must be

tested for HIV during the screening period if acceptable by local regulations or an IRB/EC.

(c) Active or uncontrolled HBV or HCV. Patients are eligible if they:

- Have controlled hepatitis C viral load defined as undetectable hepatitis C RNA by PCR either spontaneously or in response to a successful prior course of anti-hepatitis C therapy.
- Have received HBV vaccination with only anti-HBs positivity and no clinical signs of hepatitis.
- Are HBsAg- and anti-HBc+ (ie, those who have cleared HBV after infection) and meet conditions i-iii below:
- Are HBsAg+ with chronic HBV infection (lasting 6 months or longer) and meet conditions i-iii below:
  (i) HBV DNA viral load <100 IU/mL.
  (ii) Have normal transaminase values.
  (iii) Start or maintain antiviral treatment if clinically indicated as per the investigator.

(d) Patients with active hepatitis A.

10 Patients who have preoperative radiotherapy treatment as part of their care plan.

11 Patients who require or may require pneumonectomy, segmentectomies, or wedge resections, as assessed by their surgeon at baseline, to obtain potentially curative resection of primary tumour.

12 QTcF interval ≥ 470 ms (NOTE: If prolonged, then 2 additional ECGs should be obtained and the average QTcF interval should be used to determine eligibility).

13 Known allergy or hypersensitivity to any of the study interventions or any of the study intervention excipients or history of severe hypersensitivity reactions to other monoclonal antibodies.

14 Any medical contraindication to treatment with chemotherapy as listed in the local labelling.

15 Patients with moderate or severe cardiovascular disease:

- Presence of cardiac disease, including myocardial infarction or any other arterial thrombotic event including cerebrovascular accident, transient ischemic attack, or unstable angina pectoris within 6 months prior to study enrolment.
- NYHA Class 3 or 4 congestive heart failure, or uncontrolled hypertension.

16 Patients with clinically significant corneal disease (Note that this exclusion criterion only applies if Arms 4 or 7 are open for enrolment).

Prior/Concomitant Therapy

17 Any concurrent chemotherapy, investigational product, biologic, or hormonal therapy for cancer treatment. Concurrent use of hormonal therapy for non-cancer-related conditions (eg, hormone replacement therapy) is acceptable.

18 Receipt of live attenuated vaccine within 30 days prior to the first dose of study interventions. Note: Patients, if enrolled, should not receive live vaccine while receiving study interventions and up to 30 days after the last dose of study interventions.

19 Major surgical procedure (as defined by the Investigator) including highly invasive dental procedures within 30 days prior to the first dose of study interventions (Note that the exclusion of highly invasive dental procedures only applies if Arm 5 is open for enrolment).

20 Prior exposure to approved or investigational immune-mediated therapy including, but not limited to, other anti-CTLA-4, anti-TIGIT, anti-PD-1, anti-PD-L1, and anti-PD-L2 antibodies. Patients who received agents targeting the adenosine pathway (eg, anti-CD73, anti-A2AR, anti-CD39), anti-NKG2A, anti-HLA-E agents, and anti-LIF agents are also excluded. Patients who have received previous treatment with a TROP2 targeting ADC or with another ADC containing a chemotherapy agent that inhibits TOP1 activity are also excluded.

21 Current or prior use of immunosuppressive medication within 14 days before the first dose of study interventions. The following are exceptions to this criterion:

- Intranasal, inhaled, topical steroids, or local steroid injections (eg, intra articular injection).
- Systemic corticosteroids ≤ 10 mg/day of prednisone or its equivalent.
- Steroids as premedication for hypersensitivity reactions (eg, CT scan premedication).
- Steroids as premedication for chemotherapy or for Dato-DXd.

Prior/concurrent clinical study experience

22 Participation in another clinical study with an investigational product administered within 30 days prior to enrolment.

23 Previous study interventions (durvalumab, oleclumab, monalizumab, volrustomig, Dato-DXd, AZD0171, or rilvegostomig) assignment in the present study.

Other Exclusions

24 Female patients who are pregnant or breastfeeding or male or female patients of reproductive potential who are not willing to employ effective birth control from the time of screening throughout the total duration of the study and for the following period after receiving the last dose of study interventions:

- Durvalumab or volrustomig: 90 days
- Oleclumab, monalizumab, or AZD0171: 180 days

- Dato-DXd: 210 days (female patients) and 120 days (male patients)
- Rilvegostomig: 60 days

For the chemotherapy agents the local prescribing information relating to contraception, the time limits for such precautions, and any additional restrictions for the agents administered must be followed. For patients receiving more than one study intervention, the longest washout period must be followed after the last dose of study interventions to prevent pregnancy.

25 Involvement in the planning and/or conduct of the study (applies to both AstraZeneca staff and/or staff at the study site).

26 Judgement by the Investigator that the patient should not participate in the study if the patient is unlikely to comply with study procedures, restrictions and requirements.

27 Exclusion criteria for participation in the optional (DNA) genetics research component of the study include the following:

- Previous allogeneic bone marrow transplant.
- Non-leukocyte-depleted whole blood transfusion in 120 days of genetic sample collection.

5.3 Lifestyle Considerations

The following restrictions apply while the patient is receiving study interventions and for the specified times before and after:

- Patients must follow the contraception requirements outlined in Appendix G.
- Patients should not donate blood or blood components while participating in this study and after receipt of the final dose of study interventions or until alternate anticancer therapy is started for the following duration:
  - Arm 1 (oleclumab): 180 days after receipt of the final dose
  - Arm 2 (monalizumab): 180 days after receipt of the final dose
  - Arm 3A, 3B and 3C (volrustomig): 90 days after receipt of the final dose
  - Arm 4 (Dato-DXd): 210 days after receipt of the final dose
  - Arm 5 (AZD0171): 180 days after the receipt of the final dose
  - Arm 6 (rilvegostomig): 60 days after receipt of the final dose
  - Arm 7 (Dato-DXd + rilvegostomig): 210 days after receipt of the final dose of Dato-DXd or 60 days after receipt of the final dose of rilvegostomig

For patients receiving more than one study intervention, the longest washout period must be

followed after the last dose of study interventions. Restrictions relating to concomitant therapies are described in Appendix H 1.

5.4 Screen Failures

Screen failures are patients who do not fulfil the eligibility criteria for the study and therefore must not be randomised. These patients should have the reason for study withdrawal recorded as "eligibility criteria not fulfilled" (ie, patient does not meet the required inclusion/exclusion criteria). This reason for study withdrawal is only valid for screen failures (ie, not randomised patients). Patients may be rescreened a single time, but they must not be re-randomised. Where a patient is permitted to re-screen (by the AstraZeneca physician), the same e-code initially assigned by the IRT should be used.

A minimal set of screen failure information is required to ensure transparent reporting of screen failure patients to meet the Consolidated Standards of Reporting Trials publishing requirements and to respond to queries from regulatory authorities. Minimal information includes demography, screen failure details, eligibility criteria, and any SAE.

Patient enrolment and randomisation is described in Section 6.3.

6 STUDY INTERVENTION

Study intervention is defined as any investigational intervention(s), marketed product(s), placebo, or medical device(s) intended to be administered to a study patient according to the CSP. Study intervention in this study refers to durvalumab, oleclumab, monalizumab, volrustomig, Dato-DXd, AZD0171, rilvegostomig, and chemotherapy.

6.1 Study Intervention(s) Administered

6.1.1 Investigational Products

AstraZeneca will supply all investigational products. Commercial preparations for chemotherapy agents will be used in this study and sourced locally. AstraZeneca may supply chemotherapy agents per local requirements.

Dose modifications are described in Section 6.6.

Table 5 Investigational Products

**Study Intervention Name**	Durvalumab (MEDI4736, IMFINZI™)	Oleclumab (MEDI9447)	Monalizumab [b] (IPH2201)	Volrustomig (MEDI5752)	Dato-DXd	AZD0171	Rilvegostomig
**Type**	Drug	Drug	Drug	Drug	Drug	Drug	Drug
**Dosage Form** [a]	Concentrate for solution for infusion	Concentrate for solution for infusion	Lyophilised product: Powder for concentrate for solution for infusion<br><br>Liquid product: Concentrate for solution for infusion	CCI [redacted] product for concentrate for solution for infusion	Lyophilised powder for concentrate for solution for infusion	Concentrate for solution for infusion	Concentrate for solution for infusion
**Formulation**	50 mg/mL durvalumab, 26 mM histidine/histidine hydrochloride, 275 mM trehalose dihydrate, 0.02% (w/v) polysorbate 80, pH 6.0	50 mg/mL oleclumab, 25 mM histidine/histidine hydrochloride, 240 mM sucrose, 0.03% (w/v) polysorbate 80, pH 6.0	50 mg/mL monalizumab, 20 mM histidine/histidine hydrochloride, 220 mM sucrose, 0.03% (w/v) polysorbate 80, pH 6.0	CCI [redacted]	20 mg/mL Dato-DXd, 10 mM histidine/histidine hydrochloride, 9% (w/v) sucrose, 0.03% (w/v) polysorbate 80, pH 6.0	CCI [redacted]	CCI [redacted]
**Density**	1.054 g/mL	1.05 g/mL	1.041 g/mL	CCI [redacted]	1.04 g/mL	CCI [redacted]	CCI [redacted]
**Label-claim volume**	10 mL	10 mL	7.5 mL (lyophilised DP, post-reconstitution)<br><br>15 mL (liquid DP)	CCI [redacted]<br>CCI [redacted]	5 mL (post-reconstitution)	CCI [redacted]	CCI [redacted]

**Dosing Instructions** [b, c]	1500 mg IV Q3W (pre-surgery) or Q4W (post-surgery) [d]	3000 mg IV Q3W (pre-surgery) or Q4W (post-surgery) [e]	1500 mg IV Q3W (pre-surgery) or Q4W (post-surgery)	CCI [redacted]	6.0 mg/kg IV Q3W (pre-surgery) [g]	CCI [redacted]	CCI [redacted]
**Route of Administration**	IV infusion	IV infusion	IV infusion	IV infusion	IV infusion	IV infusion	IV infusion
**Use**	Experimental	Experimental	Experimental	Experimental	Experimental	Experimental	Experimental
**IMP and NIMP**	IMP	IMP	IMP	IMP	IMP	IMP	IMP
**Sourcing**	Provided centrally by the Sponsor	Provided centrally by the Sponsor	Provided centrally by the Sponsor	Provided centrally by the Sponsor	Provided centrally by the Sponsor	Provided centrally by the Sponsor	Provided centrally by the Sponsor
**Packaging and Labelling**	Study intervention will be provided in 500 mg vials. Each 500 mg vial will be labelled in accordance with GMP Annex 13 and per country regulatory requirement	Study intervention will be provided in 500 mg vials. Each 500 mg vial will be labelled in accordance with GMP Annex 13 and per country regulatory requirement	Study intervention will be provided in 375 mg vials (lyophilised DP) and 750 mg vials (liquid DP). Each vial will be labelled in accordance with GMP Annex 13 and per country	CCI [redacted]	Study intervention will be provided in 100 mg vials. Each 100 mg vial will be labelled in accordance with GMP Annex 13 and per country regulatory requirement	CCI [redacted]	CCI [redacted]

			regulatory requirement				

[a] Refer to relevant appendix for preparation instructions for study interventions.

[b] Refer to relevant appendix for details on duration of treatment.

[c] The following sequence should be used for treatment administration in days when all or more than one agent is administered:

Arm 1: Oleclumab > durvalumab > platinum doublet chemotherapy

Arm 2: Monalizumab > durvalumab > platinum doublet chemotherapy

[redacted]

Arm 4: Dato-Dxd > durvalumab > single agent platinum chemotherapy (physician choice of carboplatin or cisplatin)

[redacted]

[d] If a patient's weight falls to 30 kg or below (≤ 30 kg), the patient should receive weight-based dosing equivalent to 20 mg/kg of durvalumab Q3W or Q4W after consultation between Investigator and Study Physician/Medical Scientist until the weight improves to > 30 kg, at which point the patient should start receiving the fixed dosing of durvalumab 1500 mg Q3W or Q4W. Durvalumab will be administered Q3W during the neoadjuvant period of the study and Q4W during the adjuvant phase of the study.

[e] If a patient's weight falls to 35 kg or below (≤ 35 kg), the patient should receive weight-based dosing equivalent to 40 mg/kg of oleclumab Q3W or Q4W after consultation between Investigator and Study Physician/Medical Scientist until the weight improves to > 35 kg, at which point the patient should start receiving the fixed dosing of oleclumab 3000 mg Q3W or Q4W. Oleclumab will be administered Q3W during the neoadjuvant period of the study and Q4W during the adjuvant phase of the study.

[f] [redacted]

[g] Premedication is required prior to any dose of Dato-DXd and must include antihistamines and antipyretics, preferably acetaminophen, with or without glucocorticoids.

[h] Monalizumab will be supplied as either a lyophilised dosage form that requires reconstitution prior to use, or a liquid dosage form. These dosage forms are clinically interchangeable.

Abbreviations: Dato-DXd: datopotamab deruxtecan; DP: Drug product; GMP: Good Manufacturing Practice; IMP: Investigational medicinal product; IV: Intravenous; Q2W: Every 2 weeks; Q3W: Every 3 weeks; Q4W: Every 4 weeks; WFI: Water for injection.

Table 6 Chemotherapy

Study Intervention Name	Chemotherapy [a, b]				
	Carboplatin/ paclitaxel	Pemetrexed/ cisplatin [c, d]	Pemetrexed/ carboplatin [d]	Carboplatin	Cisplatin
Type	Drug	Drug	Drug	Drug	Drug
Dosage Form [e]	As sourced locally	As sourced locally	As sourced locally	As sourced locally	As sourced locally
Dosing Instructions [f, g]	Carboplatin AUC 6 or AUC 5 and paclitaxel 175 or 200 mg/m^2 Q3W (pre-surgery)	Pemetrexed 500 mg/m^2 and cisplatin 75 mg/m^2 Q3W (pre-surgery)	Pemetrexed 500 mg/m^2 and carboplatin AUC 5 Q3W (pre-surgery)	Carboplatin AUC 6 or AUC 5 Q3W (pre-surgery)	Cisplatin 75 mg/m^2 Q3W (pre-surgery)
Route of Administration	IV infusion	IV infusion	IV infusion	IV infusion	IV infusion
Use	Experimental	Experimental	Experimental	Experimental	Experimental
IMP and NIMP	IMP	IMP	IMP	IMP	IMP
Sourcing	Sourced locally by site [a]	Sourced locally by site [a]	Sourced locally by site [a]	Sourced locally by site [a]	Sourced locally by site [a]
Packaging and Labelling	Sourced locally by site [a]	Sourced locally by site [a]	Sourced locally by site [a]	Sourced locally by site [a]	Sourced locally by site [a]

[a] Under certain circumstances when local sourcing is not feasible, chemotherapy agents may be supplied centrally through AstraZeneca.

[b] As per HOPA position statement, chemotherapy dosing can be rounded up to 10% at Investigator discretion and institutional guidelines (Fahrenbruch et al, 2018).

[c] In the event of unfavourable tolerability, patients can switch from cisplatin to carboplatin therapy at any point during the study (assuming eligibility for the switch as assessed by Investigator). In patients with comorbidities or unable to tolerate cisplatin per Investigator's judgement, carboplatin AUC 5 can be administered from cycle 1.

[d] Patients with non-squamous NSCLC only. Administer vitamin B12 and folic acid in line with local practice.

[e] Refer to Pharmacy Manual for preparation instructions for study interventions.

[f] Refer to relevant appendix for details on duration of treatment.

[g] The following sequence should be used for treatment administration in days when all or more than one agent is administered:

Arm 1: Oleclumab > durvalumab > platinum doublet chemotherapy
Arm 2: Monalizumab > durvalumab > platinum doublet chemotherapy
[redacted]
Arm 4: Dato-Dxd > durvalumab > single agent platinum chemotherapy (physician choice of carboplatin or cisplatin)
[redacted]

Abbreviations: AUC: Area under the serum drug concentration-time curve; Dato-DXd: datopotamab deruxtecan; HOPA: Hematology/Oncology Pharmacy Association; IMP: Investigational medicinal product; IV: Intravenous; NSCLC: Non-small cell lung cancer; Q2W: Every 2 weeks; Q3W: Every 3 weeks; Q4W: Every 4 weeks.

6.1.1.1 Study Interventions

Instructions for the storage, preparation and administration of study interventions are provided in the respective agent specific appendices. Further detailed instructions are provided in the Pharmacy Manual.

6.1.1.2 Chemotherapy

The chemotherapy agents will be locally sourced and will be administered according to prescribing information or treatment guidance in general use by the investigating site. Under certain circumstances when local sourcing is not feasible, AstraZeneca will centrally supply the drug, which will be labelled with local language translated text in accordance with regulatory guidelines. Chemotherapy infusion will start no less than 30 minutes after the end of the durvalumab infusion, volrustomig infusion, or rilvegostomig infusion.

6.1.2 Treatment Regimens

Patients will receive one of the treatment regimens indicated in Table 4.

The first day of dosing is considered Week 0, Day 1 in all treatment arms (Figure 1).

Further information on the treatment regimens including any premedication required is provided in the relevant appendix for each study intervention.

Guidance for proceeding to adjuvant treatment following surgery:

(Criteria to be assessed within 10 weeks from surgery, prior to starting adjuvant treatment).

1 Patients must have recovered from all acute, reversible toxic effects from previous treatments or other AEs that could potentially adversely impact further administration of adjuvant treatment according to the Investigator's judgement.

2 Patients should be able to start adjuvant treatment as soon as clinically feasible and within 10 weeks from surgery. A minimum of 3 weeks is recommended between NSCLC surgery and adjuvant treatment start (first post-surgical scan must be performed prior to starting adjuvant treatment and PORT, if required). Complete post-operative wound healing must have occurred following any surgery.

3 NOTE: In all instances where unresolved AEs may potentially prevent further administration of adjuvant treatment within 10 weeks after surgery, the Investigator must contact the Study Physician/Medical Scientist within a reasonable period of time (approximately 2 weeks) to obtain approval prior to the 10-week limit.

4 Patients with R0/1 margins are allowed to continue to adjuvant treatment.

5 For patients in which it is recommended, per local guidance, to receive PORT, this is permitted to occur off protocol. Post-operative radiotherapy is to be given within 8 weeks after surgery, adjuvant treatment must start no longer than 3 weeks after the end of PORT,

and, in this circumstance, may be more than 10 weeks after surgery. See Section 6.1.4 for PORT standardised guidance.

6 Patients who require re-resection according to Investigator's judgement will not be allowed to receive adjuvant treatment.

6.1.2.1 Chemotherapy

In Arms 1, 2, 3A, 3B, 3C, 5, and 6, patients will receive one of the following platinum doublet chemotherapy regimens, based on the tumour histology and Investigator's discretion, as part of their treatment regimen prior to surgery (National Comprehensive Cancer Network, 2022):

- Squamous tumour histology: Carboplatin + paclitaxel: carboplatin AUC 6 (maximum dose: 900 mg) or AUC 5 (maximum dose: 750 mg) and paclitaxel 175 or 200 mg/m^2 via IV infusion on Day 1 of each 3-week cycle, for 4 cycles (Figure 1).
- Non-squamous tumour histology: Pemetrexed + cisplatin: pemetrexed 500 mg/m^2 and cisplatin 75 mg/m^2 via IV infusion on Day 1 of each 3-week cycle, for 4 cycles (Figure 1). In the event of unfavourable tolerability, patients can switch from cisplatin to carboplatin therapy at any point during the study (assuming eligibility for the switched therapy is met). In patients with comorbidities or unable to tolerate cisplatin per Investigator's judgement, carboplatin AUC 5 (maximum dose: 750 mg) can be administered from Cycle 1.
- Non-squamous tumour histology: Pemetrexed + carboplatin: pemetrexed 500 mg/m^2 and carboplatin AUC 5 (maximum dose: 750 mg) via IV infusion on Day 1 of each 3-week cycle, for 4 cycles (Figure 1).

In Arms 4 and 7, patients will receive one of the following single agent platinum chemotherapies based on the Investigator's discretion, as part of their treatment regimen prior to surgery:

- Carboplatin AUC 6 (maximum dose: 900 mg) or AUC 5 (maximum dose: 750 mg) via IV infusion on Day 1 of each 3-week cycle, for 4 cycles.
- Cisplatin 75 mg/m^2 via IV infusion on Day 1 of each 3-week cycle, for 4 cycles. In the event of unfavourable tolerability, patients can switch from cisplatin to carboplatin therapy at any point during the study (assuming eligibility for the switch as assessed by Investigator).

Note: Maximum dosing for carboplatin is based on FDA's recommendation that physicians cap the dose for desired exposure (AUC) to avoid potential toxicity due to overdosing. The maximum dose is based on a GFR estimate that is capped at 125 mL/min for patients with normal renal function (National Comprehensive Cancer Network, 2020).

Chemotherapy will be administered according to individual product labels, including, when necessary, dose adjustments. For patients with renal impairment, dosing of carboplatin, pemetrexed and cisplatin should follow their respective label.

Pemetrexed should not be administered to patients with CrCL < 45 mL/min, per its label.

6.1.3 Surgery

Evaluation

The determination of resectability, surgical staging, and pulmonary resection should be performed by a board-certified thoracic surgeon who performs lung cancer surgery as a prominent part of his/her practice. Thoracic surgeons should actively participate in multidisciplinary discussions and meetings regarding patients within the study.

Computed tomography and PET used for staging should be within 30 days before proceeding with surgical evaluation (National Comprehensive Cancer Network, 2022).

Preoperative mediastinal lymph node staging

Mediastinal staging is very important, as it provides accurate information on the extent of the disease, guides the choice of treatment, and determines the patient's prognosis. Lymph node mapping is defined by The IASLC (IASLC Staging Manual in Thoracic Oncology 2016) lymph node map. The European Society of Thoracic Surgeons guidelines (Curran et al, 2010, De Leyn et al, 2014) on imaging, endoscopic, and surgical techniques for lymph nodes staging should be followed. The staging criteria for NSCLC and lymph node definition are discussed in AJCC Cancer Staging Manual, 8th Edition.

In case of CT-enlarged or PET-positive mediastinal lymph nodes, tissue confirmation is recommended. Endosonography (EBUS/EUS) with FNA is the first choice (when available), since it is minimally invasive and has a high sensitivity to rule in mediastinal nodal disease. If negative, surgical staging with nodal dissection or biopsy is indicated. Video-assisted mediastinoscopy is preferred to mediastinoscopy. The combined use of endoscopic staging and surgical staging results in the highest accuracy. When there are no enlarged mediastinal lymph nodes on CT and when there is no uptake in lymph nodes on PET or PET–CT (cN0), direct surgical resection with systematic nodal dissection is indicated for tumours ≤ 3 cm located in the outer third of the lung. In central tumours or cN1, cN2, preoperative mediastinal staging is indicated. The choice between endoscopic staging with EBUS/EUS and FNA or video-assisted mediastinoscopy depends on local expertise to adhere to minimal requirements for staging. For tumours > 3 cm, preoperative mediastinal staging is advised, mainly in adenocarcinoma with high standardised uptake value.

Pre-treatment risk assessment

The ESMO recommendations for pre-treatment risk assessment should be followed. Overall,

the cardiopulmonary fitness of the patient will determine the choice of treatment. Before considering surgical resection, precise assessment of cardiac and pulmonary function is necessary to estimate risk of operative morbidity. For cardiac assessment, use of revised cardiac risk index is recommended. The revised cardiac risk index includes a number of weighted factors, including ischaemic heart disease, history of cerebrovascular disease, serum creatine > 2 mg/dL, and planned pneumonectomy. Each factor is assigned a point value, and patients are grouped into classes based on the total number of points. If a patient has at least 3 weighted factors or any cardiac condition requiring medications, a newly suspected cardiac condition, or inability to climb 2 flights of stairs, then a cardiac consultation with non-invasive cardiac testing treatments should be performed according to American Heart Association/American College of Cardiology guidelines. If there is a need for coronary intervention (coronary artery bypass grafting or percutaneous coronary intervention), then surgery should be postponed for 6 weeks or more. If ongoing cardiac care may continue, or any needed new medical interventions are instituted (ie, beta blockers, anticoagulants, or statins), then the patient may proceed with lung function tests.

Formal lung function testing should be undertaken to estimate post-operative lung function (see Section 8.2.7).

Comorbidities should be evaluated and optimised before surgery. In patients with limited pulmonary function due to emphysema, a lung volume reduction effect may be observed by resection of the lung cancer within emphysematous lung tissue (Paz-Ares et al, 2021, Postmus et al, 2017).

Resection

The recommendations of ESMO and NCCN for the resectability of NSCLC must be followed. Anatomic pulmonary resection is preferred for the majority of patients with NSCLC. Either open thoracotomy or video-assisted thoracoscopic surgery access can be carried out as appropriate to the expertise of the surgeon. Video-assisted thoracoscopic surgery or minimally invasive surgery (including robotic-assisted approaches) should be strongly considered for patients with no anatomic or surgical contraindications, as long as there is no compromise of standard oncologic and dissection principles of thoracic surgery. Lung sparing anatomic resection (sleeve lobectomy) is preferred over pneumonectomy, if anatomically appropriate and margin-negative resection is achieved. T3 (invasion) and T4 local extension tumours require en-bloc resection of the involved structure with negative margins. If a surgeon or centre is uncertain about potential complete resection, consider obtaining an additional surgical option from a high-volume specialised centre (National Comprehensive Cancer Network, 2022).

Nodal assessment

The NCCN guidelines must be followed for margins evaluation and nodal assessment.

N1 and N2 node resection and mapping should be a routine component of lung cancer resections. Accordingly, it is recommended that a minimum of 3 lobe-specific mediastinal nodal stations (N2) or complete lymph node dissection, one of which should include station 7, and at least 1 N1 station—inclusive of the ones removed with the pulmonary specimen—have been sampled at the end of the procedure. Formal ipsilateral mediastinal lymph node dissection is indicated for patients undergoing resection for N2 disease.

Margins

Complete resection requires free resection margins, systematic node dissection or sampling, and the highest mediastinal node negative for tumour. The resection is defined as incomplete whenever there is involvement of resection margins, unremoved positive lymph nodes, or positive pleural or pericardial effusions. A complete resection is referred to as R0 and microscopically positive resection as R1. In the case of R2 (macroscopic involvement of resection margins), patients will not be eligible for adjuvant therapy and should be treated in accordance with local SoC.

N2 disease

The presence or absence of N2 disease should be vigorously determined by both radiologic and invasive staging prior to the initiation of therapy, since the presence of mediastinal nodal disease has a profound impact on prognosis and treatment decisions. Patients with occult positive N2 nodes discovered at the time of pulmonary resection should continue with the planned resection along with formal mediastinal lymph node resection.

The determination of the role of surgery in a patient with N2-positive lymph nodes should be made prior to the initiation of any therapy by a multidisciplinary team, including a board-certified thoracic surgeon who has a major part of his/her practice dedicated to thoracic oncology. The presence of N2-positive lymph nodes substantially increases the likelihood of positive N3 lymph nodes.

Pathologic evaluation of the mediastinum must include evaluation of the subcarinal station and contralateral lymph nodes. EBUS ± EUS are additional techniques for minimally invasive pathologic mediastinal staging that are complementary to mediastinoscopy. Even when these modalities are employed, it is important to have an adequate evaluation of the number of stations involved and biopsy and documentation of negative contralateral lymph node involvement prior to a final treatment decision. Repeat mediastinoscopy, while possible, is technically difficult and has a lower accuracy compared to primary mediastinoscopy. One possible strategy is to perform EBUS (± EUS) in the initial pre-treatment evaluation and reserve mediastinoscopy for nodal restaging after neoadjuvant therapy. See NCCN guidelines for more information.

Guidance for proceeding to surgery following completion of neoadjuvant treatment

(Criteria must be assessed within 30 days prior to surgery.)

1 Patients should receive 4 cycles of neoadjuvant treatment as indicated in.

   (a) Receipt of less than 4 cycles of neoadjuvant treatment will be permitted if the patient experiences treatment-related toxicities and the Investigators judge additional safety issues will be expected with additional cycles of therapy or if the patient experiences disease progression and Investigators judge that surgery should not be further delayed until the end of neoadjuvant treatment.

   (b) In cases where the patient has completed less than 4 cycles of any of the components of neoadjuvant treatment and is able to continue with other agents in treatment arm, then the remaining cycle(s) should be administered before proceeding to surgery.

   (c)

   (d) Note: In all instances where toxicity is expected to lead to missing or withholding a dose of either chemotherapy or durvalumab, oleclumab, monalizumab, volrustomig, Dato-DXd, AZD0171, or rilvegostomig, or all, the Investigator MUST discuss the case with the Study Physician/Medical Scientist before proceeding to surgery.

   (e)

2 Surgery should happen within 40 days from the last dose of study interventions (Day 1 is the day after completion of last dose of study interventions) administration or it will be considered delayed. Reasons for delay must be reported.

   3 NOTE: Cases where surgery cannot be completed within 40 days from the last dose of study interventions needs be discussed with the Study Physician/Medical Scientist.

4 Patients must have recovered from all acute, reversible toxic effects from chemotherapy (excluding alopecia) and durvalumab, oleclumab, monalizumab, volrustomig, Dato-DXd, AZD0171, or rilvegostomig that could potentially adversely impact the surgical procedure or outcome according to the Investigator's judgement.

5 A contrast-enhanced CT/MRI scan of chest and abdomen (including the entire liver and both adrenals) is required for RECIST 1.1 assessment and for surgical planning prior to surgery. A supplemental (whole body) FDG-PET scan should also be acquired prior to surgery in order to help identify mediastinal lymph node involvement, according to the Investigator's judgement.

   6 NOTE: If apparent nodal progression is identified, then a pathologic confirmation is mandated if this finding would make the patient unresectable according to the multidisciplinary evaluation. In those instances, pathologic confirmation should drive decisions on resectability, and surgery should occur under all conditions, unless nodal pathologic confirmation unequivocally dictates otherwise.

7 If preoperative CT and/or PET are suspicious for mediastinal nodal involvement or should those be negative for mediastinal lymph node but there is T > 3 cm, cN1, or central tumour, then it is recommended that invasive mediastinal staging with thoracoscopy or mediastinoscopy or endobronchial ultrasound-guided transbronchial needle aspiration be performed if those were not performed at screening and/or according to the multidisciplinary evaluation and Investigator's judgement.

8 The patient should be deemed to have adequate cardiac and lung function, according to a multidisciplinary assessment (See Section 8.2.7).

6.1.4 Post-operative radiotherapy standardised guidance

Post-operative radiotherapy is allowed for patients in which it is indicated according to local guidance. A standardised course of PORT will include, but is not limited to, doses ranging from 50 to 60 Gy, 1.8 to 2 Gy per fraction, 5 fractions a week; for patients with positive margins of disease (R1), PORT will include, but is not limited to, doses ranging from 60 to 66 Gy, 1.8 to 2 Gy per fraction, 5 fractions a week.

Either IMRT or 3D-CRT is allowed, though IMRT is preferred.

Motion assessment is required, with motion management depending on the results of the motion assessment. Four-dimensional CT simulation is the preferred method for motion assessment. Motion management acceptable forms require that the motion management method employed by the participating institution reduces the effective motion of the target to ≤ 10 mm. Some form of immobilisation is required, with attention to patient comfort to prevent intra-fraction motion. Patients must be immobilised in a stable position using the participating institution's standard of practice.

The treatment planning CT is required for defining target volumes and organs-at-risk. Post-operative radiation therapy must not start until the first post-surgery RECIST 1.1 scan has been completed.

6.1.5 Duration of Treatment

Neoadjuvant treatment in Arms 1 to 7 will be administered for 4 cycles Q3W. Surgery is to be performed within 40 days from last dose of study interventions. After surgery, adjuvant treatment in Arm 1, Arm 2, and Arm 4 will be administered Q4W; treatment in Arm 3A, 3B and 3C will be administered [redacted] treatment in Arm 5 will be administered [redacted] (AZD0171) and [redacted] (durvalumab), and treatment in Arms 6 and 7 will be administered [redacted] (rilvegostomig) (Figure 1). Patients should be able to start adjuvant treatment following surgery as soon as clinically feasible and within 10 weeks from surgery (except for patients receiving PORT, which must be started within 8 weeks after surgery. Adjuvant treatment must be started within 3 weeks from the end of PORT).In the neoadjuvant treatment period (lasting for 4 cycles), treatment will be stopped at RECIST 1.1-defined radiological PD or clinical

progression unless it is confirmed by the Investigator in agreement with the Study Physician/Medical Scientist that the patient continues to have a resectable tumour and continues to have clinical benefit. In the adjuvant treatment period (lasting for 1 year), treatment will be stopped at RECIST 1.1-defined radiological PD or clinical progression, unless it is confirmed by the Investigator in agreement with the Study Physician/Medical Scientist that the patient continues to have clinical benefit. Treatment will be stopped at RECIST 1.1-confirmed radiological PD.

Patients with rapid tumour progression or with symptomatic progression that requires urgent medical intervention (eg, central nervous system metastasis, respiratory failure due to tumour compression, or spinal cord compression) will not be eligible for continuing study intervention but should be followed-up for survival. Likewise, if tumour resection was not achievable at surgery, the patient will not be eligible to continue on adjuvant treatment but should be followed-up for survival.

Crossover between treatment arms within the study will not be permitted.

6.1.6 Post final data cut-off

Efficacy scans post final DCO will be collected in accordance with local clinical practice. Investigators should continue to monitor and document data for all study patients in their source notes after scheduled DCO for final analysis of the adjuvant phase and DBL. Dependent on the analysis results, a decision may be made to continue further data collection for a longer period with intent to analyse long-term EFS, DFS, OS and safety data to fulfil any other potential Health Authority requirements. Any additional long-term analysis may be further clarified through an addendum to the main SAP, which will be developed for the long-term analysis.

Post final analysis DCO, recording and follow-up of SAEs, overdose, and pregnancy will be completed by sites via paper. Investigators will report all SAEs, overdose, and pregnancy to the Sponsor until 90 days after receipt of their last dose of any study interventions. Drug accountability data will be collected by sites.

Assessments will revert to the site local SoC. These data will be collected until any remaining patients have been transferred into a roll-over study (if available).

In the event that a rollover or safety extension study is available, patients may be transitioned to such a study, and the current study would reach its end. The rollover or safety extension study would ensure follow-up with visit assessments per its protocol. Any patient who would be proposed to move to such a study would be given a new informed consent. Continuation of treatment after the end of the study (ie, after cessation of all study-related assessments) is described in Section 6.7.

6.2 Preparation/Handling/Storage/Accountability of Interventions

1 Information on the preparation of study interventions is provided in the respective appendices for each treatment arm. Further information is provided in the Pharmacy Manual.

2 The Investigator or designee must confirm appropriate temperature conditions have been maintained during transit for all study interventions received and any discrepancies are reported and resolved before use of the study interventions.

3 Only patients enrolled in the study may receive study interventions and only authorised site staff may supply or administer study interventions. All study interventions must be stored in a secure, environmentally controlled, and monitored (manual or automated) area in accordance with the labelled storage conditions with access limited to the Investigator and authorised site staff.

4 The Investigator, institution, or the head of the medical institution (where applicable) is responsible for study interventions accountability, reconciliation, and record maintenance (ie, receipt, reconciliation, and final disposition records).

5 Further guidance and information for the final disposition of unused study interventions are provided in the Pharmacy Manual.

6.3 Measures to Minimise Bias: Randomisation and Blinding

6.3.1 Patient Enrolment and Randomisation

All patients will be centrally assigned to randomised study interventions using an IRT. Before the study is initiated the call/log-in directions and user guides for IRT will be provided to each site. Every attempt should be made to randomise the patient as close as possible to Day 1 of Cycle 1 and not more than 72 hours (3 days) prior to Day 1.

Study interventions will be dispensed at the study visits summarised in the SoA per SoC after DCO (refer to the SoA for each study intervention in the relevant appendix). Returned study interventions should not be re-dispensed to the patients.

If a patient withdraws from the study, then his/her enrolment/randomisation code cannot be reused. Withdrawn patients will not be replaced.

Investigators should keep a record (ie, the patient screening log) of patients who entered screening.

At screening/baseline (Days -28 to -1), the Investigator or suitably trained delegate will:

- Obtain signed informed consent before any study specific procedures are performed. If laboratory or imaging procedures were performed for alternate reasons prior to signing consent, these can be used for screening purposes with consent of the patient. However,

all screening laboratory and imaging results must have been obtained within 28 days of randomisation (with the exception of PET scan [up to 42 days]). For patients with a single TL, if screening biopsy is collected prior to screening imaging for baseline tumour assessment, allow approximately 2 weeks before imaging scans are acquired. (Informed consent of study procedures may be obtained prior to the 28-day screening)

- Obtain a unique enrolment number, through the IRT. This number is the patient's unique identifier and is used to identify the patient on the eCRFs.
- If *PD-L1*, *EGFR*, or *ALK* status is unknown, obtain tumour sample and send for local *PD-L1*, *EGFR*, and *ALK* testing. Screening procedures may be obtained while *PD-L1*, *EGFR*, and *ALK* status is being assessed. Patients with unknown *PD-L1* status, and *EGFR/ALK* status at time of randomisation will be considered screen failures and will not be eligible for randomisation in the study. In the case of screen failures due to late and/or missing results for *PD-L1*, *EGFR* or *ALK* status, rescreening will be permitted.

  For *EGFR*, *ALK*, and *PDL1* testing:

  - All patients will be tested for PD-L1.
  - Patients enrolling into Arms 6 and 7 will be restricted to baseline PD-L1 expression status ≥ 1%. When Arms 6 and 7 are open for enrolment, no other arms will be open for enrolment concurrently.
  - All patients will be tested for *EGFR*.
  - With the exception of patients with squamous cell carcinoma, all patients will be tested for *ALK*.
- Confirm tumour tissue pathology (see Sections 5.1 and 5.2).
- Obtain signed informed consent for genetic research study (optional).
- Record demographic data and other characteristics, including date of birth, age, sex, smoking history, and race/ethnicity, according to local regulations.

At randomisation, once the patient is confirmed to be eligible, the Investigator or suitably trained delegate will:

- Define the chemotherapy regimen (based on the most appropriate option for the patient [ie, per tumour histology and Investigator's discretion]; the reason for this choice should be documented) that the patient should receive prior to randomisation of the patient. This must be completed for all patients.
- Obtain a unique randomisation number via the IRT. Numbers will start at 001 and will be assigned strictly sequentially by IRT as patients are eligible for randomisation into the study. The system will randomise the eligible patient to 1 of the treatment groups open for enrolment. (The *PD-L1* expression result [< 1%, 1% to 49%, ≥ 50%] must be known prior to randomisation).

Note: For all patients with non-squamous tumour histology scheduled to receive pemetrexed, folic acid, and vitamin B12 should commence prior to treatment initiation for up to 7 days, in line with local practice. This is to ensure treatment can begin on Day 1.

If the patient is ineligible and not randomised, the IRT should be contacted to screen fail the patient in the system.

Patients will begin treatment on Day 1. Treatment should start no more than 3 days after being randomised. Patients must not be randomised and treated unless all eligibility criteria have been met.

6.3.2 Procedures for Handling Incorrectly Randomised Patients

Patients who fail to meet the eligibility criteria should not, under any circumstances, be randomised or receive study interventions. There can be no exceptions to this rule. Patients who are enrolled but subsequently found not to meet all the eligibility criteria must not be randomised or started on study interventions and must be withdrawn from the study and documented as a screen failure.

Where a patient does not meet all the eligibility criteria but is randomised in error, or incorrectly started on treatment, the Investigator should inform the AstraZeneca Study Physician/Medical Scientist immediately, and a discussion should occur between the AstraZeneca Study Physician/Medical Scientist and the Investigator regarding whether to continue or discontinue the patient from treatment. The AstraZeneca Study Physician/Medical Scientist must ensure all decisions are appropriately documented, if the decision is for the patient to remain on the study and that the potential benefit/risk profile remains positive for the patient.

6.3.3 Methods for Assigning Treatment Groups

A randomisation method with dynamically changing allocation ratio of treatment assignment will be employed to account for fluctuations in the number of enrolling treatment arms over the course of the study and to allow an increase in enrolment to one or more treatment arms at the discretion of the Sponsor and/or recommendation by the SRC. Where there is only a single arm enrolling, all patients will be allocated to that arm. Any changes to the allocation ratio will be communicated in a timely manner to the Investigators and patients will be informed by the Investigator or delegate of the enrolling treatment arms at the time of informed consent. The actual treatment given to patients will be determined by the randomisation scheme in the IRT. The randomisation scheme will be produced by a computer software programme that incorporates a standard procedure for generating randomisation numbers. One randomisation list will be produced for each of the randomisation strata. A blocked randomisation will be generated, and randomisation will be balanced within the IRT at the central level.

Randomisation codes will be assigned strictly sequentially, as patients become eligible for randomisation. The IRT will provide the kit identification number to be allocated to the patient at the randomisation visit and subsequent treatment visits.

This is an open-label study; however, the specific study interventions to be taken by a patient will be assigned using an IRT. The site will contact the IRT prior to the start of study interventions administration for each patient. The site will record the study interventions assignment on the applicable eCRF, if required. Potential bias will be reduced by the use of central randomisation.

6.4 Study Intervention Compliance

The study interventions will be administered only at the investigational sites by the authorised study personnel. As a result, treatment compliance is ensured. The date, and time if applicable, of dose administered in the centre will be recorded in the source documents and recorded in the eCRF. The dose of study interventions and study patient identification will be confirmed at the time of dosing by a member of the study site staff other than the person administering the study interventions.

Any dose reductions, change from the dosing schedule, dose delays/interruptions, and dose discontinuations should be recorded in the eCRF.

6.5 Concomitant Therapy

Any concomitant treatment, procedure, or other medication considered necessary by the Investigator for the patient's safety and wellbeing, or vaccine (including OTC or prescription medicines, vitamins, and/or herbal supplements) or other specific categories of interest that the patient is receiving from the time of screening or receives during the study including the 90-day follow-up period following the last dose of study interventions must be recorded in the eCRF along with:

- Reason for use.
- Dates of administration including start and end dates.
- Dosage information including dose and frequency.

The Study Physician/Medical Scientist should be contacted if there are any questions regarding concomitant or prior therapy.

If any concomitant therapy is administered due to new or unresolved AE, it should be recorded.

Patients must be instructed not to take any medications, including OTC products, without first consulting with the Investigator.

Restricted, prohibited, and permitted concomitant medications/therapies are described in more detail in Appendix H 1.

Per the paclitaxel label, caution should be exercised when paclitaxel is administered concomitantly with known substrates or inhibitors of the cytochrome P450 isoenzymes CYP2C8 and CYP3A4.

For chemotherapy agents, refer to the local prescribing information with regard to warnings, precautions, and contraindications.

Drug-drug Interactions

There is no information to date on drug-drug interactions with durvalumab, oleclumab, monalizumab, volrustomig, AZD0171, or rilvegostomig, either pre-clinically or in patients. As the study interventions are mAbs and therefore proteins, they will be degraded to small peptides and amino acids and will be eliminated by renal and reticuloendothelial CL. It is therefore not expected that the study interventions will induce or inhibit the major drug metabolising cytochrome P450 pathways. As a result, there are no expected PK drug-drug interactions. Based on the mechanism of action of the study interventions, no significant pharmacodynamic drug interactions with the commonly administered concomitant medications are expected. Despite this, appropriate clinical monitoring in all of the planned clinical studies will be conducted to evaluate any potential drug-drug interactions.

For Dato-DXd, in vitro studies do not report clinically significant DDIs for the MAAA-1181a drug component which is substrate of P-gp, MATE2-K, OATP1B1, OATP1B3, BCRP, MRP1 and CYP3A4.

6.5.1 Rescue Medication

As a result of imAEs that could potentially be experienced by patients on durvalumab, volrustomig or rilvegostomig, appropriate treatment (eg, steroids and specific immunosuppressant rescue medications) must be made readily available by the site to this population.

Investigators can utilise the specific TMGs for management of imAEs. Investigators can also use locally available rescue medications per best clinical judgement and local institutional guidelines for management of imAEs. All rescue medication should be sourced locally by the study site.

The following rescue medications are recommended to be made available at the site:

1 Infliximab or infliximab biosimilar (eg, for colitis).

2 Mycophenolate (eg, for hepatitis).

Under certain circumstances when local sourcing by the study site is not feasible or local regulations prevent the use of infliximab or mycophenolate for this use (as they are considered off-label for management of immunotherapy related toxicities) as allowed by local regulations, AstraZeneca will centrally supply the required rescue medications, which will be labelled and accompanied by package insert with local language translated text in accordance with regulatory guidelines. Accountability and storage requirements, as specified in Section 6.2, apply for any study intervention supplied by AstraZeneca.

The date of rescue medication administration as well as the name and dosage regimen of the rescue medication must be recorded.

6.6 Dose Modification

Dose delays are permitted for durvalumab, oleclumab, monalizumab, volrustomig, Dato-DXd, AZD0171, and rilvegostomig (see Section 6.6.1).

Dose reduction is not permitted for durvalumab, oleclumab, monalizumab, volrustomig (Arms 3B and 3C), AZD0171, or rilvegostomig. Dose reductions are permitted for Dato-DXd (see Appendix N 6.4) and volrustomig Arm 3A only (see Appendix M 7.3.3).

Dose will need to be recalculated if patient weight falls below 30 kg (durvalumab), 35 kg (oleclumab) or if weight changes by ≥ ± 10% (Dato DXd) (see Pharmacy Manual). After the recalculation, the updated patient's weight will be used as the new baseline weight. The site may follow local institutional policy for recalculating dose based on weight changes less than 10%.

Dose reductions of chemotherapy agents **are permitted**, according to the locally approved prescribing information (label), local standard clinical practice or national guidance.

For patients with renal impairment dosing of carboplatin, pemetrexed and cisplatin should follow their respective label or Package Insert. Pemetrexed should not be administered to patients with CrCL < 45 mL/min.

6.6.1 Dose Delays

Dose administration of durvalumab, oleclumab, monalizumab, volrustomig, Dato-DXd, AZD0171, or rilvegostomig can be delayed up to 3 days from the planned dose on Day 1 of each cycle due to TRAE. If delayed for > 3 days, patients will skip the planned dose and can resume at next scheduled dosing date.

As a general guidance, durvalumab, oleclumab, monalizumab, volrustomig, Dato-DXd, AZD0171, or rilvegostomig dose may be delayed for any Grade ≥3 AE that does not meet criteria for treatment discontinuation, unless agent-specific TMGs require permanent

discontinuation or have additional requirements (refer to the TMGs, Section 8.3.15) and whose causal relationship is attributable to durvalumab, oleclumab, monalizumab, volrustomig, Dato-DXd, AZD0171, or rilvegostomig. Based on the investigator's discretion, a scheduled dose of any of these study interventions should be skipped/held to permit resolution or improvement to Grade 1 or baseline per the following:

- A maximum of 4 non-consecutive doses may be skipped/held throughout the treatment period to permit resolution of the Grade ≥ 3 AE(s).
- A maximum of 3 consecutive doses may be skipped/held throughout the treatment period (to allow up to 12 weeks without treatment) to permit resolution of the Grade ≥ 3 AE(s).
- If the toxicity does not resolve to Grade ≤ 1 or to baseline after skipping/holding dose(s) as allowed per protocol, the patient will be permanently discontinued from treatment.
- If volrustomig dose is skipped/held due to toxicity, the following criteria must be fulfilled prior to resuming dosing:
  - Toxicity must resolve or improve to Grade ≤ 1 or to baseline (refer to TMGs [separate annex to the protocol] for required criteria and time course of resolution of specific toxicities).
  - Benefit-risk profile must be favourable.
  - Discussion and agreement to resume dosing with Medical Monitor.
- For patients who develop ≥ Grade 2 pulmonary oedema or ≥ Grade 3 peripheral oedema after treatment with durvalumab plus oleclumab, doses of the agent causing the AE should be omitted and may be discontinued at the discretion of the Investigator.
- A dose of Dato-DXd can be delayed for up to 3 consecutive cycles from the planned date of administration. If a patient is assessed as requiring a dose delay longer than 3 consecutive cycles, the patient must discontinue study treatment with Dato-DXd.
- If AZD0171 is withheld because of toxicity for more than 8 weeks (approximately 56 days), it is recommended the investigator contacts the study monitor if treatment planning is to be resumed.

If dosing must be delayed for reasons other than treatment-related toxicity, dosing will resume as soon as feasible.

Refer to the study intervention's TMGs for detailed guidance on dose delays and discontinuation.

Chemotherapy dose delays are permitted according to the locally approved prescribing information (label), local standard clinical practice or national guidance.

Surgery should not be delayed to catch up on treatment and should proceed after

administration of 4 cycles of other agent(s).

6.7 Continued Access to Intervention after the End of the Study

As described in Section 4.4, the study will close once all patients have completed their last scheduled visit or last scheduled procedure (SoA, Table 2).

After the final DCO for this study, AstraZeneca will continue to supply the study interventions to patients who received the study interventions, if in the opinion of their treating physician, the patients are continuing to derive clinical benefit until PD occurs as judged by the Investigator or until meeting any other discontinuation criteria as defined in Section 7.1. Patients should be followed according to the institution's standard of care assessments. No further data collection is required, except for reporting of SAEs and patient survival.

In the event that product development reaches a point where alternative product supply options become available, then these alternative product supply options will be discussed by AstraZeneca with the investigator. AstraZeneca will work with the investigator to transition the patient(s) to alternative supply, where possible.

In the event that a roll-over or safety extension study is available at the time of the final DCO and database closure, patients receiving treatment with study interventions may be transitioned to such a study, and the current study would reach its end. The roll-over or extension study would ensure treatment continuation with visit assessments per its protocol, as applicable. Any patient who would be proposed to move to such a study would be given a new informed consent, as applicable.

7 DISCONTINUATION OF STUDY INTERVENTION AND PATIENT DISCONTINUATION/WITHDRAWAL

7.1 Discontinuation of Study Interventions

It may be necessary for a patient to permanently discontinue (definitive discontinuation) one or more study interventions. If study interventions are permanently discontinued, the patient will remain in the study to be evaluated for safety and survival follow-up as described in the SoA (Table 2). A patient that decides to discontinue study interventions will always be asked about the reason(s) and the presence of any AEs. The reason for discontinuation should be documented in the source document and the appropriate section of the eCRF. The patient should continue attending subsequent study visits, and data collection should continue according to the CSP. If the patient does not agree to continue in-person study visits, a modified follow-up must be arranged to ensure the collection of endpoints and safety information. This follow-up could be a telephone contact with the patient (if agreed to by the patient and in compliance with local data privacy laws/practices), a contact with a relative or treating physician, or information from medical records. The approach taken should be

recorded in the medical records. A patient that agrees to modify follow-up is not considered to have withdrawn consent or to have withdrawn from the study.

Patients who have permanently discontinued from further receipt of all study interventions will be discontinued from the IRT.

In the event that study interventions are held or discontinued due to treatment-related toxicity, chemotherapy may still be administered as scheduled. If chemotherapy is held due to treatment-related toxicity, study interventions may still be administered at the Investigator's discretion when toxicity resolves to Grade 2 or less. Note: if the Investigator feels that a patient is ready to restart treatment prior to the toxicity resolving to Grade 2 or less, AstraZeneca should be consulted for an exception to this rule. If chemotherapy is permanently discontinued due to treatment-related toxicity, study interventions may continue at the Investigator's discretion, and AstraZeneca should be consulted.

If a patient discontinues treatment with one or more agent(s) due to toxicity, treatment may be continued with the other agent(s) including chemotherapy as long as the patient is continuing to show clinical benefit, as judged by the Investigator and Sponsor, and in the absence of discontinuation criteria.

An individual patient will not receive any further study interventions if any of the following occur in the patient in question:

- RECIST 1.1-defined radiological progression (refer to Section 8.1.1 and Appendix F).
- Investigator determination that the patient is no longer benefiting from study intervention.
- An AE that, in the opinion of the investigator or AstraZeneca, contraindicates further dosing.
- Any AE that meets criteria for discontinuation defined in the TMGs (see Section 8.3.15) or as defined in the local prescribing information for the chemotherapy agent.
- Patient decision. The patient is at any time free to discontinue treatment, without prejudice to further treatment. A patient who discontinues treatment is normally expected to continue to participate in the study (eg, for safety and survival follow-up) unless they specifically withdraw their consent to all further participation in any study procedures and assessments (see Section 7.2).
- Significant non-compliance with the CSP as judged by the Investigator or AstraZeneca.
- Pregnancy or intent to become pregnant.
- Initiation of subsequent anticancer therapy, including another investigational agent.
- Clinical progression as determined by the Investigator.

- Patients who are unable to complete surgery due to reasons other than PD discovered during the procedure.
- Enrolment in any other clinical trial involving an investigational product or any other type of medical research judged not be compatible with this study. Any case where a subject wishes to enrol in any form of trial while on study should be discussed to obtain approval from the Sponsor.

Note that discontinuation from study intervention is NOT the same thing as a withdrawal from the study.

7.1.1 Follow-up for Safety

If study interventions are permanently discontinued, the patient will remain in the study to be evaluated for safety and survival follow-up as described in the SoA (Table 2). Patients who are permanently discontinued from receiving study intervention will be followed for safety, including the collection of any protocol-specified blood specimens, unless consent is withdrawn, or the patient is lost to follow-up or administered subsequent therapy.

See the SoA (Table 2) for data to be collected at the time of study interventions discontinuation (ie, the end-of-treatment visit) and follow-up and for any further evaluations that need to be completed.

7.1.2 Follow-up for Survival

Patients will be followed up for survival status as indicated in the SoA (Table 2) until death, withdrawal of consent, or the end of the study. Survival information may be obtained via telephone contact with the patient or the patient's family, or by contact with the patient's current physician. Additional assessments to be performed at the time of survival follow-up are detailed in the SoA.

7.2 Patient Withdrawal from the Study

- A patient may withdraw from the study at any time at his/her own request or may be withdrawn at any time at the discretion of the Investigator for safety, behavioural, compliance, or administrative reasons.
- A patient who considers withdrawing from the study must be informed by the Investigator about modified follow-up options to ensure the collection of endpoints and safety information including new AEs and follow-up on any ongoing AEs and concomitant medications (eg, telephone contact at 30 days (+7 days) after study interventions are discontinued, a contact with a relative or treating physician, or information from medical records).
- At the time of withdrawal from the study, if possible, an End of Treatment and/or End of Study visit should be conducted, as shown in the SoA (Table 2). See SoA for data to be

collected at the time of study withdrawal and follow-up and for any further evaluations that need to be completed.

  - The patient will discontinue the study interventions and be withdrawn from the study at that time.

- If the patient withdraws consent for disclosure of future information, the Sponsor may retain and continue to use any data collected before such a withdrawal of consent.
- If a patient withdraws from the study, it should be confirmed if he/she still agrees for existing samples to be used in line with the original consent. If he/she requests withdrawal of consent for use of samples, destruction of any samples taken and not tested should be carried in line with what was stated in the informed consent and local regulation. The Investigator must document the decision on use of existing samples in the site study records and inform the Global Study Team.

7.3 Lost to Follow-up

A patient will be considered lost to follow-up if he or she repeatedly fails to return for scheduled visits and no contact has been established by the time the study is completed (see Section 4.4), such that there is insufficient information to determine the patient's status at that time.

Patients who decline to continue participation in the study, including telephone contact, should be documented as "withdrawal of consent" rather than "lost to follow-up." Investigators should document attempts to re-establish contact with missing patients throughout the study period. If contact with a missing patient is re-established, the patient should not be considered lost to follow-up and evaluations should resume according to the protocol.

The following actions must be taken if a patient fails to return to the study centre for a required study visit:

- The site must attempt to contact the patient and reschedule the missed visit as soon as possible and counsel the patient on the importance of maintaining the assigned visit schedule and ascertain whether or not the patient wishes to and/or should continue in the study.
- Before a patient is deemed lost to follow-up, the Investigator or designee must make every effort to regain contact with the patient (where possible, 3 telephone calls and, if necessary, a certified letter to the patient's last known mailing address or local equivalent methods). These contact attempts should be documented in the patient's medical record.
- Efforts to reach the patient should continue until the end of the study. Should the patient be unreachable at the end of the study, the patient should be considered to be lost to

follow-up with unknown vital status at the end of study and censored at latest follow-up contact.

Discontinuation of specific sites or of the study as a whole are handled as described in Appendix A 9.

In order to support key efficacy endpoints of EFS, DFS, and OS analyses, the survival status of all patients in the ITT set should be re-checked; this includes those patients who withdrew consent or are classified as "lost to follow-up."

- Potentially lost to follow-up: Site personnel should check hospital records and a publicly available death registry (if available), as well as checking with the patient's current physician, to obtain a current survival status (the applicable eCRF modules will be updated).
- In the event that the patient has actively withdrawn consent to the processing of their personal data, the survival status of the patient can be obtained by site personnel from publicly available death registries (if available) where it is possible to do so under applicable local laws to obtain a current survival status (The applicable eCRF modules will be updated).

8 STUDY ASSESSMENTS AND PROCEDURES

Study procedures and their timing are summarised in the SoA for Screening (Table 1), in the SoAs for each study intervention arm (see relevant appendix), and in the SoA for patients who have completed/discontinued treatment (Table 2). Data collection following study analysis until the end of the study is described below.

- Protocol waivers or exemptions are not allowed.
- Immediate safety concerns should be discussed with the Sponsor immediately upon occurrence or awareness to determine if the patient should continue or discontinue study interventions.
- Adherence to the study design requirements, including those specified in the SoA, is essential and required for study conduct.
- All screening evaluations must be completed and reviewed to confirm that potential patients meet all eligibility criteria. The Investigator will maintain a screening log to record details of all patients screened and to confirm eligibility or record reasons for screening failure, as applicable.
- Procedures conducted as part of the patient's routine clinical management (eg, blood count) and obtained before signing of the ICF may be utilised for screening or baseline

purposes provided the procedures met the protocol-specified criteria and were performed within the time frame defined in the SoA.

- The Investigator will ensure that data are recorded on the eCRFs. An EDC system will be used for data collection and query handling.
- The Investigator ensures the accuracy, completeness, legibility, and timeliness of the data recorded and of the provision of answers to data queries according to the Clinical Study Agreement. The Investigator will sign the completed eCRFs. A copy of the completed eCRFs will be archived at the study site.

Data Collection Following Study Analysis until the End of the Study

After the final DCO and database closure, only SAEs will be reported for the purposes of this study (see Section 8.3.11).

Following the DCO for the final analysis, all patients who remain in the study will continue the scheduled "progression/survival follow-up" site visits indicated in the SoA.

8.1 Efficacy Assessments

Efficacy assessments will be performed and determined by a central BIPR (pCR and mPR), and as determined by Investigator (EFS, DFS, ORR, and OS).

Primary tumours will be assessed by a central pathology reader for the percentage of residual viable tumour that was identified on routine haematoxylin and eosin staining, and patients with a lack of any viable tumour cells after complete evaluation of resected lung cancer specimen(s), including all sampled regional lymph nodes, will be considered to have pCR. Resected lung cancer specimens in which there is ≤ 10% residual viable tumour cells will be considered to have mPR, which is a powered secondary endpoint. Central pathology assessment of pCR and mPR will be performed according to the recommended methods and definitions described by IASLC 2020 (Travis et al, 2020) and by irpCR criteria (Cottrell et al, 2018).

Event-free survival is defined as the time from randomisation to the first of the following: a) documented local or distant recurrence as determined by Investigators using RECIST 1.1 assessments; b) death due to any cause (event date is the date of death); or c) PD that precludes surgery (event date is the date of this determination) or PD discovered and reported by the Investigator upon attempting surgery that prevents completion of surgery (event date is the date of the first attempt at surgery). Patients with R1 and/or R2 margins are not considered to represent PD and will remain in EFS follow-up, although, in the case of R2, they will not be eligible for adjuvant therapy and should be treated in accordance with local SoC. Pathological confirmation from biopsied lesions, if performed according to Investigator's judgement and local practice, will also be taken into consideration (as applicable). All new lesions should be

biopsied where possible. A new primary malignancy confirmed by pathology is not considered an EFS event, but if not confirmed by pathology, it will be considered as an EFS event.

Disease-free survival is defined as the time from the date of surgery until the first date of disease recurrence (local or distant) as determined by Investigators using RECIST 1.1 assessments, or date of death due to any cause, whichever occurs first. Pathological confirmation from biopsied lesions, if performed according to Investigator's judgement and local practice, will also be taken into consideration (as applicable). Definitions of margins are discussed in Section 6.1.3. A new primary malignancy confirmed by pathology is not considered a DFS event, but if not confirmed by pathology, it will be considered as DFS.

Radiological assessment of scans will be according to RECIST 1.1 guidelines (Appendix F). A CT/MRI scan and PET scan should be collected during pre-randomisation screening (CT/MRI scan: Days -28 to -1; PET scan: Days -42 to -1), and post-neoadjuvant treatment/pre-surgery (see Screening Table 1 and the specific study intervention SoAs in the relevant appendix) and CT scan only at Cycle 2. A first post-surgical CT/MRI scan should be collected 5 weeks (± 2 weeks) after surgery and prior to the first dose of adjuvant study interventions (see the specific study intervention SoAs in the relevant appendix). The post-surgical scan should be collected as close as possible to the initiation of first dose of adjuvant study interventions. It is also required that the scan is collected before any PORT, as applicable. It is likely that there will be no evidence of disease (no TLs or NTLs) in the first post-surgical scan, and subsequent scans will be evaluated exclusively for new lesions. Adjuvant period (and beyond) tumour assessments occur according to the schedule outlined in the specific study intervention SoAs in the relevant appendix. If an unscheduled assessment is performed and the patient has not progressed, every attempt should be made to resume subsequent assessments as soon as possible according to the original imaging visit schedule. For patients who discontinue treatment due to toxicity or other reasons in the absence of RECIST 1.1-defined radiological PD, tumour assessments should continue according to the SoA. Following radiological PD, patients do not need to have further scans per protocol since, in these patients an EFS event has occurred. Patients who do not reach surgery for any reason become ineligible for DFS.

8.1.1 Central Imaging

Images, including unscheduled visit scans, will be collected on an ongoing basis and sent to an AstraZeneca-appointed imaging CRO for quality control and storage. Guidelines for image acquisition, de-identification, storage at the investigative site as source data, and transfer to the imaging CRO will be provided in a separate document. Electronic image transfer from the sites to the imaging CRO is strongly encouraged.

8.1.2 Overall Survival

All patients including those who have disease progression or have discontinued treatment will be followed for survival until death or overall study completion as described in the SoA

(Table 2).

8.2 Safety Assessments

Planned time points for all safety assessments are provided in the SoAs for Screening (Table 1); in the specific study intervention SoAs in the relevant appendix; and in the SoA for patients who complete/discontinue treatment (Table 2).

8.2.1 Clinical Safety Laboratory Assessments

Blood and urine samples for determination of clinical chemistry, haematology, and urinalysis will be taken at the times indicated in the assessment schedules and as clinically indicated (see the SoAs for Screening [Table 1], the specific study intervention SoAs in the relevant appendix; and the SoA for patients who complete/discontinue treatment [Table 2]).

Clinical laboratory safety tests, including serum pregnancy tests, will be performed in a licensed clinical laboratory according to local standard procedures. The results must be promptly reviewed by the Investigator. Sample tubes and sample sizes may vary depending on the laboratory method used and routine practice at the site. Pregnancy tests may be performed at the site using a licensed test (urine or serum pregnancy test). Abnormal clinically significant laboratory results should be repeated as soon as possible (preferably within 24 to 48 hours).

Additional safety samples may be collected if clinically indicated at the discretion of the Investigator.

The date, time of collection, and results (values, units, and reference ranges) will be recorded on the appropriate eCRF.

The laboratory variables to be measured are presented in Table 7 (clinical chemistry), Table 8 (haematology), Table 9 (coagulation), and Table 10 (urinalysis).

Other safety tests to be performed at screening include assessment for hepatitis B surface antigen, hepatitis C antibodies, and pregnancy tests. If Arm 4, 6, or 7 are open for enrolment, all participants must be tested for HIV during the screening period if acceptable by local regulations or an IRB/EC.

The following laboratory variables will be measured:

Table 7 Clinical Chemistry

Albumin	Gamma glutamyl transferase [b, f]
Alkaline phosphatase [a, b]	Glucose
	Glycated haemoglobin [i]
ALT [a, b]	Lactate dehydrogenase [b]

Amylase [d]	Lipase [d]
Anti-thyroid antibodies (anti-TPOAb, TRAb, TgAb) [e]	Magnesium [f]
	Potassium
	Sodium
AST [a, b]	Total bilirubin [a, b]
Bicarbonate [f]	Total cholesterol
Calcium	Total protein
Chloride [f]	TSH [j, k]
Cortisol [g]	T3 free [k] (reflex) or Total T3 [k]
C-Reactive protein	T4 free [k] (reflex)
Creatinine	Troponin I or T [c]
Creatinine CL [f, h]	Urea or blood urea nitrogen, depending on local practice
Creatinine phosphokinase [c]	
Ferritin [c]	

a Tests for ALT, AST, alkaline phosphatase, and total bilirubin must be conducted and assessed concurrently. If total bilirubin is ≥ 2 × ULN (and no evidence of Gilbert's syndrome), then fractionate into direct and indirect bilirubin.

b ALT, AST, total bilirubin, ALP, GGT, and LDH will be measured at Screening for all patients CCI [redacted]

c CCI [redacted]

d It is preferable that both amylase and lipase parameters are assessed. For sites where only 1 of these parameters is routinely measured, either lipase or amylase is acceptable. CCI [redacted]

e Measured on all patients at Screening visit and may be measured as clinically indicated.

f Bicarbonate (where available), chloride, creatinine CL, gamma glutamyl transferase, and magnesium testing are to be performed at Screening, Cycle 1 Day 1 of neoadjuvant and adjuvant treatment periods (unless all screening laboratory clinical chemistry assessments are performed within 3 days prior to Day 1), and as clinically indicated.

g Cortisol testing should be obtained at screening and at the pre-surgery visit.

h Creatinine CL will be calculated using Cockcroft-Gault (using actual body weight).

i Glycated haemoglobin will be measured at Screening and as clinically indicated during the study. CCI [redacted]

j If TSH is measured within 14 days prior to Day 1 (first infusion day), it does not need to be repeated on Day 1.

k Free T3 or Total T3, or free T4 will only be measured if TSH is abnormal or if there is a clinical suspicion of an AE related to the endocrine system.

Abbreviations: AE: Adverse event; ALP: alkaline phosphatase; ALT: Alanine aminotransferase; AST: Aspartate aminotransferase; CL: Clearance; EOT: end of treatment; GGT: gamma glutamyl transferase; LDH: lactate dehydrogenase; T3: Triiodothyronine; T4: Thyroxine; TgAb: Thyroglobulin antibodies; TPOAb: Anti–thyroid peroxidase antibody; TRAb: TSH receptor antibodies; TSH: Thyroid-stimulating hormone; ULN: Upper limit of normal.

Table 8 Haematology

Absolute eosinophil count[a]	Haematocrit
Absolute lymphocyte count[a]	Haemoglobin
Absolute neutrophil count[a]	Platelet count
Red blood cell count[a]	Total white cell count

[a] Can be recorded as absolute counts or as percentages. Absolute counts will be calculated if entered as percentages. Total white cell count therefore has to be provided.

Table 9 Coagulation

aPTT or PTT[a]	International normalised ratio

[a] Either as a ratio or as an absolute value, in seconds.
Note: Coagulation parameters to be performed at screening, as clinically indicated during neoadjuvant treatment, and within 30 days before surgery.
Abbreviations: aPTT: Activated partial thromboplastin time; PTT: Partial thromboplastin time.

Table 10 Urinalysis

Bilirubin	Ketones
Blood	Protein
Glucose	White blood cells

Note: Urinalysis should be done at baseline (screening), Day 1 of adjuvant treatment, and as clinically indicated.

If a patient shows an AST or ALT ≥ 3 × ULN together with total bilirubin ≥ 2 × ULN, refer to Appendix E for further instructions on cases of increases in liver biochemistry and evaluation of Hy's law. These cases should be reported as SAEs if, after evaluation, they meet the criteria for a Hy's law case or if any of the individual liver test parameters fulfil any of the SAE criteria.

All patients should have further chemistry profiles performed at 30 days (± 3 days), 2 months (± 1 week), and 3 months (± 1 week) after permanent discontinuation of study interventions (Table 2).

Any clinically significant abnormal laboratory values should be repeated as clinically indicated and recorded on the eCRF. Situations in which laboratory safety results should be reported as AEs are described in Section 8.3.5.

Management of significant abnormal cortisol levels should follow the institutional guidelines or the Investigators' or Surgeon's clinical judgement.

All patients with Grade 3 or 4 laboratory values at the time of completion or discontinuation from study interventions must have further tests performed until the laboratory values have returned to Grade 1 or 2, unless these values are not likely to improve because of the

underlying disease.

8.2.2 Physical Examinations

Physical examinations will be performed according to the SoAs (refer to Table 1 for Screening; refer to the specific study intervention SoAs in the relevant appendix; and refer to the SoA for patients who complete/discontinue treatment (Table 2). Full physical examinations will include assessments of the head, eyes, ears, nose, mouth, and throat and the respiratory, cardiovascular, gastrointestinal, urogenital, musculoskeletal, neurological, dermatological, hematologic/lymphatic, and endocrine systems. Height will be measured at screening only. Targeted physical examinations are to be utilised by the Investigator on the basis of clinical observations and symptomatology. Situations in which physical examination results should be reported as AEs are detailed in Section 8.3.4.

8.2.3 Vital Signs

Vital signs (BP, pulse, temperature, SpO_2 and respiration rate) will be evaluated according to the SoAs: refer to Table 1 for Screening; refer to the specific study intervention SoAs in the relevant appendix; and refer to the SoA for patients who complete/discontinue treatment (Table 2). Body WT is also recorded at each visit along with vital signs.

First infusion of study interventions

On the first infusion day, patients will be monitored, and vital signs will be collected/recorded in eCRF prior to, during, and after infusion of study interventions as presented in the bulleted list below.

Blood pressure and pulse will be collected from patients before, during, and after each infusion at the following times (based on a 60-minute infusion):

- Prior to the beginning of the infusion (measured once from approximately 30 minutes before up to 0 minutes [ie, the beginning of the infusion]).
- Approximately 30 minutes after start of the infusion (**halfway** through infusion).
- At the end of the infusion (approximately 60 minutes ± 10 minutes after start of the infusion).

Note: First infusion collection guidance applies to the 1st administered IMP only, and does not include chemotherapy or durvalumab.

If the infusion takes longer than 60 minutes, then BP and pulse measurements should follow the principles as described above or be taken more frequently if clinically indicated.

Subsequent infusions of study interventions

Blood pressure, pulse, and other vital signs should be measured and collected/recorded in eCRF prior to the start of the infusion. Patients should be carefully monitored, and BP and other vital signs should be measured during and post infusion as per institution standard and as clinically indicated. Any clinically significant changes in vital signs should be entered onto an unscheduled vital signs eCRF page.

On days where chemotherapy is administered without study interventions, patients in the chemotherapy will be monitored pre-dose and as clinically indicated before every infusion or administration. Situations in which vital signs results should be reported as AEs are described in Section 8.3.5. For any AEs of infusion reactions, the vital signs values should be entered into the eCRF.

8.2.4 Electrocardiograms

Resting 12-lead ECGs will be recorded at screening and as clinically indicated throughout the study: refer to Table 1 for Screening; refer to the specific study intervention SoAs in the relevant appendix; and refer to the SoA for patients who complete/discontinue treatment (Table 2). Electrocardiograms should be obtained after the patient has been in a supine position for 5 minutes and recorded while the patient remains in that position.

In case of clinically significant ECG abnormalities, including a QTcF value ≥ 470 ms, 2 additional 12-lead ECGs should be obtained over a brief period (eg, 30 minutes) to confirm the finding.

Situations in which ECG results should be reported as AEs are described in Section 8.3.5.

8.2.5 Echocardiogram/MUGA

At the visits specified in the SoA (refer to Table 1 for Screening; refer to the specific study intervention SoAs in the relevant appendix; and refer to the SoA for patients who complete/discontinue treatment [Table 2]) echocardiograms or MUGAs will be performed to assess LVEF. Additional echocardiogram/MUGA may be performed as needed at the discretion of the investigator or designee if clinically indicated (eg, myocarditis or any myocardial imAE). Note that if a patient requires an on-study MUGA scan, in order to ensure that radioactivity levels from the radiotracer have decayed to exempt levels before receipt of samples at the central laboratory, any blood samples for flow cytometry should be collected either before the MUGA scan or at least 2 days after the MUGA scan has been performed.

Note that ECHO/MUGA assessments are only required at Screening if Arms 3A, 3B,3C, 6 or 7 are open for enrolment.

8.2.6 WHO/ECOG Performance Status

WHO/ECOG PS will be assessed at the times specified in the assessment schedules (see the SoAs: refer to Table 1 for Screening; refer to the specific study intervention SoAs in the relevant appendix; and refer to Table 2 for patients who complete/discontinue treatment) based on the following:

Table 11 WHO/ECOG Performance Status

Performance Status	Assessment Criteria
0	Fully active; able to carry out all usual activities without restrictions
1	Restricted in strenuous activity but ambulatory and able to carry out light work or work of a sedentary nature (eg, light housework or office work)
2	Ambulatory and capable of self-care but unable to carry out any work activities; up and about more than 50% of waking hours
3	Capable of only limited self-care; confined to bed or chair more than 50% of waking hours
4	Completely disabled; unable to carry out any self-care and totally confined to bed or chair
5	Dead

Any significant change from baseline or screening must be reported as an AE.

8.2.7 Pulmonary Function Assessment

As all patients in this study need to undergo surgery, the recommendations of the ESMO for pre-treatment risk assessment should be followed. Formal lung function testing should therefore be undertaken at screening to assess suitability for surgery. A pulmonary function test done as part of clinical practice within 42 days prior to randomisation is acceptable and does not need to be repeated during screening.

For patients with FEV_1 and DLCO values > 80% of their predicted pulmonary function tests and no other major comorbidities at screening, no further investigations are advised before surgical resection. For others, exercise testing and split lung function are recommended. In these patients, maximal oxygen consumption can be used to measure exercise capacity and predict postoperative complications. Surgical resection is usually acceptable if the predicted postoperative pre- or post-bronchodilator FEV_1 of 1.0 L and DLCO values are > 40%. If clinically indicated, pulmonary function tests should be repeated prior to surgery at the discretion of the Investigator.

8.2.8 Ophthalmological Assessments

Ophthalmologic assessments including but not limited to, visual acuity testing, slit lamp examination, intraocular pressure measurement, fundoscopy, and fluorescein staining will be

performed for all patients at screening and for patients randomised to Dato-DXd as clinically indicated and at the EOT visit by an ophthalmologist, or if unavailable, another licensed eye care provider (see SoAs for Screening [Table 1], the specific study intervention SoA in the relevant appendix for Dato-DXd; and the SoA for patients who complete/discontinue treatment [Table 2]). A suitable alternative to fluorescein staining of the cornea may be used in exceptional circumstances where fluorescein is not available.

Patients receiving Dato-DXd should be advised to use artificial tears 4 times daily as a preventative measure and up to 8 times daily as clinically needed and to avoid the use of contact lenses. The use of other eye medications (eg, topical corticosteroids) for prophylaxis should be at the discretion of an ophthalmologist, or if unavailable, another licensed eye care provider.

Note that ophthalmological assessments are only required at Screening if Arm 4 or 7, containing Dato-DXd, is open for enrolment.

Please refer to the Dato-DXd Ophthalmologic Assessment Manual for further details.

8.2.9 Other Safety Assessments

If new or worsening pulmonary symptoms (eg, dyspnoea) or radiological abnormality suggestive of pneumonitis/ILD are observed, toxicity management as described in detail in the TMGs (see Section 8.3.15) will be applied. The results of the full diagnostic workup (including, but not limited to, HRCT, blood and sputum culture, and haematological parameters) will be captured in the eCRF. It is strongly recommended to perform a full diagnostic workup to exclude alternative causes such as lymphangitic carcinomatosis, infection, allergy, cardiogenic oedema, or pulmonary haemorrhage. In the presence of confirmatory HRCT scans where other causes of respiratory symptoms have been excluded, a diagnosis of pneumonitis/ILD should be considered, and the TMGs should be followed. Troponin measurements will be performed to rule out cardiac aetiology.

Pneumonitis (ILD) investigation

The following assessments, and additional assessments if required, will be performed to enhance the investigation and diagnosis of potential cases of pneumonitis. The results of the assessment will be collected.

- Physical examination
  - Signs and symptoms (eg, cough, shortness of breath, and pyrexia) including auscultation for lung field will be assessed.
- Saturation of peripheral oxygen (SpO2)
  - SpO2.

- Other items
  - When pneumonitis (ILD) is suspected during study treatment, the following markers should be measured where possible:
    (i) ILD Markers (KL-6, SP-D) and β-D-glucan.
    (ii) Additional clinical chemistry: C-reactive protein and lactate dehydrogenase.
  - HRCT of the chest if feasible (non-contrast chest CT is acceptable).

Brain MRI/CT

At screening, a brain MRI (preferred) or brain CT with IV contrast will be performed.

8.3 Adverse Events and Serious Adverse Events

The Investigator is responsible for ensuring that all staff involved in the study are familiar with the content of this section.

The definitions of an AE or SAE can be found in Appendix B.

Adverse events will be reported by the patient (or, when appropriate, by a caregiver, surrogate, or the patient's legally authorised representative).

The Investigator and any designees are responsible for detecting, documenting, recording, and reporting events that meet the definition of an AE. For information on how to follow/up AEs, see Section 8.3.2, and the Clavien-Dindo Classification of surgical complications is found in Appendix I.

8.3.1 Time Period and Frequency for Collecting AE and SAE Information

Adverse events and SAEs will be collected from the time of signature of the ICF, throughout the treatment period and until the safety follow-up period is completed (90 days after the last dose of study interventions [ie, the safety follow-up visit]). If an event that starts post the defined safety follow-up period noted above is considered to be due to a late onset toxicity to study intervention, then it should be reported as an AE or SAE as applicable. Collection and reporting of AEs and SAEs after the final DCO is described in Section 8.3.11.

All SAEs will be recorded and reported to the Sponsor or designee within 24 hours, as indicated in Appendix B. The Investigator will submit any updated SAE data to the Sponsor within 24 hours of it being available.

Investigators are not obligated to actively seek AE or SAE in former study patients. However, if the Investigator learns of any SAE, including a death, at any time after a patient's last visit and he/she considers the event to be reasonably related to the study interventions or study participation, the Investigator should notify the Sponsor.

The method of recording, evaluating, and assessing causality of AE and SAE and the procedures for completing and transmitting SAE reports are provided in Appendix B.

8.3.2 Follow-up of AEs and SAEs

After the initial AE/SAE report, the Investigator is required to proactively follow each patient at subsequent visits/contacts. All events will be followed until resolution, stabilisation, the event is otherwise explained, or the patient is lost to follow-up.

Any AEs that are unresolved at patient's last safety follow-up visit in the study are followed up by the Investigator for as long as medically indicated (this may be beyond the 90 days after the last dose of study interventions), but without further recording in the eCRF. AstraZeneca retains the right to request additional information for any patient with ongoing AE(s)/SAE(s) at the end of the study, if judged necessary.

Adverse event variables

The following variables will be collected for each AE:

- AE (verbatim).
- The date when the AE started and stopped.
- The CTCAE grade reported.
- Changes in CTCAE grade (report only the maximum CTCAE grade for a calendar day).
- Whether the AE is serious or not (Appendix B).
- Investigator causality rating against the study interventions (yes or no).
- Action taken with regard to study interventions.
- Administration of treatment for the AE.
- Outcome.

In addition, the following variables will be collected for SAEs:

- Date AE met criteria for SAE.
- Date Investigator became aware of SAE.
- Seriousness criteria.
- Date of hospitalisation.
- Date of discharge.
- Probable cause of death.
- Date of death.
- Whether an autopsy was performed.

- Causality assessment in relation to study procedure(s).
- Causality assessment to other medication.
- Description of the SAE.

The grading scales found in the NCI CTCAE 5.0 will be utilised for all events with an assigned CTCAE grading. For those events without assigned CTCAE grades, the recommendation in the CTCAE criteria that converts mild, moderate, and severe events into CTCAE grades should be used. A copy of the CTCAE 5.0 can be downloaded from the Cancer Therapy Evaluation Programme website (http://ctep.cancer.gov).

8.3.3 Causality Collection

The Investigator should assess causal relationship between study interventions and each AE, and answer "yes" or "no" to the question "Do you consider that there is a reasonable possibility that the event may have been caused by the investigational product?"

The Investigator will assess causal relationship between each AE that occurs after surgery, and surgery, and answer "yes" or "no" to the question "Do you consider that there is a reasonable possibility that the event may have been caused by the surgery?"

The Investigator will assess causal relationship between each AE that occurs after PORT, and answer "yes" or "no" to the question "Do you consider that there is a reasonable possibility that the event may have been caused by the PORT?"

For SAEs, causal relationship should also be assessed for other medication and study procedures. Note that for SAEs that could be associated with any study procedure the causal relationship is implied as "yes".

A guide to the interpretation of the causality question is provided in Appendix B.

8.3.4 Adverse Events Based on Signs and Symptoms

All AEs spontaneously reported by the patient or reported in response to the open question from the study site staff: "Have you had any health problems since the previous visit/you were last asked?", or revealed by observation will be collected and recorded in the eCRF. When collecting AEs, the recording of diagnoses is preferred (when possible) to recording a list of signs and symptoms. However, if a diagnosis is known and there are other signs or symptoms that are not generally part of the diagnosis, the diagnosis and each sign or symptom will be recorded separately.

8.3.5 Adverse Events Based on Examinations and Tests

The results from the CSP-mandated laboratory tests, vital signs, physical examinations, and ECGs will be summarised in the CSR.

Deterioration as compared with baseline in protocol-mandated laboratory values and vital signs should therefore only be reported as AEs if they fulfil any of the SAE criteria, are the reason for discontinuation of treatment with the study interventions or are considered to be clinically relevant as judged by the Investigator (which may include but not limited to consideration as to whether treatment or non-planned visits were required or other action was taken with the study interventions, eg, study interventions interruption).

If deterioration in a laboratory value/vital sign is associated with clinical signs and symptoms, the sign or symptom will be reported as an AE and the associated laboratory result/vital sign will be considered as additional information. Wherever possible the reporting Investigator uses the clinical, rather than the laboratory term (eg, anaemia versus low haemoglobin value). In the absence of clinical signs or symptoms, clinically relevant deteriorations in non-mandated parameters should be reported as AE(s).

Deterioration of a laboratory value, which is unequivocally due to PD, should not be reported as an AE/SAE.

Any new or aggravated clinically relevant abnormal medical finding at a physical examination as compared with the baseline assessment will be reported as an AE unless unequivocally related to the disease under study.

8.3.6 Hy's Law

Cases where a patient shows elevations in liver biochemistry may require further evaluation and occurrences of AST or ALT ≥ 3 × ULN together with TBL ≥ 2 × ULN may need to be reported as SAEs. Please refer to Appendix E for further instruction on cases of increases in liver biochemistry and evaluation of HL.

8.3.7 Disease Progression

Disease progression can be considered as a worsening of a patient's condition attributable to the disease for which the study interventions are being studied. It may be an increase in the severity of the disease under study and/or increases in the symptoms of the disease. The development of NLs, or progression of existing lesions of the primary cancer under study, should be considered as PD and not an AE. Events, which are unequivocally due to PD, should not be reported as an AE during the study.

8.3.8 New Cancers

The development of a new cancer should be regarded as an SAE. New primary cancers are those that are not the primary reason for the administration of study interventions and are identified after the patient's inclusion in this study. They do not include metastases of the original cancer.

8.3.9 Deaths

All deaths that occur during the study treatment period, or within the protocol-defined follow-up period after the administration of the last dose of study interventions, must be reported as follows:

- Death clearly resulting from PD should be documented in the eCRF in the Statement of Death page and reported to the Study Monitor/Physician at the next monitoring visit. It should not be reported as an SAE.
- Where death is not due (or not clearly due) to PD under study, the AE causing the death must be reported to the Study Monitor/Physician as an SAE within 24 hours. It should also be documented in the Statement of Death page in the eCRF. The report should contain a comment regarding the co-involvement of PD, if appropriate, and should assign the main and contributory causes of death.
- Deaths with an unknown cause should always be reported as an SAE and documented in the Statement of Death page in the eCRF, but every effort should be made to determine a cause of death. A post-mortem may be helpful in the assessment of the cause of death, and if performed, a copy of the post-mortem results should be forwarded to AstraZeneca Patient Safety or its representative within the usual time frames.

Deaths occurring after the protocol-defined follow-up period after the administration of the last dose of study interventions should be documented in the Statement of Death page. If the death occurred as a result of an event that started after the defined follow-up period and the event is considered to be due to a late-onset toxicity to study interventions, then it should also be reported as an SAE.

8.3.10 Adverse Events of Special Interest

An adverse event of special interest (AESI) is one of scientific and medical interest specific to understanding the study interventions and may require close monitoring. An AESI may be serious or non-serious, and these events should be reported in the eCRF regardless of causality. The rapid reporting of AESIs allows ongoing surveillance of these events in order to characterise and understand them in association with the use of the study interventions.

All AESIs, regardless of severity or seriousness, must be followed until either event resolution, end of study, trial termination, withdrawal of consent, or patient death.

8.3.10.1 Adverse Events of Special Interest for Durvalumab

AESIs for durvalumab include but are not limited to events with a potential inflammatory or immune-mediated mechanism and which may require more frequent monitoring and/or interventions such as steroids, immunosuppressants, and/or hormone replacement therapy.

These AESIs are being closely monitored in clinical studies with durvalumab monotherapy and combination therapy. An imAE is defined as an AE that is associated with drug exposure and is consistent with an immune-mediated mechanism of action and where there is no clear alternate aetiology. Serologic, immunologic, and histologic (biopsy) data, as appropriate, should be used to support an imAE diagnosis. Appropriate efforts should be made to rule out neoplastic, infectious, metabolic, toxin, or other aetiologic causes of the imAE.

If the Investigator has any questions determining whether an AE is an imAE, the Investigator should promptly contact the Study Physician/Medical Scientist. AESIs observed with durvalumab include:

- Diarrhoea/colitis and intestinal perforations
- Pneumonitis
- Hepatitis
- Endocrinopathies (ie, events of hypophysitis/hypopituitarism, thyroiditis, type 1 diabetes mellitus, adrenal insufficiency, and hyper- and hypothyroidism)
- Rash/dermatitis
- Nephritis
- Pancreatitis
- Myocarditis
- Myositis/polymyositis
- Immune complex disease. The immune system can respond to foreign mAbs by producing human-anti-human antibodies, which may result in formation of immune complexes and their deposition in blood vessels, joints, and glomeruli causing symptomatic disease (eg, vasculitis, glomerulonephritis, arthritis, serum sickness). Patients who experience an AE suspected to be immune-complex related and with confirmed presence of ADAs will discontinue treatment. Immune-complex disease will be managed in accordance with SoC.
- Other inflammatory responses that are rare/less frequent with a potential immune-mediated aetiology include, but are not limited to, haematological event, neuromuscular toxicities (such as myasthenia gravis and Guillain-Barré syndrome), non-infectious encephalitis, non-infectious meningitis, pericarditis, rheumatological events, sarcoidosis, skin events, uveitis (and other events involving the eye), vasculitis, and psoriasis.

In addition, IRRs and hypersensitivity/anaphylactic reactions with a different underlying pharmacological aetiology are also considered AESIs. Infusion of biological products is commonly associated with IRRs. Anaphylaxis and IRRs have some common manifestations

and may be difficult to distinguish from each other. IRRs are commonly observed during or shortly after the first time of exposure to therapeutic mAbs delivered through IV infusion. These reactions are less common following subsequent exposures. Unlike IRRs, anaphylaxis is a rare event, usually occurring after subsequent exposure to an antigen, and it is most commonly accompanied by severe systemic, skin and/or mucosal reactions. The Investigator is advised to carefully examine symptoms of adverse reactions observed during or shortly after exposure to study interventions and consider the above-mentioned facts prior to making a final diagnosis. The Investigators may refer to NIAID and FAAN guidance (Sampson et al, 2006) for anaphylaxis diagnosis to facilitate consistency in judgements.

Further information on these risks (eg, presenting symptoms) can be found in the current version of the durvalumab IB. More specific guidelines for their evaluation and treatment are described in detail in the TMGs (Section 8.3.15).

8.3.10.2 Adverse Events of Special Interest Associated with Investigational Products

Refer to the specific appendices for AESIs associated with investigational products. See Appendix K 7 for oleclumab, Appendix L 7 for monalizumab, Appendix M 8 for volrustomig, Appendix N 7 and Appendix Q 7.2 for Dato-DXd, Appendix O 8 for AZD0171, and Appendix P 7 and Appendix Q 7.1 for rilvegostomig.

8.3.11 Safety Data to be Collected Following the Final Data Cut-off of the Study

For patients continuing to receive the study interventions after the final DCO, AEs and SAEs will be collected, but only SAEs will be reported. In addition, it is recommended that Investigators monitor the patient's safety laboratory results periodically during treatment with study interventions in order to manage AEs, consistent with the TMGs (see Section 8.3.15). All data after the final DCO and database closure will be recorded in the patient notes but, with the exception of SAEs, will not otherwise be reported for the purposes of this study.

8.3.12 Reporting of Serious Adverse Events

All SAEs have to be reported, whether or not considered causally related to the study interventions, or to the study procedure(s). All SAEs will be recorded in the eCRF.

All SAEs that occur in patients within the 90 days following the last dose of study interventions after the final DCO must be reported as detailed in Section 8.3.11.

If any SAE occurs in the course of the study, then Investigators or other site personnel inform the appropriate AstraZeneca representatives within 1 day, ie, immediately but **no later than 24 hours** of when he or she becomes aware of it.

The designated AstraZeneca representative works with the investigator to ensure that all the necessary information is provided to the AstraZeneca Patient Safety data entry site **within**

1 calendar day of initial receipt for fatal and life-threatening events **and within 5 calendar days** of initial receipt for all other SAEs.

For fatal or life-threatening AEs where important or relevant information is missing, active follow-up is undertaken immediately. Investigators or other site personnel inform AstraZeneca representatives of any follow-up information on a previously reported SAE within 1 calendar day ie, immediately but **no later than 24 hours** of when he or she becomes aware of it.

Once the Investigators or other site personnel indicate an AE is serious in the EDC system, an automated email alert is sent to the designated AstraZeneca representative.

If the EDC system is not available, then the Investigator or other study site staff reports a SAE to the appropriate AstraZeneca representative by email.

The AstraZeneca representative will advise the Investigator/study site staff how to proceed.

For further guidance on the definition of a SAE, see Appendix B.

The reference document for definition of expectedness/listedness is the IB for the AstraZeneca drug and local prescribing information for chemotherapy.

8.3.13 Pregnancy

All pregnancies and outcomes of pregnancy with conception dates following the first date of study interventions, including pregnancy in the partner of male patients, except if the pregnancy is discovered before the study patient has received any study intervention, should be reported to AstraZeneca.

If a pregnancy is reported, the Investigator should inform the Sponsor within 24 hours of learning of the pregnancy.

Abnormal pregnancy outcomes (eg, spontaneous abortion, foetal death, stillbirth, congenital anomalies, and ectopic pregnancy) are considered SAEs.

8.3.13.1 Maternal Exposure

If a patient becomes pregnant during the course of the study, study interventions should be discontinued immediately.

Pregnancy itself is not regarded as an AE unless there is a suspicion that the study interventions may have interfered with the effectiveness of a contraceptive medication. Congenital abnormalities/birth defects and spontaneous miscarriages should be reported and handled as SAEs. Elective abortions without complications should not be handled as AEs. The outcome of all pregnancies (spontaneous miscarriage, elective termination, ectopic pregnancy,

normal birth or congenital abnormality) should be followed up and documented even if the patient was discontinued from the study.

If any pregnancy occurs in the course of the study, then the Investigator or other site personnel informs the appropriate AstraZeneca representatives within 1 day, ie, immediately but no later than 24 hours of when he or she becomes aware of it.

The designated AstraZeneca representative works with the Investigator to ensure that all relevant information is provided to the AstraZeneca Patient Safety data entry site within 1 or 5 calendar days for SAEs (see Section 8.3.12) and within 30 days for all other pregnancies.

The same timelines apply when outcome information is available.

The PREGREP module in the eCRF is used to report the pregnancy and the PREGOUT is used to report the outcome of the pregnancy.

8.3.13.2 Paternal Exposure

Non-sterilised male patients who intend to be sexually active with a female partner of childbearing potential should refrain from fathering a child or donating or banking sperm for the duration of the study (from the time of screening) and for the following period after the last dose of study interventions:

- Durvalumab or volrustomig: 90 days
- Oleclumab, monalizumab, or AZD0171: 180 days
- Dato-DXd: 120 days
- Rilvegostomig: 60 days

Follow the local prescribing information relating to contraception and the time limit for such precautions for chemotherapy agents, and any additional restrictions for the agents administered. For patients receiving more than one study intervention, the longest washout period must be followed.

Pregnancy of the patient's partner is not considered to be an AE. However, the outcome of all pregnancies (spontaneous miscarriage, elective termination, ectopic pregnancy, normal birth, or congenital abnormality) occurring from the date of the first dose of study interventions until the following period after receiving the last dose of study interventions:

- Durvalumab or volrustomig: 90 days
- Oleclumab, monalizumab, or AZD0171: 180 days
- Dato-DXd: 120 days
- Rilvegostomig: 60 days

For the chemotherapy agents follow the local prescribing information relating to contraception, the time limits for such precautions, and any additional restrictions for the agents administered.

For patients receiving more than one study intervention, the longest period will apply.

Pregnancies should be followed up and documented in the medical record and provided to the AstraZeneca Patient Safety data entry site. Consent from the partner must be obtained before the information is collected and reported to AstraZeneca.

Where a report of pregnancy is received, prior to obtaining information about the pregnancy, the Investigator must obtain the consent of the patient's partner. The local study team should adopt the Master Pregnant Partner Form in line with local procedures/requirements and submit it to the relevant Regulatory Authority/IRBs/IECs prior to use.

Patients who are permanently discontinued from further receipt of study interventions, regardless of the reason, will be identified as having permanently discontinued treatment and will enter follow-up (see Table 2).

8.3.14 Medication Error, Drug Abuse, and Drug Misuse

8.3.14.1 Timelines

If an event of medication error, drug abuse, or drug misuse occurs during the study, then the Investigator or other site personnel informs the appropriate AstraZeneca representatives within 1 calendar day, ie, immediately but no later than 24 hours of when they become aware of it.

The designated AstraZeneca representative works with the Investigator to ensure that all relevant information is completed within 1 (initial fatal/life-threatening or follow-up fatal/life-threatening) or 5 (other serious initial and follow-up) calendar days if there is an SAE associated with the event of medication error, drug abuse, or misuse (see Section 8.3.12) and within 30 days for all other events.

8.3.14.2 Medication Error

For the purposes of this clinical study a medication error is an unintended failure or mistake in the treatment process for an IMP/study intervention or AstraZeneca NIMP that either causes harm to the patient or has the potential to cause harm to the patient.

The full definition and examples of a medication error can be found in Appendix B 4.

8.3.14.3 Drug abuse

Drug abuse is the persistent or sporadic intentional, non-therapeutic excessive use of IMP/study intervention or AstraZeneca NIMP for a perceived reward or desired non

therapeutic effect.

The full definition and examples of drug abuse can be found in Appendix B 4.

8.3.14.4 Drug Misuse

Drug abuse is the intentional and inappropriate use (by a study participant) of IMP/study intervention or AstraZeneca NIMP for medicinal purposes outside of the authorised product information, or for unauthorised IMPs/study intervention(s) or AstraZeneca NIMPs, outside the intended use as specified in the protocol and includes deliberate administration of the product by the wrong route.

The full definition and examples of Drug Misuse can be found in Appendix B 4.

8.3.15 Toxicity Management Guidelines

The following general guidance should be followed for management of toxicities.

- Treat each of the toxicities with maximum supportive care (including holding the agent suspected of causing the toxicity if required).
- If the symptoms promptly resolve with supportive care, consideration should be given to continuing the same dose of the assigned study interventions along with appropriate continuing supportive care.
- Patients should be thoroughly evaluated to rule out any alternative aetiology (eg, disease progression, concomitant medications, or infections). This includes chemotherapy-induced toxicity.
- In the absence of clear alternative aetiology, all events should be considered potentially immune mediated.
- In the event that an agent(s) (chemotherapy, durvalumab, oleclumab, monalizumab, volrustomig, Dato-DXd, AZD0171, or rilvegostomig) is held due to treatment-related toxicity, the other agent(s) may still be administered at the Investigator's discretion when toxicity resolves to Grade 2 or less, unless agent-specific TMGs require permanent discontinuation or have additional requirements for starting treatment. Note: if the Investigator feels that a patient is ready to restart treatment prior to the toxicity resolving to Grade 2 or less, the AstraZeneca study physician or medical scientist should be consulted for an exception to this rule. If chemotherapy is permanently discontinued due to treatment-related toxicity, study interventions may continue at the Investigator's discretion, and AstraZeneca should be consulted.
- The specific TMGs for each study intervention are held in the site TMF.
- Comprehensive TMGs have been developed to assist Investigators with the recognition and management of toxicities associated with the use of investigational products. For TEAEs that are deemed non-drug related or are not included in the TMGs, follow

standard clinical practice. Appropriate clinical experts should be consulted as deemed necessary. The TMGs for durvalumab are applicable when durvalumab is used alone or in combination with other anti-cancer drugs (ie, antineoplastic chemotherapy, targeted agents) administered concurrently or sequentially as part of a protocol-specific treatment regimen. The TMGs provide information for the management of immune-mediated reactions, IRRs, and non-immune-mediated reactions that may be observed with checkpoint inhibitor monotherapy or combination checkpoint inhibitor regimens, with specific instructions for dose modifications (including discontinuations) and treatment interventions. Investigators are advised however to use local practice guidelines and consult local references for the management of toxicities observed with other cancer treatment.

If unsure how to manage a patient, contact the Study Physician/Medical Scientist to discuss individual cases. Treatment for toxicities should be initiated prior to discussion with the Study Physician/Medical Scientist.

All toxicities will be graded according to NCI CTCAE version 5.0.

8.3.15.1 Specific Toxicity Management and Dose Modification Information – Durvalumab, Monalizumab, Oleclumab, AZD0171, Volrustomig, and Rilvegostomig

For Durvalumab and monalizumab, refer to the most current version of the durvalumab TMGs; for oleclumab, volrustomig, AZD0171, and rilvegostomig, refer to the most current version of their respective TMGs. The investigator should use clinical judgement when attributing a toxicity to agents in the combination. The choice of the TMGs (see the Annex documents to this CSP and the local label or national guidance for chemotherapy) should be based on the investigator's assessment of the causal role of the combination agents. All TMGs are provided to the investigative site as Annex document(s) and are maintained within the Site Master File.

In situations where the assessment qualifies multiple agents' TMGs for a particular toxicity, the more conservative TMG should be applied.

Patients should be thoroughly evaluated, and appropriate efforts should be made to rule out neoplastic, infectious, metabolic, toxin, or other aetiologic causes of the imAE. Serologic, immunologic, and histologic (biopsy) data, as appropriate, should be used to support an imAE diagnosis. In the absence of a clear alternative aetiology, events should be considered potentially immune related. In addition, there are certain circumstances in which study interventions should be permanently discontinued (see Section 7.1). Dose reductions are not permitted. In case of doubt, the Investigator should consult with the Study Physician/Medical Scientist.

8.3.15.2 Specific Toxicity Management and Dose Modification Information – Dato-DXd

For Dato-DXd, investigators should consult the TMGs. The investigator should use clinical judgement when attributing a toxicity to agents in the combination. The choice of the TMGs (see the Annex documents to this CSP for Dato-DXd and durvalumab, and the local label or national guidance for chemotherapy) should be based on the investigator's assessment of the causal role of the combination agents. All TMGs are provided to the investigative site as Annex document(s) and are maintained within the Site Master File.

In situations where the assessment qualifies multiple agents' TMGs for a particular toxicity, the more conservative TMG should be applied.

On improvement of an AE for which Dato-DXd was temporarily delayed, Dato-DXd may be restarted at the same dose at the discretion of the investigator, unless specified otherwise in the TMGs. If a further episode of the same AE subsequently requires dose delay, or if a different AE subsequently requires dose delay, Dato-DXd may be restarted at a one dose level reduction on improvement of the AE or discontinued if the patient is receiving the lowest protocol-specified dose level.

Appropriate and optimal treatment of the toxicity should be attempted prior to considering dose modifications. If a patient experiences a clinically significant and/or unacceptable toxicity, dosing will be delayed or permanently discontinued in accordance with the TMGs, and supportive therapy administered as required.

8.3.15.3 Specific Toxicity Management and Dose Modification Information – Chemotherapy

Investigators should follow local standard clinical practice regarding dose modifications for chemotherapy. For specific information regarding the individual agent used in this study, please refer to the local prescribing information for the relevant agent. In the event of unfavourable tolerability, patients can switch from cisplatin to carboplatin therapy at any point during the study (assuming eligibility for the switched therapy is met). In patients with comorbidities or unable to tolerate cisplatin per Investigator's judgement, carboplatin AUC 5 can be administered from Cycle 1.

In the event that an AE can reasonably be attributed to chemotherapy, dose adjustment of chemotherapy should be attempted before modifying the administration of study interventions. Every effort should be made to ensure patients receive all scheduled cycles of chemotherapy across all treatment arms in the study, if conditions allow.

For management of toxicities due to the chemotherapy agents, refer to the locally approved package insert (label) or national guidance, or manage in accordance with documented institutional guidelines.

8.4 Overdose

Use of durvalumab, oleclumab, monalizumab, volrustomig, Dato-DXd, AZD0171, or rilvegostomig in doses exceeding that specified in the protocol is considered to be an overdose. There is currently no specific treatment in the event of overdose of the study interventions, and possible symptoms of overdose are not established.

- An overdose with associated AEs is recorded as the AE diagnosis/symptoms on the relevant AE modules in the eCRF and on the Overdose eCRF module.
- An overdose without associated symptoms is only reported on the Overdose eCRF module.

If an overdose on an AstraZeneca study intervention occurs in the course of the study, the Investigator or other site personnel inform appropriate AstraZeneca representatives immediately, but no later than 24 hours of when he or she becomes aware of it.

The designated AstraZeneca representative works with the Investigator to ensure that all relevant information is provided to the AstraZeneca Patient Safety data entry site within 1 or 5 calendar days for overdoses associated with an SAE (see Section 8.3.12) and within 30 days for all other overdoses.

For chemotherapy, refer to the local prescribing information for treatment of cases of overdose. If any overdose is associated with an AE or SAE, record the AE/SAE diagnosis or symptoms in the relevant AE modules only of the eCRF.

8.5 Human Biological Samples

Instructions for the collection, labelling, handling, storage and shipping of biological samples will be provided in the study-specific laboratory manual. Samples should be stored in a secure storage space with adequate measures to protect confidentiality.

For further details on Handling of Human Biological Samples, see Appendix C.

8.5.1 Pharmacokinetics

- Blood samples will be collected for measurement of serum or plasma concentrations of study interventions as specified in the SoA (refer to the specific study intervention SoAs in the relevant appendix).
- Non-compartment PK parameters that can be derived with sparse PK sampling, such as peak and trough concentrations, may be reported as data allow. Pharmacokinetic data may be combined with data from other studies for population PK modeling purposes. Exposure-response analysis may be performed to evaluate the relationship between PK and efficacy/safety endpoints. The population PK and exposure-response analysis, if

performed, will be based on a PK modeling plan and will be reported separately from the CSR.

8.5.1.1 Determination of Drug Concentration

Samples for determination of drug concentration in serum or plasma will be assayed by bioanalytical test sites operated by or on behalf of AstraZeneca, using an appropriately validated bioanalytical method. Full details of the analytical method used will be described in a separate Bioanalytical Report.

Incurred sample reproducibility analysis, if any, will be performed alongside the bioanalysis of the test samples. The results from the evaluation, if performed, will be reported in a separate Bioanalytical Report.

8.5.2 Immunogenicity Assessments

Blood samples for determination of ADA in serum or plasma will be taken according to SoA (refer to Table 1 for Screening; refer to the specific study intervention SoAs in the relevant appendix; and refer to Table 2 for patients who complete/discontinue treatment) and assayed by bioanalytical test sites operated by or on behalf of AstraZeneca, using an appropriately validated bioanalytical method. Full details of the methods used will be described in a separate report.

ADA samples may also be further tested for characterisation of the ADA response.

8.5.3 Storage and Destruction of Pharmacokinetic/ADA samples

Pharmacokinetic and ADA samples will be destroyed within 6 months of finalisation of the bioanalytical report and within 15 years of CSR finalisation, respectively.

Pharmacokinetic and ADA samples may be disposed of or destroyed and anonymised by pooling. Additional analyses may be conducted on the anonymised, pooled PK samples to further evaluate and validate the analytical method. Results from such analyses may be reported separately from the clinical study report.

Any residual back-up PK samples may be used for future exploratory biomarker research if future use consent has been provided (in this case, residual back-up PK samples will be shipped to AstraZeneca-assigned Biobank; see details in the Laboratory Manual).

8.5.4 Pharmacodynamics

Pharmacodynamic parameters are not evaluated in this study.

8.6 Human Biological Sample Biomarkers

Patient consent to the study includes participation in the mandatory biomarker assessment

components of the study.

Samples for biomarker assessment are required and will be collected from all patients in this study as specified in the SoA (refer to Table 1 for Screening; refer to the specific study intervention SoAs in the relevant appendix; and refer to Table 2 for patients who complete/discontinue treatment).

The results may be pooled with biomarker data from other studies to evaluate biological responses across indications and to compare results to those observed in other combination settings.

8.6.1 Baseline Tumour Samples for Biomarker Assessments

Tumour Sample Collection

Tissue samples will be obtained from all screened patients and will be used to

1 Evaluate for eligibility, and
2 Explore tumour biomarkers.

Tumour For Screening Eligibility

- In the absence of documented results for *PD-L1* and *EGFR/ALK* status at screening, a tumour sample, prior to randomisation, sufficient for the purpose of *PD-L1* expression analyses and for enabling *EGFR/ALK* testing where applicable, in addition to exploratory biomarker analysis will be collected. There are 2 options for the provision of the tumour tissue sample: 1) a newly acquired tumour biopsy during screening (preferred) or 2) an archival tumour sample taken ≤ 6 months prior to screening. Patients with unknown *PD-L1* status, and *EGFR/ALK* status where applicable (see below) will be considered screen failures and will not be eligible for randomisation in the study.
  - All patients will be tested for *PD-L1*
  - All patients will be tested for *EGFR*.
  - With the exception of patients with squamous cell carcinoma, all patients will be tested for *ALK*.

Provision of formalin-fixed paraffin-embedded tumour samples obtained <6 months prior to Screening is mandatory for entry to the study to enable exploratory analyses described below:

- It is recommended that samples should be collected by core needle biopsy, excisional or incisional biopsy. Cytology samples (slides or cell blocks), such as bronchial washings, bronchial lavage, and expectorated sputum, or specimens with limited tumour content are inadequate and will not be acceptable.

- The tumour specimen should be of sufficient quantity to allow for biomarker analyses (refer to Laboratory Manual). In the event there is insufficient quantity for exploratory biomarkers, a fresh sample must be provided, or the patient will not be eligible for randomisation.
- Details for collection, volumes, storage, and shipment of biologic samples are presented in a separate Pathology Manual and Laboratory Manual.

Please consult the Laboratory Manual for specific instructions and guidelines regarding sections.

Tumour lesions used for fresh biopsies should not be the same lesions used as RECIST 1.1 TLs (when possible), unless there are no other lesions suitable for biopsy, and in this instance only core needle (not excisional/incisional) biopsy is allowed. For patients with a single TL, if screening biopsy is collected prior to screening imaging for baseline tumour assessment, allow approximately 2 weeks before imaging scans are acquired.

EGFR/ALK Tumour Mutation Assessment (Eligibility criteria – Exclusionary criteria)

- *EGFR* and *ALK* status must be confirmed wild type prior to randomisation.
- Prior test results should be performed on well validated, local-regulatory-approved assay.
- Where the patient has not already been tested, local testing should be performed on well validated, local-regulatory-approved assay.
- *EGFR* and *ALK* may be retrospectively evaluated on all samples collected at screening and/or from surgical tissues where sufficient tissue is available to aid in further biomarker development activities

If patients have squamous cell carcinoma, then *ALK* status is not required at screening. For all other patients, *EGFR* and *ALK* status must be known to be negative prior to randomisation.

PD-L1 Expression Assessment (Randomisation/Stratification; Secondary endpoint; Eligibility Criteria – Exclusionary criteria [Arm 6 & 7 only])

- *PD-L1* status (< 1%, 1% to 49%, ≥ 50%) must be known prior to randomisation.
- Prior test results are accepted if performed on well validated, local-regulatory-approved assay.
- Where patient has not already been tested, PD-L1 testing with a local regulatory-approved analytically validated assay is required; SP263 antibody is preferred if available.
- *PD-L1* may additionally be retrospectively evaluated on all samples collected at screening and/or from surgical resection tissues where sufficient tissue is available to aid in further biomarker development activities.

Tumour Samples for Exploratory Biomarker Assessments (Exploratory endpoint)

Tumour and draining lymph node samples will be collected as indicated in SoAs (Table 1, Table 2 and the specific study intervention SoAs in the relevant appendix) to provide tissue for evaluation of DNA, RNA, and/or proteins. Analyses may include, but not limited to, profiling by immunohistochemistry PD-L1, CD73, LIF, TROP2, SLFN11, CD8, HLA-E, and NKG2A. Additionally analyses may include, but not be limited to, profiling DNA mutations/TMB, tumour microenvironment and immune cell infiltration, gene expression or gene signatures, T-cell or B cell responses by TCR/BCR sequencing, proteogenomic profiling, and/or development of personalised panel for MRD detection. These analyses may address baseline expression, changes in expression by comparison of baseline levels to on-treatment levels, comparison to blood-based biomarkers, correlations with clinical outcomes, understanding disease biology, and development of algorithms to predict pCR. In addition, the differences in normal adjacent lung tissue compared to tumour lung tissue after treatment may be assessed. Further details regarding tumour sample requirements are detailed in the Laboratory Manual.

8.6.2 Surgical Tumour Samples for Assessments

Pathological assessments: cPR and mPR (Primary and Secondary Endpoints)

Evaluation for pCR, which is primary endpoint, along with associated endpoint of mPR, a key secondary endpoint, will be determined by central blinded pathologist. To evaluate for these endpoints, all resected lung and draining lymph nodes needs to be processed per AJCC standard protocol and sent to central pathology. Detailed instructions are provided in the Pathology Manual for Lung Surgical Sample Handling.

Exploratory biomarkers (Exploratory endpoint)

Additional tumour tissue should be provided from surgical samples for exploratory biomarker research, which may include but not limited to changes in RNA, DNA, and/or proteins in surgical tumour tissue compared to baseline tissue, comparison to blood-based biomarkers, differences between tumour lung tissue and normal adjacent lung tissue, correlation with clinical outcomes, and development of specific algorithms to predict pCR. Similar analysis as described for the baseline samples may be conducted (See Section 8.6.1). Instructions for processing and shipment is available in the Laboratory Manual.

8.6.3 Blood Sample Collections for Biomarker Analysis

Blood samples for biomarker analyses will be obtained according to the schedules presented in the SoAs: refer to Table 1 for Screening; refer to the specific study intervention SoAs in the relevant appendix; and refer to Table 2 for patients who complete/discontinue treatment. Details for collection, volumes, storage, and shipment of biologic samples are presented in a separate Laboratory Manual.

Baseline, on-treatment, and post-surgery measures will be correlated with outcomes.

Comparisons will be made to determine if biomarkers (or combination of markers) are prognostic or predictive of outcomes associated with study interventions across treatment arms. Analyses may also address comparison of baseline levels and on-treatment measures, comparison to tumour tissue or normal adjacent tissue measures, development of personalised panel for MRD detection, associations with adverse reactions to study interventions, understanding disease biology, and/or development of approaches to predict clinical outcomes.

Additional blood sample collections and analyses may be completed at select study sites by site-specific amendment. All samples collected for such exploratory analyses will be stored at site, a reference laboratory, or at AstraZeneca's facilities and may be used for subsequent research relevant to evaluating response to immunotherapy.

The exploratory biomarker plan is described by sample type below.

Whole Blood for [redacted] Expression

Whole blood samples will be collected from all patients according to the collection schedule in the Screening SoA (Table 1) and the specific study intervention SoAs in the relevant appendix. Analyses may include, but not limited to, changes in expression of [redacted] or [redacted] signatures, between baseline and on-treatment measures, and correlation of [redacted] expression or [redacted] expression changes to clinical outcome.

Serum Samples for Circulating Soluble Factors and Plasma Samples for Circulating Soluble Factors

A peripheral blood sample will be collected at the time points indicated in SoA (refer to Table 1 for Screening; refer to the specific study intervention SoAs in the relevant appendix) to provide serum and plasma prior to administration of any study intervention as well as on treatment during the study. These samples may be used for analysis of, but not limited to, baseline and on-treatment changes in circulating levels of target proteins (eg, LIF), soluble factors, and/or immune mediators of antitumour immune response to explore their association with treatment and clinical outcome.

Plasma Samples for Circulating Tumour DNA Analyses

Plasma for ctDNA analysis will be isolated from blood samples collected at the time points indicated in SoA: refer to Table 1 for Screening; refer to the specific study intervention SoAs in the relevant appendix; and refer to Table 2 for patients who complete/discontinue treatment. Analyses may include, but will not be limited to, evaluating baseline mutations prior to the start of treatment, changes in ctDNA levels on-treatment and/or post-surgery, and correlations with clinical outcomes. Plasma may also be used to analyse relevant cytokines, chemokines, and other immune-related markers.

The buffy coat layers obtained during the plasma isolation of the baseline sample may be retained and analysed for germline mutations as per local regulations. See Section 8.7.1.

Whole blood for DNA Analyses

Whole blood sample will be collected from all patients according to collection schedule in the relevant SoA (refer to Table 1 for Screening; refer to the specific study intervention SoAs in the relevant appendix). Sequencing of isolated DNA may include, but not be limited to, targeted sequencing of the HLA loci to assess the impact of HLA diversity on treatment outcome. Sequencing data may also involve whole-exome sequencing or whole-genome sequencing, which will be conducted in order to provide a control germline sequence (as per local regulations) for comparison to sequence from tumour DNA or ctDNA. Such comparisons are critical for certain analyses, such as for highly sensitive detection of ctDNA as a means of assessing minimal residual disease. See Section 8.7.1.

Whole Blood for Peripheral Blood Mononuclear Cells (PBMCs)

Peripheral blood mononuclear cells from blood will be collected: refer to Table 1 for Screening; refer to the specific study intervention SoAs in the relevant appendix. Analyses may include, but not limited to, measuring immune cell populations, phenotypes, and T and/or B cell receptor repertoire changes with treatment and associations with clinical outcomes as well as emergence of T-cell responses to neoantigen epitopes.

Whole Blood for Flow Cytometry

Whole blood will be collected from patients treated with volrustomig or rilvegostomig according to collection schedule in SoA (refer to the specific study intervention SoAs in the relevant appendix). This sample may be analysed for changes in peripheral cell populations, immunophenotyping, and/or receptor occupancy compared to baseline, comparison to tumour biomarkers, and/or in association with clinical outcomes. If a patient requires an on-study MUGA scan, in order to ensure that radioactivity levels from the radiotracer have decayed to exempt levels before receipt of samples at the central laboratory, blood samples for flow cytometry should be collected either before the MUGA scan or at least 2 days after the MUGA scan has been performed.

8.6.4 Collection of Optional On-Treatment Tumour Tissue Samples or Biopsy at Progression

Tumour samples that become available secondary to a surgical procedure or a biopsy during the study or at time of progression should be collected. Analyses performed on tumour tissue collected at progression may include, but are not limited to, acquired genomic alterations associated with resistance to study intervention, up- or downregulation of proteins or gene expression, characterization of immune cell infiltration and tumour microenvironment, and changes in tumour histology. Similar analyses as described for the baseline samples may also

be conducted (See Section 8.6.1). Instructions for processing and shipment is available in the Laboratory Manual.

8.6.5 Management of Biomarker Data

The biomarker data will have unknown clinical significance. AstraZeneca will not provide biomarker research results to patients, their family members, any insurance company, an employer, clinical study Investigator, general physician, or any other third party, unless required to do so by law. The patient's samples will not be used for any purpose other than those described in the CSP. Individual patients will not be identified in any report or publication resulting from this work.

The data and results of this research may be reviewed with collaborators and published, but neither the patient's name nor any other personal identifiers will appear in any publication or report.

8.6.6 Storage, Re-use, and Destruction of Biomarker Samples

Samples will be stored for a maximum of 15 years from the CSR, after which they will be destroyed. Summaries and analyses for exploratory biomarkers will be documented in a separate analysis plan and will be reported outside the CSR in a separate report.

The results of this biomarker research may be pooled with biomarker data from other studies to generate hypotheses to be tested in future research, and/or to evaluate biological responses across indications and to compare results in monotherapy versus combination settings.

8.7 Genetics

8.7.1 Blood samples for [redacted] Analyses

A whole blood sample for [redacted] analyses (see section 8.6.3) will be obtained according to the schedule presented in SoA (refer to Table 1 for Screening; refer to the specific study intervention SoAs in the relevant appendix; and refer to Table 2 for patients who complete/discontinue treatment) and may be retained and analysed for germline mutations as per local regulations. Buffy coat layers obtained during plasma isolation from the ctDNA sample may also be retained and analysed for germline mutations as per local regulations.

8.7.2 Optional [redacted] Initiative

Collection of optional samples for [redacted] initiative research is also part of this study as specified in the SoA and is subject to agreement in the ICF addendum.

If the patient agrees to participate in the optional [redacted] research study, a blood sample will be collected. Participation is optional. Patients who do not wish to participate in the [redacted] research may still participate in the study.

In the event of [redacted] extraction failure, a replacement [redacted] blood sample may be requested from the patient. Signed informed consent will be required to obtain a replacement sample unless it was included in the original consent.

See Appendix D for information regarding the storage and destruction of [redacted] Initiative [redacted] sample. Details on processes for collection and shipment and destruction of these samples can be found either in the appendices or in the Laboratory Manual.

8.8 Health Economics OR Medical Resource Utilisation and Health Economics

Health Economics/Medical Resource Utilisation and Health Economics parameters are not evaluated in this study.

9 STATISTICAL CONSIDERATIONS

Statistical analyses will be performed by AstraZeneca or its representatives, including CROs.

9.1 Statistical Hypotheses

No formal statistical comparisons among treatment arms will be performed.

9.2 Sample Size Determination

For Arms 1, 2, 3A, 4, 5, 6, and 7, the sample size of up to 70 patients per arm is to:

- Ensure the accuracy of making Go or No-Go decision at the interim analysis and final analysis.
- Obtain a preliminary assessment of antitumour activity with a certain degree of precision.

The sample size is not based on type I error and power considerations. [redacted]

[redacted] [redacted]

[redacted]	[redacted]	[redacted]	[redacted]	
			[redacted]	[redacted]
Interim Analysis Stage				
[redacted]				
[redacted]				

[redacted]	[redacted]	[redacted]	[redacted]	
			[redacted]	[redacted]

[redacted]

Arms 3B and 3C plan to enrol [redacted] patients per arm to evaluate tolerability of alternate dose regimens, with a potential to increase to [redacted] patients per arm to further understand safety and efficacy.

9.3 Populations for Analysis

The following populations are defined:

Table 13 Populations for Analysis

Population/Analysis Set	Description
Enrolled	All patients who sign the ICF
ITT	All randomised patients in the study.
Response evaluable	All dosed patients who had measurable disease at baseline
Safety	All patients who receive at least 1 dose of any study interventions
PK	All patients who receive at least 1 dose of any study interventions with at least 1 reportable PK concentration
Immunogenicity	All patients who receive at least 1 dose of any study interventions with at least 1 reportable immunogenicity concentration

Other analysis populations may be defined in the SAP, if appropriate.
Abbreviations: ICF: Informed consent form; ITT: Intent-to treat; PK: Pharmacokinetics.

9.4 Statistical Analyses

This section is a summary of the planned statistical analyses of the most important endpoints including primary and key secondary endpoints. The SAP includes a more technical and detailed description of the statistical analyses described in this section.

9.4.1 General Considerations

Statistical analyses will be performed by AstraZeneca or its representatives, including CROs.

Continuous data will be summarised by the number of observations, mean standard deviation, median, minimum, and maximum. Geometric mean and coefficient of variation may be presented as applicable. Categorical variables will be summarised by frequency counts and percentages for each category. Unless otherwise stated, percentages will be calculated from the population total. Time-to-event variables will be presented using the Kaplan-Meier methodology, including median time and rates at landmark timepoints estimated from the

Kaplan-Meier curves, where feasible.

In general, the last observed measurement prior to first dose of study interventions will be considered the baseline measurement. For assessment on the day of first day where time is not captured, a nominal pre-dose indicator, if available, will serve as sufficient evidence that the assessment occurred prior to first dose. Assessments on the day of first dose, when neither time nor nominal pre-dose indicator are captured, will be considered prior to first dose if such procedures are required by the protocol to be conducted before the first dose.

Depending on the extent of any impact, summaries of data relating to patients diagnosed with COVID-19, and impact of COVID-19 on study impact (in particular missed visits, delayed or discontinued study treatment, and other protocol deviations) may be generated. More details will be provided in the SAP.

All efficacy analyses will be performed by randomised treatment arm on the ITT population unless otherwise stated. Additional analyses may be performed using different PD-L1 cut-offs in addition to 1% and 50%, and will be further described in the SAP. All safety analyses will be performed on the safety population.

9.4.2 Efficacy

9.4.2.1 Primary Endpoint

Pathological complete response is defined as the proportion of patients who have 0% residual viable tumour cell within all resected tissue (including primary lung lesion and lymph nodes) following neoadjuvant treatment as assessed by central BIPR laboratory. Patients who are not evaluable per central pathology assessment (this includes patients with R2 margins) or who do not have a surgical specimen will be considered as non-pCR (eg, pathology assessments captured as “non-evaluable” or “missing”, as appropriate). Central pathology assessment of pCR will be performed according to the recommended methods and definitions described by IASLC 2020 (Travis et al, 2020).

Pathological complete response will be presented by the number and percentage of patients with pCR including [redacted] and [redacted] Clopper-Pearson (exact) CIs for each arm.

[redacted]

9.4.2.2 Secondary Endpoints

Event-free survival

Event-free survival is defined as the time from randomisation to the first of the following: a) documented local or distant recurrence as determined by Investigator using RECIST 1.1 assessments; b) death due to any cause (event date is the date of death); c) PD that precludes surgery (event date is date of this determination) or PD discovered and reported by the Investigator upon attempting surgery that prevents completion of surgery (event date is the date of the first attempt at surgery). A new primary malignancy, confirmed by pathology, is not considered an EFS event.

Kaplan-Meier plots of EFS will be presented by treatment arm. Summaries of number and percentage of patients experiencing an EFS event, and the type of event will be provided along with the median EFS for each treatment. Landmark analyses will be presented for 12 and 24 months.

Subgroup analysis may be performed and will further be described in the SAP.

Disease-free survival

Disease-free survival will only be evaluated for patients who had surgical resection following the neoadjuvant period, who have R0/R1 margins, and whose first post-surgical RECIST 1.1 scan shows no evaluable disease (defined as no post-surgery R2 margins and no RECIST evidence of disease). Disease-free survival is defined as the time from the date of surgery until the first date of disease recurrence as determined by Investigator using RECIST 1.1 assessments (local or distant), or date of death due to any cause, whichever occurs first. Pathological confirmation from biopsied lesions, will also be taken into consideration (as applicable). A new primary malignancy, confirmed by pathology, is not considered a DFS event.

Kaplan-Meier plots of DFS will be presented by treatment arm. Summaries of number and percentage of patients experiencing a DFS event, and the type of event will be provided along with the median DFS for each treatment. Landmark analyses will be presented at 12 and 24 months.

Feasibility to surgery

Feasibility to surgery is defined as having the planned surgical resection within 40 days from the last dose of neoadjuvant study interventions administration.

Summaries of number and percentage of patients having surgical resection within 40 days from the end of last neoadjuvant study interventions administered will be presented for each treatment arm. The rate and outcomes of surgical complications will also be summarized.

Major pathological response

Major pathological response rate is defined as the proportion of patients with 10% or less residual viable tumour tissue in lung primary tumour after neoadjuvant treatment at the time of resection as assessed per central BIPR laboratory. Patients who are not evaluable per central pathology assessment (including patients with R2 margins) or who do not have a surgical specimen will be considered as having non-mPR (eg, response captured as "non-evaluable" or "missing" as appropriate).

Major pathological response will be presented by the number and percentage of patients with mPR, including 80% and 95% Clopper-Pearson CIs for each arm.

Objective response rate

Objective response rate is defined as the proportion of patients achieving either CR or PR, prior to surgery, as assessed by Investigator.

Data obtained up until surgery, or last evaluable assessment in the absence of surgery will be included in the assessment of ORR, regardless of whether the patient withdraws from therapy. Patients who discontinue treatment without a response or surgery, receive subsequent anti-cancer therapy prior to surgery and then respond will not be included as responders in the ORR.

Objective response rate will include data from all scans, regardless of whether it was scheduled or not.

Objective response rate will be presented by the number and percentage of patients with a response including 95% Clopper-Pearson CIs for each arm.

Overall survival

The OS is defined as the time from randomisation until death due to any cause regardless of whether patient withdraws from treatment or receives another anti-cancer therapy. Any patient not known to have died at the time of analysis will be censored at the last recorded date on which the patient was known to be alive.

Kaplan-Meier plots of OS will be presented by treatment arm. Summaries on the number and percentage of patients who have died, those still in survival follow-up and those lost to follow-up and those who have withdrawn consent will be provided along with the median OS for each treatment arm if feasible.

Additional landmark analyses will be performed for OS at 12 months, 24 months and at other clinically relevant timepoints if feasible. If reached by the end of the study, the median OS will also be of interest.

Other secondary endpoints are described in Section 9.4.4.

9.4.3 Safety

Safety analyses will be performed using the safety analysis set. Safety data will be presented descriptively unless otherwise specified.

Adverse Events

Adverse events will be coded using the most recent version of the MedDRA that will have been released for execution at AZ/designee.

Adverse events will be presented for each treatment arm by chemotherapy agents and/or PT covering number and percentage of patients reporting at least one event and number of events where appropriate.

Adverse events occurring prior to the start of study interventions, treatment-emergent AEs and post-treatment AEs will be presented separately.

An overview of AEs will be presented for each treatment arm with the number and percentage of patients with any AE, AEs with outcome of death, serious AEs, AEs leading to discontinuation of investigational product, as well as AEs leading to study interventions dose interruptions, AEs leading to dose reduction and AEs leading to treatment or study withdrawal as well as the number of individual occurrences in these categories.

Separate AE tables will be provided taken into consideration relationship as assessed by the Investigator, maximum CTC grading, seriousness, death, and events leading to discontinuation of study interventions as well as other action taken related to study interventions, events of special interest, other significant adverse events and timing of events.

An additional table will present the number and percentage of patients with most common AEs. Most common will be defined in the SAP.

Key patient information will be presented for patients with AEs with outcome of death, serious AEs, and AEs leading to discontinuation of study interventions.

An AE listing for the safety analysis set will cover details for each individual AE.

Full details of AE analyses will be provided in the SAP.

Treatment emergent

The following events are considered treatment emergent:

- Adverse events with an onset date on or after first dose of study interventions and within 90 days after last dose or last study intervention (including surgery), or up to

the day prior to start of subsequent anti-cancer therapy, whichever comes first.

- Worsening of pre-existing events on or after first dose of study interventions and within 90 days after last dose or last study intervention (including surgery), or up to the day prior to start of subsequent anti-cancer therapy, whichever comes first.

Vital signs

For each scheduled post-baseline visit, descriptive statistics for all vital signs parameters will be presented for observed values and changes from baseline.

Details of vital signs analyses, including definition of abnormality criteria (eg definition of low, normal, high) and project specific criteria for treatment-emergent changes in vital signs parameters will be provided in the SAP.

Laboratory parameters

For each scheduled post-baseline visit, descriptive statistics for all clinical chemistry, haematology, and TSH parameters will be presented for observed values and change from baseline.

Details of analyses of laboratory safety variables will be provided in the SAP.

Electrocardiogram

For each scheduled post-baseline assessment, descriptive statistics for all ECG parameters will be presented for observed values and change from baseline.

Details of ECG analyses, including definition of reference values and abnormalities will be provided in the SAP.

9.4.4 Other Analyses

9.4.4.1 Pharmacokinetics

Pharmacokinetic concentrations will be listed and summarised using descriptive statistics by analyte, treatment arm, visit and scheduled timepoint. Non-compartmental PK parameters that can be derived with sparse PK sampling will be reported as data allow. Details of PK analyses will be described in the SAP.

9.4.4.2 Biomarkers

The relationship of baseline PD-L1 expression and ctDNA clearance to clinical outcomes (including but not restricted to) mPR and pCR may be presented.

Baseline PD-L1 and ctDNA clearance on-treatment prior to surgery are secondary endpoints for this study. Baseline ctDNA will be categorised as detected or not detected. For patients

with detected baseline ctDNA on treatment assessments prior to surgery will be compared to baseline and classified as complete clearance or not complete clearance. Further details will be provided in the SAP.

Biomarker status will be assessed for patients in each arm according to pre-specified criteria that may be detailed in the SAP. The relationship of biomarkers expression and, if applicable, of exploratory biomarkers to clinical outcomes (including but not restricted to) of pCR and mPR may be presented. Biomarker exploratory analyses may be described in a separate analysis plan and may be reported outside the CSR in a separate report. The results of this biomarker assessment may be reported either in the CSR itself or as an addendum, or separately in a scientific report or publication. The results of this biomarker assessment may be pooled with biomarker data from other studies with the study intervention to generate hypotheses to be tested in future research.

9.4.4.3 [redacted]

Data will be reported outside the CSR (please see Appendix D).

9.4.4.4 Immunogenicity Data

Immunogenicity results will be listed by patient, and a summary will be provided by the number and percentage of patients who develop detectable anti-study drug antibodies. The immunogenicity titre and neutralising ADA data (if available) will be listed for samples confirmed positive for the presence of anti-study drug antibodies.

9.5 Interim Analyses

A pCR interim analysis will be performed once 35 patients have received neoadjuvant treatment and completed surgery. Analyses will be based on several interim analysis sets, which are defined in the SAP. The interim analysis will be assessed against a decision framework (Freeman et al 2000

Freeman GJ, Long AJ, Iwai Y, Bourque K, Chernova T, Nishimura H, et al. Engagement of the PD-1 immunoinhibitory receptor by a novel B7 family member leads to negative regulation of lymphocyte activation. J Exp Med. 2000;192(7):1027-34.

Frewer et al, 2016) for pCR. For Arms 1, 2, 3A, 4, 5, 6, and 7, with 35 patients, futility is met if 6 or fewer pCRs are observed. The decision on futility will also be made based upon the totality of the data. Recruitment will not be paused while the interim analysis is evaluated. The SAP will describe the planned interim analysis in greater detail.

9.6 Data Monitoring Committee

The study will have no data monitoring committee. The study will have a SRC to conduct safety reviews. The membership, roles and responsibilities are defined in a charter (separate from the CSP).

For a brief description on SRC activities refer to Section 4.1 and for details on SRC structure refer to Appendix A 5.

9.7 ILD Adjudication Committee

An independent ILD Adjudication Committee is responsible for reviewing all cases of potential ILD/pneumonitis on Arms 4 and 7 as part of the Dato-DXd programme requirements. To ensure adequate and relevant independent evaluation, systematic additional data collection will be conducted for all cases that will be brought for adjudication. This additional data collection will cover a more in-depth relevant medical history (eg, smoking, radiation, COPD, and other chronic lung conditions); diagnostic evaluation, treatment, and outcome of the event. This data collection will be triggered based on a pre-defined list of PTs eligible for adjudication as described in the Event Adjudication Site Manual.

10 SUPPORTING DOCUMENTATION AND OPERATIONAL CONSIDERATIONS

Appendix A Regulatory, Ethical, and Study Oversight Considerations

A 1 Regulatory and Ethical Considerations

- This study will be conducted in accordance with the protocol and with the following:
  - Consensus ethical principles derived from international guidelines including the Declaration of Helsinki and as amended at 64th Word Medical Association (WMA) General Assembly, Fortaleza, Brazil, October 2013 and Council for International Organisations of Medical Sciences (CIOMS) International Ethical Guidelines.
  - Applicable ICH GCP Guidelines.
  - Applicable laws and regulations.
  - Specifically, for the EU, the Sponsor will be responsible to ensure that the study will be conducted in compliance with the protocol, in adherence to the requirements of the European Regulation 536/2014.
- The protocol, revised protocol, ICF, IB, and other relevant documents (eg, advertisements) must be submitted to an IRB/IEC by the investigator and reviewed and approved by the IRB/IEC before the study is initiated.
- Any revised protocol will require IRB/IEC and applicable Regulatory Authority approval before implementation of changes made to the study design, except for changes necessary to eliminate an immediate hazard to study patients.
- AstraZeneca will be responsible for obtaining the required authorisations to conduct the study from the concerned Regulatory Authority. This responsibility may be delegated to a CRO but the accountability remains with AstraZeneca.
- The investigator will be responsible for providing oversight of the conduct of the study at the site and adherence to requirements of 21 CFR 312.120, ICH guidelines, the IRB/IEC, European Regulation 536/2014 for clinical studies (if applicable), European Medical Device Regulation 2017/745 for clinical device research (if applicable), and all other applicable local regulations.

Regulatory Reporting Requirements for SAEs

- Prompt notification by the Investigator to AstraZeneca of an SAE is essential so that legal obligations and ethical responsibilities towards the safety of patients and the safety of a study intervention under clinical investigation are met.
- AstraZeneca has a legal responsibility to notify both the local regulatory authority and other regulatory agencies about the safety of a study intervention under clinical investigation. AstraZeneca will comply with country-specific regulatory requirements relating to safety reporting to the regulatory authority, IRB/IEC, and Investigators.

- In the EU, the Sponsor will comply with safety reporting requirements and procedures as described in the European Clinical Trials Regulation (EU) No 536/2014. All SUSARs to IMP will be reported to the EudraVigilance database within the required regulatory timelines.
- For all studies except those utilising medical devices, Investigator safety reports must be prepared for SUSARs according to local regulatory requirements and sponsor policy and forwarded to Investigators as necessary.
- Adherence to European Medical Device Regulation 2017/745 for clinical device research (if applicable), and all other applicable local regulations.
- An Investigator who receives an investigator safety report describing a SAE or other specific safety information (eg, summary or listing of SAEs) from AstraZeneca will review and then file it along with the [IB or state other documents] and will notify the IRB/IEC, if appropriate according to local requirements.

Regulatory Reporting Requirements for Serious Breaches

- Prompt notification by the investigator to AstraZeneca of any (potential) serious breach of the protocol or regulations is essential so that legal and ethical obligations are met.
  - A 'serious breach' means a breach likely to affect to a significant degree the safety and rights of a patient or the reliability and robustness of the data generated in the clinical study.
- If any (potential) serious breach occurs in the course of the study, investigators or other site personnel will inform the appropriate AstraZeneca representatives immediately after they become aware of it.
- In certain regions/countries, AstraZeneca has a legal responsibility to notify both the local regulatory authority and other regulatory agencies about such breaches.
  - AstraZeneca will comply with country-specific regulatory requirements relating to serious breach reporting to the regulatory authority, IRB/IEC, and investigators. If EU Clinical Trials Regulation 536/2014 applies, AstraZeneca is required to enter details of serious breaches into the European Medicines Agency (EMA) Clinical Trial Information System (CTIS). It is important to note that redacted versions of serious breach reports will be available to the public via CTIS.
- The investigator should have a process in place to ensure that:
  - The site staff or service providers delegated by the investigator/institution are able to identify the occurrence of a (potential) serious breach.
  - A (potential) serious breach is promptly reported to AstraZeneca or delegated party, through the contacts (email address or telephone number) provided by AstraZeneca.

A 2 Financial Disclosure

Investigators and sub-investigators will provide the Sponsor with sufficient, accurate financial information as requested to allow the Sponsor to submit complete and accurate financial certification or disclosure statements to the appropriate regulatory authorities. Investigators are responsible for providing information on financial interests during the course of the study and for 1 year after completion of the study.

A 3 Informed Consent Process

- The Investigator or his/her representative will explain the nature of the study to the patient or his/her legally authorised representative and answer all questions regarding the study.
- Patients must be informed that their participation is voluntary and they are free to refuse to participate and may withdraw their consent at any time and for any reason during the study. Patients or their legally authorised representative will be required to sign a statement of informed consent that meets the requirements of 21 CFR 50, local regulations, ICH guidelines, Health Insurance Portability and Accountability Act requirements, where applicable, and the IRB/IEC or study centre.
- The medical record must include a statement that written informed consent was obtained before the patient was enrolled in the study and the date the written consent was obtained. The authorised person obtaining the informed consent must also sign the ICF.
- Patients must be re-consented to the most current version of the ICF(s) during their participation in the study.
- A copy of the ICF(s) must be provided to the patient or the patient's legally authorised representative.

If a patient declines to participate in any voluntary exploratory genetic research component of the study, there will be no penalty or loss of benefit to the patient and he/she will not be excluded from other aspects of the study.

If a patient's partner becomes pregnant during or within the following time periods after last dose of study interventions the partner is asked to sign the "Adult Study Informed Consent Form for Pregnant Partners of Study Patients" and provide information about the pregnancy accordingly:

- Durvalumab or volrustomig: 90 days
- Oleclumab, monalizumab, or AZD0171: 180 days
- Dato-DXd: 120 days
- Rilvegostomig: 60 days

For the chemotherapy agents follow the local prescribing information relating to contraception, the time limits for such precautions, and any additional restrictions for the agents administered.

For patients receiving more than one study intervention, the longest period will apply.

The ICF will contain a separate section that addresses and documents the collection and use of any mandatory samples for optional exploratory research and/or optional human biological samples. The Investigator or authorised designee will explain to each patient the objectives of the analysis to be done on the samples and any potential future use. Patients will be told that they are free to refuse to participate in any optional samples or the future use and may withdraw their consent at any time and for any reason during the retention period. The patient will give a separate agreement to allow any remaining specimens to be used for exploratory research. Patients who decline to participate in this optional research will indicate this in the ICF. If a patient withdraws consent to the use of donated biological samples, the samples will be disposed of/destroyed, and the action documented. If samples already have been analysed at the time of the request, AstraZeneca will not be obliged to destroy the results of this research.

A 4 Data Protection

The ICF will incorporate wording that complies with relevant data protection and privacy legislation. In some cases, such wording will be in a separate accompanying document. AstraZeneca will not provide individual genotype results to patients, their family members, their general physician, any insurance company, any employer, or any other third party, unless required to do so by law.

Precautions are taken to preserve confidentiality and prevent genetic data from being linked to the identity of the patient. In exceptional circumstances, however, certain individuals might see both the genetic data and the personal identifiers of a patient. For example, in the case of a medical emergency, an AstraZeneca Physician or an Investigator might know a patient's identity and might also have access to his or her genetic data. Also, regulatory authorities may require access to the relevant files. Even so, the patient's medical information and the genetic files would remain physically separate.

- Patients will be assigned a unique identifier by the Sponsor. Any patient records or datasets that are transferred to the Sponsor will contain the identifier only; patient names or any information which would make the patient identifiable will not be transferred.
- The patient must be informed that his/her personal study-related data will be used by the Sponsor in accordance with local data protection law. The level of disclosure and use of their data must also be explained to the patient in the informed consent

- The patient must be informed that his/her medical records may be examined by Clinical Quality Assurance auditors or other authorised personnel appointed by the Sponsor, by appropriate IRB/IEC members, and by inspectors from regulatory authorities.

Unless previously specified, the biomarker data will have unknown clinical significance and AstraZeneca will not provide biomarker assessment results to patients, their family members, any insurance company, any employer, a clinical study Investigator, a general physician, or any other third party, unless required to do so by law.

The patient's samples will not be used for any purpose other than those described in the study protocol.

A 5 Committees Structure

The safety of all AstraZeneca clinical studies is closely monitored on an on-going basis by AstraZeneca representatives in consultation with Patient Safety. Issues identified will be addressed; for instance, this could involve amendments to the CSP and letters to Investigators.

A 5.1 Safety Review Committee

An SRC will conduct safety reviews of all enrolled patients throughout the study (See Section 4.1).

The SRC may make recommendations regarding continuation, modification, or termination of any treatment arm for safety concerns. Details on the process flow/communication plan are provided in the SRC Charter.

A 6 Dissemination of Clinical Study Data

Any results both technical and lay summaries for this study, will be submitted to EU CTIS within a year from global End of Trial Date in all participating countries, due to scientific reasons, as otherwise statistical analysis is not relevant.

A description of this clinical study will be available on www.astrazenecaclinicaltrials.com [http://www.clinicaltrials.gov and https://euclinicaltrials.eu] as will the summary of the main study results when they are available. The clinical study and/or summary of main study results may also be available on other websites according to the regulations of the countries in which the main study is conducted.

A 7 Data Quality Assurance

- All patient data relating to the study will be recorded on the eCRF unless transmitted to the Sponsor or designee electronically (eg, laboratory data). The Investigator is

responsible for verifying that data entries are accurate and correct by physically or electronically signing the eCRF.

- The Investigator must maintain accurate documentation (source data) that supports the information entered in the eCRF.
- The Investigator must permit study-related monitoring, audits, IRB/IEC review, and regulatory authority inspections and provide direct access to source data documents.
- Monitoring details describing strategy, including definition of study-critical data items and processes (eg, risk-based initiatives in operations and quality such as Risk Management and Mitigation Strategies and Analytical Risk-Based Monitoring), methods, responsibilities and requirements, including handling of noncompliance issues and monitoring techniques (central, remote, or on-site monitoring) are provided in the Monitoring Plan.
- The Sponsor or designee is responsible for medical oversight throughout the conduct of the study which includes clinical reviews of study data in accordance with the currently approved protocol. Monitoring details describing clinical reviews of study data from a medical perspective are included in more detail in the Medical Monitoring Plan.
- The Sponsor or designee is responsible for the data management of this study including quality checking of the data.
- The Sponsor assumes accountability for actions delegated to other individuals (eg, CROs).

Study monitors will perform ongoing source data verification as per the Monitoring Plan to confirm that data entered into the eCRF by authorised site personnel are accurate, complete, and verifiable from source documents; that the safety and rights of patients are being protected; and that the study is being conducted in accordance with the currently approved protocol and any other study agreements, ICH GCP, and all applicable regulatory requirements.

- Records and documents, including signed ICFs, pertaining to the conduct of this study must be retained by the investigator for 15 years from the end of the study unless local regulations or institutional policies require a longer retention period. No records may be destroyed during the retention period without the written approval of the Sponsor. No records may be transferred to another location or party without written notification to the Sponsor.

A 8 Source Documents

- Source documents provide evidence for the existence of the patient and substantiate the integrity of the data collected. Source documents are filed at the Investigator's site.

- Data reported on the eCRF or entered in the eCRF that are transcribed from source documents must be consistent with the source documents or the discrepancies must be explained. The investigator may need to request previous medical records or transfer records, depending on the study. Also, current medical records must be available.
- All information in original records and certified copies of original records of clinical findings, observations, or other activities in a clinical study necessary for the reconstruction and evaluation of the study are defined as source documents. Source data are contained in source documents (original records or certified copies).

A 9 Study and Site Start and Closure

The study start date is the date on which the clinical study will be open for recruitment of patients.

The first act of recruitment is the first site open and will be the study start date.

The Sponsor designee reserves the right to close the study site or terminate the study at any time for any reason at the sole discretion of the Sponsor. Study sites will be closed upon study completion. A study site is considered closed when all required documents and study supplies have been collected and a study-site closure visit has been performed.

The Investigator may initiate study-site closure at any time, provided there is reasonable cause and sufficient notice is given in advance of the intended termination.

Reasons for the early closure of a study site by the Sponsor or Investigator may include but are not limited to:

- Failure of the Investigator to comply with the protocol, the requirements of the IRB/IEC or local health authorities, the Sponsor's procedures, or GCP guidelines.
- Inadequate recruitment of patients by the Investigator.
- Discontinuation of further study intervention development.

If the study is prematurely terminated or suspended, the Sponsor shall promptly inform the Investigators, the IECs/IRBs, the regulatory authorities, and any CROs used in the study of the reason for termination or suspension, as specified by the applicable regulatory requirements. The Investigator shall promptly inform the patient and should assure appropriate patient therapy and/or follow-up.

Patients from terminated sites will have the opportunity to be transferred to another site to continue the study.

A 10 Publication Policy

- The results of this study may be published or presented at scientific meetings. If this is foreseen, the Investigator agrees to submit all manuscripts or abstracts to the Sponsor before submission. This allows the Sponsor to protect proprietary information and to provide comments.
- The Sponsor will comply with the requirements for publication of study results. In accordance with standard editorial and ethical practice, the Sponsor will generally support publication of multicentre studies only in their entirety and not as individual site data. In this case, a co-ordinating Investigator will be designated by mutual agreement.
- Authorship will be determined by mutual agreement and in line with International Committee of Medical Journal Editors authorship requirements.

Appendix B Adverse Events: Definitions and Procedures for Recording, Evaluating, Follow-up, and Reporting

B 1 Definition of Adverse Events

An AE is the development of any untoward medical occurrence (other than progression of the malignancy under evaluation) in a patient or clinical study patient administered a study intervention and which does not necessarily have a causal relationship with this treatment. An AE can therefore be any unfavourable and unintended sign (eg, an abnormal laboratory finding), symptom (for example nausea, chest pain), or disease temporally associated with the use of a medicinal product, whether or not related to the study intervention.

The term AE is used to include both serious and non-serious AEs and can include a deterioration of a pre-existing medical occurrence. An AE may occur at any time, including run-in or washout periods, even if no study intervention has been administered.

B 2 Definition of Serious Adverse Event

An SAE is an AE occurring during any study phase (ie, run-in, treatment, washout, follow-up), that fulfils one or more of the following criteria:

- Results in death.
- Is immediately life-threatening.
- Requires in-patient hospitalisation or prolongation of existing hospitalisation.
- Results in persistent or significant disability or incapacity.
- Is a congenital abnormality or birth defect.
- Is an important medical event that may jeopardise the patient or may require medical treatment to prevent one of the outcomes listed above.

Adverse events for **malignant tumours** reported during a study should generally be assessed as **SAEs**. If no other seriousness criteria apply, the "Important Medical Event" criterion should be used. In certain situations, however, medical judgement on an individual event basis should be applied to clarify that the malignant tumour event should be assessed and reported as a **Non-Serious** AE. For example, if the tumour is included as medical history and progression occurs during the study, but the progression does not change treatment and/or prognosis of the malignant tumour, the AE may not fulfil the attributes for being assessed as serious, although reporting of the progression of the malignant tumour as an AE is valid and should occur. Also, some types of malignant tumours, which do not spread remotely after a routine treatment that does not require hospitalisation, may be assessed as Non-Serious; examples in adults include Stage 1 basal cell carcinoma and Stage 1A1 cervical cancer removed via cone biopsy.

The above instruction applies only when the malignant tumour event in question is a new

malignant tumour (ie, it is *not* the tumour for which entry into the study is a criterion and that is being treated by the study intervention under study and is not the development of new or progression of existing metastasis to the tumour under study). Malignant tumours that – as part of normal, if rare, progression – undergo transformation (eg, Richter's transformation of B cell chronic lymphocytic leukaemia into diffuse large B cell lymphoma) should not be considered a new malignant tumour.

Life threatening

"Life-threatening" means that the patient was at immediate risk of death from the AE as it occurred, or it is suspected that use or continued use of the product would result in the patient's death. "Life-threatening" does not mean that had an AE occurred in a more severe form it might have caused death (eg, hepatitis that resolved without hepatic failure).

Hospitalisation

Outpatient treatment in an emergency room is not in itself a SAE, although the reasons for it may be (eg, bronchospasm, laryngeal oedema). Hospital admissions and/or surgical operations planned before or during a study are not considered AEs if the illness or disease existed before the patient was enrolled in the study, provided that it did not deteriorate in an unexpected way during the study.

Important medical event or medical treatment

Medical and scientific judgement should be exercised in deciding whether a case is serious in situations where important medical events may not be immediately life threatening or result in death, hospitalisation, disability or incapacity but may jeopardise the patient or may require medical treatment to prevent one or more outcomes listed in the definition of serious. These should usually be considered as serious.

Simply stopping the suspect drug does not mean that it is an important medical event; medical judgement must be used.

- Angioedema not severe enough to require intubation but requiring iv hydrocortisone treatment.
- Hepatotoxicity caused by paracetamol (acetaminophen) overdose requiring treatment with N-acetylcysteine.
- Intensive treatment in an emergency room or at-home for allergic bronchospasm.
- Blood dyscrasias (eg, neutropenia or anaemia requiring blood transfusion, etc.) or convulsions that do not result in hospitalisation.
- Development of drug dependency or drug abuse.

Intensity rating scale:

The grading scales found in the revised NCI CTCAE latest version will be utilised for all events with an assigned CTCAE grading. For those events without assigned CTCAE grades, the recommendation in the CTCAE criteria that converts mild, moderate and severe events into CTCAE grades should be used. A copy of the CTCAE can be downloaded from the Cancer Therapy Evaluation Programme website (http://ctep.cancer.gov). The applicable version of CTCAE should be described clearly.

It is important to distinguish between serious and severe AEs. Severity is a measure of intensity whereas seriousness is defined by the criteria in Appendix B 2. An AE of severe intensity need not necessarily be considered serious. For example, nausea that persists for several hours may be considered severe nausea, but not a SAE unless it meets the criteria shown in Appendix B 2. On the other hand, a stroke that results in only a limited degree of disability may be considered a mild stroke but would be a SAE when it satisfies the criteria shown in Appendix B 2.

B 3 A Guide to Interpreting the Causality Question

When making an assessment of causality consider the following factors when deciding if there is a "reasonable possibility" that an AE may have been caused by the drug.

- Time Course. Exposure to suspect drug. Has the patient actually received the suspect drug? Did the AE occur in a reasonable temporal relationship to the administration of the suspect drug?
- Consistency with known drug profile. Was the AE consistent with the previous knowledge of the suspect drug (pharmacology and toxicology) or drugs of the same pharmacological class? Or could the AE be anticipated from its pharmacological properties?
- De-challenge experience. Did the AE resolve or improve on stopping or reducing the dose of the suspect drug?
- No alternative cause. The AE cannot be reasonably explained by another aetiology such as the underlying disease, other drugs, other host or environmental factors.
- Re-challenge experience. Did the AE reoccur if the suspected drug was reintroduced after having been stopped? AstraZeneca would not normally recommend or support a re-challenge.
- Laboratory tests. A specific laboratory investigation (if performed) has confirmed the relationship.

In difficult cases, other factors could be considered such as:

- Is this a recognised feature of overdose of the drug?
- Is there a known mechanism?

Causality of "related" is made if following a review of the relevant data, there is evidence for a "reasonable possibility" of a causal relationship for the individual case. The expression "reasonable possibility" of a causal relationship is meant to convey, in general, that there are facts (evidence) or arguments to suggest a causal relationship.

The causality assessment is performed based on the available data including enough information to make an informed judgement. With no available facts or arguments to suggest a causal relationship, the event(s) will be assessed as "not related".

Causal relationship in cases where the disease under study has deteriorated due to lack of effect should be classified as no reasonable possibility.

B 4 Medication Error, Drug Abuse and Drug Misuse

For the purposes of this clinical study a medication error is an unintended failure or mistake in the treatment process for an IMP or AstraZeneca NIMP that either causes harm to the patient or has the potential to cause harm to the patient.

A medication error is not lack of efficacy of the drug, but rather a human or process related failure while the drug is in control of the study site staff or patient.

Medication error includes situations where an error:

- occurred.
- was identified and intercepted before the patient received the drug.
- did not occur, but circumstances were recognised that could have led to an error.

Examples of events to be reported in clinical studies as medication errors:

- Drug name confusion.
- Dispensing error eg, medication prepared incorrectly, even if it was not actually given to the patient.
- Drug not administered as indicated, for example, eg, wrong route, dose (error greater than +/- 10%), or wrong site of administration
- Drug not taken as indicated eg, tablet dissolved in water when it should be taken as a solid tablet.

- Drug not stored as instructed eg, kept in the refrigerator when it should be at room temperature.
- Wrong patient received the medication (excluding IRT/RTSM errors).
- Wrong drug administered to patient (excluding IRT/RTSM errors).

Examples of events that **do not** require reporting as medication errors in clinical studies:

- Errors related to or resulting from IRT/RTSM - including those which lead to one of the above listed events that would otherwise have been a medication error.
- Patient accidentally missed drug dose(s) eg, forgot to take medication.
- Accidental overdose (will be captured as an overdose).
- Patient failed to return unused medication or empty packaging.

Medication errors are not regarded as AEs, but AEs may occur as a consequence of the medication error.

Drug Abuse

For the purpose of this study, drug abuse is defined as the persistent or sporadic intentional, non-therapeutic excessive use of IMP/study intervention or AstraZeneca NIMP for a perceived reward or desired non-therapeutic effect.

Any events of drug abuse, with or without associated AEs, are to be captured and forwarded to the Data Entry Site (DES) using the Drug Abuse Report Form. This form should be used both if the drug abuse happened in a study participant or if the drug abuse regards a person not enrolled in the study (such as a relative of the study participant).

Examples of drug abuse include but are not limited to:

- The drug is used with the intent of getting a perceived reward (by the study participant or a person not enrolled in the study)
- The drug in the form of a tablet is crushed and injected or snorted with the intent of getting high.

Drug Misuse

Drug misuse is the intentional and inappropriate use (by a study participant) of IMP/study intervention or AstraZeneca NIMP for medicinal purposes outside of the authorised product information, or for unauthorised IMPs/study interventions or AstraZeneca NIMPs, outside the intended use as specified in the protocol, and includes deliberate administration of the product by the wrong route.

Events of drug misuse, with or without associated AEs, are to be captured and forwarded to the DES using the Drug Misuse Report Form. This form should be used both if the drug misuse happened in a study participant or if the drug misuse regards a person not enrolled in the study (such as a relative of the study participant).

Examples of drug misuse include but are not limited to:

- The drug is used with the intention to cause an effect in another person
- The drug is sold to other people for recreational purposes
- The drug is used to facilitate assault in another person
- The drug is deliberately administered by the wrong route
- The drug is split in half because it is easier to swallow, when it is stated in the protocol that it must be swallowed whole
- Only half the dose is taken because the study participant feels that they were feeling better when not taking the whole dose
- Someone who is not enrolled in the study intentionally takes the drug.

Appendix C Handling of Human Biological Samples

C 1 Chain of Custody

A full chain of custody is maintained for all samples throughout their lifecycle.

The Investigator at each centre keeps full traceability of collected biological samples from the patients while in storage at the centre until shipment or disposal (where appropriate) and records relevant processing information related to the samples whilst at the site.

The sample receiver keeps full traceability of the samples while in storage and during use until used or disposed of or until further shipment and keeps record of receipt of arrival and onward shipment or disposal.

AstraZeneca or delegated representatives will keep oversight of the entire life cycle through internal procedures, monitoring of study sites, auditing or process checks, and contractual requirements of external laboratory providers.

Samples retained for further use will be stored in the AstraZeneca-assigned biobanks or other sample archive facilities and will be tracked by the appropriate AstraZeneca Team during for the remainder of the sample life cycle.

If required, AstraZeneca will ensure that remaining biological samples are returned to the site according to local regulations or at the end of the retention period, whichever is the sooner.

C 2 Withdrawal of Informed Consent for Donated Biological Samples

If a patient withdraws consent specifically to the subsequent use of donated biological samples, the samples will be disposed of/destroyed/repatriated, and the action documented. If samples are already analysed, AstraZeneca is not obliged to destroy the results of this research. The patient will be presented with the option to opt out of the subsequent use of the donated samples during the withdrawal process. If the patient decides to opt out, then the donated samples will be disposed of. If the patient withdraws consent without opting out for the subsequent use of the donated samples, then the samples will be used as per protocol.

Following withdrawal of consent for biological samples, further study participation should be considered in relation to the withdrawal processes outlined in the informed consent.

The Investigator:

- Ensures patient's withdrawal of informed consent to the use of donated samples is highlighted immediately to AstraZeneca or delegate.
- Ensures that relevant human biological samples from that patient, if stored at the study site, are immediately identified, disposed of as appropriate, and the action documented.

- Ensures that the patient and AstraZeneca are informed about the sample disposal.

AstraZeneca ensures the organisation(s) holding the samples is/are informed about the withdrawn consent immediately and that samples are disposed of or repatriated as appropriate, and the action documented, and study site notified.

C 3 International Airline Transportation Association (IATA) 6.2 Guidance Document

LABELLING AND SHIPMENT OF BIOHAZARD SAMPLES

IATA (https://www.iata.org/whatwedo/cargo/dgr/Pages/download.aspx) classifies infectious substances into 3 categories: Category A, Category B or Exempt

Category A Infectious Substances are infectious substances in a form that, when exposure to it occurs, is capable of causing permanent disability, life-threatening or fatal disease in otherwise healthy humans or animals.
Category A pathogens are, for example, Ebola, Lassa fever virus. Infectious substances meeting these criteria which cause disease in humans or both in humans and animals must be assigned to UN 2814. Infectious substances which cause disease only in animals must be assigned to UN 2900.

Category B Infectious Substances are infectious Substances that do not meet the criteria for inclusion in Category A. Category B pathogens are, for example, Hepatitis A, C, D, and E viruses. They are assigned the following UN number and proper shipping name:

- UN 3373 – Biological Substance, Category B.
- are to be packed in accordance with UN3373 and IATA 650.

Exempt - Substances which do not contain infectious substances or substances which are unlikely to cause disease in humans or animals are not subject to these Regulations unless they meet the criteria for inclusion in another class.

- Clinical study samples will fall into Category B or exempt under IATA regulations.
- Clinical study samples will routinely be packed and transported at ambient temperature in IATA 650 compliant packaging. (https://www.iata.org/whatwedo/cargo/dgr/Documents/DGR-60-EN-PI650.pdf).
- Biological samples transported in dry-ice require additional dangerous goods specification for the dry-ice content.

Appendix D Optional [redacted] Initiative Sample

D 1 Use/Analysis of [redacted]

[redacted] variation may impact a patient's response to therapy, susceptibility to, and severity and PD. Variable response to therapy may be due to [redacted] determinants that impact drug absorption, distribution, metabolism, and excretion; mechanism of action of the drug; disease aetiology; and/or molecular subtype of the disease being treated. In addition, collection of [redacted] samples from populations with well described clinical characteristics may lead to improvements in the design and interpretation of clinical studies and, possibly, to [redacted] guided treatment strategies.

- AstraZeneca intends to collect and store [redacted] for [redacted] research to explore how [redacted] variations may affect clinical parameters, risk and prognosis of diseases, and the response to medications. This [redacted] research may lead to better understanding of diseases, better diagnosis of diseases or other improvements in health care and to the discovery of new diagnostics, treatments or medications. Therefore, where local regulations and IRB/IEC allow, a blood sample will be collected for [redacted] analysis from consenting patients.
- This optional [redacted] research may consist of the analysis of the structure of the patient's [redacted] ie, the entire [redacted]
- The results of [redacted] analyses may be reported in a separate study summary.
- The Sponsor will store the [redacted] samples in a secure storage space with adequate measures to protect confidentiality.
- The samples will be retained while research on study interventions continues but no longer than 15 years from the end of the study or other period as per local requirements.

D 2 Research Plan and Procedures

Selection of [redacted] research population

- All patients will be asked to participate in this [redacted] research. Participation is voluntary and if a patient declines to participate there will be no penalty or loss of benefit. The patient will not be excluded from any aspect of the main study.

Inclusion criteria

For inclusion in this [redacted] research, patients must fulfil all of the inclusion criteria described in the main body of the CSP and: Provide informed consent for the [redacted] Initiative sampling and analyses.

Exclusion criteria

- Exclusion from this [redacted] research may be for any of the exclusion criteria specified in the main study or any of the following:
  - Previous allogeneic bone marrow transplant.

– Transfusion of non-leukocyte depleted blood or blood component within 120 days of genetic sample collection.

Withdrawal of consent for [redacted] research

Patients may withdraw from this [redacted] research at any time, independent of any decision concerning participation in other aspects of the main study. Voluntary withdrawal will not prejudice further treatment. Procedures for withdrawal are outlined in Section 7.2 of the main CSP.

Collection of samples for [redacted] research

The blood sample for this [redacted] research will be obtained from the patients' pre-dose at the first dosing visit. Although DNA is stable, early sample collection is preferred to avoid introducing bias through excluding patients who may withdraw due to an AE. If for any reason the sample is not drawn at the first dosing visit, it may be taken at any visit until the last study visit. Only one sample should be collected per patient for [redacted] during the study.

Coding and storage of [redacted] samples

- The processes adopted for the coding and storage of samples for [redacted] analysis are important to maintain patient confidentiality. Samples may be stored for a maximum of 15 years from the end of the study (as defined in the protocol), after which they will be destroyed. [redacted] is a finite resource that is used up during analyses. Samples will be stored and used until no further analyses are possible or the maximum storage time has been reached.
- An additional second code will be assigned to the sample either before or at the time of [redacted] extraction replacing the information on the sample tube. Thereafter, the sample will be identifiable only by the second, unique number. This number is used to identify the sample and corresponding data at the AstraZeneca [redacted] laboratories, or at the designated organisation. No personal details identifying the individual will be available to any person (AstraZeneca employee or designated organisations working with the [redacted]
- The link between the patient enrolment/randomisation code and the second number will be maintained and stored in a secure environment, with restricted access at AstraZeneca or designated organisations. The link will be used to identify the relevant [redacted] samples for analysis, facilitate correlation of genotypic results with clinical data, allow regulatory audit, and permit tracing of samples for destruction in the case of withdrawal of consent.

Ethical and regulatory requirements

The principles for ethical and regulatory requirements for the study, including this [redacted] research component, are outlined in Appendix A.

Informed consent

The [redacted] component of this study is optional, and the patient may participate in other components of the main study without participating in this [redacted] component. To participate in the [redacted] component of the study the patient must sign and date both the consent form for the main study and the addendum for the [redacted] Initiative component of the study. Copies of both signed and dated consent forms must be given to the patient and the original filed at the study centre. The Principal Investigator(s) is responsible for ensuring that consent is given freely and that the patient understands that they may freely withdrawal from the [redacted] aspect of the study at any time.

Patient data protection

- AstraZeneca will not provide individual [redacted] results to patients, any insurance company, any employer, their family members, general physician unless required to do so by law.
- Extra precautions are taken to preserve confidentiality and prevent [redacted] data being linked to the identity of the patient. In exceptional circumstances, however, certain individuals might see both the [redacted] data and the personal identifiers of a patient. For example, in the case of a medical emergency, an AstraZeneca Physician or an Investigator might know a patient's identity and also have access to his or her [redacted] data. Regulatory authorities may require access to the relevant files, though the patient's medical information and the [redacted] files would remain physically separate.

Data management

- Any [redacted] data generated in this study will be stored at a secure system at AstraZeneca and/or designated organisations to analyse the samples.
- AstraZeneca and its designated organisations may share summary results (such as [redacted] differences from groups of individuals with a disease) from this [redacted] research with other researchers, such as hospitals, academic organisations or health insurance companies. This can be done by placing the results in scientific databases, where they can be combined with the results of similar studies to learn even more about health and disease. The researchers can only use this information for health-related research purposes. Researchers may see summary results, but they will not be able to see individual patient data or any personal identifiers.
- Some or all of the clinical datasets from the main study may be merged with the [redacted] data in a suitable secure environment separate from the clinical database.

Statistical methods

The number of patients that will agree to participate in the [redacted] research is unknown. It is therefore not possible to establish whether sufficient data will be collected to allow a formal statistical evaluation or whether only descriptive statistics will be generated. A SAP may be prepared where appropriate.

Appendix E Actions Required in Cases of Increases in Liver Biochemistry and Evaluation of Hy's Law

E 1 Introduction

This appendix describes the process to be followed in order to identify and appropriately report PHL cases and HL cases. It is not intended to be a comprehensive guide to the management of elevated liver biochemistries.

During the course of the study the Investigator will remain vigilant for increases in liver biochemistry. The Investigator is responsible for determining whether a patient meets potential PHL criteria at any point during the study.

All sources of laboratory data are appropriate for the determination of PHL and HL events; this includes samples taken at scheduled study visits and other visits including central and all local laboratory evaluations even if collected outside of the study visits; for example, PHL criteria could be met by an elevated ALT from a central laboratory **and/or** elevated TBL from a local laboratory.

The Investigator will also review AE data (for example, for AEs that may indicate elevations in liver biochemistry) for possible PHL events.

The Investigator participates, together with AstraZeneca clinical project representatives, in review and assessment of cases meeting PHL criteria to agree whether HL criteria are met. HL criteria are met if there is no alternative explanation for the elevations in liver biochemistry other than DILI caused by the study interventions.

The Investigator is responsible for recording data pertaining to PHL/HL cases and for reporting SAEs and AEs according to the outcome of the review and assessment in line with standard safety reporting processes.

E 2 Definitions

PHL

Aspartate aminotransferase or ALT $\geq 3 \times$ ULN together with TBL $\geq 2 \times$ ULN at any point during the study following the start of study interventions irrespective of an increase in alkaline phosphatase.

HL

Aspartate aminotransferase or ALT $\geq 3 \times$ ULN together with TBL $\geq 2 \times$ ULN, where no other reason, other than the study interventions, can be found to explain the combination of increases, eg, elevated ALP indicating cholestasis, viral hepatitis, another drug.

For PHL and HL the elevation in transaminases must precede or be coincident with (ie, on the same day) the elevation in TBL, but there is no specified timeframe within which the elevations in transaminases and TBL must occur.

E 3 Identification of Potential Hy's Law Cases

In order to identify cases of PHL it is important to perform a comprehensive review of laboratory data for any patient who meets any of the following identification criteria in isolation or in combination:

- ALT $\geq 3 \times$ ULN.
- AST $\geq 3 \times$ ULN.
- TBL $\geq 2 \times$ ULN.

Local laboratories being used

The Investigator will, without delay, review each new laboratory report and if the identification criteria are met will:

- Notify the AstraZeneca representative.
- Determine whether the patient meets PHL criteria (see Section E 2 Definitions within this Appendix for definition) by reviewing laboratory reports from all previous visits.
- Promptly enter the laboratory data into the laboratory eCRF.

E 4 Follow-up

E 4.1 Potential Hy's Law Criteria Not Met

If the patient does not meet PHL criteria the Investigator will:

- Perform follow-up on subsequent laboratory results according to the guidance provided in the CSP.

E 4.2 Potential Hy's Law Criteria Met

If the patient does meet PHL criteria the Investigator will:

- Notify the AstraZeneca representative who will then inform the central Study Team.
- Within 1 day of PHL criteria being met, the Investigator will report the case as an SAE of PHL; serious criterion "Important medical event" and causality assessment "yes/related" according to CSP process for SAE reporting.

- For patients that met PHL criteria prior to starting study interventions, the Investigator is not required to submit a PHL SAE unless there is a significant change# in the patient's condition.
- The Study Physician/Medical Scientist contacts the Investigator, to provide guidance, discuss and agree an approach for the study patient's follow-up (including any further laboratory testing) and the continuous review of data.
- Subsequent to this contact the Investigator will:
  - Monitor the patient until liver biochemistry parameters and appropriate clinical symptoms and signs return to normal or baseline levels, or as long as medically indicated. Completes follow-up SAE Form as required.
  - Investigate the aetiology of the event and perform diagnostic investigations as discussed with the Study Physician/Medical Scientist. This includes deciding which the tests available in the HL lab kit should be used.
  - Complete the 3 Liver eCRF Modules as information becomes available.

#**A "significant" change** in the patient's condition refers to a clinically relevant change in any of the individual liver biochemistry parameters (ALT, AST or TBL) in isolation or in combination, or a clinically relevant change in associated symptoms. The determination of whether there has been a significant change will be at the discretion of the Investigator, this may be in consultation with the Study Physician/Medical Scientist if there is any uncertainty.

E 5 Review and Assessment of Potential Hy's Law Cases

The instructions in this Section should be followed for all cases where PHL criteria are met.

As soon as possible after the biochemistry abnormality was initially detected, the Study Physician/Medical Scientist contacts the Investigator in order to review available data and agree on whether there is an alternative explanation for meeting PHL criteria other than DILI caused by the study interventions, to ensure timely analysis and reporting to health authorities within 15 calendar days from date PHL criteria was met. The AstraZeneca Global Clinical Lead or equivalent and Global Safety Physician will also be involved in this review together with other subject matter experts as appropriate.

According to the outcome of the review and assessment, the Investigator will follow the instructions below.

Where there is an agreed alternative explanation for the ALT or AST and TBL elevations, a determination of whether the alternative explanation is an AE will be made and subsequently whether the AE meets the criteria for a SAE:

- If the alternative explanation is **not** an AE, record the alternative explanation on the appropriate eCRF.
- If the alternative explanation is an AE/SAE: update the previously submitted PHL SAE and AE eCRFs accordingly with the new information (reassessing event term; causality and seriousness criteria) following the AstraZeneca standard processes.

If it is agreed that there is no explanation that would explain the ALT or AST and TBL elevations other than the study interventions:

- Send updated SAE (report term "Hy's Law") according to AstraZeneca standard processes.
  - The "Medically Important" serious criterion should be used if no other serious criteria apply.
  - As there is no alternative explanation for the HL case, a causality assessment of "related" should be assigned.

If, there is an unavoidable delay, of over 15 calendar days in obtaining the information necessary to assess whether or not the case meets the criteria for HL, then it is assumed that there is no alternative explanation until such time as an informed decision can be made:

- Provides any further update to the previously submitted SAE of PHL, (report term now "Hy's Law case") ensuring causality assessment is related to study interventions and seriousness criterion is medically important, according to CSP process for SAE reporting.
- Continue follow-up and review according to agreed plan. Once the necessary supplementary information is obtained, repeat the review and assessment to determine whether HL criteria are still met. Update the previously submitted PHL SAE report following CSP process for SAE reporting, according to the outcome of the review and amending the reported term if an alternative explanation for the liver biochemistry elevations is determined.

E 6 Actions Required When Potential Hy's Law Criteria are Met Before and After Starting Study Intervention

This section is applicable to patients who meet PHL criteria on study intervention, having previously met PHL criteria at a study visit prior to starting study intervention.

At the first on-study interventions occurrence of PHL criteria being met the Investigator will determine if there has been a **significant change** in the patient's condition compared with the last visit where PHL criteria were met.

- If there is no significant change no action is required.
- If there is a significant change, notify the AstraZeneca representative, who will inform the central Study Team, then follow the subsequent process described in Section E 4.2.

E 7 Actions Required for Repeat Episodes of Potential Hy's Law

This section is applicable when a patient meets PHL criteria on study interventions and has already met PHL criteria at a previous on study intervention visit.

The requirement to conduct follow-up, review and assessment of a repeat occurrence(s) of PHL is based on the nature of the alternative cause identified for the previous occurrence.

The investigator should determine the cause for the previous occurrence of PHL criteria being met and answer the following question:

Was the alternative cause for the previous occurrence of PHL criteria being met found to be the disease under study eg, chronic or progressing malignant disease, severe infection or liver disease?

If **No**: follow the process described in Section E 4.2 for reporting PHL as an SAE.

If **Yes**: Determine if there has been a significant change in the patient's condition compared with when PHL criteria were previously met.

- If there is no significant change no action is required.
- If there is a significant change follow the process described in Section E 4.2 for reporting PHL as an SAE.

E 8 Laboratory Tests

Suggested laboratory tests for local assessment of Hy's Law

Additional standard chemistry and coagulation tests	GGT<br>LDH<br>Prothrombin time<br>INR
Viral hepatitis	IgM anti-HAV<br>IgM and IgG anti-HBc
	HBsAg<br>HCV DNA[a]<br>IgM and IgG anti-HCV<br>HCV RNA[a]<br>IgM anti-HEV<br>HEV RNA

Other viral infections	IgM & IgG anti-CMV<br>IgM & IgG anti-HSV<br>IgM & IgG anti-EBV
Alcoholic hepatitis	Carbohydrate-deficient transferrin[b]
Autoimmune hepatitis	Antinuclear antibody<br>Anti-liver/kidney microsomal antibody<br>Anti-smooth muscle antibody
Metabolic diseases	Alpha-1-antitrypsin<br>Ceruloplasmin<br>Iron<br>Ferritin<br>Transferrin[b]<br>Transferrin saturation

[a] HCV RNA and HCV DNA are only tested when IgG anti-HCV is positive or inconclusive.

[b] Carbohydrate-deficient transferrin and transferrin are not available in China. Study teams should amend this list accordingly.

Abbreviations: CMV: cytomegalovirus; DNA: deoxyribonucleic acid; EBV: Epstein-Barr virus; GGT: gamma glutamyl transferase; HAV: hepatitis A virus; HBc: hepatitis B core antigen; HBsAg: hepatitis B surface antigen; HBV: hepatitis B virus; HCV: hepatitis C virus; HEV: hepatitis E virus; HSV: herpes simplex virus; IgG: immuno-globulin G; IgM: immuno-globulin M; INR: international normalised ratio; LDH: lactate dehydrogenase; RNA: ribonucleic acid.

E 9 References

Aithal et al 2011

Aithal GP, Watkins PB, Andrade RJ, Larrey D, Molokhia M, Takikawa H, et al. Case definition and phenotype standardization in drug-induced liver injury. Clin Pharmacol Ther 2011;89(6):806-15.

FDA Guidance 2009

Food and Drug Administration. Guidance for industry: Drug-induced liver injury: premarketing clinical evaluation. July 2009. Available from: URL: https://www.fda.gov/downloads/guidances/UCM174090.pdf. Accessed 08 October 2019.

Appendix F Guidelines for Evaluation of Objective Tumour Response Using RECIST 1.1 Criteria (Response Evaluation Criteria in Solid Tumours)

Introduction

This appendix details the implementation of RECIST 1.1 guidelines (Eisenhauer, et al. 2009). Investigator assessments will use the RECIST 1.1 guidelines described in this appendix.

Evaluation of Target Lesions

- **Complete response** - Disappearance of all target lesions. Any pathological lymph nodes (whether target or non-target) must have reduction in short axis to < 10 mm (the sum may not be "0" if there are target nodes).
- **Partial response** - At least a 30% decrease in the sum of the diameters of target lesions, taking as reference the baseline sum diameters.
- **Progressive of Disease** - At least a 20% increase in the sum of diameters of target lesions, taking as reference the smallest sum on study (this includes the baseline sum if that is the smallest on study). In addition to the relative increase of 20%, the sum must also demonstrate an absolute increase of at least 5 mm. (Note: the appearance of one or more new lesions is also considered PD.)
- **Stable Disease** - Neither sufficient shrinkage to qualify for PR nor sufficient increase to qualify for PD, taking as reference the smallest sum of diameters while on study.

Evaluation of Non-target Lesions

- **Complete response** - Disappearance of all non-target lesions and normalisation of tumour marker level. All lymph nodes must be non-pathological in size (< 10 mm short axis).
- **Non-CR/Non-PD** - Persistence of 1 or more non-target lesion(s) and/or maintenance of tumour marker level above the normal limits.
- **Progression of disease** - Unequivocal progression of existing non-target lesions will be defined as the overall level of substantial worsening in non-target disease such that, even in presence of SD or PR in target disease, the overall tumour burden has increased sufficiently to merit discontinuation of therapy. In the absence of measurable disease, change in non-measurable disease comparable in magnitude to the increase that would be required to declare PD for measurable disease. Examples include an increase in a pleural effusion from 'trace' to 'large,' an increase in lymphangitic disease from localized to widespread.

Appearance of New Lesions

The appearance of new lesions is considered PD according to RECIST v1.1. Scintigraphy for confirmation of new bone lesions is acceptable but by itself does not substantiate PD. Considering the unique response kinetics that have been observed with immunotherapy, new lesions can nonetheless derive clinical benefit (Borghaei, et al. 2015).

Evaluation of Overall Response

Table 14 provides overall responses for all possible combinations of tumour responses in target and non-target lesions with or without the appearance of new lesions.

Table 14 RECIST 1.1 Overall Visit Response

Target Lesions	Non-Target Lesions	New Lesions	Overall Response
Complete response	Complete response (or no non-target lesion)	No	Complete response
No target lesion [a]	Complete response	No	Complete response
Complete response	Not evaluable [b]	No	Partial response
Complete response	Non-complete response / non-progressive disease	No	Partial response
Partial response	Non-progressive disease and not evaluable (or no non-target lesion) [b]	No	Partial response
Stable disease	Non-progressive disease and not evaluable (or no non-target lesion) [b]	No	Stable disease
Not all evaluated	Non-progressive disease	No	Not evaluable
No target lesion [a]	Not all evaluated	No	Not evaluable
No target lesion [a]	Non-complete response / non-progressive disease	No	Non-complete response / non-progressive disease
Progressive disease	Any	Yes/No	Progressive disease
Any	Progressive disease	Yes/No	Progressive disease
Any	Any	Yes	Progressive disease
No target lesion [a]	Unequivocal progressive disease	Yes/No	Progressive disease

a Defined as no target lesion at baseline.
b Not evaluable is defined as either when no or only a subset of lesion measurements are made at an assessment.

F 1 References

Borghaei, et al. 2015
Borghaei H, Paz-Ares L, Horn L, Spigel DR, Steins M, Ready NE, et al. Nivolumab versus Docetaxel in Advanced Nonsquamous Non-Small-Cell Lung Cancer. N Engl J Med. 2015 Oct 22;373(17):1627-39

Eisenhauer, et al. 2009
Eisenhauer EA, Therasse P, Bogaerts J, Schwartz LH, Sargent D, Ford R, et al. New response evaluation criteria in solid tumours: revised RECIST guideline (version 1.1). Eur J Cancer 2009;45(2):228-47.

Appendix G Contraception Requirements

Contraception requirements for this study are as follows.

G 1 Female Patients

Women not of childbearing potential are defined as those who are surgically sterile (ie, bilateral salpingectomy, bilateral oophorectomy, or complete hysterectomy) or who are post-menopausal.

Women will be considered post-menopausal if they have been amenorrhoeic for 12 months without an alternative medical cause. The following age-specific requirements apply:

- Women < 50 years of age would be considered post-menopausal if they have been amenorrhoeic for 12 months or more following cessation of all hormonal replacement therapy and if they have luteinising hormone and follicle-stimulating hormone levels in the post-menopausal range for the institution.
- Women ≥ 50 years of age would be considered post-menopausal if they have been amenorrhoeic for 12 months or more following cessation of all hormonal replacement therapy, or had radiation-induced menopause with last menses > 1 year ago, or had chemotherapy-induced menopause with last menses > 1 year ago.
- Women who are surgically sterile (ie, bilateral salpingectomy, bilateral oophorectomy, or complete hysterectomy) are eligible.

Women of childbearing potential who are not totally sexually abstinent (ie, refraining from heterosexual intercourse during the entire period of risk associated with study interventions) and intend to be sexually active with a non-sterilised male partner must use at least 1 highly effective method of contraception (Table 15) from the time of screening throughout the total duration of the drug treatment and and for the following period after receiving the last dose of study interventions:

- Durvalumab or volrustomig: 90 days
- Oleclumab, monalizumab, or AZD0171: 180 days
- Dato-DXd: 210 days
- Rilvegostomig: 60 days

For the chemotherapy agents follow the local prescribing information relating to contraception, the time limits for such precautions, and any additional restrictions for the agents administered. For patients receiving more than one study intervention, the longest washout period must be followed.

Non-sterilised male partners of a woman of childbearing potential must use a male condom plus spermicide (condom alone in countries where spermicides are not approved) throughout this period. Cessation of birth control after this point should be discussed with a responsible physician. Periodic abstinence, the rhythm method, and the withdrawal method are not acceptable methods of contraception. Total sexual abstinence is an acceptable method provided it is the usual lifestyle of the patient. Female patients should refrain from breastfeeding throughout this period. Preservation of ova should be considered prior to enrolment in this study.

Follow the local labels or national guidance related to contraception for the study interventions including the length of the contraception periods, and any additional restrictions.

G 2 Male Patients with a Female Partner of Childbearing Potential

Non-sterilised male patients (including males sterilised by a method other than bilateral orchidectomy, eg, vasectomy) who intend to be sexually active with a female partner of childbearing potential must be using an acceptable method of contraception such as male condom plus spermicide (condom alone in countries where spermicides are not approved) from the time of screening throughout the total duration of the study and for the following period after the last dose of study interventions:

- Durvalumab or volrustomig: 90 days
- Oleclumab, monalizumab, or AZD0171: 180 days
- Dato-DXd: 120 days
- Rilvegostomig: 60 days

For the chemotherapy agents follow the local prescribing information relating to contraception, the time limits for such precautions, and any additional restrictions for the agents administered. For patients receiving more than one study intervention, the longest washout period must be followed to prevent pregnancy in a partner. Periodic abstinence, the rhythm method, and the withdrawal method are not acceptable methods of contraception. Preservation of sperm should be considered prior to enrolment in this study. Total sexual abstinence is an acceptable method provided it is the usual lifestyle of the patient.

Vasectomised (ie, sterile) males are considered fertile and should still use a male condom plus spermicide as indicated above during the clinical study.

Even if the female partner is pregnant, male patients should still use a condom plus spermicide (where approved), as indicated above during the clinical study, if there is a concern about damaging the developing foetus from drug in ejaculate.

Female partners (of childbearing potential) of male patients must also use a highly effective

method of contraception throughout this period (Table 15).

G 3 Highly Effective Methods of Contraception

Highly effective methods of contraception, defined as one that results in a low failure rate (ie, less than 1% per year) when used consistently and correctly, are described in Table 15. Note that some contraception methods are not considered highly effective (eg, male or female condom with or without spermicide; female cap, diaphragm, or sponge with or without spermicide; non copper containing intrauterine device; progestogen-only oral hormonal contraceptive pills where inhibition of ovulation is not the primary mode of action [excluding Cerazette/desogestrel which is considered highly effective]; and triphasic combined oral contraceptive pills).

Patients receiving chemotherapy should follow the local prescribing information relating to contraception, the time limits for such precautions, and any additional restrictions for agents administered.

Table 15 Highly Effective Methods of Contraception (< 1% Failure Rate)

Barrier/intrauterine methods	Hormonal Methods
• Copper T intrauterine device<br>• Levonorgestrel-releasing intrauterine system (eg, Mirena®)[a]	• Injection: Medroxyprogesterone injection (eg, Depo-Provera®)<br>• Implants: Etonogestrel-releasing implants (eg, Implanon® or Norplant®)<br>• Intravaginal devices: Ethinylestradiol/etonogestrel-releasing intravaginal devices (eg, NuvaRing®)<br>• Combined pill: Normal and low dose combined oral contraceptive pill<br>• Patch: Norelgestromin/ethinylestradiol-releasing transdermal system (eg, Ortho Evra®)<br>• Mini pill: Progesterone based oral contraceptive pill using desogestrel: Cerazette® is currently the only highly effective progesterone-based pill

[a] This is also considered a hormonal method.

Appendix H Concomitant Medications

H 1 Prohibited and Permitted Concomitant Medications/Therapies

Prohibited Concomitant Medications

Prohibited concomitant medications/therapies are described in Table 16.

Table 16 Prohibited medications/therapies

Prohibited medication/class of drug/therapy	Usage
Any investigational anticancer therapy other than those under investigation in this study	Should not be given concomitantly while the patient is on study interventions
Immune-mediated therapy including, but not limited to, other anti-CTLA-4, anti-PD-1, anti-PD-L1, and anti-PD-L2 antibodies and agents targeting the adenosine pathway (eg, anti-CD73, anti-A2AR, anti-CD39) and anti-NKG2A, anti-HLA-E and anti-LIF agents, treatment with a TROP2 targeting ADC or with another ADC containing a chemotherapy agent that inhibits TOP1, other than those under investigation in this study	Should not be given concomitantly while the patient is on study interventions
Any concurrent chemotherapy, radiotherapy, immunotherapy, or biologic or hormonal therapy for cancer treatment other than those under investigation in this study	Should not be given concomitantly while the patient is on study treatment. (Concurrent use of hormones for non-cancer-related conditions [eg, insulin for diabetes and hormone replacement therapy] is acceptable.
Live attenuated vaccines	Should not be given during the study through 30 days after the last dose of study interventions. Local guidance should be consulted to determine the acceptable timeframe for vaccine administration following chemotherapy
Immunosuppressive medications including, but not limited to, systemic corticosteroids at physiological doses exceeding 10 mg/day of prednisone or equivalent, methotrexate, azathioprine, and tumour necrosis factor-α blockers	Should not be given concomitantly or used for premedication prior to the I-O infusions. The following are allowed exceptions:<br>• Use of immunosuppressive medications for the management of study interventions-related AEs<br>• Short-term premedication for patients receiving chemotherapy or Dato-DXd where the prescribing information for the agent requires the use of steroids for documented hypersensitivity reactions or major toxicities management<br>• Use in patients with contrast allergies.<br>• Use of intranasal, inhaled, topical steroids, or local steroid injections (eg, intra articular injection) is permitted.

Prohibited medication/class of drug/therapy	Usage
	• A temporary period (≤ 14 days) of steroids will be allowed if clinically indicated and considered to be essential for the management of non-immunotherapy related events experienced by the patient (eg, chronic obstructive pulmonary disease, radiation, nausea[a], etc.)
Herbal and natural remedies that may have immune-modulating effects	Should not be given concomitantly unless agreed by the Sponsor

[a] Note: Alternative anti-emetic pre-medication should be prioritised in place of steroids (eg, 5-HT3 inhibitors, neurokinin inhibitors). Where steroid pre-medication is utilised, steroid doses should be administered in line with the Multinational Association of Supportive Care in Cancer guidelines (Roila et al, 2016) or according to local guidelines.
Note: Use of phenytoin and fosphenytoin is not recommended in conjunction with carboplatin, according to carboplatin summary of product characteristics.

Abbreviations: ADC: antibody-drug conjugate; AE: adverse event; CD73: cluster of differentiation 73; CTLA-4: Cytotoxic T-lymphocyte-associated antigen 4; Dato-DXd: datopotamab deruxtecan; HLA-E: HLA class I histocompatibility antigen, alpha chain E; I-O: immune-oncology; LIF: leukaemia inhibitory factor; NKG2a: natural killer group 2a; PD-1: programmed cell death 1; PD-L1: programmed cell death ligand-1; TOP1: topoisomerase 1; TROP2: human trophoblast cell-surface antigen 2; 5HT-3: 5-hydroxytryptamine 3.

Permitted Concomitant Medications

Investigators may prescribe concomitant medications or treatments deemed necessary to provide adequate prophylactic or supportive care except for those medications identified as prohibited in Table 16.

For patients in the volrustomig or rilvegostomig arms, Investigators may prescribe premedication for prevention of IRRs in patients who have Grade 2 or recurrent Grade 1 IRRs related to study treatment. Such premedication may include a combination of acetaminophen, H1 blocker (eg, diphenhydramine, chlorpheniramine) and H2 blocker (eg, famotidine, cimetidine). If necessary, a steroid premedication may be added (eg, dexamethasone 8 to 20 mg by mouth or IV; weak potency steroids such as hydrocortisone should be avoided).

Premedication is required prior to any dose of Dato-DXd and must include antihistamines and antipyretics, preferably acetaminophen, with or without glucocorticoids. If there are any signs or symptoms of a Grade 1 or 2 IRR, the infusion of Dato-DXd must be either slowed down or interrupted based on severity of the IRR. If the IRR is Grade 3 or 4, or if there are any signs of anaphylaxis, the infusion of Dato-DXd must be discontinued.

Colony-stimulating factors are permitted at the discretion of the Investigator based on ASCO (Roila et al, 2016, Smith, et al. 2006) and ESMO (Crawford, et al 2010) guidelines. G-CSF or similar agents are recommended as primary prophylaxis to chemotherapy agents and are strongly recommended following Grade 3 or 4 neutropenia of duration > 5 days or following any incidence of febrile neutropenia.

Antiemetic therapy is permitted and if administered should follow the Multinational Association of Supportive Care in cancer and ASCO, or institutional guidelines.

Per the paclitaxel label, caution should be exercised when paclitaxel is administered concomitantly with known substrates or inhibitors of the cytochrome P450 isoenzymes CYP2C8 and CYP3A4.

H 2 References

Crawford, et al 2010
Crawford. J, Caserta. C, and Roila. F. Hematopoietic growth factors: ESMO clinical practice guidelines for the applications. Annals of Oncology, 2010, 21(Suppl. 5), v248–v251.

Roila et al, 2016
Roila F, Molassiotis A, Herrstedt J, et al. 2016 MASCC and ESMO guideline update for the prevention of chemotherapy- and radiotherapy-induced nausea and comiting and of nausea and vomiting in advanced cancer patients. Ann Oncol 2016;27(5):v119-33. doi: 10.1093/annonc/mdw270

Smith, et al. 2006
Smith TJ, Khatcheressian J, Lyman GH, Ozer H, Armitage JO, Balducci L, et al. 2006 update of recommendations for the use of white blood cell growth factors: an evidence-based clinical practice guideline. J Clin Oncol. 2006 Jul 1;24(19):3187-205

Appendix I Clavien-Dindo Classification of Surgical Complications

Clavien-Dindo assessment will be utilised for grading post-operative complications for collection of AEs. AEs will be reported by the patient (or, when appropriate, by a caregiver, surrogate, or the patient's legally authorised representative).

The Investigator and any designees are responsible for detecting, documenting, and recording events that meet the definition of an AE or SAE. The following classification will be used:

Table 17 Classification of Surgical Complications

Grade	Definitions
1	Any deviation from the normal post-operative course without the need for pharmacological treatment or surgical, endoscopic, and radiological interventions.<br>Allowed therapeutic regimens are: drugs as antiemetics, antipyretics, analgesics, diuretics, electrolytes, and physiotherapy. This grade also includes wound infections opened at the bedside.
2	Requiring pharmacological treatment with drugs other than such allowed for grade 1 complications. Blood transfusions and total parenteral nutrition are also included.
3	Requiring surgical, endoscopic, or radiological intervention<br>• 3a: Intervention not under general anaesthesia.<br>• 3b: Intervention under general anaesthesia.
4	Life threatening complications (including CNS complications[a]) requiring intensive care/intensive care unit management<br>• 4a: Single organ dysfunction (including dialysis).<br>• 4b: Multiorgan dysfunction.
5	Death of a patient.

[a] Brain haemorrhage, ischemic stroke, subarrachnoidal bleeding, but excluding transient ischaemic attacks.
Abbreviations: CNS: central nervous system; IC: intermediate care; ICU: intensive care unit.
Source: Dindo et al, 2004

I 1 References

Dindo et al, 2004
Dindo D, Demartines N, Clavien P. Classification of surgical complications: a new proposal with evaluation in a cohort of 6336 patients results of a survey. Ann Surg 2004;240(2):205-213

Appendix J Changes related to mitigation of study disruptions due to cases of civil crisis, natural disaster, or public health crisis

Note: Changes below should be implemented only during study disruptions due to any of or a combination of civil crisis, natural disaster, or public health crisis (eg, during quarantines and resulting site closures, regional travel restrictions, and considerations if site personnel or study patients become infected with SARS-CoV-2 or similar pandemic infection) during which patients may not wish to or may be unable to visit the study site for study visits. These changes should only be implemented if allowable by local/regional guidelines and following agreement from the Sponsor.

J 1 Reconsent of Study Patients During Study Interruptions

During study interruptions, it may not be possible for the patients to complete study visits and assessments on-site and alternative means for carrying out the visits and assessments may be necessary, eg, remote visits. Reconsent should be obtained for the alternative means of carrying out visits and assessments and should be obtained prior to performing the procedures described in Sections J 2 to J 8. Local and regional regulations and/or guidelines regarding reconsent of study patients should be checked and followed. Reconsent may be verbal if allowed by local and regional guidelines (note: in the case of verbal reconsent the informed consent form should be signed at the patient's next contact with the study site).

Visiting the study sites for the sole purpose of obtaining reconsent should be avoided. Refer to the Study Instruction Manual for Mitigation Due to Civil Crisis, Natural Disaster, or Public Health Crisis for step-by-step guidance.

Refer to the Study Instruction Manual for Mitigation Due to Civil Crisis, Natural Disaster, or Public Health Crisis for step-by-step guidance.

J 2 Rescreening of Patients to Reconfirm Study Eligibility

Additional rescreening for screen failure due to study disruption can be performed in previously screened patients. The Investigator should confirm this with the designated Study Physician/Medical Scientist.

In addition, during study disruption there may be a delay between confirming eligibility of a patient and either enrolment into the study or commencing of dosing with study interventions. If this delay is outside the screening window specified in Section 5.1, the patient will need to be rescreened to reconfirm eligibility before commencing study procedures. This will provide another opportunity to re-screen a patient in addition to that detailed in Section 5.1. The procedures detailed in Section 5.1 and Section 5.4 must be undertaken to confirm eligibility using the same randomisation number as for the patient.

J 3 Home or Remote Visit to Replace On-site Visit (where applicable)

A qualified HCP from the study site or TPV service may visit the patient's home or other remote location as per local SOPs, as applicable. Supplies will be provided for a safe and efficient visit. The qualified HCP will be expected to collect information per the CSP. If applicable, assessments may be performed according to the revised SoA in the Study Instruction Manual for Mitigation Due to Civil Crisis, Natural Disaster, or Public Health Crisis.

J 4 Telemedicine Visit to Replace On-site Visit (where applicable)

In this appendix and the associated Study Instruction Manual for Mitigation Due to Civil Crisis, Natural Disaster, or Public Health Crisis, the term telemedicine visit refers to remote contact with the patients using telecommunications technology including phone calls, virtual or video visits, and mobile health devices.

During a civil crisis, natural disaster, or public health crisis, on-site visits may be replaced by a telemedicine visit if allowed by local/regional guidelines. Having a telemedicine contact with the patients will allow AEs, concomitant medication, add other information including efficacy data where relevant to be collected according to study requirements to be reported and documented. If applicable, safety procedures and blood sample collection may be performed according to the revised SoA in the Study Instruction Manual for Mitigation Due to Civil Crisis, Natural Disaster, or Public Health Crisis.

J 5 At-home or Remote Location Study Intervention Administration Instructions

If a site visit is not possible, at-home or remote location administration of study interventions may be performed by a qualified HCP, provided this is acceptable within local regulation/guidance, or by the patient or his/her caregiver. The option of at-home or remote location study interventions administration ensures patients safety in cases of a pandemic where patients may be at increased risk by traveling to the site/clinic. This will also minimise interruption of study interventions administration during other study disruptions, eg, site closures due to natural disaster.

J 6 At-home or Remote Location Study Intervention Administration by a Qualified HCP or TPV Service

A qualified HCP from the study site or TPV service may administer the study interventions at the patient's home or other remote location according to the CSP and the Study Instruction Manual for Mitigation Due to Civil Crisis, Natural Disaster, or Public Health Crisis, and if allowed by local SOPs, as applicable. All necessary supplies and instructions for administration and documentation of study interventions administration will be provided.

Additional information related to the visit can be obtained via a telemedicine or home visit.

J 7 At-home or Remote Location Study Interventions Administration by the Patient or His/her Caregiver

Prior to at-home or remote location study interventions administration, the Investigator must assess the patient or his/her caregiver to determine whether they are appropriate for at-home or remote location administration of study interventions. Once the patient or his/her caregiver is deemed appropriate for at-home or remote location administration, he/she must receive appropriate training. All necessary supplies and instructions for administration and documentation of study interventions administration will be provided. More information related to the visit can be obtained via a telemedicine or home/remote visit.

J 8 Data Capture During Telemedicine or Home/remote Visits

Data collected during telemedicine or home/remote visits will be captured by the qualified HCP from the study site or TPV service in the source documents or by the patient themselves.

Refer to the Study Instruction Manual for Mitigation Due to Civil Crisis, Natural Disaster, or Public Health Crisis for step-by-step guidance.

Appendix K Arm 1: Oleclumab + Durvalumab + Platinum Doublet Chemotherapy (Neoadjuvant Treatment) Followed by Oleclumab + Durvalumab (Adjuvant Treatment)

This appendix provides information for patients randomised to Arm 1.

K 1 Schedule of Activities

Table K18 Schedule of Activities for Neoadjuvant Treatment for Patients Randomised to Arm 1

	C1	C2	C3 and C4	Pre-surgical assessments	Surgery	For details, see CSP Section or Appendix
**Week**	**0**	**Q3W + 3 days [a]**		**Within 30 days before surgery**	**Within 40 days of the last dose of study interventions**	
**Day**	**1 [b]**	**q21d + 3 days [a]**				
**Study procedures**						
Physical examination (full)				X		Section 8.2.2
Targeted physical examination (based on symptoms)	X	X	X			Section 8.2.2
Vital signs [c]	X	X	X	X		Section 8.2.3
ECHO/MUGA		As clinically indicated				Section 8.2.5
ECG [d]	As clinically indicated			X		Section 8.2.4
Concomitant medications	X	X	X	X		Section 6.5
Contrast-enhanced CT/MRI scan of the chest and abdomen (including adrenal glands) and PET scan		X (CT only at Cycle 2)		X [e]		Section 6.1.3 and Section 8.1
Pulmonary function testing and cardiac risk assessment				X [f]		Section 8.2.7
Eligibility criteria				X (pre-surgery conditions)		Section 5 and Section 6.1.3

	C1	C2	C3 and C4	Pre-surgical assessments	Surgery	For details, see CSP Section or Appendix
**Week**	**0**	**Q3W + 3 days [a]**		**Within 30 days before surgery**	**Within 40 days of the last dose of study interventions**	
**Day**	**1 [b]**	**q21d + 3 days [a]**				
**Laboratory assessments**						
Clinical chemistry [g]	X [h]	X	X	X		Section 8.2.1
Cortisol [g]				X		Section 8.2.1
Haematology [g]	X [h]	X	X	X		Section 8.2.1
aPTT and INR	As clinically indicated			X		Section 8.2.1
TSH (reflex free T3 or Total T3, or free T4) [i]	X [j]	X	X			Section 8.2.1
Urinalysis	As clinically indicated					Section 8.2.1
Hepatitis B and C	As clinically indicated					Section 8.2.1
Pregnancy test [k]	X	X	X	X		Section 8.2.1
**Pharmacokinetics**						
Durvalumab PK sample [l]	X (pre-dose) X (post-dose)	X (pre-dose)	X (C4) (pre-dose) X (C4) (post-dose)			Section 8.5.1
Oleclumab PK sample [l]	X (pre-dose) X (post-dose)	X (pre-dose)	X (C4) (pre-dose) X (C4) (post-dose)			Section 8.5.1
**Monitoring**						
WHO/ECOG performance status	X	X	X	X		Section 8.2.6
AE/SAE assessment	X	X	X	X		Section 8.3

	C1	C2	C3 and C4	Pre-surgical assessments	Surgery	For details, see CSP Section or Appendix
**Week**	**0**	**Q3W + 3 days [a]**		**Within 30 days before surgery**	**Within 40 days of the last dose of study interventions**	
**Day**	**1 [b]**	**q21d + 3 days [a]**				
**Pre-treatment medication**						
Folic acid/vitamin B12 [m]	Continue in line with local practice					Section 6.1.1
**Study interventions administration [n]**						
Oleclumab [o]	X	X	X			Section 6.1.1
Durvalumab	X	X	X			Section 6.1.1
Chemotherapy [p]	X	X	X			Section 6.1.1
**Other assessments and assays**						
Durvalumab immunogenicity assessment (ADA sampling to identify ADA responses in patient circulation) [l]	X (pre-dose)	X (pre-dose)	X (C4) (pre-dose)			Section 8.5.2
Oleclumab immunogenicity assessment (ADA sampling to identify ADA responses) [l]	X (pre-dose)	X (pre-dose)	X (C4) (pre-dose)			Section 8.5.2
ctDNA [l]	X (pre-dose)	X (pre-dose)	X (pre-dose)	X		Section 8.6.3 and Section 8.7.1
Mandatory tumour and lymph nodes specimen					X	Section 8.6.1 and Section 8.6.2
Whole blood for Gene Expression [l]	X (pre-dose)	X (pre-dose)		X		Section 8.6.3
Serum samples for circulating soluble factors [l]	X (pre-dose)	X (pre-dose)		X		Section 8.6.3
Plasma samples for circulating soluble factors [l]	X (pre-dose)	X (pre-dose)		X		Section 8.6.3

	C1	C2	C3 and C4	Pre-surgical assessments	Surgery	For details, see CSP Section or Appendix
**Week**	**0**	**Q3W + 3 days [a]**		**Within 30 days before surgery**	**Within 40 days of the last dose of study interventions**	
**Day**	**1 [b]**	**q21d + 3 days [a]**				
Whole blood for DNA analyses [l]	X (pre-dose)					Section 8.6.3
Whole blood for PBMC [l]	X (pre-dose)	X (pre-dose)		X		Section 8.6.3
[redacted] Initiative optional, exploratory [redacted] blood sample [q]	X					Section 8.7.2 and Appendix D
**Efficacy evaluation**						
mPR and pCR determined by central pathology review					X	Section 8.1
RECIST 1.1 tumour assessments on CT and/or MRI scans	A RECIST 1.1 tumour assessment is performed after Cycle 2 and upon completion of the neoadjuvant period, prior to surgery.					Section 8.1.1 and Appendix F

a Subsequent time between 2 consecutive doses cannot be less than 21 days, based on the half-life of durvalumab.
b Every effort should be made to minimise the time between randomisation and starting treatment (ie, within 1 day and not more than 3 days of randomisation).
c Body weight is recorded at each visit along with vital signs.
d Any clinically significant abnormalities detected require triplicate ECG results.
e A CT and PET scan should be performed pre-surgery.
f Pulmonary function testing may be repeated at Investigator's or surgeon's discretion prior to surgery to inform fitness for surgery.
g Serum or plasma clinical chemistry (including LFT monitoring) and haematology may be performed more frequently based on the local clinical practice or the Investigator's discretion, or if clinically indicated. Results for LFTs, electrolytes, full blood count, and creatinine must be available before commencing an infusion (within 3 days) and reviewed by the treating physician or Investigator prior to dosing. Cortisol testing should be performed at the pre surgical visit.
h If screening clinical chemistry and haematology assessments are performed within 3 days prior to Day 1 (first infusion day), they do not need to be repeated on Day 1.
i Free T3 or Total T3, or free T4 will only be measured if TSH is abnormal or if there is clinical suspicion of an AE related to the endocrine system.
j If TSH is measured within 14 days prior to Day 1 (first infusion day), it does not need to be repeated on Day 1.

[k] For women of childbearing potential only. A urine or serum pregnancy test is acceptable. Women of childbearing potential are required to have a pregnancy test within 3 days prior to the first dose of study interventions and then Q3W prior to study interventions. Pregnancy test may occur on Day 1, but results must be available and reviewed by the treating physician or Investigator prior to commencing an infusion.

[l] Pre-dose collection: within 60 minutes prior to administration of any study intervention. Post-dose collection (durvalumab, oleclumab): end-of-infusion collection to be taken within 5 hours after the end of the respective study interventions infusion.

[m] To be administered in line with local practice for patients with non-squamous tumours who will receive pemetrexed.

[n] The following sequence should be used for treatment administration in days when all or more than one agent is administered: Oleclumab > durvalumab > platinum doublet chemotherapy.

[o] Oleclumab will be administered at 3000 mg IV, Day 1 of each 21-day cycle. Refer to Section 8.3.15 for TMGs.

[p] Platinum doublet chemotherapy (Investigator's choice of one of the regimens listed in Section 6.1.1).

[q] The sample for [redacted] research will be obtained on Day 1 pre-dose (at or after randomisation). If, for any reason, the sample is not drawn on Day 1, it may be taken at any visit until the last study visit. Only 1 sample should be collected per patient for [redacted] analysis during the study.

Note: Each cycle during the neoadjuvant treatment is 21 days (3 weeks) in duration, unless dosing needs to be held for toxicity reasons.

Note: All assessments on treatment days are to be performed prior to infusion, unless otherwise indicated.

Note: Surgery is expected within 40 days from the last dose of study interventions. Cases where surgery cannot be completed within 40 days from the last dose of study interventions should be discussed with the Study Physician.

Abbreviations: ADA: Anti-drug antibody; AE: Adverse event; aPTT: Activated partial thromboplastin time; C: Cycle; CSP: Clinical study protocol; CT: Computed tomography; ctDNA: Circulating tumour DNA; DNA: Deoxyribonucleic acid; ECG Electrocardiogram; ECHO: Echocardiogram; ECOG: Eastern Cooperative Oncology Group; INR: International normalised ratio; IV: Intravenous; LFT: Liver function test; mPR: Major pathological response; MRI: Magnetic resonance imaging; MUGA: multigated acquisition scans; PBMC: Peripheral blood mononuclear cells; pCR: Pathological complete response; PET: Positron emission tomography; PK: Pharmacokinetic(s); q21d: Every 21 days; Q3W: Every 3 weeks; RECIST: Response Evaluation Criteria in Solid Tumours; SAE: Serious adverse event; T3: Triiodothyronine; T4: Thyroxine; TMGs: Toxicity management guidelines; TSH: Thyroid-stimulating hormone; WHO: World Health Organisation.

Table K19 Schedule of Activities for Adjuvant Treatment for Patients Randomised to Arm 1

	Post-surgery, prior to starting treatment	C1	C2	C3 to C12	For details, see CSP Section or Appendix
Week	Within 10 weeks of surgery [a]	0	4	Q4W ± 3 days	
Day		1	29 ± 3	q28d ± 3 days	
**Study procedures**					
Physical examination (full)		X			Section 8.2.2
Targeted physical examination (based on symptoms)			X	X	Section 8.2.2
Vital signs [b]		X	X	X	Section 8.2.3
ECHO/MUGA		As clinically indicated			Section 8.2.5
ECG [c]		X	As clinically indicated		Section 8.2.4
Concomitant medications	X	X	X	X	Section 6.5
Post-surgery conditions	X				Section 6.1.3
**Laboratory assessments**					
Clinical chemistry [d]		X [d]	X [d]	X [d]	Section 8.2.1
Haematology [d]		X [d]	X [d]	X [d]	Section 8.2.1
aPTT and INR	As clinically indicated				Section 8.2.1
TSH (reflex free T3 or Total T3, or free T4) [e]		X	X	X	Section 8.2.1
Urinalysis		X	As clinically indicated		Section 8.2.1
Hepatitis B and C		As clinically indicated			Section 8.2.1
Pregnancy test [f]		X	X	X	Section 8.2.1

	Post-surgery, prior to starting treatment	C1	C2	C3 to C12	For details, see CSP Section or Appendix
**Week**	**Within 10 weeks of surgery** [a]	**0**	**4**	**Q4W ± 3 days**	
**Day**		**1**	**29 ± 3**	**q28d ± 3 days**	
**Pharmacokinetics**					
Durvalumab PK sample [g]		X (pre-dose) X (post-dose)	X (pre-dose)	X (C4) (pre-dose)	Section 8.5.1
Oleclumab PK sample [g]		X (pre-dose) X (post-dose)	X (pre-dose)	X (C4, C8 and C12) (pre-dose)	Section 8.5.1
**Monitoring**					
WHO/ECOG performance status		X	X	X	Section 8.2.6
AE/SAE assessment	X [h]	X	X	X	Section 8.3
**Study interventions administration** [i]					
Oleclumab [j]		X	X	X	Section 6.1.1
Durvalumab		X	X	X	Section 6.1.1
**Other assessments and assays**					
Durvalumab immunogenicity assessment (ADA sampling to identify ADA responses in patient circulation) [g]		X (pre-dose)	X (pre-dose)	X (C4 and C12) (pre-dose)	Section 8.5.2
Oleclumab immunogenicity assessment (ADA sampling to identify ADA responses) [g]		X (pre-dose)	X (pre-dose)	X (C4, C8 and C12) (pre-dose)	Section 8.5.2

	Post-surgery, prior to starting treatment	C1	C2	C3 to C12	For details, see CSP Section or Appendix
**Week**	**Within 10 weeks of surgery [a]**	**0**	**4**	**Q4W ± 3 days**	
**Day**		**1**	**29 ± 3**	**q28d ± 3 days**	
ctDNA [g]		X (pre-dose)	X (pre-dose)	X (pre-dose)	Section 8.6.3 and Section 8.7.1
Serum samples for circulating soluble factors [g]		X (pre-dose)		X ([redacted]) (pre-dose)	Section 8.6.3
Plasma samples for circulating soluble factors [g]		X (pre-dose)		X[redacted] (pre-dose)	Section 8.6.3
Whole blood for PBMCs [g]		X (pre-dose)		X[redacted] (pre-dose)	Section 8.6.3
Optional tumour biopsy specimen		X [k]			Section 8.6.1 and Section 8.6.4
**Efficacy evaluation**					
RECIST 1.1 tumour assessments (adjuvant period and beyond)	X [l, m]	Follow-up scans are acquired Q12W ± 1 week (relative to the date of surgery) until week 48; Q24W±2 weeks (relative to the date of surgery) until week 192 (approximately 4 years); and then Q48W ± 2 weeks (relative to the date of surgery) thereafter until RECIST 1.1-defined radiological PD, consent withdrawal, death, or study completion as determined by Sponsor. This on-study schedule MUST be followed regardless of any delays in dosing and regardless of tumour margins. These follow-up scans will use the original neoadjuvant screening scan as a baseline scan for RECIST 1.1 assessments.			Section 8.1.1 and Appendix F

a Patients will start treatment as soon as clinically feasible and within 10 weeks from surgery (except for patients receiving PORT, which must be started within 8 weeks after surgery).

b Body weight is recorded at each visit along with vital signs.

c Any clinically significant abnormalities detected require triplicate ECG results.

d Samples for laboratory assessment (serum or plasma clinical chemistry [including LFT monitoring] and haematology) may be obtained more frequently based on the local clinical practice or the Investigator's discretion. Results for LFTs, electrolytes, full blood count, and creatinine must be available before commencing an infusion (within 3 days) and reviewed by the treating physician or Investigator prior to dosing.

e Free T3 or Total T3, or free T4 will only be measured if TSH is abnormal or if there is clinical suspicion of an AE related to the endocrine system.

[f] For women of childbearing potential only. A urine or serum pregnancy test is acceptable. Women of childbearing potential are required to have a pregnancy test within 3 days prior to the first dose of study intervention and then Q4W prior to study interventions administration. Pregnancy test may occur on Day 1, but results must be available and reviewed by the treating physician or Investigator prior to commencing an infusion.

[g] Pre-dose collection: within 60 minutes prior to administration of any study intervention. Post dose collection (durvalumab and oleclumab): end-of-infusion collection to be performed within 5 hours after the end of the respective study interventions infusion.

[h] Must include all AEs that occur after surgery regardless of decision to start adjuvant treatment.

[i] The following sequence should be used for treatment administration in days when more than one agent is administered: Oleclumab > durvalumab

[j] Oleclumab will be administered at 3000 mg IV, Day 1 of each 28-day cycle, unless dosing needs to be held for toxicity reasons.

[k] It is strongly recommended/encouraged to collect additional tumour biopsies, if they become available at disease progression or unscheduled procedures during the study.

[l] A first post-surgical CT/MRI scan of the chest and abdomen (including the entire liver and both adrenals) must be acquired 5 weeks ± 2 weeks after surgery and prior to, but as close as possible to, the start of adjuvant treatment. This scan is used to determine eligibility to receive adjuvant treatment.

[m] A new primary malignancy confirmed by pathology is not considered an EFS or DFS event.

Note: All assessments on treatment days are to be performed prior to infusion, unless otherwise indicated.

Note: Patients should be able to start study interventions following surgery as soon as clinically feasible (unless PORT is given) and within 10 weeks from surgery.

Abbreviations: ADA: Anti-drug antibody; AE: Adverse event; aPTT: Activated partial thromboplastin time; C: Cycle; CSP: Clinical study protocol; CT: Computed tomography; ctDNA: Circulating tumour DFS: disease-free survival; DNA; DNA: Deoxyribonucleic acid; ECHO: Echocardiogram; ECG: Electrocardiogram; ECOG: Eastern Cooperative Oncology Group; EFS: Event-free survival; INR: International normalised ratio; IV: Intravenous; LFT: Liver function test; MUGA: multigated acquisition scans; MRI: Magnetic resonance imaging; PBMC: Peripheral blood mononuclear cells; PD: Progression of disease; PK: Pharmacokinetic(s); PORT: Post operative radiation therapy; Q12W: Every 12 weeks; Q24W: Every 24 weeks; q28d: Every 28 days; Q4W: Every 4 weeks; RECIST: Response Evaluation Criteria in Solid Tumours; SAE: Serious adverse event; T3: Triiodothyronine; T4: Thyroxine; TSH: Thyroid-stimulating hormone; WHO: World Health Organisation.

K 2 Disease Target

K 2.1 Programmed Cell Death Ligand-1

Programmed cell death protein-1, PD-L1, and PD-L2 are part of a complex system of receptors and ligands that control T cell activation. Programmed death-ligand 1 expression helps tumours evade detection and elimination by the immune system (Chen and Mellman, 2013, Juneja et al, 2017, Keir et al, 2008, Ohaegbulam et al, 2015). The binding of PD-L1 to PD-1 on activated T cells delivers an inhibitory signal preventing T cells from killing target tumour cells (Ohaegbulam et al, 2015, Okazaki et al, 2013, Pardoll, 2012, Wu et al, 2020, Yu et al 2009

Yu X, Harden K, Gonzalez LC, Frencesco M, Chiang E, Irving B, et al. The surface protein TIGIT suppresses T cell activation by promoting the generation of mature immunoregulatory dendritic cells. Nat Immunol. 2009;10(1):48-57.

Zou and Chen, 2008, Zou et al, 2016).

K 2.2 CD73

Adenosine is a regulatory autocrine and paracrine factor that accumulates in the tumour microenvironment, influencing immune activity, angiogenesis, and metastasis. Upon apoptotic or necrotic cell death, tumour cells release ATP into the extracellular space. Adenosine triphosphate has been shown to lead to a pro-inflammatory response. To prevent an immune overactivation, tissues express CD39 and CD73 to enzymatically convert ATP to adenosine, which induces a localised immunosuppressive response through multiple immune cell types. One mechanism by which tumours may have evolved to evade the immune system is via overexpression of CD73. Overexpression of CD73 has been associated with poor prognosis in multiple cancer types (Inoue et al, 2017, Turcotte et al, 2015, Vijayan et al, 2017). Notably, high CD73 expression in NSCLC was associated with poor OS and recurrence-free survival in a multivariate analysis (Inoue et al, 2017). It is hypothesised that blocking CD73 activity will reduce adenosine production, thus augmenting host and/or immunotherapy response to tumour.

K 3 Background for Study Interventions used in Arm 1: Durvalumab and Oleclumab

K 3.1 Durvalumab

Durvalumab is a human mAb of the IgG kappa subclass that blocks the interaction of PD-L1 (but not PD-L2) with PD 1 on T cells and CD80 (B7.1) on immune cells. It is being developed by AstraZeneca for use in the treatment of cancer. Blockade of PD-L1/PD 1 and PD-L1/CD80 interactions releases the inhibition of immune responses, including those that may result in tumour elimination.

Durvalumab (Imfinzi®) has been approved in the US and EU to treat unresectable Stage III NSCLC, and extensive-stage small cell lung cancer in combination with etoposide and either carboplatin or cisplatin in first-line treatment. Durvalumab in combination with tremelimumab and platinum-doublet chemotherapy is approved by the FDA for treatment of patients with metastatic NSCLC. Refer to the durvalumab IB and to the package insert (or label) for your specific country, as applicable.

K 3.2 Oleclumab

Oleclumab (MEDI9447) is a human IgG1 lambda mAb that selectively binds to CD73 and inhibits enzyme activity, as well as leads to a reduction in CD73 expression through internalisation, thus contributing to decreased adenosine production (Geoghegan et al, 2016, Hay et al, 2016). It contains a triple mutation in the heavy chain constant region for reduced effector function. Decreased adenosine production by binding of oleclumab to CD73 may lead to increased antitumour immunity. Refer to the current oleclumab IB for a complete summary of preclinical and clinical information including safety, efficacy, and PK.

K 4 Rationale for Combining Durvalumab with Chemotherapy and Oleclumab

The NeoCOAST study (NCT03794544) evaluated one cycle of durvalumab alone or in combination with oleclumab or monalizumab as neoadjuvant treatment in patients with resectable, early-stage NSCLC. Sixty-eight patients were enrolled. Major pathological response was demonstrated in 3 of 27 (11.1%), 4 of 21 (19.0%), and 6 of 20 (30.0%) patients, in the durvalumab monotherapy, durvalumab + oleclumab, and durvalumab + monalizumab arms, respectively. Pathological complete response was achieved in 1 (3.7%), 2 (9.5%), and 2 (10.0%) patients, in the durvalumab monotherapy, durvalumab + oleclumab, and durvalumab + monalizumab arms, respectively. Grade ≥ 3 TEAEs occurred in 5 (19.2%), 3 (14.3%), and 2 (10.0%) patients, in the durvalumab monotherapy, durvalumab + oleclumab, and durvalumab + monalizumab arms, respectively. Grade ≥ 3 TRAEs occurred in 1 (4.8%) patient in the durvalumab + oleclumab arm. One patient each experienced a serious TRAE in the durvalumab arm (immune-mediated arthritis) and durvalumab + oleclumab arm (diabetic ketoacidosis). One patient each experienced a TEAE leading to treatment discontinuation in the durvalumab + oleclumab arm (4.8%) and durvalumab + monalizumab arm (5.0%). The most common TEAE in all arms was fatigue and the most common TRAEs were fatigue (11.5%), asthenia (14.3%), fatigue and pruritus (each 10.0%), in the durvalumab monotherapy, durvalumab + oleclumab, and durvalumab + monalizumab, respectively (Cascone et al, 2022).

K 5 Justification for Dose for Study Interventions Used in Arm 1: Durvalumab and Oleclumab

K 5.1 Durvalumab

This study will utilise durvalumab (1500 mg Q3W) for 4 cycles prior to surgery, followed by durvalumab (1500 mg Q4W) for an additional 12 cycles after surgery.

Rationale for proposed 3 weeks fixed dosing pre-surgery:

The proposed dosing schedule is aligned with the standard fixed dosing of 1500 mg durvalumab for 4 cycles, which is supported by efficacy and safety as well as tolerability data across multiple studies in multiple tumour types. The 1500 mg Q3W for the first 4 cycles (prior to surgery) is selected to conform to the platinum doublet chemotherapy schedule in the study. Based on simulated durvalumab PK profiles, the overall distribution of durvalumab exposure at 1120 mg Q3W was expected to be consistent with the predicted levels at 1500 mg Q4W. Although predicted exposures of 1500 mg Q3W were predicted to be slightly higher compared to 1120 mg Q3W, the relative increase in dose density of durvalumab (ie, 1500 mg Q3W instead of Q4W or 1120 mg Q3W) is supported by the fact that durvalumab has a flat exposure-response (safety) relationship with clinically used dose levels, and PK modelling reveals no clinically meaningful differences in drug levels.

Rationale for proposed 4 weeks fixed dosing post-surgery:

Durvalumab dosing regimen of 1500 mg Q4W is the approved dosing regimen in different indications including unresectable Stage III NSCLC.

For additional details on the nonclinical and clinical data that informed durvalumab dose selection, see the durvalumab IB.

K 5.2 Oleclumab

The selected dose for oleclumab in this study is 3000 mg Q3W for 4 cycles prior to surgery, followed by 3000 mg Q4W for an additional 12 cycles post-surgery.

The safety and efficacy of the oleclumab dose of 3000 mg Q2W for 4 doses, followed by 3000 mg Q4W, in combination with durvalumab, with or without chemotherapy has been demonstrated in multiple ongoing clinical studies. This dose was selected based on available clinical safety, tolerability, efficacy and PK data from the Phase I Study D6070C00001. In this study, oleclumab doses of 5, 10, 20, and 40 mg/kg Q2W were examined both as a monotherapy and in combination with durvalumab 10 mg/kg Q2W. Oleclumab was well tolerated and there were no observed DLTs either as monotherapy or in combination with durvalumab. The oleclumab 40 mg/kg Q2W dose (equivalent to the oleclumab 3000 mg Q2W fixed dose for a 75 kg individual) was selected for evaluation with durvalumab 10 mg/kg Q2W in the dose expansion phase of that study.

The loading dose of 3000 mg Q2W for 4 doses was chosen with the intent to achieve exposures for the initial 70 days similar to those observed at 40 mg/kg Q2W dose in Study D6070C00001 where clinical activity has been observed and CD73 is expected to have been inhibited maximally. Subsequently, a Q4W schedule at 3000 mg is predicted to result in a trough concentration that is above the estimated CD73 saturating concentration, ie, is able to maintain optimal CD73 saturation throughout the dosing interval at steady state.

To conform to the platinum doublet chemotherapy schedule, 3000 mg Q3W, a dose that will generate exposure in between that of 3000 mg Q2W and 3000 mg Q4W, is selected as the dose for oleclumab in neo-adjuvant setting. After the surgery, the dose will be 3000 mg Q4W.

For additional details on the nonclinical and clinical data that informed oleclumab dose selection, see the oleclumab IB.

K 6 Study Intervention

K 6.1 Treatment Regimen

Patients will receive treatment as shown below.

	Neoadjuvant treatment/Pre-Surgery (4 cycles)	Adjuvant treatment/Post-Surgery (1 year)
**Arm 1**	Oleclumab + durvalumab + platinum doublet chemotherapy (Q3W)	Oleclumab + durvalb (Q4W)

No specific premedication is required for durvalumab or oleclumab.

Premedication for chemotherapy should be administered according to the local label and guidelines. Details of any premedication or concomitant medication given to manage or prevent AEs should be recorded on the eCRF.

A physician must be present at the site or immediately available to respond to emergencies during all administrations of study interventions. Fully functional resuscitation facilities should be available.

K 6.2 Storage of Investigational Products

Oleclumab and durvalumab vials are stored at 2°C to 8°C (36°F to 46°F) and must not be frozen. The investigator, or an approved representative (eg, pharmacist), will ensure that all study interventions are stored in a secured area and in accordance with applicable regulatory requirements. A temperature log will be used to record the temperature of the storage area. Temperature excursions outside the permissible range listed in the clinical supply packaging are to be reported to the monitor upon detection. A calibrated temperature-monitoring device

will be used to record the temperature conditions in the drug storage facility. Storage conditions stated in the IB may be superseded by the label storage.

Study interventions must be kept in original packaging until time of preparation to prevent prolonged light exposure.

K 6.3 Investigational Product Preparation and Administration

K 6.3.1 Preparation

Each vial selected for dose preparation should be inspected. If there are any defects noted with the Investigational Product, the investigator and site monitor should be notified immediately.

The use of elastomeric pumps and pneumatic tube transport with oleclumab and durvalumab and have not been studied, and AstraZeneca must be contacted prior to potential usage.

Doses of oleclumab and durvalumab for administration must be prepared by the Investigator's or site's designated investigational product manager using aseptic technique in compliance with local regulations and site requirements.

The total time from needle puncture of the oleclumab and durvalumab vial to the start of administration must not exceed 24 hours.Of this time, not more than 4 hours may be at room temperature, with the remaining time at 2°C to 8°C (36°F to 46°F), otherwise a new dose must be prepared from new vials.

Oleclumab and durvalumab vials do not contain preservatives; any unused portion of the vial must be discarded immediately after use.

Oleclumab doses will be prepared using an IV bag containing 0.9% sodium chloride for injection.

Durvalumab doses will be prepared using an IV bag containing 0.9% sodium chloride for injection or 5% dextrose for injection.

Refer to the Pharmacy Manual for detailed information about preparation of oleclumab and durvalumab.

K 6.3.2 Administration

Oleclumab and durvalumab infusions are to be administered through an IV administration set with a 0.2- or 0.22-µm filter; acceptable configurations include an IV set containing an in-line filter or the attachment of a separate filter to the distal end of the IV tubing.

The oleclumab and durvalumab infusion time is 1 hour ± 10 minutes; however, if there are interruptions, the total allowed time must not exceed 8 hours with the infusion bag kept at

room temperature, otherwise a new dose must be prepared from new vials.

Do not co-administer other drugs through the same infusion line.

The IV line will be flushed with a volume equal to the IV line volume, according to local practices, to ensure the full dose is administered. Infusion time does not include the final flush time.

K 7 Adverse Events of Special Interest Associated with Oleclumab

Cardiac Chest Pain, Transient Ischemic Attack, and Thromboembolism

Adverse events of cardiac chest pain, transient ischaemic attack, and thromboembolism are of special interest due to oleclumab potential risks of arterial ischemic disorder, and thrombosis. Because of this potential risk, potential patients with a history of myocardial infarction or any other arterial thrombotic event within 6 months before study enrolment are not eligible (see Section 5.2). These events require urgent medical management, which should be performed according to consensus guidelines developed by the American Heart Association or appropriate local SoC.

Oedema

Oedema (eg, pulmonary or peripheral) is regarded as an AESI due to oleclumab potential risks of increased microvascular permeability. For patients who develop ≥ Grade 2 pulmonary oedema or ≥ Grade 3 peripheral oedema after treatment with durvalumab plus oleclumab, doses of the agent causing the AE should be omitted (TMGs, Section 8.3.15) and may be discontinued at the discretion of the Investigator.

Appendix L Arm 2: Monalizumab + Durvalumab + Platinum Doublet Therapy (Neoadjuvant Treatment) Followed by Monalizumab + Durvalumab (Adjuvant Treatment)

This appendix provides information for patients randomised to Arm 2.

L 1 Schedule of Activities

Table L20 Schedule of Activities for Neoadjuvant Treatment for Patients Randomised to Arm 2

	C1	C2	C3 and C4	Pre-surgical assessments	Surgery	For details, see CSP Section or Appendix
Week	0	Q3W + 3 days [a]		Within 30 days before surgery	Within 40 days of the last dose of study interventions	
Day	1 [b]	q21d + 3 days [a]				
**Study procedures**						
Physical examination (full)				X		Section 8.2.2
Targeted physical examination (based on symptoms)	X	X	X			Section 8.2.2
Vital signs [c]	X	X	X	X		Section 8.2.3
ECHO/MUGA		As clinically indicated				Section 8.2.5
ECG [d]	As clinically indicated			X		Section 8.2.4
Concomitant medications	X	X	X	X		Section 6.5
Contrast-enhanced CT/MRI scan of the chest and abdomen (including adrenal glands) and PET scan		X (CT only at Cycle 2)		X [e]		Section 6.1.3 and Section 8.1
Pulmonary function testing and cardiac risk assessment				X [f]		Section 8.2.7
Eligibility criteria				X (pre-surgery conditions)		Section 5 and Section 6.1.3

	C1	C2	C3 and C4	Pre-surgical assessments	Surgery	For details, see CSP Section or Appendix
**Week**	**0**	**Q3W + 3 days [a]**		**Within 30 days before surgery**	**Within 40 days of the last dose of study interventions**	
**Day**	**1 [b]**	**q21d + 3 days [a]**				
**Laboratory assessments**						
Clinical chemistry [g]	X [h]	X	X	X		Section 8.2.1
Cortisol [g]				X		Section 8.2.1
Haematology [g]	X [h]	X	X	X		Section 8.2.1
aPTT and INR	As clinically indicated			X		Section 8.2.1
TSH (reflex free T3 or Total T3, or free T4) [i]	X [j]	X	X			Section 8.2.1
Urinalysis	As clinically indicated					Section 8.2.1
Hepatitis B and C	As clinically indicated					Section 8.2.1
Pregnancy test [k]	X	X	X	X		Section 8.2.1
**Pharmacokinetics**						
Durvalumab PK sample [l]	X (pre-dose) X (post-dose)	X (pre-dose)	X (C4) (pre-dose) X (C4) (post-dose)			Section 8.5.1
Monalizumab PK sample [l]	X (pre-dose) X (post-dose)	X (pre-dose)	X (C4) (pre-dose) X (C4) (post-dose)			Section 8.5.1
**Monitoring**						
WHO/ECOG performance status	X	X	X	X		Section 8.2.6
AE/SAE assessment	X	X	X	X		Section 8.3

	C1	C2	C3 and C4	Pre-surgical assessments	Surgery	For details, see CSP Section or Appendix
Week	0	Q3W + 3 days [a]		Within 30 days before surgery	Within 40 days of the last dose of study interventions	
Day	1 [b]	q21d + 3 days [a]				
**Pre-treatment medication**						
Folic acid/vitamin B12 [m]	Continue in line with local practice					Section 6.1.1
**Study interventions administration [n]**						
Monalizumab [o]	X	X	X			Section 6.1.1
Durvalumab	X	X	X			Section 6.1.1
Chemotherapy [p]	X	X	X			Section 6.1.1
**Other assessments and assays**						
Durvalumab immunogenicity assessment (ADA sampling to identify ADA responses in patient circulation) [l]	X (pre-dose)	X (pre-dose)	X (C4) (pre-dose)			Section 8.5.2
Monalizumab immunogenicity assessment (ADA sampling to identify ADA responses) [l]	X (pre-dose)	X (pre-dose)	X (C4) (pre-dose)			Section 8.5.2
ctDNA [l]	X (pre-dose)	X (pre-dose)	X (pre-dose)	X		Section 8.6.3 and Section 8.7.1
Mandatory tumour and lymph nodes specimen					X	Section 8.6.1 and Section 8.6.2
Whole blood for [redacted] Expression [l]	X (pre-dose)	X (pre-dose)		X		Section 8.6.3
Serum samples for circulating soluble factors [l]	X (pre-dose)	X (pre-dose)		X		Section 8.6.3
Plasma samples for circulating soluble factors [l]	X (pre-dose)	X (pre-dose)		X		Section 8.6.3

	C1	C2	C3 and C4	Pre-surgical assessments	Surgery	For details, see CSP Section or Appendix
Week	0	Q3W + 3 days [a]		Within 30 days before surgery	Within 40 days of the last dose of study interventions	
Day	1 [b]	q21d + 3 days [a]				
Whole blood for [redacted] analyses [l]	X (pre-dose)					Section 8.6.3
Whole blood for PBMC [l]	X (pre-dose)	X (pre-dose)		X		Section 8.6.3
[redacted] Initiative optional, exploratory [redacted] blood sample [q]	X					Section 8.7.2 and Appendix D
**Efficacy evaluation**						
mPR and pCR determined by central pathology review					X	Section 8.1
RECIST 1.1 tumour assessments on CT and/or MRI scans	A RECIST 1.1 tumour assessment is performed after Cycle 2 and upon completion of the neoadjuvant period, prior to surgery.					Section 8.1.1 and Appendix F

a Subsequent time between 2 consecutive doses cannot be less than 21 days, based on the half-life of durvalumab and monalizumab.
b Every effort should be made to minimise the time between randomisation and starting treatment (ie, within 1 day and not more than 3 days of randomisation).
c Body weight is recorded at each visit along with vital signs.
d Any clinically significant abnormalities detected require triplicate ECG results.
e A CT and PET scan should be performed pre-surgery.
f Pulmonary function testing may be repeated at Investigator's or surgeon's discretion prior to surgery to inform fitness for surgery.
g Serum or plasma clinical chemistry (including LFT monitoring) and haematology may be performed more frequently based on the local clinical practice or the Investigator's discretion, or if clinically indicated. Results for LFTs, electrolytes, full blood count, and creatinine must be available before commencing an infusion (within 3 days) and reviewed by the treating physician or Investigator prior to dosing. Cortisol testing should be performed at the pre surgical visit.
h If screening clinical chemistry and haematology assessments are performed within 3 days prior to Day 1 (first infusion day), they do not need to be repeated on Day 1.
i Free T3 or Total T3, or free T4 will only be measured if TSH is abnormal or if there is clinical suspicion of an AE related to the endocrine system.
j If TSH is measured within 14 days prior to Day 1 (first infusion day), it does not need to be repeated on Day 1.

[k] For women of childbearing potential only. A urine or serum pregnancy test is acceptable. Women of childbearing potential are required to have a pregnancy test within 3 days prior to the first dose of study interventions and then Q3W prior to study interventions. Pregnancy test may occur on Day 1, but results must be available and reviewed by the treating physician or Investigator prior to commencing an infusion.

[l] Pre-dose collection: within 60 minutes prior to administration of any study intervention. Post-dose collection (durvalumab, monalizumab): end-of-infusion collection to be taken within 5 hours after the end of the respective study interventions infusion.

[m] To be administered in line with local practice for patients with non-squamous tumours who will receive pemetrexed.

[n] The following sequence should be used for treatment administration in days when all or more than one agent is administered: Monalizumab > durvalumab > platinum doublet chemotherapy.

[o] Monalizumab will be administered at 1500 mg IV, Day 1 of each 21-day cycle. Refer to Section 8.3.15 for TMGs.

[p] Platinum doublet chemotherapy (Investigator's choice of one of the regimens listed in Section 6.1.1).

[q] The sample for [redacted] research will be obtained on Day 1 pre-dose (at or after randomisation). If, for any reason, the sample is not drawn on Day 1, it may be taken at any visit until the last study visit. Only 1 sample should be collected per patient for [redacted] analysis during the study.

Note: Each cycle during the neoadjuvant treatment is 21 days (3 weeks) in duration, unless dosing needs to be held for toxicity reasons.

Note: All assessments on treatment days are to be performed prior to infusion, unless otherwise indicated.

Note: Surgery is expected within 40 days from the last dose of study interventions. Cases where surgery cannot be completed within 40 days from the last dose of study interventions should be discussed with the Study Physician.

Abbreviations: ADA: Anti-drug antibody; AE: Adverse event; aPTT: Activated partial thromboplastin time; C: Cycle; CSP: Clinical study protocol; CT: Computed tomography; ctDNA: Circulating tumour DNA; DNA: Deoxyribonucleic acid; ECG Electrocardiogram; ECHO: Echocardiogram; ECOG: Eastern Cooperative Oncology Group; INR: International normalised ratio; IV: Intravenous; LFT: Liver function test; mPR: Major pathological response; MRI: Magnetic resonance imaging; MUGA: multigated acquisition scans; PBMC: Peripheral blood mononuclear cells; pCR: Pathological complete response; PD-L1: Programmed cell death ligand-1; PET: Positron emission tomography; PK: Pharmacokinetic(s); q21d: Every 21 days; Q3W: Every 3 weeks; RECIST: Response Evaluation Criteria in Solid Tumours; SAE: Serious adverse event; T3: Triiodothyronine; T4: Thyroxine; TMGs: Toxicity management guidelines; TSH: Thyroid-stimulating hormone; WHO: World Health Organisation

Table L21 Schedule of Activities for Adjuvant Treatment for Patients Randomised to Arm 2

	Post-surgery, prior to starting treatment	C1	C2	C3 to C12	For details, see CSP Section or Appendix
Week	Within 10 weeks of surgery [a]	0	4	Q4W ± 3 days	
Day		1	29 ± 3	q28d ± 3 days	
**Study procedures**					
Physical examination (full)		X			Section 8.2.2
Targeted physical examination (based on symptoms)			X	X	Section 8.2.2
Vital signs [b]		X	X	X	Section 8.2.3
ECHO/MUGA		As clinically indicated			Section 8.2.5
ECG [c]		X	As clinically indicated		Section 8.2.4
Concomitant medications	X	X	X	X	Section 6.5
Post-surgery conditions	X				Section 6.1.3
**Laboratory assessments**					
Clinical chemistry [d]		X [d]	X [d]	X [d]	Section 8.2.1
Haematology [d]		X [d]	X [d]	X [d]	Section 8.2.1
aPTT and INR	As clinically indicated				Section 8.2.1
TSH (reflex free T3 or Total T3, or free T4) [e]		X	X	X	Section 8.2.1
Urinalysis		X	As clinically indicated		Section 8.2.1
Hepatitis B and C		As clinically indicated			Section 8.2.1
Pregnancy test [f]		X	X	X	Section 8.2.1

	Post-surgery, prior to starting treatment	C1	C2	C3 to C12	For details, see CSP Section or Appendix
**Week**	**Within 10 weeks of surgery** [a]	**0**	**4**	**Q4W ± 3 days**	
**Day**		**1**	**29 ± 3**	**q28d ± 3 days**	
**Pharmacokinetics**					
Durvalumab PK sample [g]		X (pre-dose) X (post-dose)	X (pre-dose)	X (C4) (pre-dose)	Section 8.5.1
Monalizumab PK sample [g]		X (pre-dose) X (post-dose)	X (pre-dose)	X (C4, C8 and C12) (pre-dose)	Section 8.5.1
**Monitoring**					
WHO/ECOG performance status		X	X	X	Section 8.2.6
AE/SAE assessment	X [h]	X	X	X	Section 8.3
**Study interventions administration** [i]					
Monalizumab [j]		X	X	X	Section 6.1.1
Durvalumab		X	X	X	Section 6.1.1
**Other assessments and assays**					
Durvalumab immunogenicity assessment (ADA sampling to identify ADA responses in patient circulation) [g]		X (pre-dose)	X (pre-dose)	X (C4 and C12) (pre-dose)	Section 8.5.2
Monalizumab immunogenicity assessment (ADA sampling to identify ADA responses) [g]		X (pre-dose)	X (pre-dose)	X (C4, C8 and C12) (pre-dose)	Section 8.5.2

	Post-surgery, prior to starting treatment	C1	C2	C3 to C12	For details, see CSP Section or Appendix
Week	Within 10 weeks of surgery [a]	0	4	Q4W ± 3 days	
Day		1	29 ± 3	q28d ± 3 days	
ctDNA [g]		X (pre-dose)	X (pre-dose)	X (pre-dose)	Section 8.6.3 and Section 8.7.1
Serum samples for circulating soluble factors [g]		X (pre-dose)		X[redacted] (pre-dose)	Section 8.6.3
Plasma samples for circulating soluble factors [g]		X (pre-dose)		X[redacted] (pre-dose)	Section 8.6.3
Whole blood for PBMCs [g]		X (pre-dose)		X[redacted] (pre-dose)	Section 8.6.3
Optional tumour biopsy specimen		X[k]			Section 8.6.1 and Section 8.6.4
**Efficacy evaluation**					
RECIST 1.1 tumour assessments (adjuvant period and beyond)	X[l, m]	Follow-up scans are acquired Q12W ± 1 week (relative to the date of surgery) until week 48; Q24W±2 weeks (relative to the date of surgery) until week 192 (approximately 4 years); and then Q48W ± 2 weeks (relative to the date of surgery) thereafter until RECIST 1.1-defined radiological PD, consent withdrawal, death, or study completion as determined by Sponsor. This on-study schedule MUST be followed regardless of any delays in dosing and regardless of tumour margins. These follow-up scans will use the original neoadjuvant screening scan as a baseline scan for RECIST 1.1 assessments.			Section 8.1.1 and Appendix F

a Patients will start treatment as soon as clinically feasible and within 10 weeks from surgery (except for patients receiving PORT, which must be started within 8 weeks after surgery).

b Body weight is recorded at each visit along with vital signs.

c Any clinically significant abnormalities detected require triplicate ECG results.

d Samples for laboratory assessment (serum or plasma clinical chemistry [including LFT monitoring] and haematology) may be obtained more frequently based on the local clinical practice or the Investigator's discretion. Results for LFTs, electrolytes, full blood count, and creatinine must be available before commencing an infusion (within 3 days) and reviewed by the treating physician or Investigator prior to dosing.

e Free T3 or Total T3, or free T4 will only be measured if TSH is abnormal or if there is clinical suspicion of an AE related to the endocrine system.

[f] For women of childbearing potential only. A urine or serum pregnancy test is acceptable. Women of childbearing potential are required to have a pregnancy test within 3 days prior to the first dose of study intervention and then Q4W prior to study interventions administration. Pregnancy test may occur on Day 1, but results must be available and reviewed by the treating physician or Investigator prior to commencing an infusion.

[g] Pre-dose collection: within 60 minutes prior to administration of any study intervention. Post dose collection (durvalumab and monalizumab): end-of-infusion collection to be performed within 5 hours after the end of the respective study interventions infusion.

[h] Must include all AEs that occur after surgery regardless of decision to start adjuvant treatment.

[i] The following sequence should be used for treatment administration in days when more than one agent is administered: Monalizumab > durvalumab.

[j] Monalizumab will be administered at 1500 mg IV, Day 1 of each 28-day cycle, unless dosing needs to be held for toxicity reasons.

[k] It is strongly recommended/encouraged to collect additional tumour biopsies, if they become available at disease progression or unscheduled procedures during the study.

[l] A first post-surgical CT/MRI scan of the chest and abdomen (including the entire liver and both adrenals) must be acquired 5 weeks ± 2 weeks after surgery and prior to, but as close as possible to, the start of adjuvant treatment. This scan is used to determine eligibility to receive adjuvant treatment.

[m] A new primary malignancy confirmed by pathology is not considered an EFS or DFS event.

Note: All assessments on treatment days are to be performed prior to infusion, unless otherwise indicated.

Note: Patients should be able to start study interventions following surgery as soon as clinically feasible (unless PORT is given) and within 10 weeks from surgery.

Abbreviations: ADA: Anti-drug antibody; AE: Adverse event; aPTT: Activated partial thromboplastin time; C: Cycle; CSP: Clinical study protocol; CT: Computed tomography; ctDNA: Circulating tumour DFS: disease-free survival; DNA; DNA: Deoxyribonucleic acid; ECHO: Echocardiogram; ECG: Electrocardiogram; ECOG: Eastern Cooperative Oncology Group; EFS: Event-free survival; INR: International normalised ratio; IV: Intravenous; LFT: Liver function test; MUGA: multigated acquisition scans; MRI: Magnetic resonance imaging; PBMC: Peripheral blood mononuclear cells; PD: Progression of disease; PK: Pharmacokinetic(s); PORT: Post operative radiation therapy; Q12W: Every 12 weeks; Q24W: Every 24 weeks; q28d: Every 28 days; Q4W: Every 4 weeks; RECIST: Response Evaluation Criteria in Solid Tumours; SAE: Serious adverse event; T3: Triiodothyronine; T4: Thyroxine; TSH: Thyroid-stimulating hormone; WHO: World Health Organisation.

L 2 Disease Target

L 2.1 Programmed Cell Death Ligand-1

Programmed cell death protein-1, PD-L1, and PD-L2 are part of a complex system of receptors and ligands that control T cell activation. Programmed death-ligand 1 expression helps tumours evade detection and elimination by the immune system (Chen and Mellman, 2013, Juneja et al, 2017, Keir et al, 2008, Ohaegbulam et al, 2015). The binding of PD-L1 to PD-1 on activated T cells delivers an inhibitory signal preventing T cells from killing target tumour cells (Ohaegbulam et al, 2015, Okazaki et al, 2013, Pardoll, 2012, Wu et al, 2020, Yu et al 2009

Yu X, Harden K, Gonzalez LC, Frencesco M, Chiang E, Irving B, et al. The surface protein TIGIT suppresses T cell activation by promoting the generation of mature immunoregulatory dendritic cells. Nat Immunol. 2009;10(1):48-57.

Zou and Chen, 2008, Zou et al, 2016).

L 2.2 NKG2a

Major histocompatibility complex E is a non-classical major histocompatibility complex class I molecule, over-expressed by malignant cells in a variety of tumour types. Major histocompatibility complex E can present antigens to the CD94/NKG2a receptors on the surface of some types of lymphocytes. Activation of CD94/NKG2a receptors induces inhibitory signals that suppress cytokine secretion and direct cytotoxicity of cytotoxic T lymphocytes or NK cells against stressed, infected, or "transformed" cells. Such activity has been described as a possible mechanism in immune escape of cancer cells (Awad et al, 2023

Awad MM, Forde PM, Girard N, Spicer JD, Wang C, S. Lu S, et al. Neoadjuvant nivolumab (N) + ipilimumab (I) vs chemotherapy (C) in the phase III CheckMate 816 trial. Annals of Oncology. 2023;34(2):731.
Available at: https://doi.org/10.1016/j.annonc.2023.09.739.

Bai et al, 2020, Bradbury et al, 2017, Braud et al, 1998). Conversely, the blockade of CD94/NKG2a by an antagonist mAb restores the response of NK cells, enhancing notably their cytotoxicity against tumour cells expressing HLA-E.

L 3 Background for Study Interventions Used in Arm 2: Durvalumab and Monalizumab

L 3.1 Durvalumab

Durvalumab is a human mAb of the IgG kappa subclass that blocks the interaction of PD-L1 (but not PD-L2) with PD 1 on T cells and CD80 (B7.1) on immune cells. It is being developed by AstraZeneca for use in the treatment of cancer. Blockade of PD-L1/PD 1 and PD-L1/CD80

interactions releases the inhibition of immune responses, including those that may result in tumour elimination.

Durvalumab (Imfinzi®) has been approved in the US and EU to treat unresectable Stage III NSCLC, and extensive-stage small cell lung cancer in combination with etoposide and either carboplatin or cisplatin in first-line treatment. Durvalumab in combination with tremelimumab and platinum-doublet chemotherapy is approved by the FDA for treatment of patients with metastatic NSCLC. Refer to the durvalumab IB and to the package insert (or label) for your specific country, as applicable.

L 3.2 Monalizumab

Monalizumab (IPH2201) is a humanised mAb of the IgG-4 subtype produced in Chinese hamster ovary cells. It has a non-depleting and purely blocking activity directed with high affinity and specificity against the NKG2a subunit of the heterodimeric inhibitory CD94/NKG2a receptor expressed by subsets of NK cells, activated αβ CD8+ T cells and γδ T cells. By suppressing the inhibitory signal transduced by CD94/NKG2a, monalizumab enhances the antitumour functions, including cytolytic activity of these immune effector cells. Refer to the current monalizumab IB for a complete summary of preclinical and clinical information including safety, efficacy, and PK.

L 4 Rationale for Combining Durvalumab with Chemotherapy and Monalizumab

The NeoCOAST study (NCT03794544) evaluated one cycle of durvalumab alone or in combination with oleclumab or monalizumab as neoadjuvant treatment in patients with resectable, early-stage NSCLC. Sixty-eight patients were enrolled. Major pathological response was demonstrated in 3 of 27 (11.1%), 4 of 21 (19.0%), and 6 of 20 (30.0%) patients, in the durvalumab monotherapy, durvalumab + oleclumab, and durvalumab + monalizumab arms, respectively. Pathological complete response was achieved in 1 (3.7%), 2 (9.5%), and 2 (10.0%) patients, in the durvalumab monotherapy, durvalumab + oleclumab, and durvalumab + monalizumab arms, respectively. Grade ≥ 3 TEAEs occurred in 5 (19.2%), 3 (14.3%), and 2 (10.0%) patients, in the durvalumab monotherapy, durvalumab + oleclumab, and durvalumab + monalizumab arms, respectively. Grade ≥ 3 TRAEs occurred in 1 (4.8%) patient in the durvalumab + oleclumab arm. One patient each experienced a serious TRAE in the durvalumab arm (immune-mediated arthritis) and durvalumab + oleclumab arm (diabetic ketoacidosis). One patient each experienced a TEAE leading to treatment discontinuation in the durvalumab + oleclumab arm (4.8%) and durvalumab + monalizumab arm (5.0%). The most common TEAE in all arms was fatigue and the most common TRAEs were fatigue (11.5%), asthenia (14.3%), fatigue and pruritus (each 10.0%), in the durvalumab monotherapy, durvalumab + oleclumab, and durvalumab + monalizumab, respectively (Cascone et al, 2022).

L 5 Justification for Dose for Study Interventions Used in Arm 2

L 5.1 Durvalumab

This study will utilise durvalumab (1500 mg Q3W) for 4 cycles prior to surgery, followed by durvalumab (1500 mg Q4W) for an additional 12 cycles after surgery.

Rationale for proposed 3 weeks fixed dosing pre-surgery:

The proposed dosing schedule is aligned with the standard fixed dosing of 1500 mg durvalumab for 4 cycles, which is supported by efficacy and safety as well as tolerability data across multiple studies in multiple tumour types. The 1500 mg Q3W for the first 4 cycles (prior to surgery) is selected to conform to the platinum doublet chemotherapy schedule in the study. Based on simulated durvalumab PK profiles, the overall distribution of durvalumab exposure at 1120 mg Q3W was expected to be consistent with the predicted levels at 1500 mg Q4W. Although predicted exposures of 1500 mg Q3W were predicted to be slightly higher compared to 1120 mg Q3W, the relative increase in dose density of durvalumab (ie, 1500 mg Q3W instead of Q4W or 1120 mg Q3W) is supported by the fact that durvalumab has a flat exposure-response (safety) relationship with clinically used dose levels, and PK modelling reveals no clinically meaningful differences in drug levels.

Rationale for proposed 4 weeks fixed dosing post-surgery:

Durvalumab dosing regimen of 1500 mg Q4W is the approved dosing regimen in different indications including unresectable Stage III NSCLC.

For additional details on the nonclinical and clinical data that informed durvalumab dose selection, see the durvalumab IB.

L 5.2 Monalizumab

In the proposed study, monalizumab will be dosed, in the neoadjuvant setting, at 1500 mg Q3W in combination with durvalumab 1500 mg Q3W and platinum doublet chemotherapy for 4 cycles and, in the adjuvant setting, at 1500 mg Q4W in combination with durvalumab 1500 mg Q4W up to 12 cycles.

Monalizumab in combination with durvalumab is supported by the available clinical safety, tolerability, efficacy, and PK data from the ongoing Phase I Study D419NC00001 in patients with metastatic or recurrent solid tumours. In that study, monalizumab doses of 22.5, 75, 225, and 750 mg Q2W and 750 mg Q4W were examined in combination with durvalumab 1500 mg Q4W. Monalizumab was well-tolerated in all dose escalation cohorts and no DLTs were observed. The monalizumab 750 mg Q2W dose was identified for evaluation with durvalumab 1500 mg Q4W in the dose expansion phase of the study.

The safety of the combination of monalizumab (750 mg Q2W) and durvalumab (1500 mg

Q4W) is further supported by data from the COAST study in patients with unresectable stage III NSCLC. The combination treatment was well-tolerated, and no safety concerns were highlighted. The treatment-related Grade ≥ 3 rate was 27.9% with the combination compared to 32.2% with durvalumab alone. Study intervention-related SAEs occurred in 6.6% of patients with the combination vs 9.1% of patients with durvalumab, with discontinuations occurring in 13.1% vs 16.7% of patients.

The safety of the higher monalizumab dose (ie, [redacted] mg) is based on study IPH2201-203 which evaluated durvalumab [redacted] mg [redacted] in combination with monalizumab and cetuximab in patients with 1L recurrent or metastatic SCCHN. One study Arm enrolled [redacted] patients who received monalizumab at a dose of [redacted] mg [redacted] In this cohort, a tolerable safety profile was observed. Grade 3-4 treatment-emergent AEs occurred in [redacted]%. Most frequently Grade 3-4 AEs were increased amylase and lipase [redacted]%), dermatitis acneiformis [redacted]%), diarrhoea, vomiting and dyspnoea [redacted]% each). No fatal events occurred in this treatment arm.

Pharmacokinetic simulations (n=[redacted] for each cohort) were conducted using the monalizumab population PK model to predict and compare the PK exposure following [redacted] mg [redacted] [redacted] mg [redacted] [redacted] mg [redacted] and [redacted] mg [redacted] These simulations suggested that [redacted] mg [redacted] would yield a similar AUC (Day 0-168) to [redacted] mg [redacted] while the AUC of [redacted] mg [redacted] is predicted to be higher than that of the other regimens as expected. Predicted median Cmax at steady state (Cmax,ss, at month 6) following [redacted] mg [redacted] is only [redacted]% higher than that of [redacted] mg [redacted] and [redacted]% higher than that of [redacted] mg [redacted] The limited increase in monalizumab exposure at [redacted] mg [redacted] (proposed dosing regimen in neoadjuvant setting) is not expected to affect the safety profile of monalizumab compared to the [redacted] mg [redacted] dosing regimen that was showed to be tolerable in study IPH2201-203.

The PK simulations suggested that all regimen would maintain a trough concentration (Ctrough) above the target concentration (2 µg/mL) in most of the patients, where nonlinear clearance is expected to be fully saturated.

Taken together, available safety data and PK simulations support the use of monalizumab at a dose of 1500 mg Q3W (neoadjuvant setting) and 1500 mg Q4W (adjuvant setting).

For additional details on the nonclinical and clinical data that informed monalizumab dose selection, see the monalizumab IB.

L 6 Study Intervention

L 6.1 Treatment Regimen

Patients will receive treatment as shown below.

	Neoadjuvant treatment/Pre-Surgery (4 cycles)	Adjuvant treatment/Post-Surgery (1 year)
Arm 2	Monalizumab + durvalumab + platinum doublet chemotherapy (Q3W)	Monalizumab + durvalumab (Q4W)

No specific premedication is required for durvalumab or monalizumab.

Premedication for chemotherapy should be administered according to the local label and guidelines. Details of any premedication or concomitant medication given to manage or prevent AEs should be recorded on the eCRF.

A physician must be present at the site or immediately available to respond to emergencies during all administrations of study interventions. Fully functional resuscitation facilities should be available.

L 6.2 Storage of Investigational Products

Monalizumab and durvalumab vials are stored at 2°C to 8°C (36°F to 46°F) and must not be frozen. The investigator, or an approved representative (eg, pharmacist), will ensure that all study interventions are stored in a secured area and in accordance with applicable regulatory requirements. A temperature log will be used to record the temperature of the storage area. Temperature excursions outside the permissible range listed in the clinical supply packaging are to be reported to the monitor upon detection. A calibrated temperature-monitoring device will be used to record the temperature conditions in the drug storage facility. Storage conditions stated in the IB may be superseded by the label storage.

Study interventions must be kept in original packaging until time of preparation to prevent prolonged light exposure.

L 6.3 Investigational Product Preparation and Administration

Monalizumab will be supplied by AstraZeneca as either a lyophilised product or a liquid product. These dosage forms are clinically interchangeable; the end user administering the product and the patient will not be aware of any differences in the dosage form presentation.

Refer to the Pharmacy Manual for more detailed information about the lyophilised or liquid monalizumab product.

Preparation

Each vial selected for dose preparation should be inspected. If there are any defects noted with the Investigational Product, the investigator and site monitor should be notified immediately.

The use of elastomeric pumps and pneumatic tube transport with monalizumab and

durvalumab have not been studied, and AstraZeneca must be contacted prior to potential usage.

Doses of monalizumab and durvalumab for administration must be prepared by the Investigator's or site's designated investigational product manager using aseptic technique in compliance with local regulations and site requirements.

The total time from needle puncture of the monalizumab and durvalumab vial to the start of administration must not exceed 24 hours. Of this time, not more than 4 hours may be at room temperature, with the remaining time at 2°C to 8°C (36°F to 46°F), otherwise a new dose must be prepared from new vials.

Monalizumab and durvalumab vials do not contain preservatives; any unused portion of the vial must be discarded immediately after use.

Monalizumab and durvalumab doses will be prepared using an IV bag containing 0.9% sodium chloride for injection or 5% dextrose for injection.

Refer to the Pharmacy Manual for detailed information about preparation of monalizumab (lyophilised and liquid product) and durvalumab.

L 6.3.1 Administration

Monalizumab and durvalumab infusions are to be administered through an IV administration set with a 0.2- or 0.22-μm filter; acceptable configurations include an IV set containing an in-line filter or the attachment of a separate filter to the distal end of the IV tubing.

The monalizumab and durvalumab infusion time is 1 hour ± 10 minutes; however, if there are interruptions, the total allowed time must not exceed 8 hours with the infusion bag kept at room temperature, otherwise a new dose must be prepared from new vials.

Do not co-administer other drugs through the same infusion line.

The IV line will be flushed with a volume equal to the IV line volume, according to local practices, to ensure the full dose is administered. Infusion time does not include the final flush time.

L 7 Adverse Events of Special Interest Associated with Monalizumab

There are no AESIs identified for monalizumab.

Appendix N Arm 4: Dato-DXd + Durvalumab + Single Agent Platinum Chemotherapy (Neoadjuvant Treatment) Followed by Durvalumab Alone (Adjuvant Treatment)

This appendix provides information for patients randomised to Arm 4.

N 1 Schedule of Activities

Table N26 Schedule of Activities for Neoadjuvant Treatment for Patients Randomised to Arm 4

	C1	C2	C3 and C4	Pre-surgical assessments	Surgery	For details, see CSP Section or Appendix
Week	0	Q3W + 3 days [a]		Within 30 days before surgery	Within 40 days of the last dose of study interventions	
Day	1 [b]	q21d + 3 days [a]				
**Study procedures**						
Physical examination (full)				X		Section 8.2.2
Targeted physical examination (based on symptoms)	X	X	X			Section 8.2.2
Oral care plan [c]	Daily oral care plan before Dato-DXd administration and maintained throughout Dato-DXd treatment					Appendix N 6.1
Ophthalmologic assessment [d]	As clinically indicated					Section 8.2.8
Vital signs [e]	X	X	X	X		Section 8.2.3
ECHO/MUGA	As clinically indicated					Section 8.2.5
ECG [f]	As clinically indicated			X		Section 8.2.4
Concomitant medications	X	X	X	X		Section 6.5
Contrast-enhanced CT/MRI scan of the chest and abdomen (including adrenal glands) and PET scan		X (CT only at Cycle 2)		X [g]		Section 6.1.3 and Section 8.1
Pulmonary function testing and cardiac risk assessment				X [h]		Section 8.2.7
Eligibility criteria				X (pre-surgery conditions)		Section 5 and Section 6.1.3

	C1	C2	C3 and C4	Pre-surgical assessments	Surgery	For details, see CSP Section or Appendix
**Week**	**0**	**Q3W + 3 days [a]**		**Within 30 days before surgery**	**Within 40 days of the last dose of study interventions**	
**Day**	**1 [b]**	**q21d + 3 days [a]**				
**Laboratory assessments**						
Clinical chemistry [i]	X [j]	X	X	X		Section 8.2.1
Cortisol [i]				X		Section 8.2.1
Haematology [i]	X [j]	X	X	X		Section 8.2.1
aPTT and INR	As clinically indicated			X		Section 8.2.1
TSH (reflex free T3 or Total T3, or free T4) [k]	X [l]	X	X			Section 8.2.1
Urinalysis	As clinically indicated					Section 8.2.1
Hepatitis B and C	As clinically indicated					Section 8.2.1
Pregnancy test [m]	X	X	X	X		Section 8.2.1
**Pharmacokinetics**						
Durvalumab PK sample [n]	X (pre-dose) X (post-dose)	X (pre-dose)	X (C4) (pre-dose) X (C4) (post-dose)			Section 8.5.1
Dato-DXd PK sample [n]	X (pre-dose) X (post-dose)	X (pre-dose)	X (C4) (pre-dose) X (C4) (post-dose)			Section 8.5.1
**Monitoring**						
WHO/ECOG performance status	X	X	X	X		Section 8.2.6
AE/SAE assessment [o]	X	X	X	X		Section 8.3

	C1	C2	C3 and C4	Pre-surgical assessments	Surgery	For details, see CSP Section or Appendix
**Week**	**0**	**Q3W + 3 days [a]**		**Within 30 days before surgery**	**Within 40 days of the last dose of study interventions**	
**Day**	**1 [b]**	**q21d + 3 days [a]**				
**Pre-medication for Dato-DXd**						
Antihistamines and acetaminophen with or without glucocorticoids. Prophylactic anti-emetics.[p]	X					Section 6.1.1
**Study interventions administration [q]**						
Dato-DXd [r]	X	X	X			Section 6.1.1
Durvalumab	X	X	X			Section 6.1.1
Chemotherapy [s]	X	X	X			Section 6.1.1 and Table 6
**Other assessments and assays**						
Durvalumab immunogenicity assessment (ADA sampling to identify ADA responses in patient circulation) [n]	X (pre-dose)	X (pre-dose)	X (C4) (pre-dose)			Section 8.5.2
Dato-DXd immunogenicity assessment (ADA sampling to identify ADA responses) [n]	X (pre-dose)	X (pre-dose)	X (C4) (pre-dose)			Section 8.5.2
ctDNA [n]	X (pre-dose)	X (pre-dose)	X (pre-dose)	X		Section 8.6.3 and Section 8.7.1
Mandatory tumour and lymph nodes specimen					X	Section 8.6.1 and Section 8.6.2
Whole blood for CCI Expression [n]	X (pre-dose)	X (pre-dose)		X		Section 8.6.3

	C1	C2	C3 and C4	Pre-surgical assessments	Surgery	For details, see CSP Section or Appendix
Week	0	Q3W + 3 days [a]		Within 30 days before surgery	Within 40 days of the last dose of study interventions	
Day	1 [b]	q21d + 3 days [a]				
Serum samples for circulating soluble factors [a]	X (pre-dose)	X (pre-dose)		X		Section 8.6.3
Plasma samples for circulating soluble factors [a]	X (pre-dose)	X (pre-dose)		X		Section 8.6.3
Whole blood for [redacted] analyses [a]	X (pre-dose)					Section 8.6.3
Whole blood for PBMC [a]	X (pre-dose)	X (pre-dose)		X		Section 8.6.3
[redacted] Initiative optional, exploratory [redacted] blood sample [f]	X					Section 8.7.2 and Appendix D
**Efficacy evaluation**						
mPR and pCR determined by central pathology review					X	Section 8.1
RECIST 1.1 tumour assessments on CT and/or MRI scans	A RECIST 1.1 tumour assessment is performed after Cycle 2 and upon completion of the neoadjuvant period, prior to surgery.					Section 8.1.1 and Appendix F

[a] Subsequent time between 2 consecutive doses cannot be less than 21 days, based on the half-life of durvalumab.

[b] Every effort should be made to minimise the time between randomisation and starting treatment (ie, within 1 day and not more than 3 days of randomisation).

[c] As per investigator judgment, a dental evaluation before study intervention initiation and dental treatment if indicated, may reduce the risk of local and systemic infections from odontogenic sources. Participants will be provided an oral care plan prior to and during study treatment.

[d] Ophthalmologic assessments including, but not limited to, visual acuity testing, slit lamp examination, intraocular pressure measurement, fundoscopy, and fluorescein staining will be performed at screening by an ophthalmologist, or if unavailable, another licensed eye care provider. Please refer to the Dato-DXd Ophthalmologic Assessment Manual for further details.

[e] Body weight is recorded at each visit along with vital signs.

[f] Any clinically significant abnormalities detected require triplicate ECG results.

[g] A CT and PET scan should be performed pre-surgery.

[h] Pulmonary function testing may be repeated at Investigator's or surgeon's discretion prior to surgery to inform fitness for surgery.

[i] Serum or plasma clinical chemistry (including LFT monitoring) and haematology may be performed more frequently based on the local clinical practice or the Investigator's discretion, or if clinically indicated. Results for LFTs, electrolytes, full blood count, and creatinine must be available before commencing an infusion (within 3 days) and reviewed by the treating physician or Investigator prior to dosing. Cortisol testing should be performed at the pre surgical visit.

[j] If screening clinical chemistry and haematology assessments are performed within 3 days prior to Day 1 (first infusion day), they do not need to be repeated on Day 1.

[k] Free T3 or Total T3, or free T4 will only be measured if TSH is abnormal or if there is clinical suspicion of an AE related to the endocrine system.

[l] If TSH is measured within 14 days prior to Day 1 (first infusion day), it does not need to be repeated on Day 1.

[m] For women of childbearing potential only. A urine or serum pregnancy test is acceptable. Women of childbearing potential are required to have a pregnancy test within 3 days prior to the first dose of study interventions and then within 3 days prior to subsequent study interventions. Pregnancy test may occur on Day 1, but results must be available and reviewed by the treating physician or Investigator prior to commencing an infusion.

[n] Pre-dose collection: within 60 minutes prior to administration of any study intervention. Post-dose collection (durvalumab, Dato-DXd): end-of-infusion collection to be taken within 5 hours after the end of the respective study interventions infusion.

[o] For AEs/SAEs reported during screening, additional information such as medical history and concomitant medications may be needed.

[p] Premedication is required prior to any dose of Dato-DXd and must include antihistamines and antipyretics, preferably acetaminophen, with or without glucocorticoids to mitigate infusion reaction. Additionally, prophylactic anti-emetics are highly recommended prior to Dato-DXd dosing and on subsequent days.

[q] The following sequence should be used for treatment administration in days when all or more than one agent is administered: Dato-DXd > durvalumab > single agent platinum chemotherapy.

[r] Dato-DXd will be administered at 6 mg/kg IV Q3W on Day 1 of each 21-day cycle Refer to Section 8.3.15 for TMGs.

[s] Single agent platinum chemotherapy (physician choice of carboplatin or cisplatin).

[t] The sample for [redacted] research will be obtained on Day 1 pre-dose (at or after randomisation). If, for any reason, the sample is not drawn on Day 1, it may be taken at any visit until the last study visit. Only 1 sample should be collected per patient for [redacted] analysis during the study.

Note: Each cycle during the neoadjuvant treatment is 21 days (3 weeks) in duration, unless dosing needs to be held for toxicity reasons.
Note: All assessments on treatment days are to be performed prior to infusion, unless otherwise indicated.
Note: Surgery is expected within 40 days from the last dose of study interventions. Cases where surgery cannot be completed within 40 days from the last dose of study interventions should be discussed with the Study Physician.
Abbreviations: ADA: Anti-drug antibody; AE: Adverse event; aPTT: Activated partial thromboplastin time; C: Cycle; CSP: Clinical study protocol; CT: Computed tomography; ctDNA: Circulating tumour DNA; DNA: Deoxyribonucleic acid; ECG Electrocardiogram; ECHO: Echocardiogram; ECOG: Eastern Cooperative Oncology Group; INR: International normalised ratio; IV: Intravenous; LFT: Liver function test; mPR: Major pathological response; MRI: Magnetic resonance imaging; MUGA: multigated acquisition scans; PBMC: Peripheral blood mononuclear cells; pCR: Pathological complete response; PET: Positron emission tomography; PK: Pharmacokinetic(s); q21d: Every 21 days; Q3W: Every 3 weeks; RECIST: Response Evaluation Criteria in Solid Tumours; SAE: Serious adverse event; T3: Triiodothyronine; T4: Thyroxine; TMGs: Toxicity management guidelines; TSH: Thyroid-stimulating hormone; WHO: World Health Organisation.

Table N27 Schedule of Activities for Adjuvant Treatment for Patients Randomised to Arm 4

	Post-surgery, prior to starting treatment	C1	C2	C3 to C12	For details, see CSP Section or Appendix
Week	Within 10 weeks of surgery [a]	0	4	Q4W ± 3 days	
Day		1	29 ± 3	q28d ± 3 days	
**Study procedures**					
Physical examination (full)		X			Section 8.2.2
Targeted physical examination (based on symptoms)			X	X	Section 8.2.2
Vital signs [b]		X	X	X	Section 8.2.3
ECHO/MUGA		As clinically indicated			Section 8.2.5
ECG [c]		X	As clinically indicated		Section 8.2.4
Concomitant medications	X	X	X	X	Section 6.5
Post-surgery conditions	X				Section 6.1.3
**Laboratory assessments**					
Clinical chemistry [d]		X [d]	X [d]	X [d]	Section 8.2.1
Haematology [d]		X [d]	X [d]	X [d]	Section 8.2.1
aPTT and INR	As clinically indicated				Section 8.2.1
TSH (reflex free T3 or Total T3, or free T4) [e]		X	X	X	Section 8.2.1
Urinalysis		X	As clinically indicated		Section 8.2.1
Hepatitis B and C		As clinically indicated			Section 8.2.1
Pregnancy test [f]		X	X	X	Section 8.2.1

	**Post-surgery, prior to starting treatment**	**C1**	**C2**	**C3 to C12**	**For details, see CSP Section or Appendix**
**Week**	**Within 10 weeks of surgery [a]**	**0**	**4**	**Q4W ± 3 days**	
**Day**		**1**	**29 ± 3**	**q28d ± 3 days**	
**Pharmacokinetics**					
Durvalumab PK sample [g]		X (pre-dose) X (post-dose)	X (pre-dose)	X (C4) (pre-dose)	Section 8.5.1
**Monitoring**					
WHO/ECOG performance status		X	X	X	Section 8.2.6
AE/SAE assessment	X [h]	X	X	X	Section 8.3
**Study interventions administration**					
Durvalumab		X	X	X	Section 6.1.1
**Other assessments and assays**					
Durvalumab immunogenicity assessment (ADA sampling to identify ADA responses in patient circulation) [g]		X (pre-dose)	X (pre-dose)	X (C4 and C12) (pre-dose)	Section 8.5.2
ctDNA [g]		X (pre-dose)	X (pre-dose)	X (pre-dose)	Section 8.6.3 and Section 8.7.1
Serum samples for circulating soluble factors [g]		X (pre-dose)		X [redacted] (pre-dose)	Section 8.6.3
Plasma samples for circulating soluble factors [g]		X (pre-dose)		X [redacted] (pre-dose)	Section 8.6.3
Whole blood for PBMCs [g]		X (pre-dose)		X [redacted] (pre-dose)	Section 8.6.3

	Post-surgery, prior to starting treatment	C1	C2	C3 to C12	For details, see CSP Section or Appendix
Week	Within 10 weeks of surgery [a]	0	4	Q4W ± 3 days	
Day		1	29 ± 3	q28d ± 3 days	
Optional tumour biopsy specimen		X [i]			Section 8.6.1 and Section 8.6.4
**Efficacy evaluation**					
RECIST 1.1 tumour assessments (adjuvant period and beyond)	X [j, k]	Follow-up scans are acquired Q12W ± 1 week (relative to the date of surgery) until week 48; Q24W±2 weeks (relative to the date of surgery) until week 192 (approximately 4 years); and then Q48W ± 2 weeks (relative to the date of surgery) thereafter until RECIST 1.1-defined radiological PD, consent withdrawal, death, or study completion as determined by Sponsor. This on-study schedule MUST be followed regardless of any delays in dosing and regardless of tumour margins. These follow-up scans will use the original neoadjuvant screening scan as a baseline scan for RECIST 1.1 assessments.			Section 8.1.1 and Appendix F

a Patients will start treatment as soon as clinically feasible and within 10 weeks from surgery (except for patients receiving PORT, which must be started within 8 weeks after surgery).

b Body weight is recorded at each visit along with vital signs.

c Any clinically significant abnormalities detected require triplicate ECG results.

d Samples for laboratory assessment (serum or plasma clinical chemistry [including LFT monitoring] and haematology) may be obtained more frequently based on the local clinical practice or the Investigator's discretion. Results for LFTs, electrolytes, full blood count, and creatinine must be available before commencing an infusion (within 3 days) and reviewed by the treating physician or Investigator prior to dosing.

e Free T3 or Total T3, or free T4 will only be measured if TSH is abnormal or if there is clinical suspicion of an AE related to the endocrine system.

f For women of childbearing potential only. A urine or serum pregnancy test is acceptable. Women of childbearing potential are required to have a pregnancy test within 3 days prior to the first dose of study intervention and then Q4W prior to study interventions administration. Pregnancy test may occur on Day 1, but results must be available and reviewed by the treating physician or Investigator prior to commencing an infusion.

g Pre-dose collection: within 60 minutes prior to administration of any study intervention. Post dose collection (durvalumab): end-of-infusion collection to be performed within 5 hours after the end of the respective study interventions infusion.

h Must include all AEs that occur after surgery regardless of decision to start adjuvant treatment.

i It is strongly recommended/encouraged to collect additional tumour biopsies, if they become available at disease progression or unscheduled procedures during the study.

j A first post-surgical CT/MRI scan of the chest and abdomen (including the entire liver and both adrenals) must be acquired 5 weeks ± 2 weeks after surgery and prior to, but as close as possible to, the start of adjuvant treatment. This scan is used to determine eligibility to receive adjuvant treatment.

k A new primary malignancy confirmed by pathology is not considered an EFS or DFS event.

Note: All assessments on treatment days are to be performed prior to infusion, unless otherwise indicated.
Note: Patients should be able to start study interventions following surgery as soon as clinically feasible (unless PORT is given) and within 10 weeks from surgery.
Abbreviations: ADA: Anti-drug antibody; AE: Adverse event; aPTT: Activated partial thromboplastin time; C: Cycle; CSP: Clinical study protocol; CT: Computed tomography; ctDNA: Circulating tumour DFS: disease-free survival; DNA; DNA: Deoxyribonucleic acid; ECHO: Echocardiogram; ECG: Electrocardiogram; ECOG: Eastern Cooperative Oncology Group; EFS: Event-free survival; INR: International normalised ratio; LFT: Liver function test; MUGA: multigated acquisition scans; MRI: Magnetic resonance imaging; PBMC: Peripheral blood mononuclear cells; PD: Progression of disease; PK: Pharmacokinetic(s); PORT: Post operative radiation therapy; Q12W: Every 12 weeks; Q24W: Every 24 weeks; q28d: Every 28 days; Q4W: Every 4 weeks; RECIST: Response Evaluation Criteria in Solid Tumours; SAE: Serious adverse event; T3: Triiodothyronine; T4: Thyroxine; TSH: Thyroid-stimulating hormone; WHO: World Health Organisation.

N 2 Disease Target

N 2.1 Programmed Cell Death Ligand-1

Programmed cell death protein-1, PD-L1, and PD-L2 are part of a complex system of receptors and ligands that control T cell activation. Programmed death-ligand 1 expression helps tumours evade detection and elimination by the immune system (Chen and Mellman, 2013, Juneja et al, 2017, Keir et al, 2008, Ohaegbulam et al, 2015). The binding of PD-L1 to PD-1 on activated T cells delivers an inhibitory signal preventing T cells from killing target tumour cells (Ohaegbulam et al, 2015, Okazaki et al, 2013, Pardoll, 2012, Wu et al, 2020, Yu et al 2009

Yu X, Harden K, Gonzalez LC, Frencesco M, Chiang E, Irving B, et al. The surface protein TIGIT suppresses T cell activation by promoting the generation of mature immunoregulatory dendritic cells. Nat Immunol. 2009;10(1):48-57.

Zou and Chen, 2008, Zou et al, 2016).

N 2.2 TROP2

Human trophoblast cell-surface antigen-2 is a transmembrane glycoprotein that was first discovered in human trophoblast cells, which are highly invasive cells that originate from the outer layer of the blastocyst and develop into a large portion of the placenta. It was later rediscovered as a tumour-associated calcium signal transducer-2 and found to be encoded by the TACSTD2 gene (Goldenberg et al, 2015, Goldenberg et al, 2018).

Although the role of TROP2 is not yet fully understood, it is thought to participate in growth and proliferation of carcinoma cells. It is also believed to be a true oncogene involved in initiating signalling mechanisms that can result in increased tumourigenicity, aggressiveness, and metastasis (Cubas et al, 2009). Human trophoblast cell-surface antigen-2 is highly expressed on several epithelial tumours including NSCLC, making it a promising molecular target for therapeutic development.

N 3 Background for Study Intervention

N 3.1 Durvalumab

Durvalumab is a human mAb of the IgG kappa subclass that blocks the interaction of PD-L1 (but not PD-L2) with PD 1 on T cells and CD80 (B7.1) on immune cells. It is being developed by AstraZeneca for use in the treatment of cancer. Blockade of PD-L1/PD 1 and PD-L1/CD80 interactions releases the inhibition of immune responses, including those that may result in tumour elimination.

Durvalumab (Imfinzi®) has been approved in the US and EU to treat unresectable Stage III NSCLC, and extensive-stage small cell lung cancer in combination with etoposide and either

carboplatin or cisplatin in first-line treatment. Durvalumab in combination with tremelimumab and platinum-doublet chemotherapy is approved by the FDA for treatment of patients with metastatic NSCLC. Refer to the durvalumab IB and to the package insert (or label) for your specific country, as applicable.

N 3.2 Dato-DXd

Dato-DXd (DS-1062a) is an ADC comprised of a recombinant humanised anti-TROP2 IgG1 monoclonal antibody, MAAP-9001a, which is covalently conjugated via a cleavable drug linker, MAAA 1162a (the complex of MAAA- 1181a and a maleimide tetrapeptide linker), using thioether bonds to the topoisomerase I inhibitor MAAA-1181a. This tumour selective cleavable drug-linker is stable in the systemic circulation. Dato-DXd binds to the transmembrane glycoprotein TROP2 (also known as TACSTD2), which is highly expressed on the cell surface of epithelial tumours including NSCLC and is internalised. The drug component of Dato DXd, MAAA 1181a (a DNA topoisomerase I inhibitor), is released by enzymatic processes after internalisation, leading to inhibition of tumour growth and apoptosis of the target tumour cells and neighbouring tumour cells via the inhibition of DNA topoisomerase I. Further details of the non-clinical and clinical experience with Dato-DXd are provided in the IB.

N 4 Rationale for Combining Dato-DXd with Durvalumab and Single Agent Platinum Chemotherapy

Pre-clinical studies evaluating the combination of the ADC trastuzumab deruxtecan containing topoisomerase 1 payload similar to Dato-DXd with an anti-PD-1 antibody demonstrated better antitumour activity than administered as monotherapy, possibly due to increased T-cell activity and upregulation of PD-L1 expression induced by the ADC.

In addition, the clinical safety and efficacy of Dato-DXd is currently being evaluated in a Phase Ib study of patients with advanced or metastatic NSCLC (TROPION-Lung04). Preliminary safety data (TROPION-Lung04 study) support the evaluation of Dato-DXd 6 mg/kg in combination with durvalumab and carboplatin in further clinical trials. As of 28 September 2022, 35 patients received treatment with Dato-DXd + durvalumab, with 11 of the patients also receiving carboplatin. Overall, no new safety signals were observed and the AE profile of the Dato-DXd and durvalumab doublet, and Dato-DXd and durvalumab plus carboplatin triplet therapy was as expected relative to Dato-DXd monotherapy (Study DS1062 A J101) and the safety profiles of the individual treatments. Furthermore, when compared to the initial results of the TROPION Lung02 study (a study of doublet and triplet treatment with Dato-DXd plus pembrolizumab, with or without platinum-based chemotherapy in patients with advanced NSCLC [NCT04526691]), the safety profile observed was generally comparable in terms of the nature and frequency of TEAEs (Levy et al, 2022).

It is expected that combining 2 DNA damage agents (topoisomerase I inhibitor payload from Dato-DXd and platinum) could have a synergistic tumour killing effect, and together, these data support that Dato-DXd in combination with durvalumab and carboplatin has the potential to provide meaningful clinical benefit to patients with NSCLC.

N 5 Justification for Dose for Study Interventions Used in Arm 4

N 5.1 Durvalumab

This study will utilise durvalumab (1500 mg Q3W) for 4 cycles prior to surgery, followed by durvalumab (1500 mg Q4W) for an additional 12 cycles after surgery.

Rationale for proposed 3 weeks fixed dosing pre-surgery:

The proposed dosing schedule is aligned with the standard fixed dosing of 1500 mg durvalumab for 4 cycles, which is supported by efficacy and safety as well as tolerability data across multiple studies in multiple tumour types. The 1500 mg Q3W for the first 4 cycles (prior to surgery) is selected to conform to the platinum doublet chemotherapy schedule in the study. Based on simulated durvalumab PK profiles, the overall distribution of durvalumab exposure at 1120 mg Q3W was expected to be consistent with the predicted levels at 1500 mg Q4W. Although predicted exposures of 1500 mg Q3W were predicted to be slightly higher compared to 1120 mg Q3W, the relative increase in dose density of durvalumab (ie, 1500 mg Q3W instead of Q4W or 1120 mg Q3W) is supported by the fact that durvalumab has a flat exposure-response (safety) relationship with clinically used dose levels, and PK modelling reveals no clinically meaningful differences in drug levels.

Rationale for proposed 4 weeks fixed dosing post-surgery:

Durvalumab dosing regimen of 1500 mg Q4W is the approved dosing regimen in different indications including unresectable Stage III NSCLC.

For additional details on the nonclinical and clinical data that informed durvalumab dose selection, see the durvalumab IB.

N 5.2 Dato-DXd

The selected dose for Dato-DXd in this study is 6.0 mg/kg Q3W based on Dato-DXd preliminary results of an ongoing Phase 1 study (DS1062-A-J101 [TROPION-PanTumor01]; NCT03401385) in patients with solid tumours. In this study, Dato-DXd showed a tolerable safety profile across a dose range of 0.27 mg/kg to 8.0 mg/kg Q3W. During the dose escalation phase, the non-tolerated dose for Dato-DXd was 10.0 mg/kg. The maximum tolerated dose (8.0 mg/kg) was determined during the dose escalation phase.

Substantiating the use of the 6 mg/kg dose level, Dato-DXd has an important identified risk of ILD/pneumonitis that can have a life threatening or fatal outcome. As of the IB DCO date of

16 November 2023, a total of 27 (5.6%) patients experienced ILD/pneumonitis of any grade. Dato-DXd-related ILD/pneumonitis with a fatal outcome was reported in 4 (0.8%) patients in Study DS1062-A-J101 (TROPION-PanTumor01, Garon et al, 2021), and 3 out of the 4 patients received 8.0 mg/kg. A total of 219 (45.1%) patients experienced ≥ Grade 3 TEAEs. The most commonly (> 3% of patients) reported ≥ Grade 3 events were anaemia (5.8%), lymphocyte count decreased (4.1%), and stomatitis (4.1%). Toxicity management guidelines are in place to address toxicity.

Dose-dependent effects and trends were observed, with a larger proportion of patients in higher dose groups experiencing TEAEs that were severe, serious, and which resulted in dose interruption, reduction, and discontinuation (particularly stomatitis), compared with patients in lower dose groups.

In addition, an exposure-response analysis of the DS1062-A-J101 (TROPION-PanTumor01) study data, including response, sum of diameters, PFS and key AE (eg, stomatitis/mucosal inflammation) endpoints, suggested that the 6.0 mg/kg Q3W dose had better efficacy than the 4.0 mg/kg Q3W dose of Dato-DXd. At the 8.0 mg/kg Q3W dose, additional safety liability with no significant improvement in efficacy relative to the 6.0 mg/kg Q3W dose of Dato-DXd was observed. Overall, based on preliminary safety and efficacy data, the 6.0 mg/kg dose was considered better tolerated than the 8.0 mg/kg dose. Hence, 6.0 mg/kg is the optimal dose for further development of Dato-DXd in clinical studies.

The mean terminal half-life of Dato-DXd was 4.62 days at the 6.0 mg/kg dose and 4.51 days at the 4.0 mg/kg dose, which supports the Q3W dosing schedule chosen.

For additional details on the nonclinical and clinical data that informed Dato-DXd dose selection, see the Dato-DXd IB.

N 6 Study Intervention

N 6.1 Treatment Regimen

Patients will receive treatment as shown below.

	**Neoadjuvant treatment/Pre-Surgery (4 cycles)**	**Adjuvant treatment/Post-Surgery (1 year)**
**Arm 4**	Dato-DXd + durvalumab + single agent platinum chemotherapy (Q3W)	Durvalumab (Q4W)

Premedication is required prior to any dose of Dato-DXd, that must include antihistamines and antipyretics, preferably acetaminophen, with or without glucocorticoids. Based on currently available clinical data, it is highly recommended that patients receive prophylactic anti-emetic agents prior to infusion of Dato-DXd and on subsequent days as needed.

Antiemetics such as 5-HT3 antagonists and steroids (eg, dexamethasone) should be considered and administered in accordance with the prescribing information or institutional guidelines. Neurokinin-1 receptor antagonists can be used, if needed. Patients should remain at the site for at least 30 minutes post infusion of every dose of Dato-DXd for close observation for possible allergic reaction and IRR.

To mitigate ocular surface events (eg, dry eye, decreased or blurred vision, photophobia, keratitis, corneal ulcer), patients should be advised to use artificial tears 4 times daily as preventative measures and up to 8 times daily as clinically needed and to avoid the use of contact lenses. The use of other eye medications (eg, topical corticosteroids) for prophylaxis should be at the discretion of an ophthalmologist, or if unavailable, another licensed eye care provider.

Oral mucositis/stomatitis prophylaxis is also required prior to Dato-DXd dosing and during the duration of treatment. Participants will be provided an oral care kit prior to and during study treatment. It is recommended that patients randomised to Arm 4 should adhere to the following oral care plan guidance:

- Gently brush their teeth after meals and at bedtime using a soft or ultra-soft toothbrush (or swab) and a bland-flavoured fluoride-containing toothpaste.
- Floss their teeth every day, if able to do so without pain or causing gingival bleeding.
- Daily use of prophylaxis with a steroid-containing mouthwash (eg, dexamethasone oral solution 0.1 mg/mL 10 mL 4 times daily swish for 1 to 2 minutes then spit out; or a similar mouthwash regimen using an alternative steroid advocated by institutional/local guidelines) is highly recommended. Note: Patients are allowed to take oral nystatin suspension or other topical antifungal agents after the beginning steroid-containing mouthwash according to clinician preference based on institutional/local guidelines.
- In the absence of a prophylactic steroid-containing mouthwash, daily use of inert, bland mouth rinses (eg, with a non-alcoholic and/or bicarbonate-containing mouthwash, 4 to 6 times a day) is recommended.
- Prophylactic cryotherapy (ice chips or ice water held in the mouth throughout the infusion) should also be considered.
- The following algorithm, to be followed from steps 1 to 4, may be used as a guidance to select an appropriate prophylaxis mouthwash:
  (b) Dexamethasone mouthwash formulated at 0.5 mg/5 mL. If not available, then use
  (c) Dexamethasone mouthwash compounded at site/locally. If not available, then use
  (d) Other steroid-based mouthwash available at site/locally. If not available, then use
  (e) Non-steroid mouthwash or other local mouthwash.

Further recommendations for preventing and treating oral mucositis and stomatitis are available in the TMGs.

Premedication for chemotherapy should be administered according to the local label and guidelines. Details of any premedication or concomitant medication given to manage or prevent AEs should be recorded on the eCRF.

A physician must be present at the site or immediately available to respond to emergencies during all administrations of study interventions. Fully functional resuscitation facilities should be available.

N 6.2 Storage of Investigational Products

Dato-DXd and durvalumab vials are stored at 2°C to 8°C (36°F to 46°F) and must not be frozen. The investigator, or an approved representative (eg, pharmacist), will ensure that all study interventions are stored in a secured area and in accordance with applicable regulatory requirements. A temperature log will be used to record the temperature of the storage area. Temperature excursions outside the permissible range listed in the clinical supply packaging are to be reported to the monitor upon detection. A calibrated temperature-monitoring device will be used to record the temperature conditions in the drug storage facility. Storage conditions stated in the IB may be superseded by the label storage.

Study interventions must be kept in original packaging until time of preparation to prevent prolonged light exposure.

N 6.3 Investigational Product Preparation and Administration

N 6.3.1 Preparation

Each vial selected for dose preparation should be inspected. If there are any defects noted with the Investigational Product, the investigator and site monitor should be notified immediately.

The use of elastomeric pumps and pneumatic tube transport with Dato-DXd and durvalumab have not been studied, and AstraZeneca must be contacted prior to potential usage.

Doses of Dato-DXd and durvalumab for administration must be prepared by the Investigator's or site's designated investigational product manager using aseptic technique in compliance with local regulations and site requirements.

Total time from needle puncture of the **Dato-DXd** vial to the start of administration must not exceed 24 hours at 2°C to 8°C (36°F to 46°F), otherwise a new dose must be prepared from new vials.

The total time from needle puncture of the **durvalumab** vial to the start of administration

must not exceed 24 hours. Of this time, not more than 4 hours may be at room temperature, with the remaining time at 2°C to 8°C (36°F to 46°F), otherwise a new dose must be prepared from new vials.

Dato-DXd and durvalumab vials do not contain preservatives; any unused portion of the vial must be discarded immediately after use.

Following preparation and during administration, the prepared **Dato-DXd** IV bag must be covered by a light protection cover; the cover must be applied immediately after dose preparation and remain on throughout the administration time.

Dato-DXd doses will be prepared using an IV bag containing 5% dextrose for injection.

Note: Dato-DXd is incompatible with 0.9% sodium chloride for injection and this must not be used.

Durvalumab doses will be prepared using an IV bag containing 0.9% sodium chloride for injection or 5% dextrose for injection.

Refer to the Pharmacy Manual for detailed information about preparation of Dato-DXd and durvalumab.

N 6.3.2 Administration

Dato-DXd and durvalumab infusions are to be administered through an IV administration set with a 0.2- or 0.22-µm filter; acceptable configurations include an IV set containing an in-line filter or the attachment of a separate filter to the distal end of the IV tubing.

The **Dato-DXd** infusion time is approximately 90 minutes ± 10 minutes for the first infusion. If the first infusion is well tolerated and the patient does not experience an infusion related reaction, then the minimum infusion time for subsequent cycles is 30 minutes. However, if there are interruptions, the total cumulative time **from needle puncture of the vial to end of administration** must not exceed 4.5 hours with the infusion bag kept at room temperature, otherwise a new dose must be prepared from new vials.

The **durvalumab** infusion time is 1 hour ± 10 minutes; however, if there are interruptions, the total allowed time must not exceed 8 hours with the infusion bag kept at room temperature, otherwise a new dose must be prepared from new vials.

Do not co-administer other drugs through the same infusion line.

The IV line will be flushed with 5% dextrose injection according to local practices to ensure the full dose is administered. Infusion time does not include the final flush time.

N 6.4 Dose Reduction

Up to 2 dose reductions will be permitted for patients receiving Dato-DXd. Once the dose of Dato-DXd has been reduced because of toxicity, all subsequent cycles should be administered at that lower dose level unless further dose reduction is required (Table N28). Once the dose of Dato- DXd is reduced, no dose re-escalation will be permitted. More than 2 dose reductions are not allowed, and Dato-DXd will be discontinued if further toxicity that meets the requirement for dose reduction occurs.

Table N28 Dose Reduction Levels of Dato-DXd

Starting Dose	Dose Level 1	Dose Level 2
6.0 mg/kg	4.0 mg/kg	3.0 mg/kg

Dato-DXd: datopotamab deruxtecan

Investigators should consider dose reductions or discontinuations of Dato-DXd according to the patient's condition and after discussion with the Study Physician (see Dato-DXd TMGs).

Dose will need to be recalculated if patient weight falls below 30 kg (durvalumab) or if weight changes by ≥ ± 10% (Dato DXd) (see Pharmacy Manual). After the recalculation, the updated patient's weight will be used as the new baseline weight. The site may follow local institutional policy for recalculating dose based on weight changes less than 10%.

N 7 Adverse Events of Special Interest Associated with Dato-DXd

For the Dato-DXd clinical program, based on the available pre-clinical data, current clinical developmental program, review of the cumulative literature, reported toxicities for drugs with similar monoclonal antibody and payload as Dato-DXd, and biological plausibility, interstitial lung disease (ILD)/pneumonitis, oral mucositis/stomatitis, mucosal inflammation other than oral mucositis/stomatitis, and ocular surface events are considered to be AESIs.

Refer to the current IB for a summary of preliminary clinical study data.

All AESIs, regardless of severity or seriousness, must be followed until either event resolution, end of study including any post-treatment follow-up, trial termination, withdrawal of consent, or patient death.

ILD/pneumonitis

Interstitial lung disease/pneumonitis is considered an important identified risk of Dato-DXd.

Interstitial lung disease/pneumonitis should be ruled out if a patient develops radiographic changes potentially consistent with ILD/pneumonitis or develops an acute onset of new or worsening pulmonary or other related signs/symptoms such as dyspnoea, cough, or fever.

If the AE is suspected to be ILD/pneumonitis, treatment with Dato-DXd should be delayed pending further evaluations as described in the Dato-DXd TMGs. Evaluations should include high-resolution CT, pulmonologist consultation (infectious disease consultation as clinically indicated), bronchoscopy and bronchoalveolar lavage if clinically indicated and feasible, pulmonary function tests (including forced vital capacity and carbon monoxide diffusing capacity) and pulse oximetry (SpO2), clinical laboratory tests (arterial blood gases if clinically indicated, blood culture, blood cell count, differential white blood cell count, C-reactive protein, COVID-19 test) and one blood sample collection for PK analysis as soon as ILD/pneumonitis is suspected, if feasible.

All events of ILD/pneumonitis, regardless of severity or seriousness, will be followed until resolution including after Dato-DXd discontinuation.

An autopsy in cases of Grade 5 ILD/pneumonitis is encouraged.

Recommendations for treating ILD/pneumonitis are available in the Toxicity Management Guidelines.

An independent ILD Adjudication Committee is responsible for reviewing all cases of potential ILD/pneumonitis (see Section 9.7). To ensure adequate and relevant independent evaluation, systematic additional data collection will be conducted for all cases that will be brought for adjudication. These additional data collections will cover a more in depth relevant medical history (eg, smoking, radiation, COPD, and other chronic lung conditions), diagnostic evaluation, treatment and outcome of the event. This data collection will be triggered based on a pre-defined list of PTs eligible for adjudication as described in the Event Adjudication Site Manual. Further details can be found in the ILD Adjudication Charter."

Oral Mucositis/stomatitis

Oral mucositis/stomatitis AEs are considered as identified risks associated with Dato-DXd treatment. Oral mucositis/stomatitis is considered as a separate AESI from mucosal inflammation other than oral mucositis/stomatitis. Recommendations for preventing and treating oral mucositis/stomatitis are available in the TMGs.

Mucosal Inflammation Other Than Oral Mucositis/stomatitis

Mucosal inflammation AEs are considered as potential risks associated with Dato-DXd treatment. Mucosal inflammation other than oral mucositis/stomatitis is considered as a separate AESI from oral mucositis/stomatitis. Recommendations for preventing and treating mucosal inflammation other than oral mucositis/stomatitis are available in the TMGs.

Ocular Surface Events

Ocular surface events (eg, dry eye, decreased or blurred vision, photophobia, keratitis, corneal

ulcer) is considered an AESI associated with Dato-DXd treatment. Dry eye is considered as an identified risk and keratitis as an important identified-risk within this AESI. Patients are advised to use artificial tears 4 times daily as preventative measures and up to 8 times daily as clinically needed and to avoid the use of contact lenses. Recommendations for preventing and treating ocular surface events are available in the TMGs.

N 7.1 Adverse Event Reporting Requirements for Dato-DXd

All ILD/pneumonitis events regardless of severity, Grade ≥ 3 ocular surface events, Grade ≥ 2 keratitis events (includes keratitis, punctate keratitis, and ulcerative keratitis), and Grade ≥ 2 oral mucositis/stomatitis and mucosal inflammation (other than oral mucositis/stomatitis) should be reported beyond the safety follow-up period.

The following types of events should be reported by the investigator in the EDC within 24 hours of awareness:

- All potential ILD/pneumonitis cases, including both serious and non-serious potential ILD/pneumonitis cases. Additionally, all events of ILD/pneumonitis should be reported beyond the 90 day safety follow-up period.
- Hepatic events (both serious and non-serious) which meet the potential Hy's Law criteria defined as an elevated ALT or AST ≥ 3 x ULN and an elevated TBL ≥ 2 x ULN, regardless if it is due to disease progression per investigator assessment, that may occur at different time points during the study conduct should always be reported to the Sponsor. These events must be reported within the eCRF with the investigator's assessment of seriousness, severity, causality, and a detailed narrative. If the patient discontinues Dato-DXd due to liver enzyme abnormalities, the patient will have additional clinical and laboratory evaluations as described in Appendix E of the protocol in order to determine the nature and severity of the potential liver injury.
- Grade ≥ 3 ocular surface events
- Grade ≥ 2 keratitis events (includes keratitis, punctate keratitis, and ulcerative keratitis)

Appendix R Country Specific Amendment

In accordance with local regulatory requirements, Arms 4 and 5 will not open for enrolment in Belgium, thus all details in this CSP that pertain specifically to treatment Arm 4 and Arm 5 do not apply to any site located in Belgium.

Appendix S Abbreviations

Abbreviation or Special Term	Explanation
ADA	anti-drug antibody
ADC	antibody-drug conjugate
AE	adverse event
AESI	adverse event of special interest
AJCC	American Joint Committee on Cancer
ALK	anaplastic lymphoma kinase
ALP	alkaline phosphatase
ALT	alanine aminotransferase
aPTT	activated partial thromboplastin time
AST	aspartate aminotransferase
AUC	area under the curve
BIPR	Blinded Independent Pathology Review
BOR	best objective response
BP	Blood pressure
CD	cluster of differentiation
CFR	Code of Federal Regulations
CI	confidence interval
CL	clearance
Cmax	maximum observed serum concentration
cN1	clinical N1
COVID-19	Coronavirus disease 2019
COPD	chronic obstructive pulmonary disease
CPI	Checkpoint inhibition
CR	complete response
CRO	contract research organisation
CrCL	calculated creatinine clearance
CSP	Clinical Study Protocol
CSR	Clinical Study Report
CT	computed tomography
CTCAE	Common Terminology Criteria for Adverse Events
CTIS	Clinical Trial Information System
CTLA-4	cytotoxic T-lymphocyte-associated antigen-4
ctDNA	circulating tumour DNA

Abbreviation or Special Term	Explanation
CTX	platinum doublet chemotherapy
CYP	cytochrome P450
Dato-DXd	datopotamab deruxtecan
DCO	data cut-off
DES	Data Entry Site
DFS	disease-free survival
DILI	drug-induced liver injury
DLCO	diffusing capacity of the lungs for carbon monoxide
DLT	dose limiting toxicity
DNA	deoxyribonucleic acid
DNAM-1	DNAX accessory molecule-1
EBUS	endobronchial ultrasonography
EC	Ethics Committee, synonymous to Institutional Review Board (IRB) and Independent Ethics Committee (IEC)
ECG	electrocardiogram
ECHO	Echocardiogram
ECOG	Eastern Cooperative Oncology Group
eCRF	electronic Case Report Form
EDC	electronic data capture
EFS	event-free survival
EGFR	epidermal growth factor receptor
EOS	end of study
EOT	end of treatment
ESMO	European Society for Medical Oncology
EU	European Union
EUS	oesophageal ultrasonography
FAAN	Food Allergy and Anaphylaxis Network
Fc	fragment crystallizable
FDA	Food and Drug Administration
FDG	18F-Fluoro-deoxyglucose
FEV1	forced expiratory volume in 1 second
FNA	fine-needle aspiration
FTIH	first time in human
GCP	Good Clinical Practice
GGT	gamma glutamyl transferase

Abbreviation or Special Term	Explanation
HBsAg	hepatitis B surface antigen
HBV	hepatitis B virus
HCV	hepatitis C virus
HIV	human immunodeficiency virus
HL	Hy's Law
HLA-E	HLA class I histocompatibility antigen, alpha chain E
HR	hazard ratio
HRCT	High resolution computed tomography
IASLC	International Association for the Study of Lung Cancer
IATA	International Airline Transportation Association
IB	Investigator's Brochure
ICF	informed consent form
ICH	International Council for Harmonisation
IEC	Independent Ethics Committee
International co-ordinating investigator	If a study is conducted in several countries the international co-ordinating investigator is the investigator co-ordinating the investigators and/or activities internationally.
Ig	immunoglobulin
ILD	interstitial lung disease
imAE	immune-mediated adverse event
IMP	Investigational medicinal product
IMRT	intensity-modulated radiation therapy
INR	International Normalised Ratio
IRB	Institutional Review Board
IRR	infusion-related reaction
IRT	Interactive Response Technology
ITIM	immunoreceptor tyrosine-based inhibitory motif
ITT	Intent to treat
IV	intravenous
LDH	lactate dehydrogenase
LIF	leukaemia inhibitory factor
LVEF	Left ventricular ejection fraction
mAb	monoclonal antibody
mPDAC	metastatic pancreatic duct adenocarcinoma
mPR	major pathological response

Abbreviation or Special Term	Explanation
MRI	magnetic resonance imaging
MUGA	multigated acquisition scans
mRNA	messenger RNA
NC	not calculated
NCCN	National Comprehensive Cancer Network
NCI	National Cancer Institute
NECL	nectin-like
NIAID	National Institute of Allergy and Infectious Diseases
NIMP	non-investigational medicinal product
NK	natural killer
NKG2a	natural killer group 2a
NR	not reached
NSCLC	non-small cell lung cancer
NTL	non-target lesion
OR	odds ratio
ORR	objective response rate
OS	overall survival
PBMC	peripheral blood mononuclear cells
pCR	pathological complete response
PD	progression of disease
PD-1	programmed death-1
PD-L1	programmed death-ligand 1
PD-L2	programmed death-ligand 2
PET	positron emission tomography
PFS	progression-free survival
PHL	Potential Hy's Law
PK	pharmacokinetic
PORT	post-operative radiation therapy
PR	partial response
PT	preferred term
PVR	poliovirus receptor
PVRIG	poliovirus receptor-related immunoglobulin domain protein
PVRL2	poliovirus receptor-related 2
Q12W	every 12 weeks
Q24W	every 24 weeks

Abbreviation or Special Term	Explanation
Q3W	every 3 weeks
Q4W	every 4 weeks
Q2W	every 2 weeks
QTcF	QT interval corrected by Fridericia's formula
RANKL	receptor activator of nuclear factor kappa-B ligand
RECIST 1.1	Response Evaluation Criteria in Solid Tumours, Version 1.1
RNA	ribonucleic acid
RO	receptor occupancy
RP2D	recommended Phase II dose
RTSM	Randomisation and Trial Supply Management
SAE	serious adverse event
SAP	Statistical Analysis Plan
SD	stable disease
SLFN11	Schlafen 11
SoA	Schedule of Activities
SoC	Standard of care
SOP	Standard Operating Procedure
SpO2	saturation of peripheral oxygen
SRC	Safety Review Committee
SUSAR	suspected unexpected serious adverse reaction
STAT3	signal transducer and activator of transcription 3
TACSTD2	tumour-associated calcium signal transducer 2
TACTILE	T cell activation, increased late expression
TBL	total bilirubin
TEAE	treatment-emergent adverse event
TIGIT	T cell immunoreceptor with Ig and ITIM domains
TIL	tumour-infiltrating lymphocytes
TL	target lesion
TMB	tumour mutational burden
TMG	toxicity management guideline
TOP1	topoisomerase 1
TPS	tumour proportion score
TPV	third party vendor
TRAE	treatment-related adverse event
TROP2	human trophoblast cell-surface antigen 2

Abbreviation or Special Term	Explanation
ULN	upper limit of normal
US	United States
VEGF	vascular endothelial growth factor
w/v	weight/volume
WHO	World Health Organisation
WT	Weight
1L	first line

Appendix T Protocol Amendment History

The Protocol Amendment Summary of Changes Table for the current amendment is located directly before the Table of Contents.

Amendment 1 (19 August 2021)

This amendment is considered to be non-substantial based on the criteria set forth in Article 10(a) of Directive 2001/20/EC of the European Parliament and the Council of the European Union because it neither significantly impacts the safety or physical/mental integrity of patients nor the scientific value of the study.

Overall Rationale for the Amendment:

This protocol was amended in response to information requests received by the FDA on the 30 July 2021 and 06 August 2021.

Section # and Name	Description of Change	Brief Rationale	Substantial/Non-substantial
Section 1.1 Synopsis and 4.1 Overall Design	Definition of the unacceptable delay to surgery and update to stopping rules added.	FDA information request.	Non-substantial
Section 1.3 Schedule of Activities, 8.2.1 Clinical Safety Laboratory Assessments	Cortisol testing added.	FDA information request.	Non-substantial
Section 6.1.2.2 Chemotherapy, 6.5 Concomitant Therapy, 6.6 Dose Modification	Clarification added to dosing of carboplatin, pemetrexed and cisplatin.	FDA information request	Non-substantial
Section 8.5.1 Pharmacokinetics	Clarification of inclusion of PK data analysis plan.	FDA information request	Non-substantial

Amendment 2 (24 August 2022)

Overall Rationale for the Amendment:

This protocol was amended to incorporate a new study arm in addition to clarifications and updates on the clinical study protocol (CSP). This amendment includes patients with stage IIIB disease (IIA-IIIB), based on latest version of American Joint Committee on Cancer (version 8) which reassigns T3 tumours with N2 lymph node involvement (T3N2M0) from

stage IIIA to stage IIIB. The updates are summarised below. Other minor editorial updates were made throughout. Administrative changes, such as formatting, updates to abbreviations, and punctuation corrections, are not presented in this summary.

Section Number and Name	Description of Change	Brief Rationale
Throughout the document	Addition of MEDI5752 arm. Updates to reflect this have been made to all relevant sections of the CSP.	To evaluate the safety and efficacy of MEDI5752 in combination with platinum doublet chemotherapy in early-stage resectable NSCLC.
Throughout the document	Update of chemotherapy language to platinum doublet chemotherapy.	To clarify chemotherapy regimens.
Throughout the document	Updated target population to include stage IIIB (IIA-IIIB).	To allow for inclusion of patients with N2 multistation disease and to account for changes in the latest version of AJCC (version 8) which reassigns T3 tumours with N2 lymph node involvement (T3N2M0) from stage IIIA to stage IIIB.
Section 1.1 Synopsis; Section 3 Objectives and Endpoints; Section 8.1 Efficacy Assessments; Section 9.4.2.2 Secondary Endpoints	Updated event-free survival definition.	Alignment with the FDA's event-free survival definition.
Section 1.1 Synopsis; Section 3 Objectives and Endpoints; Section 9.4.2.2 Secondary Endpoints	Removed "median of the overall survival", and updated to reflect, "If reached by the end of the study, the median OS will also be of interest.	Median overall survival may not be reached at study close-out given the expected duration of survival time in this disease setting.
Section 1.1 Synopsis; Section 4.1 Overall Design	New table was added, including description of treatments arms.	To add clarity in description of treatment arms.
Synopsis 1.1 Synopsis; Section 1.3 Schedule of Activities; Section 7.1 Discontinuation of Study Interventions	Details about the Follow-up of patients after completion/discontinuation of study interventions were updated.	Clarification on follow-up period after completion/discontinuation of study intervention.
Synopsis 1.1 Synopsis	Specified that patients will be followed up for survival status, until death, withdrawal of consent or the end of the study.	To collect more mature data from time to event endpoints such as overall survival.
Section 1.2 Schema, Figure 1	Added PD-L1 stratification to schema.	For clarification.

Section 1.2 Schema; Section 6.3.3 Methods for Assigning Treatment Groups	Addition of language clarifying randomisation method with dynamically changing allocation ratio of treatment assignment.	To allow for changes in the treatment allocation ratio over the course of the study.
Section 1.3 Schedule of Activities – Table 1, Table 2, Table 3, and Table 4, Table 5	Update of table titles and inclusion of separate tables for MEDI5752 treatment (neoadjuvant and adjuvant treatment), and respective details were updated.	To provide clarity and to accommodate new arm.
Section 1.3 Schedule of Activities; Section 8.2.5 Echocardiogram/MUGA.	Addition of ECHO/MUGA assessment during screening and 'as clinically indicated' during treatment.	To ensure inclusion of patients with adequate cardiac function and to align with Section 8.2.5 of the CSP.
Section 1.3 Schedule of Activities	Hepatitis B and C assessment was updated to reflect 'as clinically indicated' during treatment.	For clarification.
Section 1.3 Schedule of Activities	In Table 3, clarification of timepoints for ctDNA collection (from Cycle 4 to cycle 12), and in Table 5 and the footnote g, timepoint was clarified for the EOS visit.	To provide clarity on ctDNA collection.
Section 1.3 Schedule of Activities; Section 8.2.1 Clinical Safety Laboratory Assessments	Added total T3 to TSH assessments in Tables 1 and 2, Tables 3, 4, 5, and 9, and relevant footnote was updated.	Total T3 is added to allow flexibility for conducting thyroid testing per local guidelines and test availability.
Section 1.3 Schedule of Activities; Section 8.6.3 Blood Sample Collections for Exploratory Biomarker Analysis; Section 8.7.1 Blood Samples for [redacted] Analyses	Collection of serum and plasma samples for circulating soluble factors were added in Table 1, Table 2, Table 3, and Table 4, and whole blood sample for [redacted] analyses were added to Table 1 and Table 2, and details were clarified in the respective sections of the CSP.	To provide information on exploratory translational analyses for the study interventions.
Section 1.3 Schedule of Activities; Section 3 Objectives and Endpoints; Section 8.6.3 Blood Sample Collections for Exploratory Biomarker Analysis	Whole blood for flow cytometry analyses was added. Subsequently, exploratory analysis details related to the flow cytometry in patient receiving MEDI5752 were updated.	To provide information on exploratory translational analyses for MEDI5752.
Section 1.3 Schedule of Activities; Section 3 Objectives and Endpoints; Section 8.6.3 Blood Sample Collections for Exploratory Biomarker Analysis	In Table 1 and Table 2, additional collection of whole blood for gene expression was updated and subsequently text was added in exploratory analysis endpoint. In Table 1, Table 2, Table 3, Table 4 PBMCs samples were added. Table 6 for	To provide information on exploratory translational analyses for the study interventions.

	the exploratory analysis related to PBMC sample was updated.	
Section 1.3 Schedule of Activities; Section 6.3.1 Patient Enrolment and Randomisation	Increased PET scan window acceptability to 42 days prior randomisation . In Table 1 and Table 2, respective footnotes were updated.	To allow the use of PET scan done as per standard practice to be part of the screening procedures.
Section 1.3 Schedule of Activities	Table 1 and Table 2, footnote c was updated to reflect subsequent time between 2 consecutive doses duration for monalizumab and MEDI5752, respectively. Additionally, footnote l was updated to reflect the assessment of anti-thyroid antibodies.	For clarification.
Section 1.3 Schedule of Activities	In Table 1 and Table 2, footnote d language related to fine needle aspiration was updated.	To allow flexibility on the requirements for the collection of tumour biopsy, and for alignment with laboratory and pathology manual.
Section 1.3 Schedule of Activities; Section 8.2.1 Clinical Safety Laboratory Assessments	In Table 1 and Table 2, footnote related to clinical chemistry and haematology were updated. Subsequently Table 9 footnotes related to clinical chemistry and haematology were clarified for the respective arms.	For clarification and assessments for MEDI5752 were added to perform weekly monitoring of safety laboratories.
Section 1.3 Schedule of Activities	Footnote 'q' of Table 1 and Table 2 were updated to specify that post-dose collection is after the end of each study intervention infusion.	To capture the end of infusion PK point for each of the intervention.
Section 1.3 Schedule of Activities (Table 3 and Table 4); Appendix F: Appearance of New Lesions	Updated language related to bone scintigraphy.	General update.
Section 1.3 Schedule of Activities	In Table 3, It was specified that follow-up scans during adjuvant treatment and beyond will be used for assessing disease recurrence.	To clarify RECIST assessments after surgical resection are used primarily to monitor disease recurrences.
Section 1.3 Schedule of Activities	In Table 5, removed weight evaluation from Month 6.	General update.

Section 1.3 Schedule of Activities; Section 8.6 Human Biological Sample Biomarkers; Section 8.6.1 Baseline Tumour Samples for Biomarker Assessments; Section 8.6.4 Collection of Optional On-Treatment Tumour Tissue Samples or Biopsy at Progression	In Table 3 and Table 4, optional tumour biopsy specimen collection was added, according to footnote n of the tables. In Table 5, footnote h was clarified. Subsequently, Section 8.6.1 and Section 8.6.4 were updated with a language on the tumour biopsy requirements and flexibility with respect to needle gauge for sample collection.	To allow flexibility on the requirements for the collection of tumour biopsy, and for alignment with laboratory and pathology manual.
Section 1.3 Schedule of Activities; Section 4.1 Overall Design	In Table 3, Table 4, and Table 5, RECIST 1.1 tumour assessment and imaging related language was updated.	To extend imaging follow-up to capture EFS events based on emerging data.
Section 1.3 Schedule of Activities	In Table 5, ADA samples for all study interventions were added for EOT visit. In the same table, footnote 'a' was removed	General update and for clarification.
Section 2 Introduction; Section 4.2 Scientific Rationale for Study Design	Inclusion of additional literature references and according to updated study population and study treatment arm (Arm 3), details were updated.	To add more background to the study.
Section 2.4.2.1 Durvalumab;<br>Section 8.3.10.1 AESIs for Durvalumab Containing Regimens	New risk added: thyroiditis, encephalitis, meningitis.	To align with updates in durvalumab IB.
Section 3 Objectives and Endpoints	Updates in tertiary/exploratory endpoints.	To reflect addition of collection of serum samples for circulating soluble factors and PBMCs samples.<br>To reflect addition of collection of flow cytometry samples.
Section 4.3 Justification for Dose	Inclusion of further information from studies with MEDI5752.	To support selected dose.

Section 4.4 End of Study Definition; Section 6.7 Intervention after the End of the Study	Specified that end of study refers to completion of last scheduled visit or procedure.	To clarify the follow-up for survival and/or disease recurrence is longer than 12 months after completion/discontinuation of the study treatment.
Section 5.1 Inclusion Criterion #5	Removed language specifying patients must have 1 single nodal station ≤ 3 cm. Also, text included to reflect exclusion of patients with N3 disease.	This protocol version allows N2 disease regardless of extension of nodal station involvement. Hence this language has been updated.
Section 5.1 Inclusion criterion #7	Updated eligibility criteria to include LVEF and Troponin I or T	To align with safety requirements related to MEDI5752
Section 5.1 Inclusion criterion #10; Section 5.2, Exclusion criterion 22; Section 8.3.13 Pregnancy; Appendix A3 Informed Consent Process; Appendix G: Contraception requirements	Text related to contraception, breastfeeding and ova donation was updated for all study interventions.	General update to reflect contraception requirements with the study interventions.
Section 5.1 Inclusion criterion #12; Section 6.3.1 Patient Enrolment and Randomisation; Section 8.6.1 Baseline Tumour samples for Biomarkers Assessments; Section 1.3 Schedule of Activities	In inclusion criterion #12, tumour sample requirements for the new or archival tumour tissue were added. Criterion #12 (b) language related to EGFR mutation and ALK rearrangement was updated and requirement of patients with documented KRAS mutation with EGFR/ALK status was removed. Accordingly, Table 1 and Table 2, footnotes related to KRAS mutation were updated and in Section 6.3.1 respective details were updated and a statement about obtaining the tumour biopsy sample prior to the 28 days screening window in order to permit analysis was removed.	For clarification and general update.
Section 5.1 Inclusion criterion #14	Inclusion criterion #14 related to surgery eligibility (pneumonectomy,	General update with respect to the exclusion criterion 10.

	segmentectomies, or wedge resections) was updated.	
Section 5.2 Exclusion criterion #3	Wegener syndrome was removed.	General update.
Section 5.2 Exclusion criterion #5	Updated to include patients with other primary malignancies treated with curative intent for ≥3 years.	To align with the FDA guidance.
Section 5.2 Exclusion criterion #8	Added guidance for evaluating eligibility in the presence of infections.	To provide clarity
Section 5.2 Exclusion criterion #18.	Patients receiving anti-HLA-E agents are excluded.	To reflect exclusion of prior therapies targeting ligand of monalizumab.
Section 5.2 Exclusion criterion #19; Appendix H: Prohibited medications/therapies	Current or prior use of systemic corticosteroids dose was updated to ≤ 10 mg/day of prednisone or its equivalent.	Steroid adjustment to match with new arm (MEDI5752) requirement.
Section 5.3 Lifestyle Considerations	Number of days between receipt of the final dose of study interventions or until alternate anticancer therapy and blood donations updated to reflect the study intervention's requirements.	Align CSP with exclusion criteria.
Section 6.1.1 Investigational Products – Table 8	Updated chemotherapy section from IMP to NIMP designation.	Typo update.
Section 6.1.1 Investigational Products – Table 8	Updated to allow chemotherapy dosing to be rounded up to 10% at investigator discretion and institutional guidelines.	To allow flexibility and align with the clinical practice.
Section 6.1.1 Investigational Products – Table 8, Section 6.1.2.2 Chemotherapy	Updated carboplatin maximum dosing for AUC6 or AUC5.	Alignment with FDA's recommended maximum dose for carboplatin AUC calculations.
Section 6.1.1.1 Study Interventions	All study interventions supply, packaging, and preparation details were updated.	For alignment with the CSP update.
Section 6.1.1.2 Storage of Study Interventions	Updated to include safe storage, handling, and monitoring guidance.	To provide clarity.
Section 6.1.1.3 Study Intervention Preparation and Administration (Oleclumab Infusion Preparation)	Update on oleclumab concentration and IV bag size.	Concentration and bag size were updated based on endotoxin considerations.

Section 6.1.1.3 Study Intervention Preparation and Administration (Oleclumab Infusion Preparation)	Oleclumab infusion preparation details were updated to reflect weight cut-off of 35 kg.	For clarification.
Section 6.1.1.3 Study Intervention Preparation and Administration (Oleclumab Administration and Monalizumab Administration)	Total allowed infusion bag stability increased from 4 to 8 hours and infusion time changed from 1 hour ± 15 minutes to 1 hour ± 10 minutes.	To extend the allowed time of infusion to a total of 8 hours.
Section 6.1.1.3 Study Intervention Preparation and Administration (Monalizumab Infusion Preparation)	Updated Monalizumab dose from 750 mg to 1500 mg and updated infusion preparation instructions to align with 1500 mg dose.	Correct an error on monalizumab dosing and alignment with established study procedures.
Section 6.1.2.2 Chemotherapy	Updated chemotherapy language to allow Day 1 dose modifications. Also, language related to maximum dosing details were added.	General update. According to FDA guidance, details were updated.
Section 6.1.3 Surgery	Addition of cN2.	Per NCCN guidelines and inclusion of study population.
Section 6.3.2 Procedures for Handling Incorrectly Randomised Patients; Section 6.3.3 Methods for Assigning Treatment Groups	Language related to the procedures in case of incorrectly randomised patients was updated.	For clarification.
Section 6.5.1 Rescue Medications; Section 6.6 Dose modification; Section 6.6.1 Dose Delays	Rescue medication, dose delays, and dose modifications details were updated.	To provide clarity.
Section 8.1 Efficacy Assessment	Removal of "EFS event of PD discovered during surgery will be determined by the Investigator".	General update.
Section 8.2.1 Clinical Safety Laboratory Assessments	Addition of glycated haemoglobin and ferritin in Table 9, and WBC in Table 12.	To include additional laboratory tests.

Section 8.2.2 Physical Examination<br>Section 8.2.3 Vital Signs	Text was updated to include 'mouth' for full physical examination and 'SpO_2' for vital sign evaluation. Timepoint for collection of blood pressure and pulse reading was updated to 60 minutes ± 10 minutes at the end of the infusion.	For clarification and flexibility.
Section 8.3.15 Toxicity Management Guidelines	Details related to TMGs for each study interventions were clarified.	For clarification.
Section 8.5.1 Pharmacokinetics	Addition of 'plasma' samples.	General update.
Section 8.6.1 Baseline Tumour Samples for Biomarker Assessments	Tumour samples for exploratory biomarker assessment was updated to include 'HLA-E', 'proteogenomic profiling', and 'development of algorithms to predict pCR'.	To provide information on exploratory translational analyses.
Section 8.6.2 Surgical Tumour Samples for Assessments	New language added to exploratory biomarkers.	To provide information on exploratory translational analyses.
Section 8.6 Human Biological Sample Biomarkers; Section 8.6.3 Blood sample Collection of Mandatory Samples for Biomarker Analysis	Section title updated.	To clarify that these are mandatory samples.
Section 8.6.6	Sample storage duration was updated to 15 years from the CSR.	General update.
Section 9.2 Sample Size Determination	[redacted]	Typo update.
Section 9.3 Populations for Analysis	Interim safety population removed.	To reduce redundancy with the safety population language.
Section 9.4.2.1 Primary Endpoint; Section 9.4.2.2 Secondary Endpoints; Section 9.4.4.2 Biomarkers	[redacted]	To clarify how certain endpoints (mPR, pCR, and ORR) will be summarised.

Section 9.5	Futility criteria for number of pCRs changed from [redacted] patients.	The decision framework at the IA has been adjusted based on emerging data.
Appendix H1	Note has been added, reflecting use of phenytoin and fosphenytoin according to carboplatin summary of product characteristics.	For clarification.
Appendix J7	Language about patient's caregiver training and needed supplies and instruction for administration and documentation of study intervention was updated.	General update.

Abbreviations: ADA: anti-drug antibody; AEs: adverse events; AESI: adverse event of special interest; BIPR: blinded independent pathologist review; CD: cluster of differentiation; CI: confidence interval; CR: complete response; CSP: clinical study protocol; CSR: clinical study report; ctDNA: circulating tumour DNA; DFS: disease-free survival; EFS: event-free survival; EOT: end-of-treatment; EOS: end of study; FDA: Food and Drug Administration; HLA: human leukocyte antigen serotype; IASLC: International Association for the Study of Lung Cancer; IA: interim analysis; IMP: investigational medicinal product; mPR: major pathological response; NIMP: non-investigational medicinal product; NSCLC: non-small cell lung cancer; OS: overall survival; ORR: objective response rate; PBMC: peripheral blood mononuclear cells; pCR: pathological complete response; PET: positron emission tomography; PD: progression of disease; PD-L1: programmed cell death ligand-1; PK: pharmacokinetics; pCR: pathological complete response; PR: partial response; RECIST: Response Evaluation Criteria in Solid Tumours; SpO_2: saturation of peripheral oxygen; TMB: tumour mutational burden; TMG: toxicity management guideline; TSH: thyroid-stimulating hormone; T3: triiodothyronine; WBC: white blood cells.

Amendment 3 (16 December 2022)

Overall Rationale for Amendment

This protocol was amended to incorporate 2 new study arms in addition to clarifications and updates on the clinical study protocol (CSP). The structure of the protocol has also been reorganised by adding agent specific appendices to provide easier access to relevant information for each treatment arm in order to facilitate review by the Investigators. The updates are summarised below. Other minor editorial updates were made throughout. Administrative changes, such as formatting, updates to abbreviations, and punctuation corrections are not presented in this summary.

Section Number and Name	Description of Change	Brief Rationale
Throughout the document.	Addition of Arms 4 and 5. Updates to reflect this have been made to all relevant sections of the CSP.	To evaluate the safety and efficacy of Dato-DXd + durvalumab + single agent platinum chemotherapy in a neoadjuvant setting followed by durvalumab in an adjuvant setting in early stage resectable NSCLC. To evaluate the safety and efficacy of AZD0171 + durvalumab + platinum doublet chemotherapy in a neoadjuvant setting followed by AZD0171 + durvalumab in an adjuvant setting in early- stage resectable NSCLC.
	Re-organisation of information into treatment arm specific appendices.	To facilitate review by Investigators.
Sections 1.1 Synopsis.<br>Section 4.1 Overall Design.<br>Section 9.2 Sample Size Determination.	Sample size has been amended from 70 patients per arm to up to 70 patients per arm. The overall number of patients has been increased to 350.	To account for potential changes in the final sample size based on emerging safety and efficacy data.
Table 1 Schedule of Activities for the Screening Period: All Patients.<br>Section 8.2.7 Pulmonary Function Assessment.	Text added to table footnote to state that pulmonary function test done as part of clinical practice within 42 days prior to randomisation is acceptable and doesn't need to be repeated during screening.<br>Text added to table footnote to clarify that ECHO/MUGA is mandatory for Arm 3 and all patients must be assessed at screening when Arm 3 is open for enrolment. When Arm 3 is not open for enrolment, ECHO/MUGA can be done at the Investigator's discretion.	Update and clarification.

Section Number and Name	Description of Change	Brief Rationale
Table 1 Schedule of Activities for the Screening Period: All Patients.<br>Schedule of Activities tables for Arms 1-5.	Change in time that women of childbearing potential are required to have a pregnancy test to the first dose of study intervention – reduced from within 7 days to within 3 days.	Updated for all arms in order to match requirement for Arm 4.
Table 1 Schedule of Activities for the Screening Period: All Patients.<br>Table 2 Schedule of Activities for Patients who have Completed/Discontinued Treatment: All Patients.<br>Section 8.2.8 Ophthalmological Assessments.	Ophthalmological assessments added to Schedule of Activities.<br>New section added to describe ophthalmological assessments.	To include and provide details on the ophthalmological assessments that are required for Arm 4.
Section 3 Objectives and Endpoints.	Tertiary/exploratory endpoints updated to amend description of serum protein levels (renamed as circulating soluble factors from plasma and serum).<br>Biomarker expression updated to include LIF and TROP2.	Update.
Section 4.4 End of Study Definition.	End of study dates defined.	In accordance with updates to CSP template.
Section 5.1 Inclusion Criteria.	Updated inclusion criteria to clarify eligibility based on T4 tumours.<br>Addition of the following wording to inclusion criterion 7: red blood cell/plasma transfusion is not allowed within 1 week prior to screening assessment.	Clarification.<br><br>Required following addition of Arm 4.
Section 5.1 Inclusion Criteria.<br>Section 5.2 Exclusion Criteria.<br>Section 8.3.13.2 Paternal Exposure.<br>Appendix A3 Informed Consent Process.<br>Appendix G Contraception Requirements.	Change in contraception requirements after stopping MEDI5752.	Initial contraception requirements set to align with FDA guidance for genotoxic pharmaceuticals have been updated based on confirmation that MEDI5752 is non-genotoxic.

Section Number and Name	Description of Change	Brief Rationale
Section 5.2 Exclusion Criteria.	Addition of autoimmune pneumonitis and autoimmune myocarditis as examples of active or prior documented autoimmune or inflammatory disorders.<br>Addition of bleeding diseases as example of uncontrolled intercurrent illness.<br>Clarification that a major surgical procedure includes highly invasive dental procedures (note added that this only applies if Arm 5 is open for enrolment).<br>Addition of the following new exclusion criteria:<br>• History of non-infectious ILD/pneumonitis that required steroids, has current ILD/pneumonitis, or has suspected ILD/pneumonitis that cannot be ruled out by imaging at screening.<br>• History of allogeneic organ transplant.<br>• Patients with a history of severe hypersensitivity reactions to other monoclonal antibodies.<br>• Patients with clinically significant corneal disease (Note that this exclusion criterion only applies if Arm 4 is open for enrolment).<br>• Patients who have received previous treatment with a TROP2 targeting ADC or with another ADC containing a chemotherapy agent that inhibits TOP1 activity.<br>• Patients who have received previous Dato-DXd or AZD0171 assignment in the present study.	Clarification and update for new treatment arms.

Section Number and Name	Description of Change	Brief Rationale
Section 6 Study Intervention.	Table 5 Investigational Products updated with additional information.<br>Addition of separate columns to Table 6 Chemotherapy for single agent chemotherapy (required for Arm 4).	Updated information.
Section 6.3.1 Patient Enrolment and Randomisation.<br>Section 8.6.1 Baseline Tumour Samples for Biomarker Assessments.	Text added to clarify that with the exception of patients with squamous cell carcinoma, all patients will be tested for ALK.	Clarification.
Section 8.2.5 Echocardiogram/MUGA.<br>Section 8.6.3 Blood Sample. Collections for Biomarker Analysis.<br>Appendix M1 Schedule of Activities.	Information added to state that any blood samples for flow cytometry should be collected either before the MUGA scan or at least 2 days after the MUGA scan has been performed. Note added that ECHO/MUGA assessments are only required at screening if Arm 3 containing MEDI5752 is open for enrolment.	Update and clarification.
Section 8.3.10.1 Adverse Events of Special Interest for Durvalumab-containing Regimens.	Updates made to list of adverse events of special interest for durvalumab.	Clarification and update to align with latest durvalumab IB.
Section 8.3.14 Medication Error	Definition of medication error updated.	Updated following change to CSP template.
Section 8.5.2 Immunogenicity Assessments	Clarification added that blood samples for determination of ADA will be taken as serum or plasma.	Clarification.
Section 8.5.3 Storage and Destruction of Pharmacokinetic/ADA samples.	Text added to amend the time period that PK samples will be kept from 15 years to 6 months of finalisation of the bioanalytical report and to clarify that any residual back-up PK samples may be used for future exploratory biomarker research only if future use consent has been provided.	Update and clarification.
Section 8.6.1 Baseline Tumour Samples for Biomarker Assessments.	LIF and TROP2 added to list of biomarkers.	Update for addition of Arms 4 and 5.
Section 1.1 Synopsis.<br>Section 3 Objectives and Endpoints.<br>Section 8.1 Efficacy Assessments.<br>Section 9.4.2.2 Secondary Endpoints.	Text added to clarify definition of EFS events.	Clarification.

Section Number and Name	Description of Change	Brief Rationale
Appendix A1 Regulatory, Ethical, and Study Oversight Considerations.<br><br>Appendix A6 Dissemination of Clinical Study Data	Addition of requirements for reporting serious breaches.<br><br>Addition of text relating to the timing of the submission of results from the trial.	Updated following change to protocol template.
Appendix H1 Prohibited and Permitted Concomitant Medications/Therapies.	Text added to exclude anti-HLA-E and anti-LIF agents, treatment with a TROP2 targeting ADC or with another ADC containing a chemotherapy agent that inhibits TOP1.	Required for new treatment arms.
Appendix H1 Prohibited and Permitted Concomitant Medications/Therapies.	Deleted the following wording from Table 16 Prohibited medications/therapies: local treatment of isolated lesions, excluding TLs, for palliative intent is acceptable [eg, by local surgery or radiotherapy]).<br>Deleted paragraph allowing palliative therapy.	Change made to provide clarity and streamline allowed therapies in the study.
Appendix K1 Schedule of Activities.<br>Appendix L1 Schedule of Activities.	ECHO/MUGA assessments added to neoadjuvant tables.<br>aPTT and INR added to adjuvant table.	Update and clarification.
Appendix M1 Schedule of Assessments.	Clarification added to state that CT is only after Cycle 2.<br>Clarification added to state the visit days at which clinical chemistry tests will be required during the first 9 weeks (Day 1, Day 8, Day 15 of Cycle 1, 2, and 3).<br>pTT and INR added to adjuvant table.	Clarification.
Appendix M4 Rationale for Combining MEDI5752 with Chemotherapy.	Updated with latest data from ongoing study D7980C00001.	Update.

Section Number and Name	Description of Change	Brief Rationale
Minor updates and clarifications throughout the document	Text updated in Synopsis, Introduction, and Benefit Risk Assessment sections.<br>Deletion of "approximately" in requirement for surgery to be performed within "approximately" 40 days from last dose of study interventions.<br>Clarification to add missing wording ("*and continues to have clinical benefit*") from the neoadjuvant treatment period that treatment will be stopped at RECIST 1.1-defined radiological PD or clinical progression unless it is confirmed by the Investigator in agreement with the Study Physician/Medical Scientist that the patient continues to have a resectable tumour and continues to have clinical benefit.<br>Text added to state that for patients receiving more than one study intervention, the longest washout period must be followed after the last dose of study interventions (text already stated in other sections).<br>Text added to clarify the visit days at which specified laboratory parameters should be measured during the first 9 weeks for patients receiving MEDI5752.<br>Text added to clarify that the updated patient weight will be used as the new baseline weight in any dose recalculations.<br>Text added to clarify that in situations where the assessment qualifies multiple agents' TMGs for a particular toxicity, the more conservative TMG should be applied.<br>Clarification added that the ctDNA sample will be used to obtain buffy coat layers.	Minor updates and clarifications.

Abbreviations: ADA: anti-drug antibody; ADC: antibody-drug conjugate; ALK: anaplastic lymphoma kinase; aPTT: activated partial thromboplastin time; CSP: clinical study protocol; CT: computed tomography; ctDNA: circulating tumour DNA; Dato-DXd: datopotamab deruxtecan; ECHO: echocardiogram; FDA: Food and Drug Administration; HLA-5: HLA class I histocompatibility antigen, alpha chain E; ILD: interstitial lung disease; INR: International Normalised Ratio; LIF: leukaemia inhibitory factor; MUGA: multigated acquisition scans; NSCLC: non-small cell lung cancer; PD: progression of disease; PK: pharmacokinetics; RECIST: response evaluation criteria in solid tumours; TL: target lesion; TMG: toxicity management guideline; TOP1: topoisomerase 1; TROP2: human trophoblast cell-surface antigen 2.

Amendment 4 (28 November 2023)

Overall Rationale for Amendment

This protocol was amended to incorporate 2 additional study arms (3B and 3C) in addition to clarifications and updates on the clinical study protocol (CSP). The updates are summarised below, and details are provided in Appendix M. Other minor editorial updates were made throughout. Administrative changes, such as formatting, updates to abbreviations, and punctuation corrections are not presented in this summary.

List of Substantial Modifications

Section Number and Name	Description of Change	Brief Rationale
Section 1.1 Synopsis, Section 1.2 Schema, Section 4.1 Overall Design<br>Section 6.1.1 Investigational Products<br>Appendix M	Addition of two new treatment arms 3B and 3C (neoadjuvant/adjuvant treatment with volrustomig), with the re-naming of Arm 3 to Arm 3A.<br>• Arm 3A (previously Arm 3): Neoadjuvant: volrustomig [redacted] mg + CTX [redacted] Adjuvant: volrustomig [redacted] mg [redacted]<br>• Arm 3B: Neoadjuvant: volrustomig [redacted] mg + [redacted]<br>• Arm 3C: Neoadjuvant: volrustomig [redacted] mg + [redacted]<br>Updated sample size from 350 to 490, details of treatment modalities in treatment Arms 3A, 3B and 3C.	[redacted]<br>For details see Appendix M 5.

List of Non-substantial Modifications

Section Number and Name	Description of Change	Brief Rationale
Throughout the CSP	Naming for MEDI5752 used in previous CSP versions has been updated to volrustomig.	Update following the finalization of MEDI5752's INN.

Section Number and Name	Description of Change	Brief Rationale
Section 1.1 Synopsis	Discrepancies in EOT visit window clarified. Aligned to "30 days +/- 3 days since last dose or last study intervention (including surgery)".	Clarification.
Table 1 Schedule of Activities for the Screening Period	Footnote added for Dato-DXd to refer to Ophthalmologic Assessment Manual for details.<br>Rationale for change in pregnancy test prior to dosing from within 7 days to within 3 days was updated.	Clarification.<br>Updated in response to an Ireland Health Authority request.
Table 2 Schedule of Activities for Patients who have Completed/Discontinued Treatment	Minor updates in footnote a to add clarifications for initial EOT visits done outside the protocol specified window.	Clarification
Section 2.1 Disease Background, Section 2.3 Study Rationale, Section 2.4.1 Potential Benefits	Update from results of recently conducted clinical trials for treatment for resectable, early stage NSCLC.	Update of background information.
Section 2.4.2.1 Durvalumab	Additional risks for durvalumab monotherapy added (uveitis and immune-mediated arthritis).	Update.
Section 2.4.2.2 Combination Therapy with Durvalumab Plus Oleclumab, Monalizumab, Dato-DXd or AZD0171	Addition of the 2 most relevant risks considered for the benefit/risk assessment for durvalumab combination therapy (important identified risk of ILD/pneumonitis and identified risk of IRR). Embryo-foetal toxicity added as an important potential risk.	Update.
Section 4.4 End of Study Definition	Defined that arm-specific CSRs may be produced as each of the arms complete. A final integrated CSR will be written containing all arms.	Clarification on the reporting of study results.
Section 5.1 Inclusion Criteria	Updated inclusion criterion #7 (LVEF ≥ 50% as assessed by echocardiogram or MUGA scan). Added that this criterion only applies if Arms 3A, 3B, and 3C are open for enrolment.	Update.
Section 5.2 Exclusion Criteria	Updated exclusion criterion #9b (Known HIV infection that is not well controlled). Added that if an HIV infection meets the defined criteria, monitoring of viral RNA load and CD4+ count is recommended. If Arm 4 is open for enrolment, all participants must be tested for HIV during the screening period if acceptable by local regulations or an IRB.<br>Updated exclusion criterion #9c (ii) (Active or uncontrolled HBV or HCV, patients are eligible if they have normal transaminase values). Removed the previously defined criteria for patients with metastatic disease as they are not eligible for this study.	Update.

Section Number and Name	Description of Change	Brief Rationale
Section 6.6.1 Dose Delays	Additional guidance provided: if dosing must be delayed for reasons other than treatment-related toxicity, dosing will resume as soon as feasible.	Guidance on delays not due to toxicity.
Section 6.7 Continued Access to Study Intervention After the End of the Study	Updated with further information regarding post-trial access to study intervention.	Updated following change to protocol template.
Section 7.1 Discontinuation of Study Interventions	Additional instructions added. Any subject seeking to enrol in any form of trial while on study should consult and obtain approval from AZ prior.	Update and clarification.
Section 8.2.3 Vital Signs	Added clarification that for neoadjuvant Cycle 1, where pre, 30 min post, and end of infusion vital signs are required, this applies to the 1st administered IMP only and does not include chemotherapy or durvalumab.	Clarification.
Section 8.2.4 Electrocardiograms	Clarification of discordance in QTcF interval cutoff between Section 8.2.4 and exclusion criterion #12. Aligned to QTcF interval ≥ 470 ms.	Clarification
Section 8.2.8 Ophthalmological Assessments	Addition of referencing to the Dato-DXd Ophthalmologic Assessment Manual for further details.	Clarification.
Section 8.2.9 Other Safety Assessments	Further instruction for patients with new or worsening pulmonary symptoms or radiological abnormality suggestive of pneumonitis/ILD added: troponin measurements will be done to rule out cardiac aetiology.<br>Further information for pneumonitis (ILD) investigation added: High Resolution CT if feasible, otherwise non-contrast chest CT is also acceptable.	Update and clarification.
Section 8.3.14 and Appendix B4 Medication Error, Drug Abuse, and Drug Misuse	Further updates of the definition of medication error and new subsections added to provide additional information on drug abuse and drug misuse.	Updated following change to protocol template.
Section 9.2 Sample Size Determination	Text updated to consider the new treatment Arms 3B and 3C to enrol 35 patients per arm to evaluate tolerability of alternate dose regimens, with a potential to increase to 70 patients per arm.	Update.
Section 9.3 Populations for Analysis	The analysis set "Interim pathological response evaluable" was removed.	This originally defined interim pathological response evaluable analysis set is not of interest in the interim analysis.

Section Number and Name	Description of Change	Brief Rationale
Section 9.4.2.2 Secondary Endpoints	Surgery-related statistical analysis updated: the rate and outcomes of surgical complications will also be summarised.	Update and clarification.
Section 9.7 ILD Adjudication Committee and Appendix N 7	Section added. An independent ILD Adjudication Committee is responsible for reviewing all cases of potential ILD/pneumonitis on arm 4 as part of the Dato-DXd programme requirements.	Update.
Appendix A1 Regulatory and Ethical Considerations	Update of AstraZeneca standard text.<br>New subsection added on "Regulatory Reporting Requirements for Serious Breaches".	Updated following change to protocol template.
Appendix A6 Dissemination of Clinical Study Data	Website address for study disclosure updated following latest Transcelerate CSP update (version 9).	Update.
Appendix A7 Data Quality Assurance	Bullet point added to confirm that AstraZeneca or designee is responsible for medical oversight throughout the conduct of the study.	Updated following change to protocol template.
Appendix B4 Medication Error, Drug Abuse and Drug Misuse	New mandatory subsection on drug abuse and drug misuse added.	Updated following change to protocol template.
Appendix G Contraception Requirements<br>Appendix G1 Female Patients<br><br>Appendix G2 Male Patients with a Female Partner of Childbearing Potential	 <br>Additional guidance provided. Preservation of ova should be considered prior to enrolment in this study.<br><br>Additional guidance provided. Total sexual abstinence is an acceptable method provided it is the usual lifestyle of the patient.	Update.
Appendix H1 Prohibited and Permitted Concomitant Medications/Therapies	Addition to Table 16 Prohibited medications/therapies: Use of intranasal, inhaled, topical steroids, or local steroid injections (eg, intra articular injection) is permitted.	Update and clarification.
Appendix M Arm 3A, 3B, and 3C: Volrustomig + Platinum Doublet Chemotherapy (Neoadjuvant Treatment) Followed by Volrustomig Alone (Adjuvant Treatment)	This appendix provides information for patients randomised to Arm 3A, 3B, and 3C.	Update. Changes made following the addition of new treatment arms (Arm 3B and 3C).

Section Number and Name	Description of Change	Brief Rationale
Appendix M1 Schedule of Activities	Update of Table M22 Schedule of Activities for Neoadjuvant Treatment for Patients Randomised to Arm 3A, 3B, or 3C. Additional time points added for flow cytometry and PBMC.	Update.
Appendix M4 Rationale for Combining with Chemotherapy	Information from results of recent studies with combination therapy added.	Update.
Appendix M5 Rationale for Additional Volrustomig Treatment Arms 3B and 3C.	Rationale added for additional volrustomig treatment arms 3B and 3C.	Updates following the introduction of new treatment arms using volrustomig.
Appendix M6 Justification for Dose for Study Interventions Used in Arms 3A, 3B and 3C.<br>(Appendix M6.1, Appendix M6.2 and Appendix M6.3)	Justification added for doses used in treatment arms 3A, 3B and 3C .	Updates following the introduction of new treatment arms using volrustomig.
Appendix M7.3.3 Volrustomig Intrasubject Dose Reduction (Arm 3A only)	New section with guidelines for dose reductions added, applicable to Arm 3A only.	Update.
Appendix N1 Schedule of Activities	Additional instructions added in footnotes for Table N25 Schedule of Activities for Neoadjuvant Treatment for Patients Randomised to Arm 4:<br>Footnote c: recommendation of professional dental evaluation before study intervention initiation and dental treatment if indicated. Patients will be provided an oral care plan prior to and during study treatment.<br>Footnote d: Ophthalmologic assessments including visual acuity testing, slit lamp examination, intraocular pressure measurement, fundoscopy, and fluorescein staining will be performed at screening by an ophthalmologist, or if unavailable, another licensed eye care provider.	Update and clarification.
Appendix N6.1 Treatment Regimen	Minor updates. Patients should remain at the site for at least 1 hour post infusion of every dose of Dato-DXd for close observation for possible allergic reaction **and IRR**. Participants will be provided an oral care plan prior to and during study treatment.	Update and clarification.

Section Number and Name	Description of Change	Brief Rationale
Appendix N7 Adverse Events of Special Interest Associated with Dato-DXd	Updates for ILD/Pneumonitis. If the AE is suspected to be ILD/pneumonitis, treatment with Dato-DXd is to be delayed pending further evaluations as **described in the Dato-DXd TMGs.** Added information on the independent ILD Adjudication Committee responsible for reviewing all cases of potential ILD/pneumonitis.<br>Updates for infusion related reaction. Infusion-related IRR is an identified risk associated with Dato-DXd treatment (no longer classified as important identified risk).	Updates and clarification.

Abbreviations: CD: cluster of differentiation; CSP: clinical study protocol; CSR: clinical study report; CTX: double platinum chemotherapy; CT: computed tomography; Dato-DXd: datopotamab deruxtecan; EOT: end of treatment; HIV: human immunodeficiency virus; HBV: hepatitis B virus; HCV: hepatitis C virus; ILD: interstitial lung disease; IMP: investigational medicinal product; INN: International Nonproprietary Name; IRB: Institutional Review Board; IRR: infusion-related reaction; LVEF: left ventricular ejection fraction; MUGA: multigated acquisition scans; NSCLC: non-small cell lung cancer; PMBC: Peripheral blood mononuclear cells; QTcF: QT interval corrected by Fridericia's formula; Q3W: every 3 weeks; RNA: ribonucleic acid; TEAE: treatment-emergent adverse event; TMG: toxicity management guideline.

Amendment 5 (06 Mar 2024)

Overall Rationale for Amendment

This protocol was amended to include the monalizumab liquid drug product presentation. The updates are summarised below. Other minor editorial updates were made throughout. Administrative changes, such as formatting, updates to abbreviations, and punctuation corrections are not presented in this summary.

List of Substantial Modifications

Section Number and Name	Description of Change	Brief Rationale
Section 6.1.1 Investigational Products, Table 5, Appendix L 6.3 Investigational Product Preparation and Administration	Addition of liquid product presentation of monalizumab (750 mg/vial, 50 mg/mL).	A second presentation of monalizumab is being added to the study. Sites may receive either the lyophilised product or the liquid product. Over time, sites will switch completely to the liquid product as the manufacture of the lyophilised product will be discontinued.

List of Non-substantial Modifications

Section Number and Name	Description of Change	Brief Rationale
Section 6.1.1 Investigational Products, Table 5	Footnote h added to clarify the monalizumab drug products that are available.	Clarification.
Section 9.5 Interim Analyses	The text 'Analyses will be based on the interim analysis set as defined in Section 9.3' was replaced with 'Analyses will be based on several interim analysis sets, which are defined in the SAP'.	Clarification to align with previous CSP amendment update to analysis sets.
Appendix Q Protocol Amendment History, Amendment 4, Overall Rationale for Amendment	Text states 'This protocol was amended to incorporate 2 additional study arms (3A and 3B)'. This is a typographical error and was updated to say '(3B and 3C)'.	Correction of typographical error.

Amendment 6 (22 May 2024)

Overall Rationale for Amendment

This protocol was amended following a review of [redacted] safety data from the volrustomig clinical programme. As a result of this review, AstraZeneca is implementing additional preventative safety measures including changes to the [redacted] monitoring schedule and toxicity management guidelines.

List of Substantial Modifications

Section Number and Name	Description of Change	Brief Rationale
8.2.1 Clinical Safety Laboratory Assessments	Addition of text to specify that clinical laboratory safety test results must be promptly reviewed by the Investigator.	Implementation of additional preventative safety measures including changes to the [redacted] monitoring schedule and toxicity management guidelines, following review of [redacted] safety data from the volrustomig clinical programme.
8.2.1 Clinical Safety Laboratory Assessments, Table 7; Appendix M 1 Schedule of Activities, Table M22 and Table M23	Table footnotes amended to clarify when [redacted]	
Appendix M 1 Schedule of Activities, Table M22	[redacted] replaced with a footnote to clarify when each clinical chemistry parameter will be measured.	
Appendix M9 [redacted] Safety Monitoring Strategy for Participants Assigned to Volrustomig	Appendix added to clarify the [redacted] monitoring measures in place for patients receiving volrustomig.	

11 REFERENCES

Abou-Alfa et al, 2022
Abou-Alfa GK, Lau G, Kudo M, Chan SL, Kelly RK, Furuse J, et al. Tremelimumab plus Durvalumab in unresectable hepatocellular carcinoma. NEJM Evid. 2022;1(8).

AEGEAN press release 2022
AstraZeneca. Imfinzi plus chemotherapy significantly improved pathologic complete response in AEGEAN Phase III trial in resectable non-small cell lung cancer. Available at https://www.astrazeneca.com/media-centre/press-releases/2022/imfinzi-improved-pcr-in-resectable-lung-cancer.html. Accessed 04 August 2022.

AJCC Cancer Staging Manual, 8th Edition
Amin MB, Edge S, Greene F, Byrd DR, Brookland RK, Washington MK, et al, editors. AJCC Cancer Staging Manual, Eighth Edition. American Joint Committee on Cancer (AJCC); 2017. (Available on request).

American Cancer Society, 2018
American Cancer Society. Cancer Facts & Figures 2018. Atlanta: American Cancer Society, 2018. Available at: https://www.cancer.org/research/cancer-facts-statistics/all-cancer-factsfigures/cancer-facts-figures-2018.html

Antonia et al, 2016
Antonia S, Goldberg SB, Balmanoukian A, Chaft JE, Sanborn RE, Gupta A et al. Safety and antitumour activity of durvalumab plus tremelimumab in non-small cell lung cancer: a multicentre, phase 1b study. Lancet Oncol. 2016;17(3):299-308.

Ascierto et al, 2017
Ascierto PA, Vecchio M, Robert C, Mackiewicz A, Chiarion-Sileni V, Arance, et al. Ipilimumab 10 mg/kg versus ipilimumab 3 mg/kg in patients with unresectable or metastatic melanoma: a randomised, double-blind, multicentre, phase 3 trial. Lancet Oncol. 2017, 18(5):611-62.

Awad et al, 2023
Awad MM, Forde PM, Girard N, Spicer JD, Wang C, S. Lu S, et al. Neoadjuvant nivolumab (N) + ipilimumab (I) vs chemotherapy (C) in the phase III CheckMate 816 trial. Annals of Oncology. 2023;34(2):731.
Available at: https://doi.org/10.1016/j.annonc.2023.09.739.

Bai et al, 2020
Bai R, Li L, Chen X, Chen N, Song W, Cui J. Neoadjuvant and Adjuvant Immunotherapy: Opening New Horizons for Patients With Early-Stage Non-small Cell Lung Cancer. Front Oncol. 2020;10:575472.

Borazanci et al, 2022
Borazanci E, Schram A M, Garralda E, Brana I, Vieito Villar M, Spreafico A et al. Phase I, first-in-human study of MSC-1 (AZD0171), a humanized anti-leukemia inhibitory factor monoclonal antibody, for advanced solid tumors. ESMO open. 2022;7(4):100530.

Bottino et al 2003
Bottino C, Castriconi R, Pende D, Rivera P, Nanni M, Carnemolla B, et al. Identification of PVR (CD155) and Nectin-2 (CD112) as cell surface ligands for the human DNAM-1 (CD226) activating molecule. J Exp Med. 2003;198(4):557-67.

Bradbury et al, 2017
BradburyP, Sivajohanathan S, Chan A, Kulkarni S, Ung Y, Ellis PM. Postoperative Adjuvant Systemic Therapy in Completely Resected Non-Small-Cell Lung Cancer: A Systematic Review. Clin Lung Cancer. 2017;18(3):259-273.e8

Braud et al, 1998
Braud VM, Allan DS, O'Callaghan CA, Soderstrom K, D'Andrea A, Ogg GS et al. HLA-E binds to natural killer cell receptors CD94/NKG2A, B and C. Nature. 1998;391(6669):795-9.

Burdett et al, 2006
Burdett S, Stewart LA, Rydzewska L. A systematic review and meta-analysis of the literature: chemotherapy and surgery versus surgery alone in non-small cell lung cancer. J Thorac Oncol. 2006;1(7):611-21.

Burdett et al, 2015
Burdett S, Pignon JP, Tierney J, Tribodet H, Stewart L, Le Pechoux C et al. Adjuvant chemotherapy for resected early-stage non-small cell lung cancer. Cochrane Database Syst Rev. 2015;(3):CD011430.

Cascone et al, 2018
Cascone T, Gold KA, Swisher SG, Liu DD, Fossella FV, Sepesi B, et al. Induction Cisplatin Docetaxel Followed by Surgery and Erlotinib in Non-Small Cell Lung Cancer. Ann Thorac Surg. 2018;105(2):418-24.

Cascone et al, 2022
Cascone T, García-Campelo R, Spicer J, Weder W, Daniel D, Spigel D, et al. NeoCOAST: open-label, randomized, Phase 2, multidrug platform study of neoadjuvant durvalumab alone or combined with novel agents in patients (pts) with resectable, early-stage non-small-cell lung cancer (NSCLC). Presented at: AACR Annual Meeting; April 8-13, 2022.

Cascone et al, 2023
Cascone T, Leung CH, Weissferdt A, Pataer A, Carter BW, Godoy MCB, et al. Neoadjuvant chemotherapy plus nivolumab with or without ipilimumab in operable non-small cell lung cancer: the phase 2 platform NEOSTAR trial. Nat Med 2023;29(3):593-604.

Available at: https://doi.org/10.1038/s41591-022-02189-0.

Cottrell et al, 2018
Cottrell TR, Thompson ED, Forde PM, Stein JE, Duffield AS, Anagnostou V, et al. Pathologic features of response to neoadjuvant anti-PD-1 in resected non-small-cell lung carcinoma: a proposal for quantitative immune-related pathologic response criteria (irPRC). Ann Oncol 2018;29(8):1853-1860.

Chaft et al, 2013
Chaft JE, Rusch V, Ginsberg MS, Paik PK, Finley DJ, Kris MG, et al. Phase II trial of neoadjuvant bevacizumab plus chemotherapy and adjuvant bevacizumab in patients with resectable non-squamous nonsmall cell lung cancers. J Thorac Oncol 2013;8(8):1084-90.

Chai et al 2020
Chai LF, Prince E, Pillarisetty VG, Katz SC. Challenges in assessing solid tumor responses to immunotherapy. Cancer Gene Ther. 2020;27(7-8):528-38.

Chansky et al, 2017
Chansky K, Detterbeck FC, Nicholson AG, Rusch VW, Vallieres E, Groome P, et al. The IASLC Lung Cancer Staging Project: External Validation of the Revision of the TNM Stage Groupings in the Eighth Edition of the TNM Classification of Lung Cancer. J Thorac Oncol. 2017;12(7):1109-21.

Chauvin and Zarour 2020
Chauvin J-M and Zarour HM. TIGIT in cancer immunotherapy. J Immunother Cancer. 2020;8(2):e000957.

Chen and Mellman, 2013
Chen DS, Mellman I. Oncology meets immunology: the cancer-immunity cycle. Immunity. 2013;39(1):1-10.

Cubas et al, 2009
Cubas R, Li M, Chen C, Yao Q. Trop2: a possible therapeutic target for late stage epithelial carcinomas. Biochim Biophys Acta. 2009;1796(2):309-14.

Curran et al, 2010
Curran MA, Montalvo W, Yagita H, Allison JP. PD-1 and CTLA-4 combination blockade expands infiltrating T cells and reduces regulatory T and myeloid cells within B16 melanoma tumors. Proc Natl Acad Sci USA. 2010;107(9):4275-80.

De Leyn et al, 2014
De Leyn P, Dooms C, Kuzdzal J, Lardinois D, Passlick B, Rami-Porta R, et al. Revised ESTS guidelines for preoperative mediastinal lymph node staging for non-small-cell lung cancer. Eur J Cardiothorac Surg 2014;45(5):787-98.

Dovedi et al 2021
Dovedi SJ, Elder MJ, Yang C, Sitnikova SI, Irving L, Hansen A, et al. Design and efficacy of a monovalent bispecific PD-1/CTLA4 antibody that enhances CTLA4 blockade on PD-1+ activated T Cells. Cancer Discov. 2021;11(5):1100-17.

EMA 2017
European Medicines Agency. Guideline on the evaluation of anticancer medicinal products in man. London, 2017. Doc Ref. EMA/CHMP/205/95/Rev.5. Available from UR: https://www.ema.europa.eu/en/documents/scientific-guideline/guideline-evaluation-anticancer-medicinal-products-man-revision-5_en.pdf. (Accessed on 21 May 2021)

Fahrenbruch et al, 2018
Fahrenbruch R, Kintzel P, Bott AM, Gilmore S, Markham R. Dose Rounding of Biologic and Cytotoxic Anticancer Agents: A Position Statement of the Hematology/Oncology Pharmacy Association. J Oncol Pract. 2018;14(3):e130-e136.

FDA 2007
Food and Drug Administration. Guidance for Industry, Clinical Trial Endpoints for the Approval of Cancer Drugs and Biologics. Available from URL: https://www.fda.gov/media/71195/download. (Accessed on 21 May 2021).

Felip et al, 2010
Felip E, Rosell R, Maestre JA, Rodriguez-Paniagua JM, Moran T, Astudillo J, et al. Preoperative chemotherapy plus surgery versus surgery plus adjuvant chemotherapy versus surgery alone in early-stage non-small-cell lung cancer. J Clin Oncol. 2010;28(19):3138-45.

Felip et al, 2021
Felip E, Altorki N, Zhou C, Csőszi T, Vynnychenko I, Goloborodko O, et al. Adjuvant atezolizumab after adjuvant chemotherapy in resected stage IB–IIIA non-small-cell lung cancer (IMpower010): a randomised, multicentre, open-label, phase 3 trial. The Lancet. 2021;398(10308):1344-57.

Fife and Bluestone, 2008
Fife BT, Bluestone JA. Control of peripheral T-cell tolerance and autoimmunity via the CTLA-4 and PD-1 pathways. Immunol Rev. 2008;224:166-82.

Forde et al, 2021
Forde PM, Spicer J, Lu S, Provencio M, Mitsudomi T, Awad MM, et al. Nivolumab (NIVO) + platinum-doublet chemotherapy (chemo) vs chemo as neoadjuvant treatment (tx) for resectable (IB-IIIA) non-small cell lung cancer (NSCLC) in the phase 3 CheckMate 816 trial. Presented at: AACR Annual Meeting; April 10-15, 2021

Forde et al, 2022
Forde PM, Spicer J, Lu S, Provencio M, Mitsudomi T, Awad MM, et al. Neoadjuvant

Nivolumab plus Chemotherapy in Resectable Lung Cancer. N Engl J Med. 2022;386(21):1973-85.

Freeman et al 2000
Freeman GJ, Long AJ, Iwai Y, Bourque K, Chernova T, Nishimura H, et al. Engagement of the PD-1 immunoinhibitory receptor by a novel B7 family member leads to negative regulation of lymphocyte activation. J Exp Med. 2000;192(7):1027-34.

Frewer et al, 2016
Frewer P, Mitchell P, Watkins C, and Matcham J. Decision-making in early clinical drug development. Pharma Statist. 2016;15(3):255-263.

Gandhi et al, 2018
Gandhi L, Rodríguez-Abreu D, Gadgeel S, Esteban E, Felip E, De Angelis F, et al. Pembrolizumab plus chemotherapy in metastatic non-small-cell lung cancer. N Engl J Med. 2018;378(22):2078-2092.

Garon et al, 2021
Garon E, Johnson M, Lisberg A, Spira A, Yamamoto N, Heist R, et al. TROPION-PanTumor01: Updated Results From the NSCLC Cohort of the Phase 1 Study of Datopotamab Deruxtecan in Solid Tumors. J Thorac Oncol. 2021;16(10):S892-3.

Goldenberg et al, 2015
Goldenberg DM, Cardillo TM, Govindan SV, Rossi EA, Sharkey RM. Trop-2 is a novel target for solid cancer therapy with sacituzumab govitecan (IMMU-132), an antibody-drug conjugate (ADC). Oncotarget. 2015;6(26):22496-512.

Goldenberg et al, 2018
Goldenberg DM, Stein R, Sharkey RM. The emergence of trophoblast cell-surface antigen 2 (TROP-2) as a novel cancer target. Oncotarget. 2018;9(48):28989-9006.

Goldstraw et al, 2007
Goldstraw P, Crowley J, Chansky K, Giroux DJ, Groome PA, Rami-Porta R, et al. The IASLC Lung Cancer Staging Project: proposals for the revision of the TNM stage groupings in the forthcoming (seventh) edition of the TNM Classification of malignant tumours. J Thorac Oncol. 2007;2(8):706-14

Geoghegan et al, 2016
Geoghegan JC, Diedrich G, Lu X, Rosenthal K, Sachsenmeier KF, Wu H, et al. Inhibition of CD73 AMP hydrolysis by a therapeutic antibody with a dual, non-competitive mechanism of action. MAbs. 2016;8(3):454-67.

Hay et al, 2016
Hay CM, Sult E, Huang Q, Mulgrew K, Fuhrmann SR, McGlinchey KA, et al. Targeting

CD73 in the tumor microenvironment with MEDI9447. Oncoimmunology. 2016;5(8):e1208875.

Hellmann et al, 2014
Hellmann MD, Chaft JE, William WN Jr, Rusch V, Pisters KM, Kalhor N, et al. Pathological response after neoadjuvant chemotherapy in resectable non-small-cell lung cancers: proposal for the use of major pathological response as a surrogate endpoint. Lancet Oncol 2014;15(1):e42-50.

Hellmann et al, 2019
Hellmann MD, Paz-Ares L, Bernabe Caro R, Zurawski B, Kim SW, Carcereny Costa E, et al. Nivolumab plus Ipilimumab in Advanced Non-Small-Cell Lung Cancer. N Engl J Med. 2019;381(21):2020-31.

Heymach et al, 2023
Heymach JV, Harpole D, Mitsudomi T, Taube JM, Galffy G, Hochmair M, et al (AEGEAN Investigators). Perioperative Durvalumab for Resectable Non-Small-Cell Lung Cancer. N Engl J Med 2023;389(18):1672-1684
https://doi.org/10.1056/NEJMoa2304875

Hiltermann et al 2024
Hiltermann J, Izumi H, Cho BC, Cunha S, Danchaivijitr P, Felip E, et al. Efficacy and safety of rilvegostomig, an anti-PD-1/TIGIT bispecific, for CPI-naïve metastatic NSCLC with PD-L1 1-49% or ≥50%. Abstract #OA11.03. Presented at: IASLC WCLC; 09 September 2024.

Hodi, 2010
Hodi FS. Overcoming immunological tolerance to melanoma: Targeting CTLA-4. Asia Pac J Clin Oncol. 2010;6 Suppl 1:S16-23.

Hui et al 2017
Hui E, Cheung J, Zhu J, Su X, Taylor MJ, Wallweber HA, et al. T cell costimulatory receptor CD28 is a primary target for PD-1–mediated inhibition. Science. 2017;355(6332):1428-33.

IASLC Staging Manual in Thoracic Oncology 2016
Rami-Porta R, ed. Staging manual in thoracic oncology (2nd ed; includes 8th ed): tumor, node, and metastasis classification (TNM) information for lung cancer, plural mesothelioma, thymic malignancies, and carcinoma of the oesophagus and of oesophagogastric junction). IASLC 2016. (Available on request).

IMFINZI® US Package Insert [PI], 2018
IMFINZI Package Insert. Wilmington, DE: AstraZeneca Pharmaceuticals LP; February 2018.

Inoue et al, 2017
Inoue Y, Yoshimura K, Kurabe N, Kahyo T, Kawase A, Tanahashi M, et al. Prognostic impact of CD73 and A2A adenosine receptor expression in non-small-cell lung cancer. Oncotarget. 2017;8(5):8738-51.

Johnson et al, 2021
Johnson ML, Cho BC, Luft A, Alatorre-Alexander J, Geater SL, Laktionov K, et al. PL02.01. Durvalumab ± Tremelimumab + Chemotherapy as First-Line Treatment for mNSCLC: Results from the Phase 3 POSEIDON Study. J Thorac Oncol. 2021;16:S844.

Juneja et al, 2017
Juneja VR, McGuire KA, Manguso RT, LaFleur MW, Collins N, Haining WN, et al. PD-L1 on tumor cells is sufficient for immune evasion in immunogenic tumors and inhibits CD8 T cell cytotoxicity. J Exp Med. 2017;214(4):895-904.

Keir et al, 2008
Keir ME, Butte MJ, Freeman GJ, Sharpe AH. PD-1 and its ligands in tolerance and immunity. Annu Rev Immunol. 2008;26:677-704.

Kelley et al 2021
Kelley RK, Sangro B, Harris W, Ikeda M, Okusaka T, Kang YK, et al. Safety, efficacy, and pharmacodynamics of tremelimumab plus durvalumab for patients with unresectable hepatocellular carcinoma: randomized expansion of a phase I/II study. J Clin Oncol. 2021;39(27):2991-3001.

Knight et al, 1999
Knight DA, Lydell CP, Zhou D, Weir TD, Robert Schellenberg R, Bai TR. Leukemia inhibitory factor (LIF) and LIF receptor in human lung. Distribution and regulation of LIF release. Am J Respir Cell Mol Biol. 1999;20(4):834-41.

Latchman et al 2001
Latchman Y, Wood CR, Chernova T, Chaudhary D, Borde M, Chernova I, et al. PD-L2 is a second ligand for PD-1 and inhibits T cell activation. Nat Immunol. 2001;2(3):261-8.

Lee et al 2022
Lee K, Malhotra D, Pryts S, Clancy-Thompson E, Omar B, Naiman B, et al. 469 Preclinical studies support clinical development of AZD2936, a monovalent bispecific humanized antibody targeting PD-1 and TIGIT. J Immunother Cancer. 2022;10(Suppl 2):A489.

Levin et al 2011
Levin SD, Taft DW, Brandt CS, Bucher C, Howard ED, Chadwick EM, et al. Vstm3 is a member of the CD28 family and an important modulator of T-cell function. Eur J Immunol. 2011;41(4):902-15.

Levy et al, 2022
Levy B, Paz-Ares L, Rixie O, Su W-C, Yang T-Y, Tolcher A, et al. TROPION-Lung02: Initial Results for Datopotamab Deruxtecan Plus Pembrolizumab and Platinum Chemotherapy in Advanced NSCLC. J Thorac Oncol. 2022;17(9):S91.

Li et al 2021
Li TR, Chatterjee M, Lala M, Abraham AK, Freshwater T, Jain L, et al. Pivotal dose of pembrolizumab: a dose-finding strategy for immuno-oncology. Clin Pharmacol Ther. 2021 Jul;110(1):200-209.

Liu et al, 2016
Liu J, Blake SJ, Yong MC, Harjunpaa H, Ngiow SF, Takeda K, et al. Improved Efficacy of Neoadjuvant Compared to Adjuvant Immunotherapy to Eradicate Metastatic Disease. Cancer Discov. 2016;6(12):1382-99

Loriot et al, 2021
Loriot Y, Marabelle A, Guégan JP, Danlos FX, Besse B, Chaput N, et al. Plasma proteomics identifies leukemia inhibitory factor (LIF) as a novel predictive biomarker of immune-checkpoint blockade resistance. Ann Oncol. 2021;32(11):1381-90.

Machtay et al, 2004
Machtay M, Lee JH, Stevenson JP, Shrager JB, Algazy KM, Treat J, et al. Two commonly used neoadjuvant chemoradiotherapy regimens for locally advanced stage III non-small cell lung carcinoma: long-term results and associations with pathologic response. J Thorac Cardiovasc Surg. 2004;127(1):108-13.

Mangsbo et al, 2010
Mangsbo SM, Sandin LC, Anger K, Korman AJ, Loskog A, Tötterman TH. Enhanced tumor eradication by combining CTLA-4 or PD-1 blockade with CpG therapy. J Immunother. 2010;33(3):225-35.

Manieri et al 2017
Manieri NA, Chiang EY, Grogan JL. TIGIT: A key inhibitor of the cancer immunity cycle. Trends Immunol. 2017;38(1):20-8.

Mauguen et al, 2013
Maugen Al, Pignon JP, Burdett S, Domerg C, Fisher D, Paulus R, et al. Surrogate endpoints for overall survival in chemotherapy and radiotherapy trials in operable and locally advanced lung cancer: a re-analysis of meta-analyses of individual patients' data. Lancet Oncol 2013;14(7):619-26.

Mok et al 2019
Mok TSK, Wu Y-L, Kudaba I, Kowalski DM, Cho BC, Turna HZ, et al. Pembrolizumab versus chemotherapy for previously untreated, PD-L1-expressing, locally advanced or

metastatic non-small-cell lung cancer (KEYNOTE-042): a randomised, open-label, controlled, phase 3 trial. Lancet. 2019;393(10183):1819-30.

National Comprehensive Cancer Network, 2020
National Comprehensive Cancer Network. Carboplatin Dosing in Adults, Appendix B. NCCN Templates, 2020. Available:
https://ctep.cancer.gov/content/docs/carboplatin_information_letter.pdf. Accessed on 05-August-2022.

National Comprehensive Cancer Network, 2022
National Comprehensive Cancer Network. Non-small Cell Lung Cancer: NCCN Guidelines, 2022.

National Comprehensive Cancer Network, 2023
NCCN. Clinical Practice Guidelines in Oncology: Non-small Cell Lung Cancer. Version 5.2023. Accessed on 08 November 2023.

Nicola and Babon, 2015
Nicola NA and Babon JJ. Leukemia inhibitory factor (LIF). Cytokine Growth Factor Rev. 2015;26(5):533-44.

Oh et al, 2017
Oh DY, Cham J, Zhang L, Fong G, Kwek SS, Klinger M, et al. Immune Toxicities Elicted by CTLA-4 Blockade in Cancer Patients Are Associated with Early Diversification of the T-cell Repertoire. Cancer Res. 2017;77(6):1322-30.

Ohaegbulam et al, 2015
Ohaegbulam KC, Assal A, Lazar-Molnar E, Yao Y, Zang X. Human cancer immunotherapy with antibodies to the PD-1 and PD-L1 pathway. Trends Mol Med. 2015;21(1):24-33.

Okazaki et al, 2013
Okazaki T, Chikuma S, Iwai Y, Fagarasan S, Honjo T. A rheostat for immune responses: the unique properties of PD-1 and their advantages for clinical application. Nat Immunol. 2013;14(12):1212-8.

Padrón et al, 2022
Padrón LJ, Maurer DM, O'Hara MH, O'Reilly EM, Wolff RA, Wainberg ZA, et al. Sotigalimab and/or nivolumab with chemotherapy in first-line metastatic pancreatic cancer: clinical and immunologic analyses from the randomized phase 2 PRINCE trial. Nat Med. 2022; 28(6):1167–77.

Pardoll, 2012
Pardoll DM. The blockade of immune checkpoints in cancer immunotherapy. Nat Rev Cancer. 2012;12(4):252-64.

Pataer et al, 2012
Pataer A, Kalhor N, Correa AM, Raso MG, Erasmus JJ, Kim ES, et al. Histopathologic response criteria predict survival of patients with resected lung cancer after neoadjuvant chemotherapy. J Thorac Oncol. 2012;7(5):825-32.

Paz-Ares et al, 2018
Paz-Ares L, Luft A, Tafreshi A, Gumus M, Mazières J, Hermes B, et al. KEYNOTE-407: Phase 3 study of carboplatin-paclitaxel/nab-paclitaxel with or without pembrolizumab for metastatic squamous NSCLC. Poster presented at: ASCO Annual Meeting 2018.

Paz-Ares et al, 2021
Paz-Ares L, Ciuleanu TE, Cobo M, Schenker M, Zurawski B, Menezes J, et al. First-line nivolumab plus ipilimumab combined with two cycles of chemotherapy in patients with non-small-cell lung cancer (CheckMate 9LA): an international, randomised, open-label, phase 3 trial. Lancet Oncol. 2021;22(2):198-211.

Paz-Ares et al, 2022
Paz-Ares L, O'Brien M, Mauer M, Dafni U, Oselin K, Havel L, et al. VP3-2022: Pembrolizumab (pembro) versus placebo for early-stage non-small cell lung cancer (NSCLC) following complete resection and adjuvant chemotherapy (chemo) when indicated: Randomized, triple-blind, phase III EORTC-1416-LCG/ETOP 8-15 – PEARLS/KEYNOTE-091 study. Annals of Oncology. 2022;33(4):451-3.

Pentheroudakis et al, 2020
Pentheroudakis G; ESMO Guidelines Committee. Recent eUpdate to the ESMO Clinical Practice Guidelines on early and locally advanced non-small-cell lung cancer (NSCLC). Ann Oncol. 2020;31(9):1265-1266.

Postmus et al, 2017
Postmus PE, Kerr KM, Oudkerk M, Senan S, Waller DA, Vansteenkiste J, et al. Early and locally advanced non-small-cell lung cancer (NSCLC): ESMO Clinical Practice Guidelines for diagnosis, treatment and follow-up. Ann Oncol 2017;28(suppl_4):iv1-iv21.

Provencio et al, 2020
Provencio M, Nadal E, Insa A, García-Campelo MR, Casal-Rubio J, Dómine M, et al. Neoadjuvant chemotherapy and nivolumab in resectable non-small-cell lung cancer (NADIM): an open-label, multicentre, single-arm, phase 2 trial. Lancet Oncol 2020:21(11):1413-1422.

Provencio et al, 2022
Provencio M, Serna-Blasco R, Nadal E, Insa A, García-Campelo MR, Casal-Rubio J, Dómine M, et al. Overall survival and biomarker analysis of neoadjuvant nivolumab plus chemotherapy in operable stage IIIA non-small-cell lung cancer (NADIM phase II trial).

Journal of clinical oncology: official journal of the American Society of Clinical Oncology, JCO2102660. 2022; doi:10.1200/JCO.21.02660.

Provencio-Pulla M et al, 2022
Procencio-Pulla M, Nadal E, Gozalez-Larriba JL, Martinez-Marti A, Bernabe R, Bosch-Barrera J, et al. Nivolumab + chemotherapy versus chemotherapy as neoadjuvant treatment for resectable stage IIIA NSCLC: primary endpoint results of pathological complete response (PCR) from Phase II NADIM II trial. Journal of clinical oncology. 2022;40(16):8501-8501. https://doi.org/10.1200/jco.2022.40.16_suppl.8501.

Reck et al 2016
Reck M, Rodríguez-Abreu D, Robinson AG, Hui R, Csőszi T, Fülöp A, et al. Pembrolizumab versus chemotherapy for PD-L1–positive non–small-cell lung cancer. N Engl J Med. 2016;375(19):1823-33.

Ricciardi et al, 2014
Ricciardi GR, Russo A, Franchina T, Ferraro G, Zanghì M, Picone A, et al. NSCLC and HER2: between lights and shadows. J Thorac Oncol. 2014;9(12):1750-62.

Sampson et al, 2006
Sampson HA, Muñoz-Furlong A, Campbell RL, Adkinson FN Jr, Bock SA, Branum A, et al. Second symposium on the definition and management of anaphylaxis: Summary report-Second National Institute of Allergy and Infectious Disease/Food Allergy and Anaphylaxis Network symposium. J Allergy Clin Immunol. 2006;117:391-7.

Shu et al, 2020
Shu CA, Gainor JF, Awad MM, Chiuzan C, Grigg C, Pabani A, et al. Neoadjuvant atezolizumab and chemotherapy in patients with resectable non-small-cell lung cancer: an open-label, multicentre, single-arm, phase 2 trial. Lancet Oncol 2020;21:786–95.

Shulgin et al, 2020
Shulgin B, Kosinsky Y, Omelchenko A, Chu L, Mugundu G, Aksenov S, et al. Dose dependence of treatment-related adverse events for immune checkpoint inhibitor therapies: a model-based meta-analysis. Oncoimmunology 2020;9(1):1748982 DOI:10.1080/2162402X.2020.1748982

Socinski et al, 2018
Socinski MA, Jotte RM, Cappuzzo F, Orlandi F, Stroyakovskiy D, Nogami N, et al. Atezolizumab for first-line treatment of metastatic nonsquamous NSCLC. N Engl J Med. 2018;378(24):2288-301.

Spicer et al, 2023a
Spicer J, Forde PM, Provencio M, Lu S, Wang C, Mitsudomi T et al. Clinical outcomes with

neoadjuvant nivolumab (N) + chemotherapy (C) vs C by definitive surgery in patients (pts) with resectable NSCLC: 3-y results from the phase 3 CheckMate 816 trial. Journal of Clinical Oncology 2023; 41(16_suppl): 8521
Available at: https://ascopubs.org/doi/10.1200/JCO.2023.41.16_suppl.8521

Spicer et al, 2023b
Spicer, JD, Gao S, Liberman M, Kato T, Tsuboi M, Lee SH, et al. LBA56 Overall survival in the KEYNOTE-671 study of perioperative pembrolizumab for early-stage non-small-cell lung cancer (NSCLC). NSCLC, Early Stage 2023; 34 (2):1297-1298.
DOI:https://doi.org/10.1016/j.annonc.2023.10.052

Stanietsky et al 2009
Stanietsky N, Simic H, Arapovic J, Toporik A, Levy O, Novik A, et al. The interaction of TIGIT with PVR and PVRL2 inhibits human NK cell cytotoxicity. Proc Natl Acad Sci USA. 2009;106(42):17858-63.

Tempero et al, 2021
Tempero M, Oh DY, Tabernero J, Reni M, Van Cutsem E, Hendifar A, et al. Ibrutinib in combination with nab-paclitaxel and gemcitabine for first-line treatment of patients with metastatic pancreatic adenocarcinoma: phase III RESOLVE study. Ann Oncol. 2021;32(5): 600-8.

Travis et al, 2020
Travis, et al. IASLC Multidisciplinary Recommendations for Pathologic Assessment of Lung Cancer Resection Specimens After Neoadjuvant Therapy. J Thorac Oncol. 2020;15(5):709-740. doi:10.1016/j.jtho.2020.01.005.

Turcotte et al, 2015
Turcotte M, Spring K, Pommey S, Chouinard G, Cousineau I, George J, et al. CD73 is associated with poor prognosis in high-grade serous ovarian cancer. Cancer Res. 2015;75(21):4494-503.

Uramoto and Tanaka, 2014
Uramoto H, Tanaka F. Recurrence after surgery in patients with NSCLC. Transl Lung Cancer Res. 2014;3(4):242-9.

Vijayan et al, 2017
Vijayan D, Young A, Teng MWL, Smyth MJ. Targeting immunosuppressive adenosine in cancer. Nat Rev Cancer. 2017;17(12):709-24.

Wakelee et al, 2023
Wakelee H, Liberman M, Kato T, Tsuboi M, Lee SH, Gao S. Perioperative Pembrolizumab for Early-Stage Non–Small-Cell Lung Cancer. N Engl J Med. 2023;389(6):491-503.

Wang et al, 2021
Wang H, Si S, Jiang M, Chen L, Huang K, Yu W. Leukemia inhibitory factor is involved in the pathogenesis of NSCLC through activation of the STAT3 signaling pathway. Oncol Lett. 2021;22(3):663.

Wherry and Kurachi 2015
Wherry EJ, Kurachi M. Molecular and cellular insights into T cell exhaustion. Nat Rev Immunol. 2015;15(8):486-99.

Wu et al, 2020
Wu YL, Tsuboi M, He J, John T, Grohe C, Majem M, et al. Osimertinib in resected EGFR-mutated non–small-cell lung cancer. N Eng J Med 2020;383:1711-23.

Yu et al 2009
Yu X, Harden K, Gonzalez LC, Frencesco M, Chiang E, Irving B, et al. The surface protein TIGIT suppresses T cell activation by promoting the generation of mature immunoregulatory dendritic cells. Nat Immunol. 2009;10(1):48-57.

Zou and Chen, 2008
Zou W, Chen L. Inhibitory B7-family molecules in the tumour microenvironment. Nat Rev Immunol. 2008;8(6):467-77.

Zou et al, 2016
Zou W, Wolchok JD, Chen L. PD-L1 (B7-H1) and PD-1 pathway blockade for cancer therapy: Mechanisms, response biomarkers, and combinations. Sci Transl Med. 2016;8(328):328rv4.

Signature Page for VV-RIM-02709974 System v11.0
D9077C00001 Clinical Study Protocol Version 8

Approve: Document Level Task<br>Verdict: Approved	PPD<br>val<br>11-Sep-2024 11:02:15 GMT+0000
Approve: Document Level Task<br>Verdict: Approved	PPD<br>Approval<br>11-Sep-2024 16:34:47 GMT+0000
Approve: Document Level Task<br>Verdict: Approved	PPD<br>roval<br>11-Sep-2024 19:07:02 GMT+0000

Signature Page for VV-RIM-02709974 System v11.0
D9077C00001 Clinical Study Protocol Version 8

STATISTICAL ANALYSIS PLAN	
Study Code	D9077C00001
Edition Number	4.0
Date	4-Dec-2024

A Phase II Open-label, Multicentre, Randomised Study of Neoadjuvant and Adjuvant Treatment in Patients with Resectable, Early-stage (II to IIIB) Non-small Cell Lung Cancer (NeoCOAST-2)

TABLE OF CONTENTS

TITLE PAGE 1
TABLE OF CONTENTS 2
LIST OF ABBREVIATIONS 6
AMENDMENT HISTORY 9
1 INTRODUCTION 15
2 CHANGES TO PROTOCOL PLANNED ANALYSES 15
3 DATA ANALYSIS CONSIDERATIONS 15
3.1 Timing of Analyses 15
3.2 Analysis Populations 16
3.3 General Considerations 18
3.3.1 General Study Level Definitions 18
3.3.2 Visit Window 20
3.3.3 Handling of Unscheduled Visits 23
3.3.4 Multiplicity/Multiple Comparisons 23
3.3.5 Handling of Protocol Deviations in Study Analysis 24
3.3.6 Missing Dates 24
3.3.7 Sample Size 25
4 STATISTICAL ANALYSIS 26
4.1 Study Population 27
4.1.1 Patient Disposition and Completion Status 27
4.1.1.1 Definitions and Derivations 27
4.1.1.2 Presentation 27
4.1.2 Analysis Sets 28
4.1.2.1 Definitions and Derivations 28
4.1.2.2 Presentation 28
4.1.3 Protocol Deviations 28
4.1.3.1 Definitions and Derivations 28
4.1.3.2 Presentation 29
4.1.4 Demographics 29
4.1.4.1 Definitions and Derivations 29
4.1.4.2 Presentation 29
4.1.5 Baseline Characteristics 30
4.1.5.1 Definitions and Derivations 30
4.1.6 Disease Characteristics 30
4.1.6.1 Definitions and Derivations 30
4.1.6.2 Presentation 30
4.1.7 Medical History and Concomitant Disease 31
4.1.7.1 Definitions and Derivations 31
4.1.7.2 Presentation 31
4.1.8 Prior and Concomitant Medications 31
4.1.8.1 Definitions and Derivations 31

4.1.8.2 Presentation ... 32
4.1.9 Study Drug Compliance ... 32
4.1.9.1 Definitions and Derivations ... 32
4.1.9.2 Presentation ... 32
4.2 Endpoint Analyses ... 32
4.2.1 Primary Endpoint: pCR ... 33
4.2.1.1 Definition (pCR) ... 33
4.2.1.2 Derivations (pCR) ... 33
4.2.1.3 Handling of Dropouts and Missing Data (pCR) ... 33
4.2.1.4 Primary Analysis of Primary Endpoint (pCR) ... 33
4.2.1.5 Sensitivity Analyses of the Primary Endpoint (pCR) ... 33
4.2.1.6 Supplementary Analyses of the Primary Endpoint (pCR) ... 33
4.2.1.7 Subgroup Analyses (pCR) ... 33
4.2.2 Secondary Endpoint: EFS ... 34
4.2.2.1 Definition (EFS) ... 34
4.2.2.2 Derivations (EFS) ... 34
4.2.2.3 Handling of Dropouts and Missing Data (EFS) ... 38
4.2.2.4 Primary Analysis of Secondary Endpoint (EFS) ... 38
4.2.2.5 Sensitivity Analyses of the Secondary Endpoint (EFS) ... 38
4.2.2.6 Supplementary Analyses of the Secondary Endpoint (EFS) ... 38
4.2.2.7 Subgroup Analyses (EFS) ... 38
4.2.3 Secondary Endpoint: DFS ... 38
4.2.3.1 Definition (DFS) ... 39
4.2.3.2 Derivations (DFS) ... 39
4.2.3.3 Handling of Dropouts and Missing Data (DFS) ... 40
4.2.3.4 Primary Analysis of Secondary Endpoint (DFS) ... 40
4.2.3.5 Sensitivity Analyses of the Secondary Endpoint (DFS) ... 40
4.2.3.6 Supplementary Analyses of the Secondary Endpoint (DFS) ... 40
4.2.3.7 Subgroup Analyses (DFS) ... 40
4.2.4 Secondary Endpoint: Feasibility to Surgery ... 40
4.2.4.1 Definition (Feasibility to Surgery) ... 41
4.2.4.2 Derivations (Feasibility to Surgery) ... 41
4.2.4.3 Handling of Dropouts and Missing Data (Feasibility to Surgery) ... 41
4.2.4.4 Primary Analysis of Secondary Endpoint (Feasibility to Surgery) ... 41
4.2.4.5 Sensitivity Analyses of the Secondary Endpoint (Feasibility to Surgery) ... 41
4.2.4.6 Supplementary Analyses of the Secondary Endpoint (Feasibility to Surgery) ... 41
4.2.4.7 Subgroup Analyses (Feasibility to Surgery) ... 41
4.2.5 Secondary Endpoint: mPR ... 41
4.2.5.1 Definition (mPR) ... 42
4.2.5.2 Derivations (mPR) ... 42
4.2.5.3 Handling of Dropouts and Missing Data (mPR) ... 42
4.2.5.4 Primary Analysis of Secondary Endpoint (mPR) ... 42
4.2.5.5 Sensitivity Analyses of the Secondary Endpoint (mPR) ... 42
4.2.5.6 Supplementary Analyses of the Secondary Endpoint (mPR) ... 42
4.2.5.7 Subgroup Analyses (mPR) ... 42

4.2.6 Secondary Endpoint: ORR 43
4.2.6.1 Definition (ORR) 43
4.2.6.2 Derivations (ORR) 44
4.2.6.3 Handling of Dropouts and Missing Data (ORR) 44
4.2.6.4 Primary Analysis of Secondary Endpoint (ORR) 44
4.2.6.5 Sensitivity Analyses of the Secondary Endpoint (ORR) 44
4.2.6.6 Supplementary Analyses of the Secondary Endpoint (ORR) 44
4.2.6.7 Subgroup Analyses (ORR) 44
4.2.7 Secondary Endpoint: OS 44
4.2.7.1 Definition (OS) 45
4.2.7.2 Derivations (OS) 45
4.2.7.3 Handling of Dropouts and Missing Data (OS) 46
4.2.7.4 Primary Analysis of Secondary Endpoint (OS) 46
4.2.7.5 Sensitivity Analyses of the Secondary Endpoint (OS) 46
4.2.7.6 Supplementary Analysis of the Secondary Endpoint (OS) 46
4.2.7.7 Subgroup Analyses (OS) 46
4.2.8 Secondary Endpoint: ctDNA 47
4.2.8.1 Definition (ctDNA) 47
4.2.8.2 Derivations (ctDNA) 47
4.2.8.3 Handling of Dropouts and Missing Data (ctDNA) 47
4.2.8.4 Primary Analysis of Secondary Endpoint (ctDNA) 47
4.2.8.5 Sensitivity Analyses of the Secondary Endpoint (ctDNA) 47
4.2.8.6 Supplementary Analysis (ctDNA) 47
4.2.8.7 Subgroup Analyses (ctDNA) 48
4.2.9 Secondary Endpoint: PK 48
4.2.9.1 Definition (PK) 48
4.2.9.2 Derivations (PK) 48
4.2.9.3 Handling of Dropouts and Missing Data (PK) 48
4.2.9.4 Primary Analysis of Secondary Endpoint (PK) 49
4.2.9.5 Additional Analyses of the Secondary Endpoint (Pharmacokinetics) 50
4.2.9.6 Subgroup Analyses (PK) 50
4.2.10 Secondary Endpoint: Immunogenicity 50
4.2.10.1 Definition (Immunogenicity) 50
4.2.10.2 Presentations (Immunogenicity) 50
4.2.11 Safety Analyses 52
4.2.11.1 Exposure 52
4.2.11.2 Adverse Events 57
4.2.11.3 Clinical Laboratory, Blood Sample 64
4.2.11.4 Clinical Laboratory, Urinalysis 65
4.2.11.5 Other Laboratory Evaluations 66
4.2.11.6 Vital Signs 66
4.2.11.7 Electrocardiogram 66
4.2.11.8 Other Safety Assessments 67
4.2.12 Other Endpoint: Biomarkers 67
4.2.12.1 Definition: Biomarkers 67
4.2.12.2 Presentations: Biomarkers 67

4.3 Pharmacodynamic Endpoint(s) 67
4.3.1 Analysis 67
4.3.2 Definitions and Derivations 67
4.3.3 Presentation 68
5 INTERIM ANALYSIS 68
6 REFERENCES 69
7 APPENDICES 70
Appendix A RECIST 70

LIST OF ABBREVIATIONS

Abbreviation or special term	Explanation
ADA	Anti-drug antibody
AE	Adverse event
AEF	Alive and Event-free
AESI	Adverse event of special interest
AJCC	American Joint Committee on Cancer
ALT	Alanine aminotransferase
AST	Aspartate aminotransferase
ATC	Anatomical therapeutic chemical
BILI	Bilirubin
BIPR	Blinded Independent Pathological Review
BMI	Basal metabolic index
BOR	Best overall response
BP	Blood pressure
BSR	Baseline scaled ratio
CCr	Creatinine clearance rate
CDL	Clinical Data Lock
CI	Confidence interval
COVID-19	Coronavirus Disease 2019
CR	Complete response
CRF	Case report forms
CSP	Clinical Study Protocol
CSR	Clinical Study Report
ctDNA	Circulating tumour DNA
CT	Computerised tomography
CV	Coefficient of variation
Dato-DXd	Datopotamab Deruxtecan
DCO	Data cut off
DFS	Disease-free Survival
ECG	Electrocardiogram
ECOG	Eastern Cooperative Oncology Group
eCRF	electronic Case Report Form
EFS	Event-free Survival
GFR	Glomerular filtration rate

gCV	Geometric coefficient of variation
gSD	Geometric standard deviation
IASLC	International Association for the Study of Lung Cancer
ICF	Informed Consent Form
imAE	Immune mediated adverse event
IP	Investigational Product
IPD	Important Protocol Deviation
ITT	Intention-to-Treat
IRT	Interactive Response Technology
IV	Intravenous
LLOQ	Lower limit of quantification
LD	Longest Diameter
MedDRA	Medical Dictionary for Regulatory Activities
mPR	Major pathological response
MRI	Magnetic resonance imaging
NCI CTCAE	National Cancer Institute Common Terminology Criteria for Adverse Events
NE	Non-evaluable
NED	No evidence of disease
NQ	Non-quantifiable
NS	No sample
NTL	Non-target lesion
OAE	Other significant adverse events
OR	Objective response
ORR	Objective response rate
OS	Overall survival
pCR	Pathological complete response
PD	Progressive disease
PD-L1	Programmed cell death ligand 1
PFS	Progression-free survival
PK	Pharmacokinetics
PR	Partial response
PT	Preferred term
RDI	Relative dose intensity
RECIST v1.1	Response Evaluation Criteria in Solid Tumours version 1.1
RVT	Residual viable tumour
SAE	Serious adverse event

SAP	Statistical Analysis Plan
SD	Stable disease
SoA	Schedule of assessment
SOC	System organ class
SRC	Safety Review Committee
Std Dev	Standard deviation
TEAE	Treatment-emergent adverse events
TFL	Tables, figures, listings
TL	Target lesion
ULN	Upper limit of normal range
WHO	World Health Organisation

AMENDMENT HISTORY

CATEGORY Change refers to:	Date	Description of change	In line with CSP?	Rationale
N/A	19 Apr 2022	Initial approved SAP	N/A	N/A
Data presentation	23 May 2023	Section 1 updated to include reference to the latest CSP and CRF and schedule of assessments for all the 5 arms of CSP V4.	Yes	New update
Data presentation	23 May 2023	Analysis population (Section 3.2 Updated [redacted] Resected set and modified ITT set are added. Wording for safety and immunogenicity set are updated)	Yes	Clarification
Data presentation	23 May 2023	Analyses based on PD-L1 results and definition of periods in Section 3.3.1 are updated	Yes	New update
Data presentation	23 May 2023	Visit windows updated in section 3.3.2 of [redacted] and [redacted] arms for adjuvant period	Yes	Update based on CSP V4.0
Data presentation	23 May 2023	Imputation rule for partial death date is updated	Yes	This is to align the imputation rule of death date with latest early oncology SAP template
Data presentation	23 May 2023	Concomitant medication text removed from section 4.1.8.2	Yes	Concomitant medication is described in section 4.1.8.1 and text in section 4.1.8.2 is redundant.
Subgroup analyses of primary endpoint	23 May 2023	Description of subgroup analysis updated for patients who received surgery by treatment group	Yes	Clarification
Secondary endpoint(s)	23 May 2023	• Definition of event free survival updated in section 4.2.2.1 to include local or distant recurrence as determined by investigator.<br>• Added more wording for censoring rule in section Table 4.	Yes	Clarification.<br>Also to update the two missed visit rule according to the schedule of RECIST assessments of CSP V4.0

CATEGORY Change refers to:	Date	Description of change	In line with CSP?	Rationale
		• Removed 'potential' from duration of follow up.<br>• Removed supplementary analysis from section 4.2.2.6.		
Derivation of secondary endpoint(s)	23 May 2023	Removed 'No tumour assessments post surgery' from situation column of Table 5 in section 4.2.3.2	Yes	Clarification
Secondary endpoint(s)	23 May 2023	Updated the primary analysis of DFS using the resected set in section 4.2.3.4	Yes	New update
Derivation of secondary endpoint(s)	23 May 2023	Added neoadjuvant in the definition of feasibility of surgery in section 4.2.4.1	Yes	Clarification
Secondary endpoint(s)	23 May 2023	Updated the primary analysis of feasibility to surgery using the safety set in section 4.2.4.4	Yes	New update
Secondary endpoint(s)	23 May 2023	Added supplementary analyses for feasibility to surgery	Yes	New update
Secondary endpoint(s)	23 May 2023	Updated subgroup analyses for mPR	Yes	Clarification
Data presentation	23 May 2023	New categories added for immunogenicity in section 4.2.10.2	Yes	New update
Data presentation	23 May 2023	Section 4.2.11.1.1 updated for the definitions of all the arms including [redacted] Dato-DXd and [redacted]	Yes	CSP V4.0 updated for new arms. Clarification
Data presentation	23 May 2023	Section 4.2.11.1.2 updated to include the arms [redacted] Dato-DXd and [redacted]	Yes	CSP V4.0 updated for new arms
Data presentation	23 May 2023	Section 4.2.11.2.1 updated to include arms [redacted] Dato-DXd and [redacted]	Yes	CSP V4.0 updated for new arms
Data presentation	23 May 2023	Section 4.2.11.2.2 updated to include arms [redacted] Dato-DXd and [redacted]	Yes	CSP v4.0 updated for new arms

CATEGORY Change refers to:	Date	Description of change	In line with CSP?	Rationale
		Adverse events of possible interest added		New update
Derivation of secondary endpoint(s)	23 May 2023	Treatment-emergent and off-treatment definitions updated to add surgery and exclude radiotherapy in section 4.2.11.2.1	Yes	Clarification
Data presentation	23 May 2023	Section 5 (interim analysis) updated for futility criteria	Yes	Threshold changed for futility criteria
Data presentation	23 May 2023	• Appendix A Table 7 and Table 11 updated for Not Evaluable visit response definition<br>• Nadir definition and new lesion clarified<br>• Irradiated lesions/lesion intervention section added in Appendix A	Yes	Clarification
N/A	02 Jun 2023	Approved SAP v2.0	Yes, version 4	N/A
Data presentation	20 Sep 2023	PD-L1 values to be presented as continuous values when ranges are collected	Yes	New update
Data presentation	23 Nov 2023	Analysis population (Section 3.2 Updated. [redacted], exploratory mITT added for Final efficacy analyses, and PK and immunogenicity analysis sets definitions updated)	Yes	New update
Data presentation	23 Nov 2023	Section 3.3.7 Sample size and Section 5 Interim Analysis updated to accommodate changes of LRV and TV in the GNG decision framework based on emerging data	Yes	Clarification
Secondary endpoint(s)	23 Nov 2023	Section 4.2.4.6 Secondary Endpoint: ORR Definition updated to add details about BOR and best response of SD	Yes	New update

CATEGORY Change refers to:	Date	Description of change	In line with CSP?	Rationale
Secondary endpoint(s)	23 Nov 2023	Section 4.2.6.1 Secondary Endpoint: the Clavien-Dindo Classification of surgical complications will be presented as supplementary analyses	Yes	New update
Data presentation	23 Nov 2023	Naming for [redacted] used in previous SAP versions has been updated to [redacted]	Yes	To align with CSP updates
Data presentation	23 Nov 2023	Database lock (DBL) updated to clinical data lock (CDL) throughout the document	Yes	To align with new AZ SOP
Derivation of secondary endpoint(s)	16 Jan 2024	Section 4.2.11.1.1: Dose intensity section updated to add definitions of actual cumulative dose, intended cumulative dose, and planned dose at each cycle for each study drug	Yes	Clarification
Secondary endpoint(s)	16 Jan 2024	Sections 4.2.1.6 and 4.2.5.6: a Waterfall plot will be presented for pCR and mPR as supplementary analyses	Yes	New update
Data presentation	16 Jan 2024	Section 4.2.11.2.2: Data collected for Interstitial Lung Disease / pneumonitis investigation will be listed as part of Dato-DXd AESI	Yes	New update
Secondary endpoint(s)	16 Jan 2024	Sections 4.2.11.4.1 and 4.2.11.4.2: Urinalysis data presentation has been updated.	Yes	New update
Secondary endpoint(s)	06 Feb 2024	Sections 4.2.2.2 and 4.2.3.2: Removed the word "evaluable" from the definitions of censoring rules for EFS and DFS	Yes	New update
N/A	14 Feb 2024	Approved SAP v3.0	Yes, version 5	N/A
Secondary endpoint(s)	11 Apr 2024	Section 4.2.10.2: New ADA categories and summary tables were added. [redacted] specific categories were added.	Yes	New update
Secondary endpoint(s)	17 Jun 2024	Subgroup analyses by stage (II, IIIA, IIIB) were added in sections 4.2.1.7, 4.2.2.7, 4.2.3.7, 4.2.5.7, 4.2.6.7 and 4.2.7.7	Yes	New update
Data presentation	17 Jun 2024	Section 3.2: ctDNA analysis population added	Yes	New update

CATEGORY Change refers to:	Date	Description of change	In line with CSP?	Rationale
Data presentation	28 Jun 2024	Flexible language added for optional analysis	Yes	New update
Secondary endpoint(s)	02 Jul 2024	Supportive analyses using mITT population were added in sections 4.1.4.2, 4.1.5.1, 4.1.6.2, 4.2.1.6, 4.2.2.6, 4.2.5.6, 4.2.6.6 and 4.2.7.6	Yes	New update
Primary and secondary endpoint(s)	13 Sep 2024	CCI [redacted]	Yes	New update
Secondary endpoint(s)	13 Sep 2024	Text added to clarify that new equivocal lesions must be converted to unequivocal at a follow-up visit to contribute to a PD	Yes	New update
Stratification factors	13 Sep 2024	Section 4.1.3.2: Stratification factors added for new treatment arms 6 and 7 (PD-L1 1-49% versus ≥50%)	Yes	New update
Secondary endpoint(s)	13 Sep 2024	Section 4.1.6.2: For treatment arms 6 and 7, local results of PD-L1 should be used. For all other treatment arms, central results of PD-L1 should be used when possible.	Yes	New update
Secondary endpoint(s)	30 Oct 2024	Section 4.2.8.6: The association of ctDNA with clinical endpoints may be analyzed based ctDNA clearance status alone or in combination with pCR/mPR status.	Yes	New update
Secondary endpoint(s)	19 Sep 2024	Section 4.2.11.2.2: List of AE summary tables amended to keep only the ones required as per the core list of tables, study-specific, or needed for the SRC. Flexible language used for optional tables.	Yes	New update

CATEGORY Change refers to:	Date	Description of change	In line with CSP?	Rationale
Secondary endpoint(s)	13 Nov 2024	Section 4.2.11.2.2: AEs leading to dose omission of each IP and chemotherapy added to the overall summary table and to the summary by SOC and PT	Yes	New update
N/A		Approved SAP v4.0	Yes, version 8	N/A

1 INTRODUCTION

The purpose of this document is to give details for the statistical analysis of study D9077C00001 supporting the clinical study report (CSR). The reader is referred to the latest clinical study protocol (CSP) and the latest case report form (CRF) for details of objectives, study design, study conduct and data collection.

The study design schema is presented in Figure 1 of the CSP, and the Schedule of Activities (SoA) are presented in Table 1, Table 2, Table K18, Table K19, Table L20, Table L21, Table M22, Table M23, Table N26, Table N27, Table O29, Table O30, Table P31, Table P32, Table Q33 and Table Q34 of the CSP.

2 CHANGES TO PROTOCOL PLANNED ANALYSES

No changes from the planned analyses specified in the protocol.

3 DATA ANALYSIS CONSIDERATIONS

3.1 Timing of Analyses

There are interim analysis and final analysis planned for each arm separately.

An interim futility analysis primarily evaluating pCR will be performed once approximately 35 patients in each arm have received neoadjuvant treatment and received surgery or discontinued without receiving surgery. Refer to Section 5 for further details of the planned interim analyses.

The final analysis for each arm and the overall study will take place when all patients in the arm or the overall study complete 1 year of adjuvant therapy plus the follow-up period, or complete the end-of-study visit if withdrawing early from treatment, respectively. In the event that the study or certain treatment arms are terminated early by sponsor decision, the data cut-off may occur before every patient completes all the scheduled assessments. Primary and secondary endpoints including pathological complete response (pCR), objective response rate (ORR), event-free survival (EFS), disease-free survival (DFS), feasibility to surgery, major pathological response (mPR), and overall survival (OS) will be analysed during the final analysis.

Individual CSRs for each arm may be written after the interim clinical data lock (CDL) preceding the final analysis for each arm respectively. A final integrated CSR will be written after the final CDL containing all arms.

An early safety evaluation will be done by a safety review committee (SRC) to review all available data when the first [redacted] patients in each treatment arm have completed [redacted] cycles of

neoadjuvant treatment. An additional review of safety data by the SRC will occur when the first [redacted] patients in each treatment arm have undergone surgery and have had [redacted] days of follow-up, in order to assess perioperative mortality and surgery delays. For more details, refer to the SRC charter.

Additionally, throughout the study if more than [redacted]% of patients experience a delay in surgery of more than 8 weeks or become unable to receive surgery due to toxicity on any treatment, enrolment in that treatment will be halted and SRC members will be notified to review these cases and provide their recommendation to the Sponsor.

The SRC will meet regularly at approximately 6-month intervals to review the safety and tolerability of treatment as neoadjuvant and adjuvant regimen, until all patients have had the opportunity to undergo surgery and those having surgery had at least 6 months of adjuvant treatment.

3.2 Analysis Populations

All patients who received any amount of study interventions are included in the Safety set. For the safety and PK analyses, patients are classified according to the actual treatment received. For all efficacy analyses, and for baseline and demography, patients are classified according to the treatment they were randomised to (i.e. the planned treatment).

The following populations are defined in Table 1:

Table 1 Populations for Analysis

Population/Analysis set	Description	Endpoint/Output
Enrolled	All patients who sign the ICF	Disposition
ITT	All randomised patients in the study	Baseline and demography<br>pCR, mPR<br>EFS, EFS12 and EFS24<br>OS, OS12 and OS24
Resected	All the patients in the ITT who had surgical resection following the neoadjuvant period, who do not have R2 margins, and whose first scan following surgery shows no evaluable disease (defined as no post-surgery R2 margins and no RECIST evidence of disease based on the investigator data i.e., no target lesions, non-target lesions or new lesions (unless they are equivocal or pathologically confirmed to be a new primary malignancy))	DFS, DFS12 and DFS24<br>& Subgroup / Supplementary analyses
modified ITT (mITT)	All randomised patients with confirmed NSCLC histology who received at least 1 dose of any study intervention	Baseline and demography [supportive]<br>pCR [supportive]

Population/Analysis set	Description	Endpoint/Output
		mPR [supportive]<br>EFS [supportive]<br>EFS12 and EFS24 [supportive]<br>OS [supportive]<br>OS12 and OS24 [supportive]<br>ORR [supportive]<br>& Subgroup / Supplementary analyses, unless otherwise specified
Exploratory mITT	All patients in the mITT who received surgery	pCR<br>mPR
[redacted]	CCI [redacted]	[redacted]
[redacted]	[redacted]	[redacted]
Response evaluable	All dosed patients who had measurable disease at baseline	ORR
Safety	All patients who received at least 1 dose of any study intervention	Exposure<br>Adverse Events<br>Laboratory evaluations<br>Vital Signs<br>ECG<br>Feasibility to surgery
Pharmacokinetics (PK)	All patients who received at least 1 dose of study intervention with at least 1 post-dose reportable PK concentration for that study intervention.	PK concentrations
Immunogenicity	All patients who received at least 1 dose of study intervention with at least one non-missing ADA result at any time, either baseline or post-baseline, according to the actual treatment received	ADAs
ctDNA	All patients in the mITT who had at least one sample processed	ctDNA

ADA = anti-drug antibody ; ctDNA = Circulating tumour DNA ; DCF = Disease free survival; DCF12 = Disease free survival at 12 months; DCF24 = Disease free survival at 24 months; ECG = electrocardiogram; ICF = informed consent form; ITT = intention-to-treat; mPR = major pathological response; ORR = objective response rate; OS = overall survival; OS12 = overall survival at 12 months; OS24 = overall survival at 24 months; pCR = pathological complete response, EFS = event free survival; EFS12 = event free survival at 12 months; EFS24 = event free survival at 24 months; PK = pharmacokinetics.

* Individual PK concentration and parameter data for any patients who are excluded from the descriptive summary tables, figures and/or inferential statistical analyses are included in the listings and are flagged with an appropriate footnote.

3.3 General Considerations

3.3.1 General Study Level Definitions

The general principles described below are followed throughout the study:

- Continuous endpoints will be summarised by the number of observations, mean, standard deviation (Std Dev), median, upper and lower quartiles (as applicable), minimum, and maximum. For data that requires log-transformation, geometric mean, coefficient of variation (CV), median, minimum and maximum will be presented. Categorical endpoints will be summarised by frequency counts and percentages for each category.

- If data are available for less than 3 patients, no summary statistics other than minimum, maximum and number of observations will be presented.

- Unless otherwise stated, percentages will be calculated out of the analysis set total (excluding efficacy and exposure) and for treatment group.

- For continuous data, descriptive summary statistics (mean, median, standard deviation, standard error, confidence intervals) will be rounded to 1 additional decimal place compared to the original data. Minimum and maximum will be displayed with the same accuracy as the original data.

- Derived variables will be rounded to 1 more decimal place compared to the least number of decimal places among the raw data used for calculation, provided the scale of the data is not changing.

- For categorical data, percentages will be rounded to 1 decimal place.

- SAS® version 9.4 (as a minimum) will be used for all analyses.

- Baseline is the last non-missing value obtained prior to the first dose/administration of any study treatment and any information taken after first dose/administration of study treatment is regarded as post-baseline information. If two visits are equally eligible to assess patient status at baseline (eg, screening and baseline assessments both on the same date prior to first dose/administration with no washout or other intervention in the screening period), the average is taken as the baseline value with the exception of ctDNA. For ctDNA the Cycle 1 pre-dose sample will be regarded as baseline if multiple pre-treatment samples are collected and analysed. For non-numeric laboratory tests (i.e. some of the urinalysis parameters) where taking an average is not possible then the best value is taken as baseline as this is the most conservative. In the scenario where there

are two assessments on Day 1 prior to first dose, one with time recorded and the other without time recorded, the one with time recorded is selected as baseline. Where safety data are summarised over time, study day is calculated in relation to date of first treatment. For assessments on the day of first dose where time is not captured, a nominal pre-dose indicator, if available, serves as sufficient evidence that the assessment occurred prior to first dose. Assessments on the day of the first dose where neither time nor a nominal pre-dose indicator are captured is considered prior to the first dose if such procedures are required by the protocol to be conducted before the first dose. If no value exists before the first dose/administration, then the baseline value is treated as missing. For the analysis which summarise values by visit will use different baseline values for visit windowing (see section 3.3.2)

- In all summaries, change from baseline endpoints will be calculated as the post-treatment value minus the value at baseline. The percentage change from baseline is calculated as (post-baseline value - baseline value) / baseline value × 100. For any endpoint subjected to log transformation, the change from baseline calculated and summarised on the log scale are back-transformed and presented as a 'baseline scaled ratio' (BSR). Percentage change is then calculated as (BSR - 1) × 100.]

- Unless stated otherwise, two-sided confidence intervals are produced at 95%.

- For percentiles of survival times based on the Kaplan-Meier method (eg, median survival), CI will be calculated using the default method available in the SAS LIFETEST procedure (ie, the Klein and Moeschberger extension of the Brookmeyer-Crowley method).

- For point-estimates of survival based on the Kaplan-Meier method (eg, for EFS and DFS), CI will be calculated using the default method available in the SAS LIFETEST procedure (ie, using Greenwood's estimate of standard error and a log-log transformation).

- For the purposes of summarising safety data assessed at visits, in addition to baseline data, only on treatment data are included in the summary tables. On treatment data is defined as data after the first dose of investigation product (IP) and with assessment date up to and including the date of last IP (or surgery, whichever is later) + 90 days and prior to start of any subsequent cancer therapy (excluding radiotherapy).

- Missing safety data will generally not be imputed. However, safety assessment values of the form of "<x" (i.e, below the lower limit of quantification) or >x (ie, above the upper limit of quantification) will be imputed as "x" in the calculation of summary statistics but displayed as "<x" or ">x" in the listings.

- Global country situation referred to in this plan (if any) is related to COVID-19 analysis.

- PD-L1 source data will be used for subgroup analyses. For treatment arms 6 and 7, local results should be used. For all other treatment arms, central results will be used for patients who have central results, otherwise local results will be used. In absence of source data, stratification factors as recorded in the IRT will be used. PD-L1 cut offs other than 1% may be explored. If the original value is a range, then values will be rounded to the lowest integers for continuous summary, for example, 20-30 will be converted into 20, ≥1 and <5 will be converted into 1, and >50 will be converted into 50.

For any safety summaries by period, the following data will be included:

- **Neoadjuvant period**: Date of first dose of neoadjuvant study treatment until the date of surgery for patients who undergo the surgery; For patients who did not undergo surgery, this period is up to min (date of last dose of neoadjuvant treatment + 90 days, date of first dose of subsequent anti-cancer therapy (excluding radiotherapy), date of DCO). Note: For assessments recorded on the day of surgery, time will be used to determine if it's pre or post surgery, if time is not available it will be assumed to occur post surgery.

- **Post-surgery period:** Date of the day of surgery until min (day of first dose of study treatment post-surgery, date of DCO, date of first dose of subsequent anti-cancer therapy (excluding radiotherapy), date of surgery + 90 days).

- **Adjuvant period**: Date of first dose of study treatment post-surgery until min (last study treatment post-surgery + 90 days, date of DCO, date of first dose of subsequent anti-cancer therapy (excluding radiotherapy)).

- **Overall period:** First dose of study treatment until min (last dose of study treatment (or surgery, whichever is later) + 90 days, date of DCO, date of first dose of subsequent anti-cancer therapy (excluding radiotherapy)).

3.3.2 Visit Window

For safety, time windows are defined for any presentations that summarise values by visit. The following conventions apply:

- The time windows are exhaustive so that data recorded at any timepoint has the potential to be summarised. Inclusion within the time window are based on the actual date and not the intended date of the visit.
- All unscheduled visit data have the potential to be included in the summaries.
- The window for the visits following baseline are constructed in such a way that the upper limit of the interval falls half way between the two visits (the lower limit of

the first post-baseline visit is Day 2). If an even number of days exists between two consecutive visits, then the upper limit is taken as the midpoint value minus 1 day.

- Time windowing will be done separately for each assessment based on the schedule of events specific to that assessment.

- Should Study Day be missing (due to partial or missing dates), then visit will be assigned to the nominal visit at which that assessment was recorded, and no windowing will be performed.

- Visit windowing will be conducted up to and including the end of treatment visit. That is, the end of treatment visit will be reassigned to a scheduled visit based on the study day the end of treatment visit occurred at.

  Neoadjuvant period (21 day cycle), calculated using the day of the first dose of study interventions as Day 1. For assessments up to and including last neoadjuvant dose, the following windows are used:

  - Week 0 (Neoadjuvant baseline), visit window (up to and including the date of first dose) Note: only assessments before the first dose of neoadjuvant treatment are considered valid for baseline, e.g., Neoadjuvant cycle 1 day 1 pre-dose or screening)
  - Week 3; nominal day 22, visit window (latest of Day 2 or date of first dose+1) – 32
  - Week 6; nominal day 43, visit window 33 – 53
  - Week 9; nominal day 64, visit window 54 – 74

  In addition, there will be a summary of patients who complete or discontinue treatment in the neoadjuvant phase, but do not enter the adjuvant phase. For this summary these patients only will have their post-treatment discontinuation follow-up assessments windowed to the 'Follow-up schedule' based on the last day of study medication. For compliance calculations, all data collected after a subject permanently discontinues study treatment will use these windows and neoadjuvant treatment discontinuation visits and adjuvant treatment discontinuation visits will be presented separately.

  - Follow up Day 30, visit window Days 2 - 46
  - Follow up Month 2, visit window Days 47 - 76
  - Follow up Month 3, visit window Days 77 - 136

  Adjuvant period (28 day cycle), calculated using the day of the first dose of durvalumab/oleclumab/monalizumab following surgery as Day 1, or the date of the first assessment post-surgery for subjects who have not started adjuvant treatment. Use windows regardless of treatment discontinuation:

- Week 0 (Adjuvant baseline), visit window: Date of surgery + 1 to day 1, Note: only assessments before the first dose of adjuvant treatment, or the first assessment post-surgery for subjects who have not started adjuvant treatment are considered valid for baseline e.g., Adjuvant cycle 1 day 1 pre-dose
- Week 4; nominal day 29, visit window 2 – 43
- Week 8; nominal day 57, visit window 44 – 71
- Week 12; nominal day 85, visit window 72 – 99
- Week 16; nominal day 113, visit window 100 – 127
- Week 20; nominal day 141, visit window 128 – 155
- Week 24; nominal day 169, visit window 156 – 183
- Week 28; nominal day 197, visit window 184 – 211
- Week 32; nominal day 225, visit window 212 – 239
- Week 36; nominal day 253, visit window 240 – 267
- Week 40; nominal day 281, visit window 268 – 295
- Week 44; nominal day 309, visit window 296 – 323

CCI [redacted]

- [redacted]
- [redacted]
- [redacted]
- [redacted]
- [redacted]
- [redacted]
- [redacted]
- [redacted]
- [redacted]

[redacted]

CCI [redacted]

- [redacted]
- [redacted]
- [redacted]
- [redacted]
- [redacted]
- [redacted]
- [redacted]
- [redacted]
- [redacted]

…

- For summaries showing the maximum or minimum values, the maximum/minimum value recorded on treatment are used (regardless of where it falls in an interval).
- Listings display all values contributing to a time point for a patient.
- For visit based summaries, if there is more than one value per patient within a time window then the closest value to the scheduled visit date are summarised, or the earlier, in the event the values are equidistant from the nominal visit date. The listings highlight the value for the patient that contributed to the summary table, wherever feasible. Note: in summaries of extreme values, all post baseline values collected are used including those collected at unscheduled visits regardless of whether or not the value is closest to the scheduled visit date.
- For summaries at a patient level, all values are included, regardless of whether they appear in a corresponding visit-based summary, when deriving a patient level statistic such as a maximum.

3.3.3 Handling of Unscheduled Visits

See Section 3.3.2 for safety.

3.3.4 Multiplicity/Multiple Comparisons

Not Applicable.

3.3.5 Handling of Protocol Deviations in Study Analysis

Important protocol deviations (IPDs) may include, but are not limited to the following:

- Patients randomised but who did not receive study treatment (Deviation 1)
- Patients who deviate from any entry criteria per the CSP (Deviation 2)
- Baseline RECIST CT/MRI scan >28 days before start date of randomisation and/or baseline PET scan >42 days before start date of randomisation (Deviation 3)
- No baseline RECIST 1.1 assessment on or before date of randomisation (Deviation 4)
- Patients who received prohibited concomitant medications during study period. (Deviation 5). See CSP Appendix H1
- Patients who met study intervention discontinuation criteria but continued study intervention, and potentially had major impact to safety of patients according to clinical judgement (Deviation 6)
- RECIST scans performed outside of the scheduled window on more than 1 occasion (Deviation 7)
- Patients randomised who received their randomised study treatment at an incorrect dose or received an alternate study treatment to that which they were randomised (Deviation 8)
- Missed visits, assessments, or treatments that, in the opinion of the investigator were due to the COVID-19 global pandemic and where there was a significant effect on either completeness, accuracy and/or reliability of the patient data, or the patient's rights, safety or wellbeing (Deviation 9)

More details will be provided in protocol deviation assessment plan. None of the deviations lead to patients being excluded from any analysis populations described in the SAP, unless otherwise specified. If a deviation is serious enough to have a potential impact on the primary analysis, sensitivity analyses may be performed. A list of all protocol deviations is reviewed and decisions regarding how to handle these deviations are documented by the study team physician, clinical pharmacology scientist and statistician prior to database lock.

3.3.6 Missing Dates

Generally, the imputation of dates is used to decide if an observation is treatment emergent for adverse events or concomitant medications. The imputed dates should not be used to calculate durations, where the results would be less accurate.

The following are the guidelines used when partial dates are detected in the study:

- For missing diagnostic dates (e.g., disease diagnosis), if day and/or month are missing use 01 and/or Jan. If year is missing, put the complete date to missing.

- For missing AE and concomitant medication start dates, the following is applied:

    a. Missing day - impute the 1st of the month unless month is the same as month of the first dose of study drug then impute first dose date.

    b. Missing day and month - impute 1st January unless year is the same as first dose date then impute first dose date.

    c. Completely missing - impute first dose date unless the end date suggests it could have started prior to this in which case impute the 1st January of the same year as the end date.

    d. Imputed start date should be no later than the end date.

- For missing AE and concomitant medication end dates, the following is applied:

    a. Missing day - impute the last day of the month unless both the month and the year are the same as the last dose date or the primary analysis data cut-off date then impute the last dose date or the primary analysis data cut-off date.

    b. Missing day and month - impute 31st December unless the year is the same as the last dose date or the primary analysis data cut-off date then impute the last dose date or the primary analysis data cut-off date. Flags are retained in the database indicating where any programmatic imputation has been applied, and in such cases, any durations would not be calculated.

    c. Completely Missing – need to look at whether the AE/medication is still ongoing before imputing a date and also when it started in relation to study drug. If the ongoing flag is missing, then assume that AE is still present / medication is still being taken (i.e. do not impute a date). If the AE/medication has stopped and start date is prior to first dose date, then impute first dose date. Or if it started on or after first dose date then impute a date that is after the last dose of study drug date.

- If a patient is known to have died where only a partial death date is available, then the date of death will be imputed according to the rules for imputing AE start dates unless this date is before the last date the patient is known to be alive then the date of death will be imputed as the date the patient was last known to be alive +1.

  If death has been recorded but the date is entirely missing, then date of death will be imputed as the date the patient was last known to be alive +1.

3.3.7 Sample Size

The sample size of up to 70 patients per treatment arm is to:

- Ensure the accuracy of making a Go or No-Go decision at the interim analysis and final analysis.
- Obtain a preliminary assessment of anti-tumour activity with a certain degree of precision.

The sample size is not based on type I error and power considerations. Instead, it is based on the model-based drug development approach (Lalonde et al., 2007; Frewer et al., 2016). In this decision framework, the maximum false-go risk is set to be [redacted]% while maximum false-stop risk is [redacted]%. The sample size of up to 70 is calculated such that there is at least an [redacted]% chance of making a go decision at the final analysis if the true pCR rate is at target value for the respective treatment arm. Single-arm analyses are conducted. Meanwhile, the ultimate Go No-Go decision will be based on the totality of the data including, but not limited to, efficacy, safety, and biomarker data. The estimated pCR rate and its two-sided [redacted]% exact CIs can be found in Table 2.

[redacted] [redacted]

[redacted]	[redacted]	[redacted]	[redacted]	
			[redacted]	[redacted]
[redacted]				
[redacted]	[redacted]	[redacted]	[redacted]	[redacted]
[redacted]	[redacted]	[redacted]	[redacted]	[redacted]
[redacted]	[redacted]	[redacted]	[redacted]	[redacted]
[redacted]	[redacted]	[redacted]	[redacted]	[redacted]
[redacted]				
[redacted]	[redacted]	[redacted]	[redacted]	[redacted]
[redacted]	[redacted]	[redacted]	[redacted]	[redacted]
[redacted]	[redacted]	[redacted]	[redacted]	[redacted]
[redacted]	[redacted]	[redacted]	[redacted]	[redacted]

[redacted] plan to enroll [redacted] patients per arm to evaluate tolerability of alternate dose regimens, with a potential to increase to [redacted] patients per arm to further understand safety and efficacy. Arm 4 may enroll 50 patients if it is sufficient to understand safety and efficacy. Arm 6 may enroll [redacted] patients if it is sufficient to understand safety and preliminary efficacy.

4 STATISTICAL ANALYSIS

This section provides information on definitions, derivation and analysis/data presentation per domain.

4.1 Study Population

The domain study population covers patient disposition, analysis sets, protocol deviations, demographics, baseline characteristics medical history, prior and concomitant medication and study drug compliance.

4.1.1 Patient Disposition and Completion Status

4.1.1.1 Definitions and Derivations

Study participation (i.e., a patient is "enrolled") and randomisation is defined in CSP Section 5. Completion of study is defined in CSP Section 4.4.

4.1.1.2 Presentation

Patient disposition including screen failures and reason for screen failure is summarised and listed based on all patients enrolled (i.e., informed consent signed) by treatment and for all patients combined as defined by the current relevant tables, figures, and listings (TFL) standards. The number and percentage of patients for the following will be summarised:

- Patients enrolled;
- Patients who screen failed;
- Reason for screen failures;
- Patients randomised;
- Patients who received treatment;
- Patients who were randomised but did not receive treatment;
- Patients who discontinued treatment in the neoadjuvant period, i.e. discontinued any part of the study intervention;
- Patients who discontinued treatment in the adjuvant period, i.e. discontinued any part of the study intervention;
- Reasons for treatment discontinuation for both neoadjuvant and adjuvant period (including the reason due to global/country situation) – presented separately for each study intervention;
- Patients who completed or discontinued the neoadjuvant period but did not undergo surgery;
- Reasons for surgery not done;
- Patients who completed or discontinued the neoadjuvant period and received surgery;

- Patients who did not enter the adjuvant period;
- Patients completing surgery but not continuing to adjuvant period;
- Reasons for adjuvant treatment not given;
- Patients who had delay in receiving adjuvant treatment;
- Reasons for delay in adjuvant treatment;
- Patients ongoing study treatment at data cut-off (DCO) date;
- Patients ongoing study at DCO date;
- Patients who terminated study;
- Reasons for study termination.

The number of patients by region, country and centre and disruptions due to global/country situation (COVID-19) may also be summarised. The number of patients with confirmed/suspected COVID-19, and number of patients with confirmed/suspected COVID-19 who died may also be presented for the ITT analysis set.

Listings will be presented for disposition details for discontinued patients and patients ongoing in the study.

Listings may be presented for:

- Disposition due to global/country situation
- Patients with reported issues in the Clinical Trial Management System due to the global/country situation

4.1.2 Analysis Sets

4.1.2.1 Definitions and Derivations

For the definitions of each analysis set, refer to Section 3.2.

4.1.2.2 Presentation

The analysis sets are summarised by treatment group and for all patients combined. Any exclusions from analysis sets are listed.

4.1.3 Protocol Deviations

4.1.3.1 Definitions and Derivations

The list of the categories is presented in Section 3.3.5.

4.1.3.2 Presentation

The incidence of IPDs are summarised for the ITT analysis set by deviation categories. The number and percentage of patients in the following categories are summarised:

- Number of patients with at least 1 important protocol deviation;
- Number of patients with at least 1 pandemic related important protocol deviation;
- Number of patients with at least 1 important protocol deviation, excluding pandemic related IPDs.

A listing is provided with the important protocol deviation details.

Errors in stratifications (based upon stratification information recorded in the IRT and source data) may also be summarised separately to the important protocol deviations in order to show any discrepancies. Patients will be stratified by baseline PD-L1 expression status (1-49% versus ≥50% for treatment arms 6 and 7, and < 1% versus ≥ 1% for all other treatment arms).

4.1.4 Demographics

4.1.4.1 Definitions and Derivations

Age (years) will be grouped accordingly: ≥ 18 - <50, ≥ 50 - < 65, ≥ 65 - < 75 and ≥ 75. Each race category counts patients who selected only that category.

4.1.4.2 Presentation

Demographics will be summarised and listed based on the ITT analysis set by treatment group and for all patients combined as defined by the current relevant TFL standards. The following will be summarised:

- age (years)
- age group
- sex
- race
- ethnicity
- region
- country

A supportive analysis of demographics is based on the mITT.

Demographic characteristics in patients with confirmed/suspected COVID-19 infection may also be summarised and listed similarly.

4.1.5 Baseline Characteristics

4.1.5.1 Definitions and Derivations

Baseline characteristics are listed and summarised for the ITT analysis set population by treatment group and for all patients combined as defined by the current relevant TFL standards. Body mass index (BMI) (kg/m^2) will be calculated as: weight/(height^2). Weight (kg) will be grouped accordingly: <50, ≥ 50 - < 70, ≥ 70 - <90 and ≥ 90, and BMI will be grouped accordingly: <18.5, ≥18.5 - < 25.0, ≥ 25.0 - < 30.0 and ≥30.0.

The following will be summarised by treatment group using the ITT analysis set:

- weight (kg)
- weight group
- BMI (kg/m^2)
- BMI group
- Smoking status (current, former, never)
- Number of pack-years
- Eastern Cooperative Oncology Group (ECOG) performance status

A supportive analysis of baseline characteristics is based on the mITT.

4.1.6 Disease Characteristics

4.1.6.1 Definitions and Derivations

Not applicable

4.1.6.2 Presentation

Disease characteristics at baseline are listed and summarised for the ITT analysis set population by treatment group and for all patients combined as defined by the current relevant TFL standards.

Summaries are produced that present the number and percentage of patients on their:

- Histology type
- American Joint Committee on Cancer (AJCC) stage at diagnosis

- Regional lymph nodes
- Stage at study entry
- Baseline PD-L1 (1-49% and ≥50% for treatment arms 6 and 7, and <1%, ≥1%, 1-49% and ≥50% for all other treatment arms)
- Baseline ctDNA (detected, undetected and unevaluable)

Both categorical and continuous data of baseline PD-L1 and ctDNA will be summarised. For treatment arms 6 and 7, local results of PD-L1 should be used. At the end of the study, if retrospective PD-L1 central results are available, a corresponding table based on central results will be presented. For all other treatment arms, central results of PD-L1 should be used for patients who have central results, otherwise local results will be used. In the absence of source data, stratification factors as recorded in the IRT will be used for categorical summaries.

Summary statistics will also be presented for patients' time from original diagnosis to randomisation, number of target and non-target lesions, and sum of target lesions (longest diameter [or short axis for lymph nodes], mm).

A supportive analysis of disease characteristics is based on the mITT.

4.1.7 Medical History and Concomitant Disease

4.1.7.1 Definitions and Derivations

Medical history and relevant surgical history will be coded using the latest version of the Medical Dictionary for Regulatory Activities (MedDRA).

4.1.7.2 Presentation

Medical history and concomitant disease are summarised and may be listed for the ITT analysis set by treatment group, system organ class (SOC) and preferred term (PT). Medical history for patients with confirmed/suspected COVID-19 infection may be summarised and listed similarly.

4.1.8 Prior and Concomitant Medications

4.1.8.1 Definitions and Derivations

For the purpose of inclusion in prior and/or concomitant medication or therapy summaries, incomplete medication or radiotherapy start and stop dates are imputed as detailed in Section 3.3.6.

Prior medications, concomitant and post-treatment medications are defined based on imputed start and stop dates as follows:

- Prior medications are those taken prior to study treatment with a stop date prior to the first dose of study treatment.
- Concomitant medications are those with a stop date on or after the first dose date of study treatment and must have started prior to or during treatment so there is at least one day in common with the study treatment.
- Post-treatment medications are those with a start date after the last dose date of study treatment.

4.1.8.2 Presentation

The number and percentage of patients who took prior and concomitant medications will be summarised and may be listed by Anatomical therapeutic chemical (ATC) decode and the generic name/term coded by World Health Organisation (WHO) Drug Dictionary.

4.1.9 Study Drug Compliance

4.1.9.1 Definitions and Derivations

Not applicable

4.1.9.2 Presentation

Not applicable

4.2 Endpoint Analyses

This section covers details related to the endpoint analyses such as primary, secondary, other endpoints including sensitivity and supportive analyses.

Efficacy assessments such as pCR and mPR will be performed and determined by a central blinded independent pathological review (BIPR), while other efficacy assessments (EFS, DFS, ORR, and OS) are conducted by Investigator. The EFS event of PD discovered during surgery will be determined by the investigator.

Efficacy analyses of EFS, DFS and ORR are based on programmatic application of Response Evaluation Criteria in Solid Tumours (RECIST) 1.1 (Eisenhauer et al., 2009) to investigator assessed tumour measurements. Programmatic derivation guidance used for the application of RECIST 1.1 are provided in Appendix A, which is used to determine disease response.

RECIST data, including visit-level overall response is listed. Additional listing(s) are produced for pCR and mPR data.

All efficacy analyses are presented by planned treatment group.

4.2.1 Primary Endpoint: pCR

The primary efficacy endpoint is pCR.

4.2.1.1 Definition (pCR)

Pathological complete response is defined as the proportion of patients who have 0% residual viable tumour cells within all resected tissue (including primary lung lesion and lymph nodes) following neoadjuvant treatment as assessed by central BIPR laboratory. Central pathology assessment of pCR will be performed according to the recommended methods and definitions described by IASLC 2020 (Travis et al., 2020). Samples may also be evaluated by a different method (Cottrell et al., 2018) if deemed appropriate by the Sponsor.

4.2.1.2 Derivations (pCR)

The measure of interest is the proportion of patients with 0% residual viable tumour cells within all resected tissue as assessed by the central blinded pathologist.

4.2.1.3 Handling of Dropouts and Missing Data (pCR)

Patients who are not evaluable per central pathology assessment (this includes patients with R2 margins) or who do not have a surgical specimen will be considered as non-pCR (eg, pathology assessments captured as "non-evaluable" or "missing", as appropriate).

4.2.1.4 Primary Analysis of Primary Endpoint (pCR)

Pathological complete response will be presented by the number and percentage of patients with pCR including 95% and 80% Clopper-Pearson (exact) (Clopper-Pearson et al., 1934) CIs for each arm using the ITT analysis set. CCI [redacted]

4.2.1.5 Sensitivity Analyses of the Primary Endpoint (pCR)

Sensitivity analysis may be performed using the method of Cottrell mentioned in section 4.2.1.1.

4.2.1.6 Supplementary Analyses of the Primary Endpoint (pCR)

A supportive analysis of pCR is based on the mITT. A waterfall plot of pathological regression in primary tumour based on independent pathological review may be presented.

4.2.1.7 Subgroup Analyses (pCR)

The pCR data will be analysed by treatment group using the exploratory mITT analysis set.

The pCR data may be analysed by treatment group and baseline ctDNA (detected, undetected and unevaluable), ctDNA clearance status, baseline PD-L1 expression (<1%, ≥1%, 1-49% and ≥50%), histology (adenocarcinoma, squamous cells tumours, other), and stage (II, IIIA and IIIB). Baseline PD-L1 expression <1% will not be applicable to arms 6 and 7.

4.2.2 Secondary Endpoint: EFS

EFS is a secondary efficacy endpoint.

4.2.2.1 Definition (EFS)

Event-free survival is defined as the time from randomisation to the first of the following: a) documented local or distant recurrence as determined by investigator using RECIST 1.1 assessment; b) death due to any cause (event date is the date of death); c) PD that precludes surgery (event date is date of this determination) or PD discovered and reported by the Investigator upon attempting surgery that prevents completion of surgery (event date is the date of the first attempt at surgery).

Patients with R1 and/or R2 margins are not considered to represent PD and will remain in EFS follow-up, although, in the case of R2, they will not be eligible for adjuvant therapy and should be treated in accordance with local standard of care. Pathological confirmation from biopsied lesions, if performed according to Investigator's judgment and local practice, will also be taken into consideration (as applicable). All new lesions should be biopsied where possible. A new primary malignancy confirmed by pathology is not considered an EFS event, but if not confirmed by pathology, a new equivocal lesion will need to be confirmed at a following visit to be converted to a new unequivocal lesion and thus be considered as an EFS event. The date of EFS event will then be attributed to the first visit with new equivocal lesion (later converted to unequivocal).

4.2.2.2 Derivations (EFS)

EFS (months) = (date of EFS event (progression/death) or censoring – date of randomisation + 1) / (365.25/12)

Patients who have not experienced an event or died at the time of analysis are censored at the time of the latest date of assessment from their last disease assessment. For the purpose of EFS, the date of determination of whether or not to proceed to surgery as well as a decision to continue surgery are also considered disease assessments. However, if the patient has an event or dies immediately after two or more consecutive missed visits, the patient is censored at the time of the latest disease assessment prior to the two missed visits. Note: a NE visit is not considered as a missed visit.

If a patient has no disease assessments post-baseline, or does not have any baseline data, they will be censored at baseline, unless they die or progress (either RECIST 1.1 progression or a progression which precludes surgery, or with progression as the reason for surgery not being completed) in the period prior to surgery (Expected date of surgery is 40 days after the last neoadjuvant dose, or day 104 for subjects who did not receive neoadjuvant treatment), in which case they will have an EFS event on the date of death/progression.

Definition of two missed RECIST visits after surgery (calculated from the date of surgery) are defined as follows:

For subjects without a surgery date, an expected surgery date will be imputed as 40 days after the last neoadjuvant dose or day 104 for subjects who did not receive neoadjuvant treatment. For subjects with surgery performed outside the study, the surgery date will be used where available, otherwise the expected surgery date will be imputed as detailed above.

Given the scheduled visit assessment scheme (i.e. 12-weekly for the first 48 weeks after surgery/pre-planned surgery for no surgery performed, 24-weekly until week 192 and then 48-weekly thereafter) the definition of 2 missed visits changes.

For example, if the previous RECIST assessment is from day 50 after surgery to day 245 (i.e. week 7 to week 35) then two missing visits equates to 26 weeks since the previous RECIST assessment, allowing for early and late visits (i.e. 2 x 12 weeks + 1 week for an early assessment + 1 week for a late assessment = 26 weeks).

If the two missed visits occur over the period when the scheduled frequency of RECIST assessments changes from 12-weekly to 24-weekly this equates to 39 weeks (i.e. take the average of 12 and 24 weeks which gives 18 weeks and then apply same rationale, hence 2 x 18 weeks + 1 week for an early assessment + 2 week for a late assessment = 39 weeks). The time period for the previous RECIST assessment is from days 245 to 329 after surgery (i.e. week 35 to week 47).

Table 3 Two missed visits rule for EFS for schedule changes

Scheduled Assessment	Previous RECIST assessment	Two missed RECIST visits window
No disease assessment after surgery, or assessment prior to Day 21	(<Day 21)	13 weeks (91 days) after surgery*
Post-surgery assessment	Week 5±2 weeks<br>(Day 21 – 49)	22 Weeks (154 days)
Q12W±1 week up to Week 48	Week 7 – Week 35 after surgery (>Day 49 – Day 245)	2 x 12 weeks + 2 weeks = 26 weeks (182 days)
	Week 35 – Week 47<br>(>Day 245 – Day 329) (change period from Q12W to Q24W)	2 x [(12 weeks+24 weeks)/2] + 1 week for an early assessment +2 week for a late assessment= 39 weeks (273 days)
Q24W±2 weeks up to week 192	Week 47 – Week 70<br>(Day 330 – 490)	2 x 24 weeks + 1 week for an early assessment +2 week for a late assessment = 51 weeks (357 days)
	Week 70 – Week 166<br><br>(Day 491 – 1162)	2 x 24 weeks + 4 weeks = 52 weeks (364 days)
	Week 166 – Week 190<br><br>(Day 1163 – 1330) (change period from Q24W to Q48W)	2 x [(24 weeks+48 weeks)/2] + 2 weeks for an early assessment +2 weeks for a late assessment= 76 weeks (532 days)
Q48W±2 weeks thereafter	>Day 1330 onwards	2 x 48 weeks + 4 weeks=100 weeks (700 days)

* Window, in this case only, is measured from date (or planned date) of surgery, as this is the first scan post-surgery.

A summary of censoring rules and the date of PD/death or censoring after surgical resection are given in Table 4. Note that censoring overrides event in certain specified cases.

Table 4 Summary of Censoring Rules for EFS after Surgical Resection/Pre-planned surgery

Situation	Date of PD/Death or Censoring	EFS Outcome
Progressive disease (PD) or death in the absence of progression	Date of earliest documentation of PD or date of death in the absence of progression	Event
No tumour assessment after surgery/pre-planned surgery AND death prior to second scheduled post-baseline disease assessment	Date of death	Event
No tumour assessment after surgery/pre-planned surgery AND no death prior to second scheduled post-baseline disease assessment	Date of surgery/pre-planned surgery for no surgery.	Censored
PD or death (in the absence of progression) immediately after ≥ 2 consecutive missed disease assessments as per the protocol specified assessment schedule	Last event-free disease assessment prior to missed assessments	Censored
On-going with neither PD nor death at the time of analysis or lost to follow-up or withdrawn consent	Date of last disease assessment	Censored

PD = progressive disease; EFS = Event-free survival

The EFS time for RECIST scans is always derived based on scan/assessment dates, not visit dates.

RECIST assessments/scans contributing towards a particular visit may be performed on different dates. The following rules are applied:

- The date of progression is determined based on the earliest of the dates of the component that triggered the progression.
- When censoring a patient for EFS the patient is censored at the latest of the dates contributing to a particular overall visit assessment.

Note: for TLs only the latest scan date is recorded out of all scans performed at that assessment for the TLs and similarly for NTLs only the latest scan date is recorded out of all scans performed at that assessment for the NTLs.

The proportion of patients alive and event free at 12 and 24 months (AEF-12/24 [Alive and Event Free] or EFS-12/24) is defined as the Kaplan-Meier estimate of EFS (per RECIST 1.1 as assessed by the site investigator) at 12/24 months.

The duration of follow-up is defined from randomisation to date of censoring (date last known to have not progressed) in censored (not progressed) patients only.

4.2.2.3 Handling of Dropouts and Missing Data (EFS)

Please see section 4.2.2.2.

4.2.2.4 Primary Analysis of Secondary Endpoint (EFS)

The main analysis of EFS is based on the ITT. The number and percentage of patients experiencing a EFS event broken down by type of event/censoring and Kaplan-Meier plots of EFS are presented by treatment group. The median EFS and its two-sided 95% CI are estimated using the Kaplan-Meier method.

The treatment status at EFS event of patients at the time of analysis are summarised. This includes the number (%) of patients who were on treatment at the time of event, the number (%) of patients who discontinued study treatment prior to event, the number (%) of patients who have no event and were on treatment or discontinued treatment.

A summary of the duration of follow-up for EFS is included using median (range). This is presented for censored patients (including all types of EFS censoring).

The proportion of patients alive and event free at 12 and 24 months (landmark analysis) and associated two-sided 95% CI are estimated using the Kaplan-Meier method.

4.2.2.5 Sensitivity Analyses of the Secondary Endpoint (EFS)

Sensitivity analysis may be performed by censoring any patient who had an EFS event due to death where: a) primary/secondary cause of death was due to Confirmed/Suspected COVID-19 Infection, or b) a Confirmed/Suspected COVID-19 infection reported as a fatal AE, at their last assessment prior to their Confirmed/Suspected COVID infection death date.

4.2.2.6 Supplementary Analyses of the Secondary Endpoint (EFS)

A supportive analysis of EFS is based on the mITT. Supplementary analysis of EFS data may be performed by summarising the results of R0, R1 and R2 resection separately.

4.2.2.7 Subgroup Analyses (EFS)

The EFS data may be analysed by treatment group and baseline ctDNA (detected, undetected and unevaluable), ctDNA clearance status, baseline PD-L1 expression (<1%, ≥1%, 1-49% and ≥50%), histology (adenocarcinoma, squamous cells tumours, other), and stage (II, IIIA and IIIB).

4.2.3 Secondary Endpoint: DFS

DFS is a secondary endpoint.

4.2.3.1 Definition (DFS)

Disease-free survival will only be evaluated for patients who had surgical resection following the neoadjuvant period, who have R0/R1 margins, and whose first post-surgical RECIST 1.1 scan shows no evaluable disease (defined as no post-surgery R2 margins and no RECIST evidence of disease). Disease-free survival is defined as the time from the date of surgery until the first date of disease recurrence as determined by Investigator using RECIST 1.1 assessments (local or distant), or date of death due to any cause, whichever occurs first. Pathological confirmation from biopsied lesions, will also be taken into consideration (as applicable). A new primary malignancy, confirmed by pathology, is not considered a DFS event.

Patients who did not have an event at the time of analysis will be censored at the latest date of disease assessment after the surgical resection date.

However, if any of these events occur after 2 or more consecutive missed visits after the first post-surgery RECIST 1.1 scan then the patient will be censored at the time of latest disease assessment prior to the 2 missed visits. If a patient dies between the first post-surgical RECIST scan and the next scheduled RECIST scan (12 weeks relative to surgery date), this will still be considered as an event.

4.2.3.2 Derivations (DFS)

DFS (months) = (date of DFS event (recurrence/death) or censoring – date of surgery + 1) / (365.25/12)

For two missed visit rules please see section 4.2.2.2 after post-surgery. The censoring rules for DFS is defined in Table 5.

Table 5 Censoring rules used in the calculation of DFS

Situation	Date of PD/Death or Censoring	DFS outcome
Documented recurrence (local or metastatic) during the study	Date of the 1st assessment of the series of tests that determined recurrence	Event[a]
Death during the study before recurrence	Date of death	Event
Death or recurrence immediately after two or more missed scheduled assessments	Date of last tumour assessment prior to missed visits as defined in section 4.2.2.2 (post-surgery)	Censored
Patients without recurrence as of data cut-off	Date of last tumour assessment	Censored

[a] Earliest date among the dates used in calculating the disease-free survival.

4.2.3.3 Handling of Dropouts and Missing Data (DFS)

Please see section 4.2.3.2

4.2.3.4 Primary Analysis of Secondary Endpoint (DFS)

The main analysis of DFS is based on the resected analysis set. The number and percentage of patients experiencing a DFS event broken down by type of event/censoring and Kaplan-Meier plots of DFS are presented by treatment group. The median DFS and its two-sided 95% CI are estimated using the Kaplan-Meier method.

The treatment status at DFS event of patients at the time of analysis are summarised. This includes the number (%) of patients who were on treatment at the time of event, the number (%) of patients who discontinued study treatment prior to event, the number (%) of patients who have no event and were on treatment or discontinued treatment.

A summary of the duration of follow-up for DFS is included using median (range). This is presented for censored patients (including all types of DFS censoring).

The proportion of patients alive and disease free at 12 and 24 months (landmark analysis) and associated two-sided 95% CI are estimated using the Kaplan-Meier method.

4.2.3.5 Sensitivity Analyses of the Secondary Endpoint (DFS)

Sensitivity analysis may be performed by censoring any patient who had a DFS event due to death where: a) primary/secondary cause of death was due to Confirmed/Suspected COVID-19 Infection, or b) a Confirmed/Suspected COVID-19 infection reported as a fatal AE, at their last assessment prior to their Confirmed/Suspected COVID infection death date.

4.2.3.6 Supplementary Analyses of the Secondary Endpoint (DFS)

Supplementary analysis of DFS data may be performed by summarising the results of R0 and R1 resection separately.

4.2.3.7 Subgroup Analyses (DFS)

The DFS data may be analysed by treatment group and baseline ctDNA (detected, undetected and unevaluable), ctDNA clearance status, baseline PD-L1 expression (<1%, ≥1%, 1-49% and ≥50%), histology (adenocarcinoma, squamous cells tumours, other), and stage (II, IIIA and IIIB).

4.2.4 Secondary Endpoint: Feasibility to Surgery

Feasibility to surgery is a secondary endpoint.

4.2.4.1 Definition (Feasibility to Surgery)

Feasibility to surgery is defined as having the planned surgical resection within 40 days from the last dose of neoadjuvant study interventions administration. The measure of interest is the proportion of patients that have intended surgery within 40 days from the last neoadjuvant study interventions administered. Patients who do not intend to have surgery within 40 days of last dose of neoadjuvant study interventions will be considered as not feasible for surgery.

4.2.4.2 Derivations (Feasibility to Surgery)

See Section 4.2.4.1

4.2.4.3 Handling of Dropouts and Missing Data (Feasibility to Surgery)

Please see section 4.2.4.1

4.2.4.4 Primary Analysis of Secondary Endpoint (Feasibility to Surgery)

Summaries of number and percentage of patients having surgical resection within 40 days from the last neoadjuvant study interventions administered will be presented by treatment group using the safety set.

4.2.4.5 Sensitivity Analyses of the Secondary Endpoint (Feasibility to Surgery)

No sensitivity analysis is planned for feasibility to surgery.

4.2.4.6 Supplementary Analyses of the Secondary Endpoint (Feasibility to Surgery)

A summary of surgical details will be produced for the safety set and will include:

- Number of subjects who underwent surgery
- Number of subjects who completed surgery
- Frequency of surgery type/procedure performed
- Summary statistics for days from last IP administration (derived using date of last dose of IP and date of surgery)
- Frequency of R0, R1, R2
- Time from surgery to first dose of adjuvant treatment (days).

A summary of surgical complications will be presented for the safety set. Clavien-Dindo assessment will be utilised for grading post-operative complications (See CSP Appendix I for more details).

4.2.4.7 Subgroup Analyses (Feasibility to Surgery)

No subgroup analysis is planned for feasibility to surgery.

4.2.5 Secondary Endpoint: mPR

mPR is a secondary endpoint.

4.2.5.1 Definition (mPR)

Major pathological response rate is defined as the proportion of patients with 10% or less residual viable tumour tissue in lung primary tumour after neoadjuvant treatment at the time of resection as assessed per central BIPR laboratory. Central pathology assessment of mPR will be performed according to the recommended methods and definitions described by IASLC 2020 (Travis et al., 2020). Samples may also be evaluated by a different method (Cottrell et al., 2018) if deemed appropriate by the Sponsor. Estimated % residual viable tumour cells will be recorded. This will be used to programmatically assign a response of Yes if the value is 10% or less, or No if the value is above 10%. Patients who are not evaluable per central pathology assessment (including patients with R2 margins) or who do not have a surgical specimen will be considered as having non-mPR (eg, response captured as "non-evaluable" or "missing" as appropriate).

4.2.5.2 Derivations (mPR)

Please see section 4.2.5.1

4.2.5.3 Handling of Dropouts and Missing Data (mPR)

Please see section 4.2.5.1

4.2.5.4 Primary Analysis of Secondary Endpoint (mPR)

Major pathological response will be presented by the number and percentage of patients with mPR including 95% and 80% Clopper-Pearson (exact) CIs for each treatment using the ITT analysis set. CCI [redacted]

4.2.5.5 Sensitivity Analyses of the Secondary Endpoint (mPR)

Sensitivity analysis may be performed using the method of Cottrell mentioned in section 4.2.1.1

4.2.5.6 Supplementary Analyses of the Secondary Endpoint (mPR)

A supportive analysis of mPR is based on the mITT. Supplementary analysis of mPR data may be performed by summarising the results of R0, R1 and R2 resection separately.

A waterfall plot of pathological regression in primary tumour based on independent pathological review may be presented.

4.2.5.7 Subgroup Analyses (mPR)

The mPR data will be analysed by treatment group using the exploratory mITT analysis set.

The mPR data may be analysed by treatment group and baseline ctDNA (detected, undetected and unevaluable), ctDNA clearance status, baseline PD-L1 expression (<1%, ≥1%, 1-49% and ≥50%), histology (adenocarcinoma, squamous cells tumours, other), and stage (II, IIIA and IIIB).

4.2.6 Secondary Endpoint: ORR

ORR is a secondary endpoint.

4.2.6.1 Definition (ORR)

Objective response rate is defined as the proportion of patients achieving either CR or PR, prior to surgery, as assessed by the Investigator.

Objective Response (OR) is defined as a programmatically derived, best overall response of CR or PR that occurs prior to the initiation of subsequent anticancer treatment and prior to surgery. Objective Response Rate (ORR) is defined as the percentage of patients with objective response.

Best Overall Response (BOR) is calculated based on the overall visit responses from each RECIST assessment, described in Appendix A. It is the best response a subject has had following randomisation up until surgery, or the last evaluable assessment up to and including RECIST progression in the absence of surgery, but prior to starting any subsequent cancer therapy (excluding radiotherapy).

Data obtained up until surgery, or last evaluable assessment in the absence of surgery will be included in the assessment of ORR, regardless of whether the patient withdraws from therapy. Patients who discontinue treatment without a response or surgery, receive subsequent anti-cancer therapy prior to surgery and then respond will not be included as responders in the ORR.

A confirmed response of CR/PR means that a response of CR/PR is recorded at 1 visit and confirmed by repeat imaging not less than 4 weeks after the visit when the response was first observed with no evidence of progression between the initial and CR/PR confirmation visit. For determination of a best response of SD, the earliest of the dates contributing towards a particular overall visit assessment is used. SD should be recorded at least 6 weeks from randomisation.

In the case where a patient has two non-consecutive visit responses of PR, then, as long as the time between the 2 visits of PR is greater than 4 weeks and there is no PD between the PR visits, the patient is defined as a responder. Similarly, if a patient has visit responses of CR, NE, CR, then, as long as the time between the 2 visits of CR is greater than 4 weeks, then a best response of CR is assigned.

Both unconfirmed and confirmed ORR will be reported, considering there are only 2 post-baseline scheduled tumour assessments prior to surgery.

4.2.6.2 Derivations (ORR)

Please see section 4.2.6.1

4.2.6.3 Handling of Dropouts and Missing Data (ORR)

Please see section 4.2.6.1

4.2.6.4 Primary Analysis of Secondary Endpoint (ORR)

Summaries are produced that present the number and percentage of patients with a CR or PR as best overall tumour response. Confirmed and unconfirmed ORR will be calculated and presented as well. The ORR is presented with a two-sided 95% CI using the Clopper-Pearson (exact probability) method. CCI [redacted]

Patients that have missing overall response assessments at all visits are considered as non-responders, and are therefore counted in the denominator of ORR. The main analysis of ORR is based on the Response evaluable set.

4.2.6.5 Sensitivity Analyses of the Secondary Endpoint (ORR)

No sensitivity analysis is planned for ORR.

4.2.6.6 Supplementary Analyses of the Secondary Endpoint (ORR)

A supportive analysis of ORR is based on the mITT. Supplementary analysis may be conducted by calculating the percentage (and its 95% CI) of patients who achieve either objective response (CR/PR) or pathological response (pCR/mPR), based on the mITT set.

Supplementary analysis of ORR data may be performed by summarising the results of R0, R1 and R2 resection separately.

4.2.6.7 Subgroup Analyses (ORR)

The ORR data may be analysed by treatment group and baseline ctDNA (detected, undetected and unevaluable), ctDNA clearance status, baseline PD-L1 expression (<1%, ≥1%, 1-49% and ≥50%), histology (adenocarcinoma, squamous cells tumours, other), and stage (II, IIIA and IIIB).

4.2.7 Secondary Endpoint: OS

OS is a secondary endpoint.

4.2.7.1 Definition (OS)

Overall survival is defined as the time from the date of randomisation until death due to any cause regardless of whether the patient withdraws from study therapy or receives another anti-cancer therapy.

4.2.7.2 Derivations (OS)

OS (months) = (date of death or censoring – date of randomisation + 1) / (365.25/12)

Any patient not known to have died at the time of analysis is censored based on the last recorded date on which the patient was known to be alive.

Note: Survival calls are made in the week following the date of data cut-off (DCO) for the analysis, and if patients are confirmed to be alive or if the death date is post the DCO date these patients are censored at the date of DCO. The status of ongoing, withdrawn (from the study) and "lost to follow-up" patients at the time of the final OS analysis should be obtained by the site personnel by checking the patient's notes, hospital records, contacting the patient's general practitioner and checking publicly-available death registries. In the event that the patient has actively withdrawn consent to the processing of their personal data, the vital status of the patient can be obtained by site personnel from publicly available resources where it is possible to do so under applicable local laws.

Note: For any OS analysis performed (prior to the final OS analysis), in the absence of survival calls being made, it may be necessary to use all relevant CRF fields to determine the last recorded date on which the patient was known to be alive for those patients still on treatment (since the *SURVIVE* module is only completed for patients off treatment if a survival sweep is not performed). The last date for each individual patient is defined as the latest among the following dates recorded on the CRFs:

- AE start, stop and change in severity dates
- Admission and discharge dates of hospitalisation
- Study treatment date
- End of treatment date
- Concomitant medication start and stop dates
- Laboratory test dates
- Date of vital signs
- Disease assessment dates on RECIST CRF
- Start and stop dates of alternative anticancer treatment
- Date last known alive on survival status CRF

• End of study date

The proportion of patients alive at 12/24 months (OS-12/24) is defined as the Kaplan-Meier estimate of OS at 12/24 months.

Duration of follow-up for OS of all patients is defined as follows:
Duration of follow-up for OS (months) = (date of death or censoring (date last known to be alive) – date of randomisation + 1) / (365.25/12).

4.2.7.3 Handling of Dropouts and Missing Data (OS)

See section 3.3.6.

4.2.7.4 Primary Analysis of Secondary Endpoint (OS)

The analysis of OS is based on the ITT analysis set. The number and percentage of patients experiencing an OS event and Kaplan-Meier plots of OS will be presented. The median OS and two-sided 95% CI are estimated using the Kaplan-Meier method. Summaries on the number and percentage of patients who have died, those still in survival follow-up and those lost to follow-up and those who have withdrawn consent will be provided along with the median OS for each treatment.

The proportion of patients alive at 12 and 24 months (landmark analysis) and associated two-sided 95% CI are estimated using the Kaplan-Meier method.

A summary of the duration of follow-up for OS is included using median (range). This is presented separately for censored and non-censored patients.

4.2.7.5 Sensitivity Analyses of the Secondary Endpoint (OS)

Sensitivity analysis may be performed by censoring any patient who had a death where: a) primary/secondary cause of death was due to Confirmed/Suspected COVID-19 Infection, or b) a Confirmed/Suspected COVID-19 infection reported as a fatal AE, at their last assessment prior to their Confirmed/Suspected COVID infection death date.

4.2.7.6 Supplementary Analysis of the Secondary Endpoint (OS)

A supportive analysis of OS is based on the mITT. Supplementary analysis of OS data may be performed by summarising the results of R0, R1 and R2 resection separately.

4.2.7.7 Subgroup Analyses (OS)

The OS data may be analysed by treatment group and baseline ctDNA (detected, undetected and unevaluable), ctDNA clearance status, baseline PD-L1 expression (<1%, ≥1%, 1-49% and ≥50%), histology (adenocarcinoma, squamous cells tumours, other), and stage (II, IIIA and IIIB).

4.2.8 Secondary Endpoint: ctDNA

ctDNA clearance on-treatment prior to surgery is a secondary endpoint for this study.

4.2.8.1 Definition (ctDNA)

Baseline ctDNA is defined as two groups: Detected and Undetected. Only patients with detected ctDNA at baseline are evaluable for molecular response. Complete molecular response or "ctDNA clearance" is defined as 100% reduction in ctDNA from baseline to post-baseline prior to surgery. Partial molecular response is defined as at least 50% reduction in ctDNA from baseline to post-baseline prior to surgery. Molecular responders are patients who achieve at least partial molecular response.

4.2.8.2 Derivations (ctDNA)

Please see section 4.2.8.1

4.2.8.3 Handling of Dropouts and Missing Data (ctDNA)

No imputation will be performed for ctDNA.

4.2.8.4 Primary Analysis of Secondary Endpoint (ctDNA)

The number and percentages of patients who achieve ctDNA clearance prior to surgery will be summarised across each treatment. CCI [redacted]

4.2.8.5 Sensitivity Analyses of the Secondary Endpoint (ctDNA)

No sensitivity analysis is planned for ctDNA.

4.2.8.6 Supplementary Analysis (ctDNA)

The association between the ctDNA clearance and clinical endpoints (pCR, mPR, EFS and DFS etc.) will also be assessed by cross-tabulations and Kaplan-Meier analysis for EFS and DFS if numbers allow. This association may be analyzed based ctDNA clearance status alone or in combination with pCR/mPR status (e.g. pCR+/ctDNA+ vs pCR-/ctDNA+ vs pCR-/ctDNA-, and mPR+/ctDNA+ vs mPR-/ctDNA+ vs mPR-/ctDNA-).

The association between ctDNA molecular responses and histology, tumor size, smoking history and stage of disease may also be explored.

Supplementary analysis of ctDNA data may be performed by summarising the results of R0, R1 and R2 resection separately.

4.2.8.7 Subgroup Analyses (ctDNA)

No subgroup analysis is planned for ctDNA.

4.2.9 Secondary Endpoint: PK

4.2.9.1 Definition (PK)

This section covers details related to pharmacokinetics endpoints and analyses.

4.2.9.2 Derivations (PK)

The gmean is calculated as exp(μ), where μ is the mean of the data on the natural log scale.

The gSD is calculated as exp(σ), where σ is the standard deviation of the data on the natural log scale.

The gCV is calculated as 100 x sqrt[exp(s^2)-1], where s is the Std Dev of the data on the natural log scale.

The gmean ± gSD (gmean-gSD and gmean+gSD) are calculated as exp[μ ± s].

4.2.9.3 Handling of Dropouts and Missing Data (PK)

Handling of Non-Quantifiable Concentrations

Individual concentrations below the LLOQ of the bioanalytical assay are reported as NQ in the listings with the LLOQ defined in the footnotes of the relevant TFLs. Individual plasma concentrations that are Not Reportable are reported as NR and those that are missing are reported as NS (No Sample) in the listings. Plasma concentrations that are NQ, NR or NS are handled as follows for the provision of descriptive statistics:

- Any values reported as NR or NS are excluded from the summary tables and corresponding figures.
- At a time point where less than or equal to 50% of the concentration values are NQ, all NQ values are set to the LLOQ, and all descriptive statistics are calculated accordingly.
- At a time point where more than 50% (but not all) of the values are NQ, the gmean, gmean ± gSD and gCV% are set to NC. The maximum value is reported from the individual data, and the minimum and median are set to NQ.
- If all concentrations are NQ at a time point, no descriptive statistics are calculated for that time point. The gmean, minimum, median and maximum are reported as NQ and the gCV% and gmean ± gSD as NC.
- The number of values below LLOQ (n < LLOQ) are reported for each time point together with the total number of collected values (n).

Three observations > LLOQ are required as a minimum for a plasma concentration or PK parameter (e.g. Cmax, Cmin, Clast) to be summarised. Two observations > LLOQ are presented as minimum and maximum with the other summary statistics as NC.

4.2.9.4 Primary Analysis of Secondary Endpoint (PK)

Precision and Rounding Rules for Pharmacokinetic Data

PK concentration data

PK concentration data listings present to the same number of significant figures as the data received from the bioanalytical laboratory (usually but not always to 3 significant figures) and against the same units as received.

PK concentration descriptive statistics present 4 significant figures with the exception of the min and max which present 3 significant figures and n and n<LLOQ which present as integers.

For each analyte, serum concentrations for each scheduled time-point are summarised by analyte, cohort, Visit and scheduled timepoint using appropriate descriptive statistics.

The following descriptive statistics are presented for serum concentrations:

- n
- n below LLOQ
- geometric mean (gmean)
- geometric standard deviation (gSD)
- gmean±gSD
- geometric coefficient of variance (%) (gCV)
- arithmetic mean of non log-transformed data (mean)
- standard deviation of non log-transformed data (Std Dev)
- coefficient of variance (%) (CV)
- median
- minimum (min)
- maximum (max)

Protocol scheduled times are used to present the PK concentration summary tables and corresponding gmean concentration-time figures.

Graphical presentation of PK data

All mean (arithmetic mean and/or gmean) plots or combined plots showing all patients by treatment are based on the PK analysis set.

For consistency, the serum concentration values used in the mean (arithmetic mean and/or gmean) data graphs are those given in the descriptive statistics summary table for each time point.

For gmean concentration-time plots, NQ values are handled as described for the descriptive statistics; if the geometric mean is NQ, the value plotted is zero for linear plots and missing for semi-logarithmic plots. Any gmean±gSD error bar values that are negative are truncated at zero on linear concentration-time plots and omitted from semi-logarithmic plots.

For individual plots, serum concentrations which are NQ prior to the first quantifiable concentration are set to a value of zero (linear plots only). After the first quantifiable concentration, any NQ plasma concentrations are regarded as missing.

4.2.9.5 Additional Analyses of the Secondary Endpoint (Pharmacokinetics)

All the analysis of PK are mentioned in Section 4.2.9.4

4.2.9.6 Subgroup Analyses (PK)

No subgroup analysis is planned for PK data.

4.2.10 Secondary Endpoint: Immunogenicity

4.2.10.1 Definition (Immunogenicity)

Blood samples for determination of ADA for IP(s) in serum will be taken according to the SoA in the CSP and assayed by bioanalytical test sites operated by or on behalf of AstraZeneca, using an appropriately validated bioanalytical method. Full details of the methods used will be described in a separate report.

ADA samples may also be further tested for characterisation of the ADA response.

4.2.10.2 Presentations (Immunogenicity)

ADA table and listings will be produced according to all AZ corporate and/or Oncology TA standard TFL shells. Immunogenicity results will be listed for each patient and summarised for the immunogenicity analysis population. ADA results from each sample will be reported as either positive or negative. If the sample is positive, the ADA titer will be reported as well. Number and percentage of patients in the following categories will be provided.

- ADA positive post-baseline and positive at baseline.
- ADA not detected post-baseline and positive at baseline.
- Anti-drug antibody (ADA) positive at baseline and/or post-baseline visits. The percentage of these subjects in a population is known as ADA prevalence.

- Treatment-induced ADA positive (positive post-baseline and not detected at baseline).
- Treatment-boosted, defined as baseline positive ADA titre that was boosted to a 4-fold or higher level following drug administration.
- Treatment-emergent ADA positive (either treatment-induced ADA positive or treatment-boosted ADA positive). The percentage of these subjects in a population is known as ADA incidence.
- Non-treatment-emergent ADA positive, defined as being ADA positive but not fulfilling the conditions for treatment-emergent ADA positive.
- Treatment-emergent persistently positive ADA, defined as being TE ADA+ and having at least 2 post-baseline ADA-positive measurements with at least 16 weeks (112 days) between the first and last positive measurements.
- Treatment-emergent transiently positive ADA, defined as being TE ADA+ and having at least 1 post-baseline ADA-positive measurement and not fulfilling the conditions for persistently positive.
- Treatment-emergent ADA positive with maximum post-baseline titre ≥ median of maximum post-baseline titres.

A summary will be provided of the number and percentage of patients who develop detectable anti-drug antibodies by ADA categories using the immunogenicity set. Descriptive statistics (minimum, Q1, median, Q3, and maximum) for the maximum post-baseline ADA titers of patients in each ADA category will also be included. Line plots for ADA titers by visit will be provided. The number and percentage of patients who develop detectable anti-drug antibodies by visit will also be provided.

CCI [redacted]

Impact of ADA on PK will be explored by presenting serum concentration descriptive statistics in TE-ADA+, ADA+, and ADA negative. CCI [redacted]. Spaghetti plots of individual concentration data over time profiles of subjects with the same ADA status will be presented.

ADA safety tables will include the number and percentage of patients who had at least 1 AE in any category summarised by ADA status based on categories described below:

- [redacted]
- TE-ADA+, non-TE-ADA+, and ADA-negative for all other treatment arms.

ADA safety tables will also include the number and percentage of patients from all treatment arms who had AE Grade ≥3 or SAE by system organ class and preferred term by ADA status based on the same categories described above.

All valid assay results from patients who received any study drug are included in immunogenicity summaries. Blood samples collected outside of the protocolled window are summarised at the closest nominal time point that does not already have a value.

Immunogenicity results will be listed for all patients in safety set regardless of ADA-evaluable status. Anti-drug antibody titer data will be listed for samples confirmed positive for the presence of anti-drug antibodies. AEs in ADA positive patients by ADA positive category will be listed.

4.2.11 Safety Analyses

The domain safety covers exposure, adverse events, clinical laboratory, vital signs, and electrocardiogram (ECG).

Tables are provided for the safety set, listings are provided for all patients or the safety set depending on the availability of data.

4.2.11.1 Exposure

4.2.11.1.1 Definitions and Derivations

Total Exposure during Neoadjuvant period

Duration of exposure (weeks) will be defined separately for each study intervention and for neoadjuvant and adjuvant period as follows:

- Duration of exposure (weeks) of durvalumab in neoadjuvant period= (earliest of (last date of actual dosing of durvalumab where dose>0 mg in the last cycle+ 20, death, DCO)– first dose date+1)/7
- Duration of exposure (weeks) of oleclumab in neoadjuvant period= (earliest of (last date of actual dosing of oleclumab where dose>0 mg in the last cycle+ 20, death, DCO)– first dose date+1)/7

- Duration of exposure (weeks) of monalizumab in neoadjuvant period= (earliest of (last date of actual dosing of monalizumab where dose>0 mg in the last cycle+ 20, death, DCO)– first dose date+1)/7
- Duration of exposure (weeks) of chemotherapy in neoadjuvant period= (earliest of (last date of actual dosing of chemotherapy where dose>0 mg in the last cycle+ 20, death, DCO)– first dose date+1)/7
- CCI [redacted]
- Duration of exposure (weeks) of Dato-DXd in neoadjuvant period= (earliest of (last date of actual dosing of Dato-DXd where dose>0 mg in the last cycle+ 20, death, DCO)– first dose date+1)/7
- CCI [redacted]
- [redacted]
- Duration of exposure (cycles) is defined as the number of cycles in which at least one portion of investigational agent was administered (i.e. dose > 0 mg). If a cycle is prolonged due to toxicity, this should still be counted as one cycle.

Total Exposure during Adjuvant period

- Duration of exposure (weeks) of durvalumab in adjuvant period= (earliest of (last date of actual dosing of durvalumab where dose>0 mg in the last cycle+ 27, death, DCO)– first dose date+1)/7.
- Duration of exposure (weeks) of oleclumab in adjuvant period= (earliest of (last date of actual dosing of oleclumab where dose>0 mg in the last cycle+ 27, death, DCO)– first dose date+1)/7
- Duration of exposure (weeks) of monalizumab in adjuvant period= (earliest of (last date of actual dosing of monalizumab where dose>0 mg in the last cycle+ 27, death, DCO)– first dose date+1)/7
- CCI [redacted]
- [redacted]
- [redacted]

- Duration of exposure (cycles) is defined as the number of cycles in which at least one portion of investigational agent was administered (i.e. dose > 0 mg). If a cycle is prolonged due to toxicity, this should still be counted as one cycle.

Actual Exposure

Duration of actual exposure (which takes into account dose delay) of durvalumab/oleclumab/monalizumab/chemotherapy/[redacted]Dato-DXd/[redacted]

- Actual exposure = total exposure – total duration of dose delays, where total exposure will be calculated as above, and a dose delay is defined as any length of time (weeks) where the patient has not taken any of the planned dose according to the last dose date.

Neoadjuvant period

Calculation of duration of dose delays (for actual exposure) in the neoadjuvant period:

Since patients will receive durvalumab/oleclumab/monalizumab/[redacted]Dato-DXd/[redacted] via IV infusion q3w, the duration of dose delays will be calculated as follows:

- For all dosing dates which are delayed per protocol: Total duration of dose delays = Sum of (Date of the dose – Date of previous dose – 21 days).

Adjuvant period

For patients receiving durvalumab/oleclumab/monalizumab via IV infusion q4w, the duration of dose delays will be calculated as follows:

- For all dosing dates which are delayed per protocol: Total duration of dose delays = Sum of (Date of the dose – Date of previous dose – 28 days).

[redacted]

- [redacted]

[redacted]

- [redacted]

Number of treatment cycles received

Exposure will also be measured by the number of cycles received. A cycle corresponds to a period of 21 days in the neoadjuvant period and 28 days in the adjuvant period except [redacted] and [redacted] for which a cycle corresponds to [redacted] days. If a cycle is prolonged due to toxicity, this should still be counted as one cycle. A cycle will be counted if treatment is started even if the full dose is not delivered.

Dose Intensity

Dose intensity of IP(s) is addressed by considering relative dose intensity (RDI), where RDI is the percentage of the actual dose delivered relative to the intended dose through to treatment discontinuation. More specifically, RDI is defined as follows:

- RDI = 100% × d/D, where d is the actual cumulative dose delivered up to the actual last day of dosing and D is the intended cumulative dose up to the actual last day of dosing. D is the total dose that would be delivered, if there were no modification to dose or schedule.

Actual cumulative dose = sum over all study drug administration recorded on the study drug exposure form up to min (date of last dose date where dose > 0, date of death, date of DCO).

The percentage of volume left after the infusion will be used to calculate how much of the study drug they received, i.e.

- Volume left = Volume after infusion/Volume before infusion
- Actual dose received = (1 - Volume left) x Total dose

For example, if a patient has 100ml left after the infusion from 250ml before the infusion, then:

Volume left = 100/250=40%

Actual mg of study drug received= (1 - 0.4) x 1500 mg = 900 mg

Intended cumulative dose is calculated by summing the planned individual doses that should have been received up to and including the last day of treatment according to the planned dose and schedule.

The planned dose at each cycle for each study drug will be calculated as:

- Durvalumab: 1500 mg IV Q3W (pre-surgery) or Q4W (post-surgery)
- Oleclumab: 3000 mg IV Q3W (pre-surgery) or Q4W (post-surgery)
- Monalizumab: 1500 mg IV Q3W (pre-surgery) or Q4W (post-surgery)
- [redacted]

  - [redacted]
    - [redacted]
    - [redacted]
- Dato-DXd: 6.0 mg/kg x baseline weight
  Weight at day 1 of each cycle will be used if the percentage change from baseline weight is ≥ ± 10%. Otherwise, baseline weight will be used. After the recalculation, the updated patient's weight will be used as the new baseline weight. If the weight at day 1 of a cycle is not available, the last weight measurement prior to the day 1 of that cycle will be used.
- [redacted]
  - [redacted]

RDI will be calculated for each for the following:

- Neoadjuvant period - durvalumab/oleclumab/monalizumab/[redacted]Dato-DXd/ [redacted]
- Adjuvant period - durvalumab/oleclumab/monalizumab/[redacted]
- Overall - durvalumab/oleclumab/monalizumab/[redacted] /[redacted] calculated as the average between the neoadjuvant and adjuvant RDI. Overall RDI for Dato-DXd is the same as the RDI for the neoadjuvant period because Dato-DXd is administered during the neoadjuvant period only.

4.2.11.1.2 Presentation

The summaries related to study treatment will be produced for the safety set by actual treatment group and will be repeated for each treatment period (neoadjuvant, adjuvant).

Duration of exposure to IP(s) in weeks (and cycles) is summarised by descriptive statistics and by frequency using the safety set for each treatment group. RDI is summarised by descriptive statistics using the safety set for each treatment period (neoadjuvant, adjuvant and overall). Exposure to IP(s) i.e. total amount of study drug received is listed for all patients. Exposure swimmer plot(s) are produced, with a line presented for each patient to display relevant exposure and disposition details.

Dosing deviations for IP(s) will be summarised with reasons for deviations for the following categories: dose delays, omissions, interruptions and reductions (if applicable). Dosing delays are derived based on the scheduled dosing dates using the previous dose given as reference. The number of patients with dosing delays and total dose delays are summarised. In addition, the number of patients who had delay to the first adjuvant cycle

will be summarised with reasons for delay.

4.2.11.2 Adverse Events

4.2.11.2.1 Definitions and Derivations

The Medical Dictionary for Regulatory Activities (MedDRA) (using the latest or current MedDRA version) is used to code the AEs. AEs are graded according to the National Cancer Institute Common Terminology Criteria for AEs (using the CTCAE version referenced in the CSP).

Treatment emergent adverse events (TEAEs) are all AEs which onset or worsen in severity following the first administration of IP within the duration of the treatment period, up to and including 90 days after the last dose of study treatment (or surgery, whichever is later) as per the study safety follow-up period but prior to subsequent cancer therapy (excluding radiotherapy). Worsening in severity is determined by comparison with the pre-treatment CTCAE grade of the AE recorded closest to the start of dosing.

For rules on missing or partial dates, see Section 3.3.6. AEs with a missing start time (or where time is not collected) which occur on the same day as first IP administration are reported as treatment emergent.

When assigning AEs to the relevant phase of the study the following rules apply and any deviations must be agreed by the study team:

- Pre-treatment phase: All AEs with a start date after signing the informed consent form (ICF), prior to the first administration of IP that do not subsequently go on to worsen during the treatment emergent phase.
- Treatment emergent phase: All AEs (starting or worsening) on or after the first dose of study intervention and within 90 days after the last dose of study intervention (or surgery, whichever is later) or up to the day prior to start of subsequent therapy (excluding radiotherapy), whichever comes first. The treatment emergent phase is separated by neoadjuvant, post-surgery, adjuvant period and overall period.
- Off-treatment phase: All AEs starting more than 90 days after last dose of study intervention (or surgery, whichever is later) or once subsequent cancer therapy (excluding radiotherapy) is started, whichever is earlier.

SAEs

A serious adverse event (SAE) is any AE that:

- Results in death
- Is immediately life-threatening
- Requires inpatient hospitalisation or prolongation of existing hospitalisation
- Results in persistent or significant disability/incapacity
- Is a congenital anomaly/birth defect in offspring of the patient
- Is an important medical event that may jeopardise the patient.

AEs of special interest/possible interest

An adverse event of special interest (AESI) is one of scientific and medical interest specific to understanding of the IP and may require close monitoring and rapid communication by the investigator to the sponsor. An AESI may be serious or non-serious. The rapid reporting of AESIs allows ongoing surveillance of these events in order to characterise and understand them in association with the use of study intervention.

AESIs for durvalumab include but are not limited to events with a potential inflammatory or immune-mediated mechanism and which may require more frequent monitoring and/or interventions such as steroids and other immunosuppressants, and/or hormone replacement therapy. These AESIs are being closely monitored in clinical studies with durvalumab monotherapy and combination therapy.

An adverse event of possible interest (AEPI) is defined as an AE that could have a potential inflammatory or immune-mediated pathophysiological basis resulting from the mechanism of action of durvalumab but are more likely to have occurred due to other pathophysiological mechanisms, thus, the likelihood of the event being inflammatory or immune-mediated in nature is not high and/or is most often or usually explained by the other causes. These AEs not routinely arising from an inflammatory or immune-mediated mechanism of action – typically quite general clinical terms that usually present from a multitude of other causes – are classified as AEPIs.

An immune mediated Adverse Event (imAE) is defined as an AE that is associated with drug exposure and is consistent with an immune-mediated mechanism of action and where there is no clear alternate aetiology. The list of AESIs/imAEs for durvalumab is presented in Section 8.3.10.1 of the CSP. There are no specific AESIs for monalizumab. AESIs for oleclumab, [redacted] Dato-DXd, [redacted] and [redacted] are specified in CSP Appendix K7, M7, N7, O7 and P7 respectively.

More information regarding AESIs can be found in the protocol. Other categories may be added, or existing terms may be modified as necessary. An AstraZeneca medically qualified expert after consultation with the Global Patient Safety Physician has reviewed the AEs of interest and identified which higher-level terms and which preferred terms contribute to each AESI. Further reviews may take place prior to CDL to ensure any further terms not already included are captured within the categories. Preferred terms used to identify AESI is listed before CDL.

Other significant adverse events (OAE)

During the evaluation of the AE data, an AstraZeneca medically qualified expert reviews the list of AEs that were not reported as SAEs and AEs leading to discontinuation of study treatment. Based on the expert's judgement, adverse events of particular clinical importance may, after consultation with the Global Safety Physician, be considered other significant adverse events (OAEs) and reported as such in the Clinical Study Report. A similar review of laboratory values, vital signs, ECGs and other safety assessments are performed for identification of other significant adverse events. This review takes place prior to database lock, and any AEs identified are fully documented in meeting minutes. Further review following database lock may result in ad-hoc OAEs being identified, in this case, the OAEs and resulting summaries are fully documented in the CSR.

Examples of these are marked haematological and other laboratory abnormalities, and certain events that lead to intervention (other than those already classified as serious), dose reduction or significant additional treatment.

4.2.11.2.2 Presentation

All AEs are presented based on safety set.

All treatment emergent AEs (TEAEs) are summarised and listed.

TEAEs are counted once for each patient for calculating percentages of patients experiencing TEAE. In addition, if the same TEAE occurs multiple times within a particular patient, the highest severity and level of relationship observed are reported. For tables by MedDRA system organ class (SOC) and MedDRA preferred term (PT), patients with multiple TEAEs are counted once for each SOC/PT.

An overall summary table of the number of patients experiencing each category of adverse event is produced, including but not limited to:

- Any AE
- AEs with CTCAE grade 3 or higher

- AEs leading to discontinuation of each IP and chemotherapy
- AEs leading to interruption of each IP and chemotherapy
- AEs leading to dose omission of each IP and chemotherapy
- AEs leading to dose reduction of Dato-DXd
- SAEs
- SAEs with outcome of death
- AEs causally related to each IP and chemotherapy (as determined by the reporting investigator)
- Grade 3 or higher AEs causally related to any IP and chemotherapy (as determined by the reporting investigator)
- AEs leading to surgery not done
- AEs leading to a delay in surgery (i.e. surgery more than 40 days after the last dose of study treatment in the neoadjuvant period)
- AEs leading to a delay in adjuvant treatment
- AEs leading to adjuvant treatment not given

The number of patients experiencing TEAEs by MedDRA SOC and PT are summarised. Summary information (the number and percent of patients) by SOC and PT will be tabulated for:

- All AEs
- AEs by maximum CTCAE grade
- AEs with CTCAE grade 3 or higher
- AEs leading to discontinuation of each IP and chemotherapy
- AEs leading to interruption of each IP and chemotherapy
- AEs leading to dose omission of each IP and chemotherapy
- AEs leading to dose reduction of Dato-DXd
- SAEs

- SAEs with outcome of death

Summary information (the number and percent of patients) by SOC and PT may be tabulated for:

- All AEs causally related to each IP and chemotherapy (as determined by the reporting investigator)
- All AEs causally related to any IP or chemotherapy (as determined by the reporting investigator)
- All AEs causally related to surgery (as determined by the reporting investigator)
- AEs by maximum reported CTCAE grade on a subset of key group term and preferred term level (as determined by the reporting investigator)
- Immune mediated AEs (as derived programmatically or determined by the reporting investigator, if applicable)

An overall summary table of the number and percentage of patients experiencing each category of adverse event will be produced, as well as an overall summary of the number of events in each category. In addition, a truncated AE table of most common AEs and another table showing most common AEs with CTCAE grade 3 or higher, showing all events that occur in at least 5% of patients overall will be summarised by preferred term, by decreasing frequency. This cut-off may be modified after review of the data. When applying a cut-off (i.e., x %), the raw percentage should be compared to the cut-off, no rounding should be applied first (i.e., an AE with frequency of 4.9% will not appear if a cut-off is 5%).

AEs will be assigned CTCAE grades and summaries of the number and percentage of patients will be provided by maximum reported CTCAE grade, system organ class, preferred term and actual treatment group.

Details of any deaths will be summarised and listed for all patients. AEs leading to death will also be summarised.

The overall AE summary table, and all AEs by system organ class and preferred term will be repeated firstly by period as defined in section 3.3.1 and then repeated for the overall period including AEs observed up until 90 days after last dose of study drugs (or surgery, whichever is later) or up to the day prior to start of subsequent anti-cancer therapy (excluding radiotherapy), whichever comes first.

Additionally, summary information (number and percent of patients) may be presented for AEs related to COVID-19. A listing may be provided for patients with AEs with confirmed/suspected COVID-19. If there are no patients with COVID-19, these tables and listings will not be required.

SAEs

SAEs are summarised as described above for the TEAEs.

Any SAE occurring before study treatment will be included in the data listings but will not be included in the summary tables of AEs.

Any SAE occurring within 90 days of discontinuation of IP (i.e. the last dose of study treatment) will be included in the relevant SAE summaries. Any events in this period that occur after a patient has received further therapy for cancer (following discontinuation of study treatment) will be flagged in the data listings.

AEs of special interest or possible interest

Grouped summary tables of certain MedDRA preferred terms are produced and may also show the individual preferred terms which constitute each AESI grouping. Groupings are based on preferred terms provided by the medical team prior to CDL, and a listing of the preferred terms in each grouping are provided. There is no AESI for monalizumab.

An overall summary of AESI or AEPI may include number and percentage (%) of patients who have:

- Any AESI or AEPI (per preferred terms disclosed as of clinical data lock)
- At least one AESI or AEPI presented by event outcome
- Any AESI or AEPI leading to a delay in surgery (i.e., surgery more than 40 days after the last dose of study treatment in the neoadjuvant period)
- At least one durvalumab AESI or AEPI causally related to durvalumab
- At least one oleclumab AESI causally related to oleclumab
- [redacted]
- At least one Dato-DXd AESI causally related to Dato-DXd
- [redacted]
- [redacted]
- At least one durvalumab AESI or AEPI leading to discontinuation of durvalumab
- At least one oleclumab AESI leading to discontinuation of oleclumab
- [redacted]

- At least one Dato-DXd AESI leading to discontinuation of Dato-DXd
- CCI [redacted]
- CCI [redacted]

Data collected for Interstitial Lung Disease / pneumonitis investigation for patients exposed to Dato-DXd will be listed.

<u>Listings</u>

Key subject information listings will be produced as follows:

- A key subject information listing of SAEs
- A key subject information listing of SAEs with outcome of death
- A key subject information listing of deaths
- A key subject information listing of AEs leading to discontinuation of IP(s)
- A key subject information listing of AESIs

By patient listings will be produced as follows:

- A by patient listing of AEs
- A by patient listing of AEs presenting any events that occur prior to dosing or starting more than 90 days after discontinuing therapy.

<u>Deaths</u>

A summary of deaths will be provided with number and percentage of patients, categorised as:

- Related to disease under investigation,
- AE outcome=death,
- Both related to disease under investigation and with AE outcome=death,
- AE with outcome=death ≥90 days after last treatment dose,
- Deaths ≥90 days after last treatment dose, unrelated to AE or disease under investigation, and
- Patients with unknown reason for death.

The following summary tables of AEs with an outcome of death will be prepared and presented by actual treatment group.

- By SOC and PT
- Causally related to IP(s) by SOC and PT

4.2.11.3 Clinical Laboratory, Blood Sample

4.2.11.3.1 Definitions and Derivations

Laboratory tests are grouped according to chemistry and haematology. Laboratory parameters are assessed at baseline as well as throughout the study. The parameters from Table 7, 8 and 9 of CSP will be presented.

For chemistry and haematology parameters, laboratory abnormalities with toxicity grades according to the NCI CTCAE version 5.0 are derived.

Change from baseline in haematology and clinical chemistry parameters are calculated for each post-dose visit. CTCAE grade is calculated at each visit. Maximum post-baseline CTCAE grades are also calculated. Absolute values are compared to the local laboratory reference range and classified as low (below range), normal (within range or on limits of range) and high (above range). All values classified as high or low are flagged on the listings.

Liver Function Parameters

Patients with elevated post-baseline alanine aminotransferase (ALT), aspartate aminotransferase (AST) or Total Bilirubin that fall into these categories are identified.

Table 6 Liver Function Parameters

Liver Function Parameters	Category
ALT	• ≥3 × – ≤5 × ULN<br>• >5 × – ≤8 × ULN<br>• >8 × – ≤10 × ULN<br>• >10 × – ≤20 × ULN<br>• >20 × ULN
AST	• ≥3 × – ≤5 × ULN<br>• >5 × – ≤8 × ULN<br>• >8 × – ≤10 × ULN,<br>• >10 × – ≤20 × ULN<br>• >20 × ULN
Total bilirubin	• ≥2 × – ≤3 × ULN<br>• >3 × – ≤5 × ULN<br>• >5 × ULN
ALT or AST	• ≥3 × – ≤5 × ULN<br>• >5 × – ≤8 × ULN<br>• >8 × – ≤10 × ULN,<br>• >10 × – ≤20 × ULN<br>• >20 × ULN

Liver Function Parameters	Category
Potential Hy's law	• (AST $\geq 3 \times$ ULN or ALT $\geq 3 \times$ ULN) and (Total Bilirubin $\geq 2\times$ULN)[a]

ULN: upper limit of normal range.

[a] It includes all patients who have ALT or AST≥3xULN and total bilirubin (BILI) ≥2xULN, and in which the elevation in transaminases precede or coincide with (that is, on the same day as) the elevation in BILI.

Assessment of Nephrotoxicity

Creatinine clearance rate (CCr) is calculated using serum creatinine and the Cockcroft-Gault formula to estimate glomerular filtration rate (GFR). Baseline and "worst-case" on treatment C_{Cr} value are categorised for the following categories:

- Normal: ≥ 90 mL/min
- Mild Impairment: $\geq 60 - < 90$ mL/min
- Moderate Impairment: $\geq 30 - < 60$ mL/min
- Severe Impairment: $\geq 15 - < 30$ mL/min
- Kidney Failure: < 15 mL/min

4.2.11.3.2 Presentations

Summaries of results over time and change from baseline will be presented for each laboratory parameter. Shift from baseline to the worst on-treatment value is presented.

Listings are provided for all laboratory results.

Liver Function Parameters

Number and percentage of patients with elevated post-baseline ALT, AST and/or Total Bilirubin are tabulated. Individual patient data where elevated ALT or AST plus total bilirubin fall into the "Potential Hy's law" are summarised and/or listed. Maximum on-treatment ALT and AST versus maximum on-treatment total bilirubin will be presented.

Assessment of Nephrotoxicity

Shift tables from baseline to "worst-case" on treatment C_{Cr} value are provided.

4.2.11.4 Clinical Laboratory, Urinalysis

4.2.11.4.1 Definitions and Derivations

Laboratory parameters are assessed at baseline as well as throughout the study.

Change from baseline in urinalysis parameters are calculated for each post-dose visit. CTCAE grade is calculated at each visit. Maximum post-baseline CTCAE grade is also calculated. Absolute values are compared to the local laboratory reference range and classified as low (below range), normal (within range or on limits of range) and high (above range). All values classified as high or low are flagged in the listings.

4.2.11.4.2 Presentations

Shift from baseline to worst on-treatment results are presented. Urinalysis abnormalities occurring from the start of IP administration to the last assessment on treatment are presented. Listings may be provided for urinalysis.

4.2.11.5 Other Laboratory Evaluations

4.2.11.5.1 Definitions and Derivations

Not applicable

4.2.11.5.2 Presentations

Not applicable

4.2.11.6 Vital Signs

4.2.11.6.1 Definitions and Derivations

Vital signs (BP, pulse, temperature, and respiration rate) will be evaluated according to the SoAs in CSP. Body weight is also recorded at each visit along with vital signs.

Vital signs are assessed at baseline and throughout the study.

4.2.11.6.2 Presentations

Vital signs will be summarised by study visit which may include descriptive statistics for the value of the parameters and the changes from baseline.

4.2.11.7 Electrocardiogram

4.2.11.7.1 Definitions and Derivations

Electrocardiogram (ECG) parameters are assessed at baseline as well as throughout the study. ECG parameters include: QT and QTcF. The QTcF is considered as the primary correction method to assess patient cardiac safety.

The notable ECG interval values while on treatment are:

- Maximum QTcF intervals > 450 milliseconds, >= 470 milliseconds, and > 500 milliseconds.
- Maximum changes from baseline in QTcF > 30, >60, and > 90 milliseconds.

4.2.11.7.2 Presentations

ECG parameters are summarised using descriptive statistics by visit for each treatment and change from baseline in ECG parameters are calculated for each post-dose visit.

The number and percentage of patients having notable ECG interval values while on treatment may be summarised.

4.2.11.8 Other Safety Assessments

No other safety assessments are planned.

4.2.11.8.1 Definitions and Derivations

Not applicable

4.2.11.8.2 Presentations

Not applicable

4.2.12 Other Endpoint: Biomarkers

CCI [redacted]

[redacted]

4.2.12.1 Definition: Biomarkers

This will be discussed in a separate analysis plan.

4.2.12.2 Presentations: Biomarkers

The presentations will be discussed in a separate analysis plan.

4.3 Pharmacodynamic Endpoint(s)

Exploratory analyses pertaining to pharmacodynamic endpoint(s) may be conducted. More details will be specified upon data availability.

4.3.1 Analysis

More details about exploratory analyses of pharmacodynamic endpoint(s) will be specified upon data availability.

4.3.2 Definitions and Derivations

More details about exploratory analyses of pharmacodynamic endpoint(s) will be specified upon data availability.

4.3.3 Presentation

More details about exploratory analyses of pharmacodynamic endpoint(s) will be specified upon data availability.

5 INTERIM ANALYSIS

Continuous Monitoring

[redacted] Each treatment arm will be evaluated separately and independently. [redacted] Enrolment is not paused during the interim analysis before the No-Go decision is made.

[redacted]. For any interim analyses and the final analysis, a pCR response is defined as lack of any viable tumour cells after complete evaluation in the resected lung cancer specimen and all sampled regional lymph nodes as determined by central BIPR and described by IASLC 2020 (Travis et al., 2020). In addition to pCR, mPR, Feasibility to surgery, ORR, PK and ADA may also be evaluated at the interim using the appropriate analysis population as specified in Section 3.2.

The interim conducted at [redacted] patients will be evaluated to determine futility. Based on the Go/No-Go decision framework (Frewer et al., 2016), a conclusion of lack of evidence of the targeted anti-tumour activity is reached if 6 or fewer pCRs are observed out of [redacted]. The TV and LRV values may be revised based on emerging data. The futility criterion at interim is calculated such that interim results would lead to a predictive probability of a stop decision at final analysis being at least [redacted]. Moreover, the ultimate Go No-Go decision will be based on totality of the data including, but not limited to, efficacy, safety, and biomarker data.

6 REFERENCES

Clopper CJ, Pearson ES. The use of confidence or fiducial limits illustrated in the case of the binomial, Biometrika 1934; 26:404–416.

Miettinnen O, Nurminen N. Comparative analysis of two rates. Stat Med. 1985; 4:213-226

Cottrell, T. R, Thompson ED, Forde P M, Stein J E, Duffield A S, Anagnostou V, Rekhtman N, Anders R A, Cuda J D, Illei P B, Gabrielson E, Askin F B, Niknafs N, Smith K N, Velez M J, Sauter J L, Isbell J M, Jones D R, Battafarano R J, Yang S C, Danilova L, Wolchock J D, Topalian S L, Velculescu V E, Pardoll D M, Brahmer J R, Hellmann M D, Chaft J E, Cimino-Mathews A, Taube J M. Pathologic features of response to neoadjuvant anti-PD-1 in resected non-small-cell lung carcinoma: a proposal for quantitative immune-related pathologic response criteria (irPRC). Annals of Oncology 2018 ; 29.8: 1853-1860

Eisenhauer EA, Therasse P, Bogaerts J, Schwartz LH, Sargent D, Ford R, et al. New response evaluation criteria in solid tumours: Revised RECIST guidelines (version 1.1). European Journal of Cancer 2009; 45:228-47.

Frewer P, Mitchell P, Watkins C, Matcham J. Decision-making in early clinical drug development. Pharma Statist. 2016; 15:255-263.

Lalonde R L , Kowalski K G, Hutmacher M M, Ewy W, Nichols D J, Milligan P A, Corrigan B W, Lockwood P A, Marshall S A, Benincosa L J, Tensfeldt T G, Parivar K, Amantea M, Glue P, Koide H, Miller R. Model-based drug development. Clin Pharmacol Ther. 2007 Jul; 82(1):21-32

Travis et al, 2020 IASLC Multidisciplinary Recommendations for Pathologic Assessment of Lung. Cancer Resection Specimens After Neoadjuvant Therapy. J Thorac Oncol. 2020 May;15(5):709-740. doi:10.1016/j.jtho.2020.01.005.

7 APPENDICES

Appendix A RECIST

Derivation of RECIST Visit Responses

For all patients, the RECIST tumour response data is used to determine each patient's visit response according to RECIST version 1.1. It is also used to determine if and when a patient has progressed in accordance with RECIST and their best objective response to study treatment.

RECIST assessments will be performed according to the following schedule:

1 Baseline scan – performed within 28 days prior to and as close as possible to randomisation.

2 Neoadjuvant follow-up scan – at Cycle 2 and acquired upon completion of neoadjuvant period, i.e. end of neoadjuvant treatment if applicable, and prior to surgery.

3 First post-surgical scan – acquired 5 weeks (+/- 2 weeks) after surgery and prior to, but as close as possible to, the start of adjuvant study interventions. For subjects taking optional post-surgery radiotherapy, this scan should be performed prior to starting radiotherapy.

4 Post-surgical follow-up scans –

- Follow-up scans are acquired Q12W ± 1 week (relative to the date of surgery) until week 48;
- Then Q24W ± 2 weeks (relative to the date of surgery) until week 192 (approximately 4 years);
- And then Q48W ± 2 weeks (relative to the date of surgery) thereafter, until RECIST 1.1-defined radiological PD, consent withdrawal, death, or study completion as determined by Sponsor.

For patients who do not have surgery and therefore will not have a first post-surgical scan or adjuvant treatment, follow-up scans are acquired Q12±1 week (relative to the date of the pre-planned surgery) until week 48 and then Q24±2 weeks (relative to the date of pre-planned surgery) until week 192 (approximately 4 years) and then Q48±2 weeks (relative to the date of pre-planned surgery). These follow-up scans will use the original neoadjuvant screening as a baseline scan for RECIST 1.1 assessments.

If a pre-planned surgery date is not available for planning RECIST 1.1 follow-up scans on patients who did not undergo surgery (for reasons other than PD), the follow-up schedule will use 40 days after the last study drugs administration as the date of pre-planned surgery.

If an unscheduled assessment is performed, and the patient has not progressed, every attempt is made to perform the subsequent assessments at their scheduled visits. This schedule is followed in order to minimise any unintentional bias caused by some patients being assessed at a different frequency than other patients.

From the investigator's review of the imaging scans, the RECIST tumour response data is used to determine each patient's visit response according to RECIST version 1.1. At each visit, patients are programmatically assigned a RECIST 1.1 visit response of CR, PR, SD or PD, using the information from target lesions (TLs), non-target lesions (NTLs) and new lesions and depending on the status of their disease compared with baseline and previous assessments. If a patient has had a tumour assessment that cannot be evaluated then the patient is assigned a visit response of not evaluable (NE) (unless there is evidence of progression in which case the response is assigned as PD). For subjects following surgery, given the surgery is considered an intervention, the lesion intervention = Yes will be marked on all subsequent RECIST eCRFs and hence only NE or PD are potential outcomes unless scaling results in SD/PR/CR.

Please refer to Table 7 for the definitions of CR, PR, SD and PD.

RECIST outcomes (i.e. EFS, ORR etc.) are calculated programmatically for the site investigator data (see last section of this Appendix) from the overall visit responses.

During the adjuvant period, the imaging schedule must be followed regardless of any delays in dosing. Patients who do not have surgery, will not have adjuvant treatment and scans for these patients will be conducted according to the post-surgical follow-up schedule relative to the date of the pre-planned surgery. Follow-up scans will use the original neoadjuvant screening scan as a baseline scan for RECIST 1.1 assessments.

Target lesions (TLs) – site investigator data

Measurable disease is defined as having at least one measurable lesion, not previously irradiated, which is ≥ 10 mm in the longest diameter (LD), (except lymph nodes which must have short axis ≥ 15 mm) with computed tomography (CT) or magnetic resonance imaging (MRI) and which is suitable for accurate repeated measurements. A patient can have a maximum of five measurable lesions recorded at baseline with a maximum of two lesions per organ (representative of all lesions involved and suitable for accurate repeated measurement) and these are referred to as target lesions (TLs). If more than one baseline scan is recorded then measurements from the one that is closest and prior to randomisation/first dose is used to define the baseline sum of TLs. It is the case that, on

occasion, the largest lesion does not lend itself to reproducible measurement. In which circumstance the next largest lesion, which can be measured reproducibly, is selected.

All other lesions (or sites of disease) not recorded as TL are identified as non-target lesions (NTLs) at baseline. Measurements are not required for these lesions, but their status are followed at subsequent visits.

Note: For patients who do not have measurable disease at entry (i.e. no TLs) but have non-measurable disease, evaluation of overall visit responses are based on the overall NTL assessment and the absence/presence of new unequivocal lesions (see section below for further details). If a patient does not have measurable disease at baseline then the TL visit response is Not Applicable (NA).

Table 7 TL Visit Responses (RECIST 1.1)

Visit Responses	Description
Complete response (CR)	Disappearance of all TLs. Any pathological lymph nodes selected as TLs must have a reduction in short axis to <10mm.
Partial response (PR)	At least a 30% decrease in the sum of diameters of TLs, taking as reference the baseline sum of diameters as long as criteria for PD are not met.
Progressive disease (PD)	A ≥ 20% increase in the sum of diameters of TLs and an absolute increase of ≥ 5mm, taking as reference the smallest sum of diameters pre-surgery since treatment started including the baseline sum of diameters.
Stable disease (SD)	Neither sufficient shrinkage to qualify for PR nor sufficient increase to qualify for PD.
Not evaluable (NE)	Only relevant in certain situations (i.e. if any of the TLs were not assessed or not evaluable or had a lesion intervention at this visit). Note: If the sum of diameters meets the progressive disease criteria, progressive disease overrides not evaluable as a TL response. If no PD, subjects with surgery are considered having an intervention and hence a response of NE unless scaling results in SD/PR/CR.
Not Applicable (NA)	No TLs are recorded at baseline.

Rounding of TL data

For calculation of PD and PR for TLs percentage changes from baseline and previous minimum are rounded to one d.p. before assigning a TL response. For example 19.95% is rounded to 20.0% but 19.94% is rounded to 19.9%

Missing TL data

For a visit to be evaluable then all TL measurements are recorded. However, a visit response of PD is still assigned if any of the following occurred

- A new unequivocal lesion, or a new equivocal lesion, converted to unequivocal during a follow-up visit, is recorded,
- A NTL visit response of PD is recorded,
- The sum of TLs is sufficiently increased to result in a 20% increase, and an absolute increase of ≥ 5mm, from nadir even assuming the non-recorded TLs have disappeared.

Note: the nadir can only be taken from assessments where all the TLs had a LD recorded. If a particular TL measurement is missing, progression can still be assessed by assuming the TL LD = 0mm. Nadir is classed as the prior smallest sum of diameters pre-surgery since treatment started including the baseline sum of diameters.

If the TL visit response is not recorded as PD, then the TL visit response is NE.

If there is at least one TL measurement missing and a visit response of PD cannot be assigned, the visit response is NE.

If all TL measurements are missing then the TL visit response is NE. Overall visit response is also NE, unless there is a progression of non-TLs or new unequivocal lesions, in which case the response is PD.

Lymph nodes

For lymph nodes, if the size reduces to < 10mm then these are considered non-pathological. However, a size is still given and this size is still used to determine the TL visit response as normal. In the special case where all lymph nodes are < 10mm and all other TLs are 0mm then although the sum may be > 0mm the calculation of TL response should be over-written as a CR. Note that deviations to this approach, such as counting such diameters as 0mm are not recommended.

TL visit responses subsequent to CR

Only CR, PD or NE can follow a CR. If a CR has occurred then the following rules at the subsequent visits must be applied:

- If all lesions meet the CR criteria (i.e. 0mm or < 10mm for lymph nodes) then response is set to CR irrespective of whether the criteria for PD of TL is also met i.e. if a lymph node short axis increases by 20% but remains < 10mm.

- If some lesion measurements are missing but all other lesions meet the CR criteria (i.e. 0mm or < 10mm for lymph nodes) then response is set to NE irrespective of whether the criteria for PD of TL is also met i.e. if a lymph node short axis increases by 20% but remains < 10mm.
- If not all lesions are missing, and those that are non-missing do not meet the CR criteria (i.e. a pathological lymph node selected as TL has short axis ≥ 10mm or the reappearance of previously disappeared lesion), then response is set to PD.
- If all lesions are missing the response is set to NE.

TL too big to measure

If a TL becomes too big to measure this is indicated in the database and a size ('*x*') above which it cannot be accurately measured is recorded. If using a value of *x* in the calculation of TL response does not give an overall visit response of PD, then this is flagged and reviewed by the study team blinded to treatment assignment. It is expected that a visit response of PD remains in the vast majority of cases.

TL too small to measure

If a TL becomes too small to measure then this is indicated as such on the case report form and a value of 5mm is entered into the database and used in TL calculations. However a smaller value is used if the radiologist has not indicated 'too small to measure' on the case report form and has entered a smaller value that can be reliably measured. If a TL response of PD results (at a subsequent visit) then this is reviewed by the study team blinded to treatment assignment.

Lesions that split in two

If a TL splits in two, then the LDs of the split lesions should be summed and reported as the LD for the lesion that split.

Lesions that merge

If two TLs merge, then the LD of the merged lesion should be recorded for one of the TL sizes and the other TL size should be recorded as 0 mm.

Change in method of assessment of TLs

CT, MRI and clinical examination are the only methods of assessment that can be used within a trial, with CT and MRI being the preferred methods and clinical examination only used in special cases. If a change in method of assessment occurs, between CT and MRI this will be considered acceptable and no adjustment within the programming is needed.

If a change in method involves clinical examination (e.g., CT changes to clinical examination or vice versa), any affected lesions should be treated as missing.

Irradiated lesions/lesion intervention

The surgical resection in this study will be considered a lesion intervention and should be treated as having intervention for the remainder of the study noting that an intervention will most likely shrink the size of tumors.

After surgery the investigator must record lesion measurements as usual, but also record a TL Intervention for all visits after the surgery. In the case of a complete surgical resection, a lesion measurement of 0 mm will be recorded with the TL intervention flag. If an unequivocal lesion, or a new equivocal lesion, converted to unequivocal during a follow-up visit, appears in the same position as one that "disappears" and is confirmed during a follow-up visit, it is considered a New Unequivocal Lesion and PD.

Table 8 Example: RECIST visit responses following complete resection

	**Baseline**	**Neoadjuvant follow-up scan**	**Complete Surgical Resection**	**1st post-surgery scan**	**2nd post-surgery scan**	**3rd post-surgery scan**
Sum of diameters	80 mm	50 mm		0 mm	0 mm	0 mm<br><br>(lesion that appears in same position as lesion that has disappeared is recorded as new lesion)
TL<br><br>Intervention Flag	No	No		Yes	Yes	Yes

VISIT RESPONSE	-	PR		NE	NE	PD (due to new unequivocal lesion otherwise it would be NE)

In the scenario where a subject has a partial resection, it is marked as having intervention and assessed as NE at all subsequent visits until there's clear progression by increase in sum of diameters of >=20% and 5 mm over nadir or new unequivocal lesion. Nadir can only be taken from assessments where all the TLs had a LD recorded or scaling applied. Nadir is classed as the smallest sum of diameters pre-surgery since treatment started, including the baseline sum of diameters.

Table 9 Example: RECIST visit responses following partial resection

	Baseline	Neoadjuvant follow-up scan	Partial Surgical Resection	1st post-surg scan	2nd post-surg scan	3rd post-surg scan
Sum of diameters	80 mm	50 mm		4 mm	4 mm	66 mm
TL Intervention Flag	No	No		Yes	Yes	Yes
VISIT RESPONSE	-	PR		NE	NE	PD

Table 10 Example: RECIST visit responses following partial resection

	Baseline	Neoadjuvant follow-up scan	Partial Surgical Resection	1st post-surg scan	2nd post-surg scan	3rd post-surg scan
Sum of diameters	80 mm	50 mm		4 mm	4 mm	51 mm
TL Intervention Flag	No	No		Yes	Yes	Yes
VISIT RESPONSE	-	PR		NE	NE	NE

Any TL (including lymph nodes), which has had intervention on the study should be handled in the following way in programming:

- Step 1: the diameters of the TLs (including the lesions that have had intervention) will be summed and the calculation will be performed in the usual manner. If the visit response is PD, this will remain as a valid response category.

- Step 2: If there was no evidence of progression after step 1, treat the lesion diameter (for those lesions with intervention) as missing and if ≤ 1/3 of the TLs have missing measurements then scale up as described in the 'Scaling' section below. If the scaling results in a visit response of PD then the subject would be assigned a TL response of PD.

- Step 3: If, after both steps, PD has not been assigned then, if appropriate (i.e. if ≤ 1/3 of the TLs have missing measurements), the scaled sum of diameters calculated in step 2 should be used, and PR or SD then assigned if applicable as the visit response. Subjects with intervention are evaluable for CR as long as all non-intervened lesions are 0 (or <10mm for lymph nodes) and the lesions that have been subject to intervention have a value of 0 (or <10mm for lymph nodes) recorded. If scaling up is not appropriate due to too few non-missing measurements then the visit response will be set as NE.

At subsequent visits, the above steps will be repeated to determine the TL and overall visit response.

Scaling (applicable only for irradiated lesions/lesion intervention)

If > 1/3 of TL measurements are missing (because of intervention) then the TL response will be NE, unless the sum of diameters of non-missing TL would result in PD (i.e. if using a value of 0 for missing lesions, the sum of diameters has still increased by 20% or more compared to nadir and the sum of TLs has increased by ≥5mm from nadir).

If ≤ 1/3 of the TL measurements are missing (because of intervention) then the results will be scaled up (based on the sizes at the nadir visit to give an estimated sum of diameters) and this will be used in calculations; this is equivalent to comparing the visit sum of diameters of the non-missing lesions to the nadir sum of diameters excluding the lesions with missing measurements.

Example of scaling

Lesion 5 is missing at the follow-up visit; the nadir TL sum including lesions 1-5 was 74 mm.

The sum of lesions 1-4 at the follow-up is 68 mm. The sum of the corresponding lesions at the nadir visit is 62 mm.

Scale up as follows to give an estimated TL sum of 81 mm:
68 x 74 / 62 = 81 mm.

CR will not be allowed as a TL response for visits where there is missing data. Only PR, SD or PD (or NE) could be assigned as the TL visit response in these cases. However, for visits with ≤ 1/3 lesion assessments not recorded, the scaled up sum of TLs diameters will be included when defining the nadir value prior to surgery for the assessment of progression.

Non-target lesions (NTLs) and new lesions – site investigator data.

At each visit, the investigator is to record an overall assessment of the NTL response. This section provides the definitions of the criteria used to determine and record overall response for NTL at the investigational site at each visit.

NTL response is derived based on the investigator's overall assessment of NTLs as follows:

Table 11 NTL Visit Responses

Visit Responses	Description
Complete response (CR)	Disappearance of all NTLs present at baseline with all lymph nodes non-pathological in size (<10 mm short axis).
Progressive disease (PD)	Unequivocal progression of existing NTLs. Unequivocal progression may be due to an important progression in one lesion only or in several lesions. In all cases, the progression MUST be clinically significant for the physician to consider changing (or stopping) therapy.
Non-CR/Non-PD	Persistence of one or more NTLs with no evidence of progression.
Not evaluable (NE)	Only relevant when one or some of the NTLs were not assessed and, in the investigator's opinion, they are not able to provide an evaluable overall NTL assessment at this visit.<br>Note: For patients without TLs at baseline, this is relevant if any of the NTLs were not assessed at this visit and the progression criteria have not been met.<br>Note: subjects with intervention and no PD will be classed as NE.
Not Applicable (NA)	Only relevant if there are no NTLs at baseline.

To achieve 'unequivocal progression' on the basis of NTLs, there must be an overall level of substantial worsening in non-target disease such that, even in the presence of SD or PR in TLs, the overall tumour burden has increased sufficiently to merit a determination of disease progression. A modest 'increase' in the size of one or more NTLs is usually not sufficient to qualify for unequivocal progression status.

Details of any new lesions are also recorded with the date of assessment. The presence of one or more new lesions is assessed as progression.

A lesion identified at a follow up assessment in an anatomical location that was not scanned at baseline is considered a new lesion and indicates disease progression.

New lesions will be identified at each visit and recorded on the CRF. Biopsy data will be collected for new lesions to determine if the new lesion is a new primary malignancy.

The finding of a new lesion should be unequivocal: i.e. not attributable to differences in scanning technique, change in imaging modality or findings thought to represent something other than tumour.

New lesions are identified via a Yes/No tick box. The absence and presence of new lesions at each visit are listed alongside the TL and NTL visit responses.

A new unequivocal lesion indicates progression so the overall visit response is PD irrespective of the TL and NTL response.

For the EFS and DFS endpoints, pathological confirmation from biopsied lesions, if performed according to investigators judgement and local practice, will also be taken into consideration (as applicable). A new malignancy, that is not NSCLC, as confirmed by pathology, is not considered an EFS/DFS event. In the absence of pathology confirmation, a new unequivocal lesion will be considered an EFS/DFS event.

If the question 'Any new lesions since baseline' has not been answered with Yes or No and the new lesion details are blank, this is not evidence that no new lesions are present, but should not overtly affect the derivation. This scenario (i.e. whereby new lesion response is NE), should only occur in exceptional cases, as missing data for the new lesion field should always be queried.

Symptomatic progression is not a descriptor for progression of NTLs: it is a reason for stopping study therapy and is not included in any assessment of NTLs.

Patients with 'symptomatic progression' requiring discontinuation of treatment without objective evidence of disease progression at that time should continue to undergo tumour assessments where possible until objective disease progression is observed.

Overall visit response – site investigator data

Table defines how the previously defined TL and NTL visit responses are combined with new lesion information to give an overall visit response.

Table 12 Overall visit responses

TARGET	NON-TARGET	NEW LESIONS*	OVERALL VISIT RESPONSE
CR	CR or NA	No (or NE)	**CR**
CR	Non-CR/Non-PD or NE	No (or NE)	**PR**
PR	Non-PD or NE or NA	No (or NE)	**PR**
SD	Non-PD or NE or NA	No (or NE)	**SD**
PD	Any	Any	**PD**
Any	PD	Any	**PD**
Any	Any	Yes	**PD**
NE	Non-PD or NE or NA	No (or NE)	**NE**
NA	CR	No (or NE)	**CR**
NA	Non-CR/Non-PD	No (or NE)	**SD**
NA	NE	No (or NE)	**NE**
NA	NA	No (or NE)	**NED**

*One or more new unequivocal lesions of the primary disease (NSCLC). If all lesions are pathologically confirmed to be new primary malignancies (not NSCLC) then PD by new lesions is not met.

Signature Page for VV-RIM-07535980 System v1.0
D9077C00001 SAP v4.0

Approve: Document Level Task<br>Verdict: Approved	Na Zhang<br>Content Approval<br>04-Dec-2024 15:55:40 GMT+0000

Signature Page for VV-RIM-07535980 System v1.0
D9077C00001 SAP v4.0
