# Supplementary material for: Perioperative durvalumab plus chemotherapy plus new agents for resectable non-small-cell lung cancer: the platform phase 2 NeoCOAST-2 trial
Source: Nat Med. 2025 May 31;31(8):2788–96. doi: 10.1038/s41591-025-03746-z (PMC12353838; doi:10.1038/s41591-025-03746-z)
Supplement: Supplementary file 2 — Reporting Summary [file 41591_2025_3746_MOESM2_ESM.pdf]

nature portfolio

Corresponding author(s): Tina Cascone

Last updated by author(s): Apr 25, 2025

Reporting Summary

Nature Portfolio wishes to improve the reproducibility of the work that we publish. This form provides structure for consistency and transparency in reporting. For further information on Nature Portfolio policies, see our Editorial Policies and the Editorial Policy Checklist.

Statistics

For all statistical analyses, confirm that the following items are present in the figure legend, table legend, main text, or Methods section.

n/a	Confirmed	
☐	☒	The exact sample size (*n*) for each experimental group/condition, given as a discrete number and unit of measurement
☐	☒	A statement on whether measurements were taken from distinct samples or whether the same sample was measured repeatedly
☒	☐	The statistical test(s) used AND whether they are one- or two-sided<br>*Only common tests should be described solely by name; describe more complex techniques in the Methods section.*
☒	☐	A description of all covariates tested
☒	☐	A description of any assumptions or corrections, such as tests of normality and adjustment for multiple comparisons
☐	☒	A full description of the statistical parameters including central tendency (e.g. means) or other basic estimates (e.g. regression coefficient) AND variation (e.g. standard deviation) or associated estimates of uncertainty (e.g. confidence intervals)
☒	☐	For null hypothesis testing, the test statistic (e.g. *F*, *t*, *r*) with confidence intervals, effect sizes, degrees of freedom and *P* value noted<br>*Give P values as exact values whenever suitable.*
☒	☐	For Bayesian analysis, information on the choice of priors and Markov chain Monte Carlo settings
☒	☐	For hierarchical and complex designs, identification of the appropriate level for tests and full reporting of outcomes
☒	☐	Estimates of effect sizes (e.g. Cohen's *d*, Pearson's *r*), indicating how they were calculated

Our web collection on statistics for biologists contains articles on many of the points above.

Software and code

Policy information about availability of computer code

Data collection	No software were used in the collection of the data
Data analysis	SAS version 9.4 was used for all analyses

For manuscripts utilizing custom algorithms or software that are central to the research but not yet described in published literature, software must be made available to editors and reviewers. We strongly encourage code deposition in a community repository (e.g. GitHub). See the Nature Portfolio guidelines for submitting code & software for further information.

Data

Policy information about availability of data

All manuscripts must include a data availability statement. This statement should provide the following information, where applicable:

- Accession codes, unique identifiers, or web links for publicly available datasets
- A description of any restrictions on data availability
- For clinical datasets or third party data, please ensure that the statement adheres to our policy

Data underlying the findings described in this manuscript may be obtained in accordance with AstraZeneca's data sharing policy described at: https://astrazenecagrouptrials.pharmacm.com/ST/Submission/Disclosure.Data for studies directly listed on Vivli can be requested through Vivli at www.vivli.org. Data for studies not listed on Vivli could be requested through Vivli at https://vivli.org/members/enquiries-about-studies-not-listed-on-the-vivli-platform/. The AstraZeneca Vivli member page is also available outlining further details: https://vivli.org/ourmember/astrazeneca/.

Research involving human participants, their data, or biological material

Policy information about studies with human participants or human data. See also policy information about sex, gender (identity/presentation), and sexual orientation and race, ethnicity and racism.

Reporting on sex and gender	Data on sex were collected via the Case Report Form as allowed by local regulatory guidelines and are reported in the patient baseline characteristics table. Analyses reported in this paper are not controlled for sex. No further sex-based analysis were conducted as a significant difference in pathological response and safety were not expected between male and female patients.
Reporting on race, ethnicity, or other socially relevant groupings	Data on race were collected via the Case Report Form as allowed by local regulatory guidelines using standard reporting categories and are reported in the patient baseline characteristics table. Analyses were not controlled for race or ethnicity.
Population characteristics	Newly diagnosed patients with early stage resectable NSCLC, without actionable genomic alterations. The median age in arm 1 was 66.5 (30-79), in arm 2 was 66.0 (48-83), and arm 4 was 65.0 (38-81).
Recruitment	Patients were recruited by participating cancer treatment centers. Eligible patients were assessed by multidisciplinary providers; patients who then signed informed consent were considered 'enrolled' patients per protocol and underwent the screening process. Each enrolled patient was identified by a unique E-code. Enrolled patients provided tumor samples (newly acquired or archival tumor tissue) to determine biomarker specification and provided information regarding the study eligibility criteria, via a computer system. Patients then receive a randomization number and are centrally assigned to randomized study interventions using a web-based Interactive Response System (IRT). Every attempt was made to randomize patients as close as possible to Day 1 of Cycle 1 and not more than 72 hours (3 days) prior to Day 1. If a patient withdrew from the study, then his/her enrollment/randomization code was not reused. Withdrawn patients were not replaced. There was no potential for self-selection bias or other biases.
Ethics oversight	The study was performed in accordance with consensus ethical principles derived from international guidelines including the Declaration of Helsinki and Council for International Organizations of Medical Sciences (CIOMS) International Ethical Guidelines, applicable International Council for Harmonisation (ICH) Good Clinical Practice (GCP) Guidelines, and all applicable laws and regulations. The study included a Safety Review Committee that conducted safety reviews of all enrolled patients throughout the study. This study was funded by AstraZeneca. The study protocol, protocol amendments, informed consent form, investigator brochure, and other relevant documents were reviewed and approved by the Institutional Review Board/ Ethics Committee at each participating center: Belgium - UZ Leuven. Canada - McGill University Health Centre; Centre hospitalier de l'Université de Montréal; Alberta Health Services (AHS) - Cross Cancer Institute. France - CHU de Bordeaux - Hopital Saint André; Centre Hospitalier de Cornouaille; CHU Dupuytren; Hopital Foch; CHU Rouen Hopital; Hopital d'Instruction des Armées Sainte Anne; Centre Hospitalier D'Avignon - Hopital Henri Duffaut - Médecine Interne Onco-Hématolo. Hungary - Bacs-Kiskun Varmegyei Oktatakorhaz; Tudogyogyintezet Torokbalint; Fejér Vármegyei Szent György Egyetemi Oktató Kórház; Szent Borbala Korhaz. Ireland - University Hospital Galway; Mater Misercordiae Hospital. Italy - Regina Elena; Istituto Nazionale dei Tumori ; IFO; IRCCS; Ospedale San Gerardo; ASST di Monza; IRCCS; Istituto Clinico Humanitas Rozzano; IRCCS; IOV; IRCCS; Ospedale S.Maria della Misericordia; AO di Perugia S.C. Oncologia Medica ; Ospedale Policlinico San Martino; IRCCS; SO di Cisanello; AOU Pisana; AOU Careggi UO Radioterapia ; Ospedale Niguarda; ASST Grande Ospedale Metropolitano Niguarda; ASST Spedali Civili di Brescia. Portugal - Hospital da Luz; Centro Hospitalar e Universitário Lisboa Central - Hospital Santo António dos Capuchos; Instituto Português Oncologia Francisco Gentil do Porto; Instituto Português de Oncologia de Lisboa Francisco Gentil. South Korea - Asan Medical Center; Seoul National University Hospital - Department of Internal; The Catholic University of Korea; St. Vincent's Hospital - Oncology; CHA Bundang Medical Center; CHA University; Inje University Haeundae Paik Hospital. Spain - Hospital Teresa Herrera (CHUAC); Hospital Universitario Puerta De Hierro De Majadahonda; H.U.M. de Terrassa; H.U. Sant Joan de Reus; Hospital Clínico San Carlos; H. Clínico de Valencia; Hospital Universitario Virgen De La Macarena; Hospital Regional Universitario de Malaga; Hospital Clinic De Barcelona; Fundacion Jimenez Diaz; Hospital General Universitario De Alicante. Taiwan - National Cheng Kung University Hospital; Taipei Medical University - Shuang Ho Hospital - Pulmonology. Turkey - Goztepe Prof. Dr. Suleyman Yalcin Sehir Hastanesi Tibbi Onkoloji. United States of America - MD Anderson Cancer Center; Cleveland Clinic - Medical Oncology/ Hematology; John Hopkins Medicine - Hematology/oncology; US Oncology - Virginia Cancer Specialists, P.C. (VCS); Cleveland Clinic Florida - Martin Health.

Note that full information on the approval of the study protocol must also be provided in the manuscript.

Field-specific reporting

Please select the one below that is the best fit for your research. If you are not sure, read the appropriate sections before making your selection.

☒ Life sciences ☐ Behavioural & social sciences ☐ Ecological, evolutionary & environmental sciences

For a reference copy of the document with all sections, see nature.com/documents/nr-reporting-summary-flat.pdf

Life sciences study design

All studies must disclose on these points even when the disclosure is negative.

Sample size	A decision framework, as outlined in the works of Lalonde et al. (2007) and Frewer et al. (2016), was built around pCR rate to calculate the sample size per treatment arm. The sample size of up to 70 patients per treatment arm was calculated such that there was at least an 80%

	chance of making a go decision at the final analysis if the true pCR rate was at target value for the respective treatment arm. The sample size was not based on type I error and power considerations. Decisions related to stopping criteria were at the discretion of the sponsor and were based on emerging efficacy, safety, and tolerability data.
Data exclusions	No data were excluded.
Replication	No replication was performed as this was a clinical trial.
Randomization	A randomization method with dynamically changing allocation ratio of treatment assignment was employed to account for fluctuations in the number of enrolling treatment arms over the course of the study and to allow an increase in enrollment to one or more treatment arms at the discretion of the Sponsor and/or recommendation by the Safety Review Committee. Where there was only a single arm enrolling, all patients were allocated to that arm. The actual treatment given to patients was determined by the randomization scheme which was produced by a computer software program that incorporates a standard procedure for generating randomization numbers. A blocked randomization was generated, and randomization was balanced within the IRT at the central level.
Blinding	This is an open-label study; however, the specific study interventions were assigned using an IRT. The site contacted the IRT prior to the start of study interventions administration for each patient. The site recorded the study intervention assignment on the applicable eCRF, if required. Potential bias was reduced by the use of central randomization.

Reporting for specific materials, systems and methods

We require information from authors about some types of materials, experimental systems and methods used in many studies. Here, indicate whether each material, system or method listed is relevant to your study. If you are not sure if a list item applies to your research, read the appropriate section before selecting a response.

Materials & experimental systems

n/a	Involved in the study
☒	☐ Antibodies
☒	☐ Eukaryotic cell lines
☒	☐ Palaeontology and archaeology
☒	☐ Animals and other organisms
☐	☒ Clinical data
☒	☐ Dual use research of concern
☒	☐ Plants

Methods

n/a	Involved in the study
☒	☐ ChIP-seq
☒	☐ Flow cytometry
☒	☐ MRI-based neuroimaging

Clinical data

Policy information about clinical studies

All manuscripts should comply with the ICMJE guidelines for publication of clinical research and a completed CONSORT checklist must be included with all submissions.

Clinical trial registration	NCT05061550
Study protocol	Uploaded as a supplementary file
Data collection	Data collection from occurred at hospitals, clinical trial sites, clinics, or infusion centers entered via electronic data capture. All methods of data collection were reviewed and ensured patient privacy was upheld at all times. Data collection for arm 1 started 03May2022, for arm 2 started 12Aug2022, and arm 4 started 23Aug2023. Data collection for all three arms were collected until 19December2024.
Outcomes	The primary objective of the study was to evaluate the antitumor activity of neoadjuvant treatment administered prior to surgery, in terms of pathologic complete response (pCR) and to assess the safety and tolerability of neoadjuvant and adjuvant treatment. Secondary objectives included assessing the antitumor activity of neoadjuvant treatment administered prior to surgery in terms of major pathologic response (mPR) and assessing the feasibility of receiving the planned surgical tumor resection in patients receiving neoadjuvant treatment.<br>Pathological response was defined by the absence of viable tumor cells in the resected lung cancer specimen and all sampled regional lymph nodes (pCR) or as having less than or equal to 10% viable tumor cells in resected tissue (mPR), as determined by a central Blinded Independent Pathology Review (BIPR) using IASLC 2020 criteria. Pathological responses were collected locally and subsequently collated and reported by central blinded independent pathology review. Primary tumors and sampled lymph nodes were assessed by central pathology review for the percentage of residual viable tumor that was identified on routine hematoxylin and eosin staining. Central pathology assessments of pCR and mPR were performed according to International Association for the Study of Lung Cancer (IASLC) 2020 criteria. Patients were considered to have no response if they were not eligible for assessment or if a surgical specimen was not available.<br>Safety and tolerability assessments included monitoring adverse events (AEs), vital signs, and clinical laboratory parameters throughout the study. AEs were recorded from the time of informed consent signing through the treatment period and during the safety follow-up 90 days post-final dose, unless events occurring after this period were considered late-onset toxicities related to study interventions. The severity of AEs was evaluated according to the National Cancer Institute's Common Terminology Criteria for Adverse Events (NCI CTCAE) version 5.0, and causal relationship between study drug and each AE was assessed by the investigator.

In Arm 4, an independent ILD Adjudication Committee reviewed all potential interstitial lung disease (ILD)/pneumonitis cases to ensuring independent evaluation through systematic additional data collection for adjudicated cases. This additional data included detailed medical histories, diagnostic evaluations, treatments, and outcomes related to the event, triggered by a predefined list of preferred terms. Safety oversight was further ensured by a Safety Review Committee (SRC), composed of Investigators, the Sponsor, and an independent chair, which conducted early safety evaluations after the first 10 patients in each treatment arm completed two cycles of neoadjuvant treatment. They also reviewed safety data after the first 10 patients underwent surgery and had 21 days of follow-up. The SRC met approximately every six months afterwards to continually assess the safety and tolerability of both the neoadjuvant and adjuvant regimens until all patients had the opportunity to undergo surgery and those who underwent surgery completed at least six months of adjuvant treatment.
Feasibility of surgery was defined as having the planned surgical resection within 40 days after the last dose of neoadjuvant treatment. Computed tomography (CT) and positron emission tomography (PET) were used for staging within 30 days before surgical evaluation. In case of CT-enlarged or PET-positive lymph nodes, tissue confirmation was recommended. The European Society of Medical Oncology and National Comprehensive Cancer Network guidelines for the resectability of NSCLC were followed. The Clavien-Dindo assessment was utilized for grading postoperative complications.

Plants

Seed stocks

NA

Novel plant genotypes

NA

Authentication

NA
